# Three-Coordinate Iron(0) Complex-Catalyzed Regioselective C–H Alkylation of Indole Derivatives

**DOI:** 10.1021/jacs.4c17316

**Published:** 2025-02-17

**Authors:** Zi-Jing Zhang, Stéphane Golling, Silvia Cattani, Xinran Chen, Lutz Ackermann

**Affiliations:** Wöhler Research Institute for Sustainable Chemistry (WISCh), Georg-August-Universität Göttingen, Tammannstraße 2, 37077 Göttingen, Germany

## Abstract

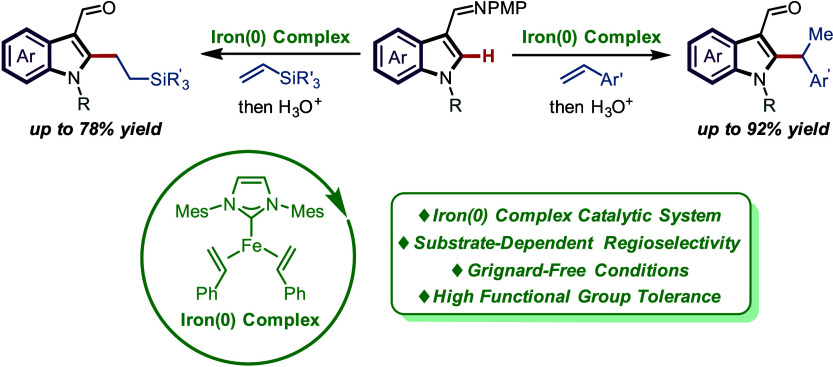

The synthesis of alkylated indoles, which are key intermediates for various drugs and bioactive molecules, is of great importance. However, most reports on the synthesis of functionalized indoles use toxic and expensive 4d or 5d metal catalysts, limiting the further application of these methods. Herein, we disclose a versatile regioselective C–H alkylation of indole derivatives using a well-defined three-coordinate iron(0) complex. Neither Grignard reagents nor additional additives are required, making the reaction sustainable, environmentally friendly, and compatible with a broad variety of functional groups to afford C2-alkylated indoles in high yields. In addition, by variation of the aryl substituent on the alkene substrate to the trisubstituted silyl group, the regioselectivity of the C–H alkylation can be altered from Markovnikov to *anti*-Markovnikov. Detailed mechanistic studies further revealed the catalytic mode of reaction.

## Introduction

Alkylated indoles, privileged motifs in drug design, crop protection, and material science, have attracted extensive attention from the scientific community.^1−4^ The efficient synthesis of these compounds is rapidly expanding.^5,6^ In addition to classical protocols, direct and regioselective C–H alkylation-based protocols have recently demonstrated strong potential in the preparation of alkylated indoles.^7^ However, regioselective C–H alkylation of indole derivatives mostly requires the use of prefunctionalized organometallic compounds, alkyl halides, or alkyl carbonates as the alkyl source, which need to be prepared in advance.^8−12^ Likewise, the stoichiometric amounts of metal waste or halogen byproducts produced during the process arguably limit the application of such reactions.

**Figure 1:**
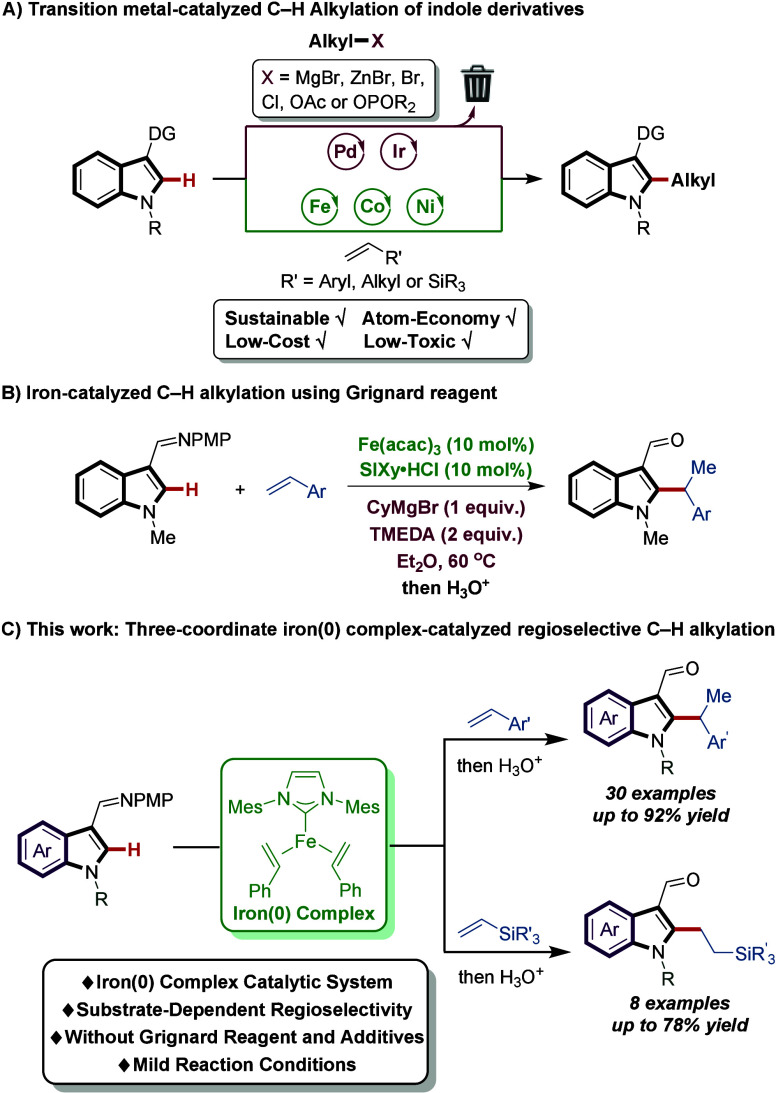
Transition-metal-catalyzed regioselective C–H alkylation of indole derivatives. A) Transition-metal-catalyzed C–H alkylation of indole derivatives. B) Iron-catalyzed C–H alkylation using Grignard reagent. C) This work: Three-coordinate iron(0) complex-catalyzed regioselective C–H alkylation.

C–H alkylation *via* direct additions of indoles onto alkenes is the most efficient and atom-economical approach to access functionalized indoles (Figure 1A).^13,14^ Despite considerable advances, these hydroarylations continue to be dominated by expensive, toxic, and less abundant 4d and 5d transition metals,^15−17^ while transformations involving more sustainable 3d metal catalysts remain limited.^18−23^ In this context, cobalt- and nickel-catalyzed^24−27^ C–H alkylation of indole derivatives has been successfully achieved by Hiyama,^28^ Yoshikai,^29,30^ Hartwig,^31^ and Ackermann,^32−34^ among others.^35−37^ In contrast, the Yoshikai group made significant strides by successfully accomplishing C–H alkylation and alkenylation of indoles using the most abundant, low-cost, and nontoxic iron^38−43^ catalyst (Figure 1B).^44^ Then, our research group developed a series of chiral *N*-heterocyclic carbene (NHC) ligands, successfully enabling high atropo- and enantioselective C–H alkylation of indole derivatives with styrenes via iron catalysis.^45,46^ However, the (super)stoichiometric amounts of Grignard reagents are necessary, which require vigorously dried solvents, inert gas atmospheres, and additional metal preactivation steps during their preparation, and translate into a considerable amount of waste, while significantly affecting the functional group tolerance of the reaction.

Recently, the synthesis and application of low-valent metal complexes have received widespread attention.^47^ In this context, Deng,^48−51^ Chirik,^52,53^ and Neidig,^54^ among others,^55−58^ have successfully synthesized low-valent iron complexes, providing insights into C–H functionalizations. Herein, we disclose a three-coordinate iron(0) complex-catalyzed regioselective C–H alkylation of indole derivatives. The C–H alkylation exhibited substrate-dependent regioselectivity by varying the steric hindrance of the alkenes and thereby obtained alkylated indole products with Markovnikov *versus anti*-Markovnikov selectivity. The efficient three-coordinate iron(0) complex allows the reaction to proceed at room temperature. The absence of Grignard reagents and additional additives makes the reaction more sustainable, step-economical, and functional-group-tolerant (Figure 1C).

## Results and Discussion

### Optimization of the Reaction Conditions

**Scheme 1:**
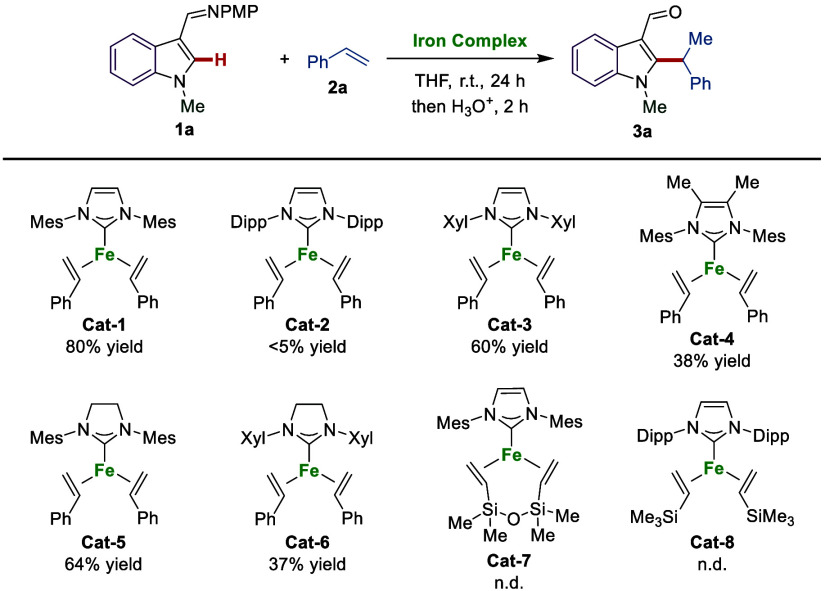
Optimization of Three-Coordinate Iron(0) Complex-Catalyzed Regioselective C–H Alkylation Reaction conditions: **1a** (0.1 mmol), **2a** (0.15 mmol), and Iron Complex (10 mol %) were stirred in THF (0.3 mL) at room temperature for 24 h under N_2_. Then HCl aq. (1 M, 1.0 mL) was added and stirred for 2 h. The ratio of b:l is >95:5 for all cases. Yields are those of the isolated products.

We initiated our studies into the regioselective C–H alkylation of indole derivative **1a** with styrene **2a** using the well-defined iron(0) complexes as catalysts (Scheme 1). Pleasingly, the desired alkylated indole **3a** was obtained in 80% yield with complete Markovnikov selectivity when using Fe(IMes)(η^2^-styrene)_2_ (**Cat-1**)^51^ as the catalyst. The reaction was also performed in environmentally friendly solvent 2-MeTHF, affording the targeted product **3a** in 74% yield. Subsequently, the structure of the NHC ligand was further probed. Changing the steric hindrance and electronic properties of the aromatic substituents on the NHC ligand (**Cat-2** and **Cat-3**) led to a diminution of the reaction efficiency. Introducing substituents at the backbone of the NHC ligand (**Cat-4**) significantly reduced the yield of the reaction. The use of NHC ligands with saturated backbones (**Cat-5** and **Cat-6**) resulted in a slight decrease in the yield of the reaction. Then, the complex containing a bis-coordinated divinyltetramethyldisiloxane (dvtms) ligand (**Cat-7**) was also probed, resulting in a complete inhibition of the reactivity, probably due to the strong coordination ability of the ligand. When we used the iron complex containing IPr and trimethyl(vinyl)silane (**Cat-8**), the desired alkylated indole **3a** was not observed.

### Substrate Scope

**Scheme 2:**
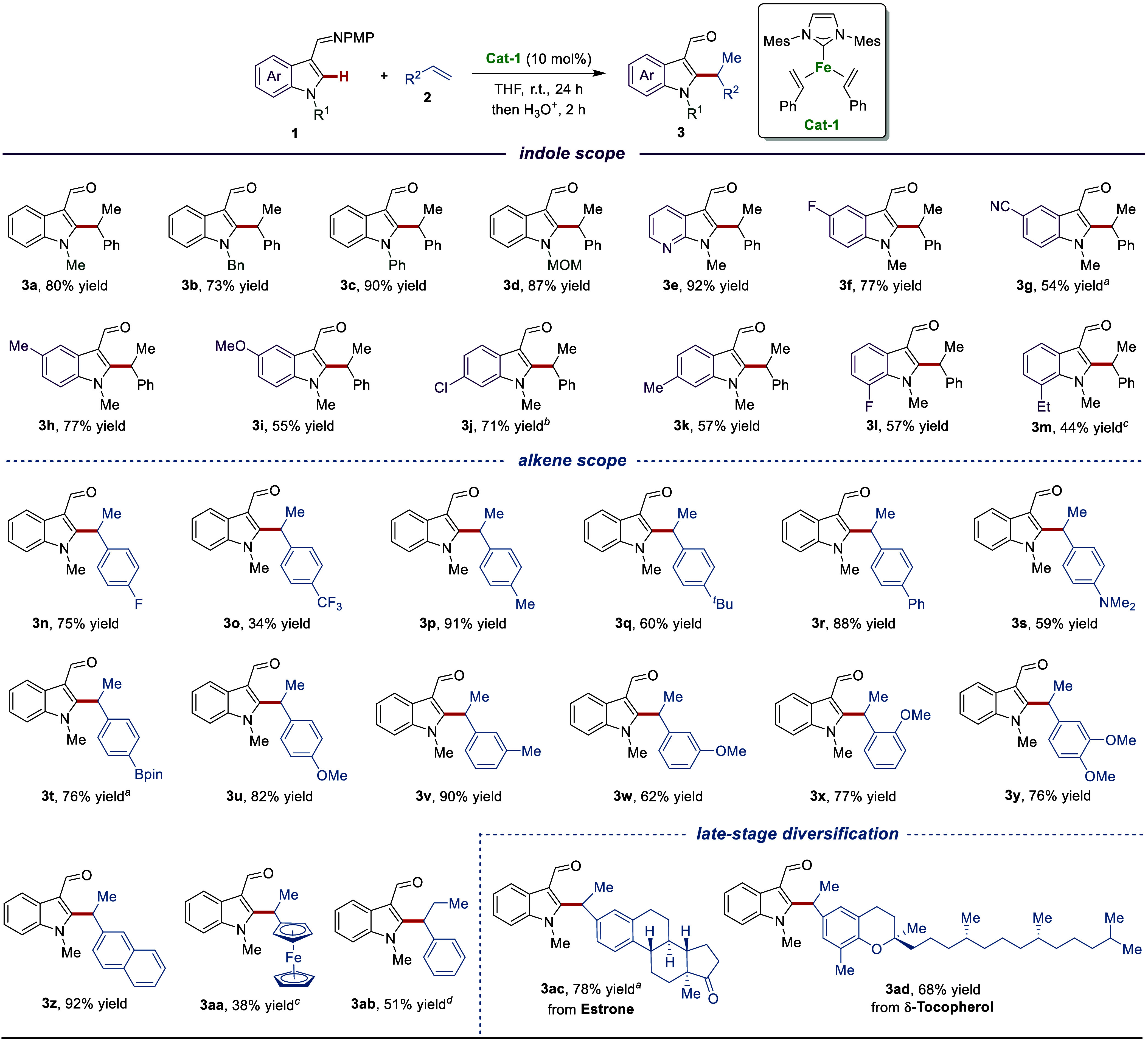
Substrate Scope for the C–H Alkylation of Substituted Indoles with Aryl Alkenes Reaction conditions: **1** (0.1 mmol), **2** (0.15 mmol), and **Cat-1** (10 mol %) were stirred in THF (0.3 mL) at room temperature for 24 h under N_2_. Then HCl aq. (1 M, 1.0 mL) was added and stirred for 2 h. The ratio of b:l is >95:5 for all cases. Yields are those of the isolated products. No product was obtained with the Grignard reagent system. 34% yield with the Grignard reagent system. 60 °C. Use *cis*-β-methylstyrene.

With the optimal three-coordinate iron(0) complex (Fe(IMes)(η^2^-styrene)_2_) in hand, we evaluated its robustness under the optimized reaction conditions (Scheme 2). Indole substrates **1** bearing a variety of *N*-substituents were first investigated. Changing the *N*-substituent of indole had a minor impact on the reaction, and all afforded the desired alkylated indole products **3a**–**3d** in excellent yields. The azaindole **1e** proved to be a suitable substrate, as well. Both electron-withdrawing and electron-donating substituents on the indole ring were well tolerated, delivering desired products **3f**–**3m** in moderate to good yields. Then, we explored the scope of viable alkenes. A variety of substituted styrenes containing either electron-withdrawing or electron-donating substituents on the *para*-, *meta*-, or *ortho*-position of the aromatic ring performed well and provided the corresponding products **3n**–**3y** in good yields. 2-Vinylnaphthalene and vinylferrocene were also compatible, affording the target products **3z** and **3aa**. It is worth noting that *cis*-β-methylstyrene effectively engaged in the reaction, yielding the desired alkylated indole **3ab** with moderate yield. Alkenes derived from estrone and δ-tocopherol were subjected to the reaction, giving products **3ac** and **3ad** in good yields. Notably, cyano and pinacol boronic ester substituents (**3g** and **3t**), which were incompatible with the Grignard reagent system, were well tolerated by the well-defined iron(0) catalyst.

**Scheme 3:**
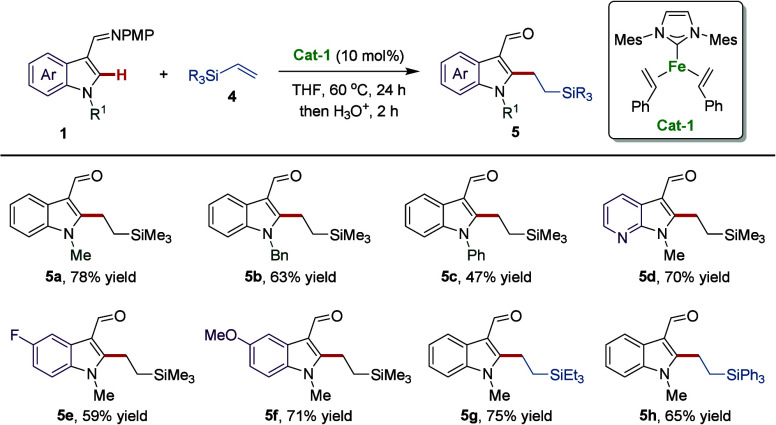
Substrate Scope for the C–H Alkylation of Substituted Indoles with Vinyl Silanes Reaction conditions: **1** (0.1 mmol), **4** (0.15 mmol), and **Cat-1** (10 mol %) were stirred in THF (0.3 mL) at 60 °C for 24 h under N_2_. Then HCl aq. (1 M, 1.0 mL) was added and stirred for 2 h. The ratio of l:b is >95:5 for all cases. Yields are those of the isolated products.

Organosilanes represent key structural motifs in numerous natural products and materials.^59,60^ However, only a limited number of approaches for the catalytic synthesis of silyl-substituted indoles are so far available.^42,61,62^ Hence, we attempted to obtain the desired silyl-substituted indoles efficiently through the direct C–H alkylation of indole derivatives and vinyl silanes catalyzed by the three-coordinate iron(0) complex. Interestingly, when we used trisubstituted(vinyl)silanes as substrates, the alkylated indoles were obtained with complete *anti*-Markovnikov selectivity. We proposed that the steric hindrance and electronic properties of the trisubstituted(vinyl)silane substrates affected the regioselectivity by a ligand-to-ligand hydrogen transfer (LLHT) process and finally led to *anti*-Markovnikov selectivity. Then, we also explored the scope of the reaction with amenable vinyl silanes (Scheme 3). Using indole derivatives with different *N*-substituents, electron-withdrawing or electron-donating substituents, the corresponding alkylated indole products **5a**–**5f** were obtained with a complete *anti*-Markovnikov selectivity. Various trialkyl(vinyl)silanes and triaryl(vinyl)silanes proved compatible with this reaction, yielding the desired products **5g** and **5h** in high yields.

### Scale-Up and Late-Stage Transformations

**Scheme 4:**
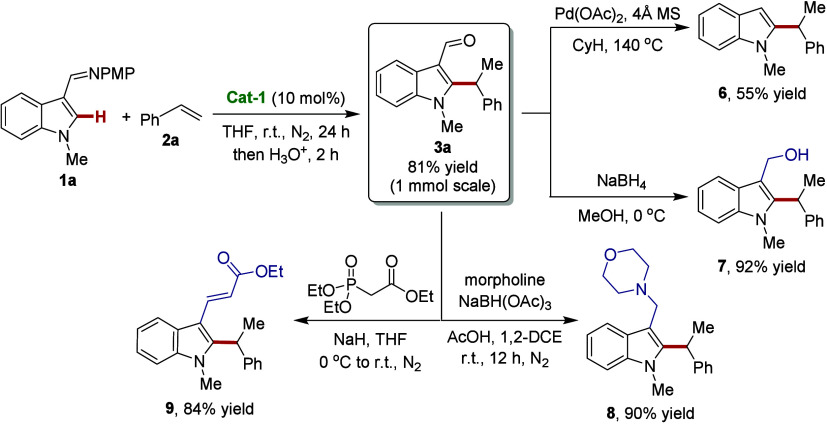
Scale-Up and Diversification of the Indole Product

The robustness of the well-defined iron(0)-NHC catalyst was further reflected by a successful scale-up with indole **1a** to furnish the desired product **3a** in 81% yield, thereby demonstrating the three-coordinate iron(0) complex catalytic system in preparative-scale organic synthesis. The presence of the formyl group on the alkylated indole product provided important opportunities for expanding the molecular diversity. Accordingly, the corresponding indole **6**, alcohol **7**, amine **8**, and olefin **9** were accessed with high yield, as shown in Scheme 4.

### Mechanistic Studies

**Scheme 5:**
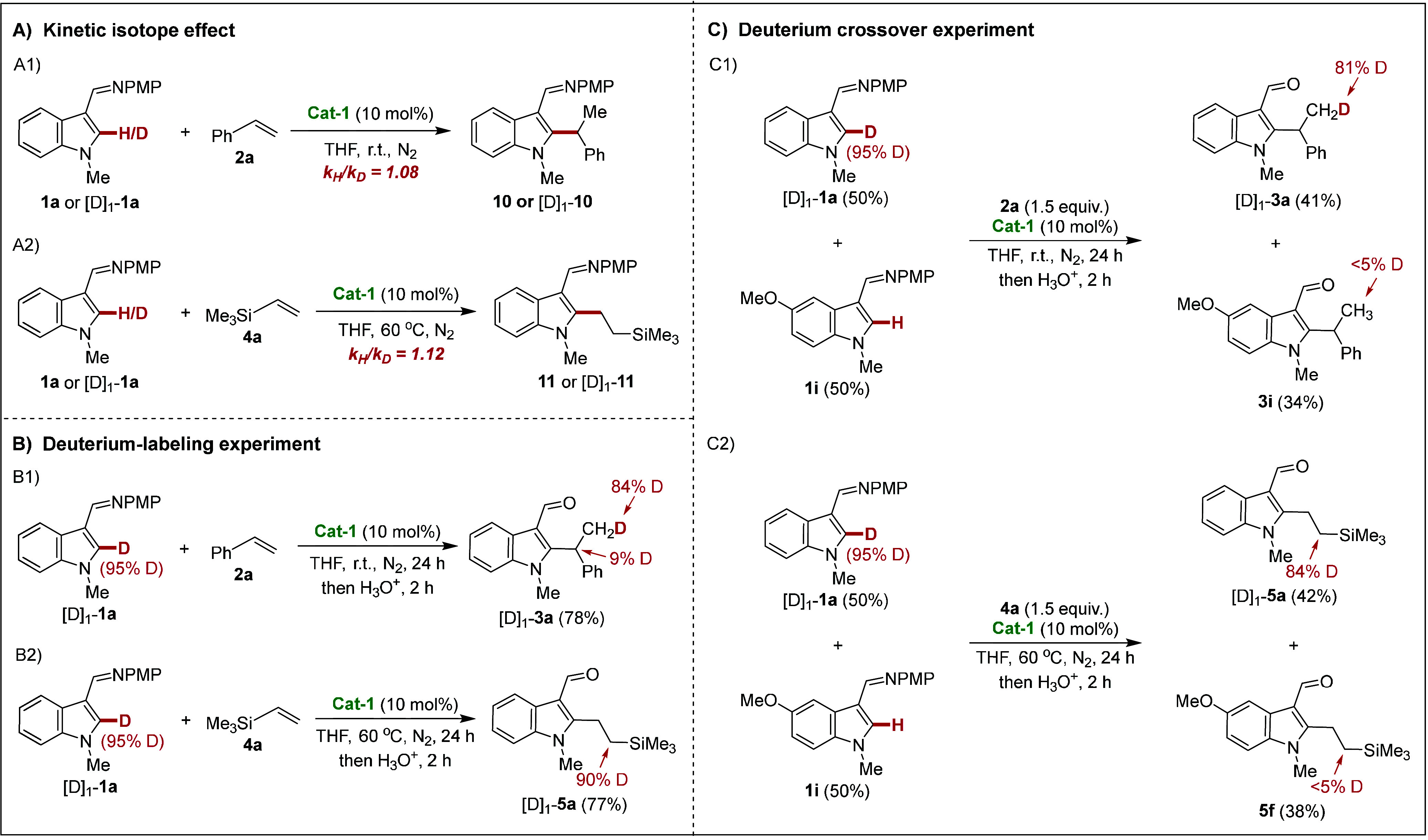
Mechanistic Studies

To gain mechanistic insights into the regioselective C–H alkylation, kinetic isotope effect (KIE) studies, deuterium-labeling experiments, and deuterium crossover experiments were carried out (Scheme 5). First, we conducted two parallel intermolecular KIE experiments with styrene (**2a**) and trimethyl(vinyl)silane (**4a**). The KIE ratios (*k*_H_/*k*_D_) of the two experiments were 1.08 and 1.12, respectively, indicating that the C–H cleavage is facile (Scheme 5A). The reaction of the C_2_-deuterated indole substrate [D]_1_-**1a** with styrene (**2a**) afforded the product [D]_1_-**3a**. The transfer of the C_2_-deuterium from the indole to the methyl position (84% D) and methine position (9% D) indicated that the reversibility of C–H activation is possible (Scheme 5B). The reaction of the C_2_-deuterated indole substrate [D]_1_-**1a** with trimethyl(vinyl)silane (**4a**) resulted in significant transfer from the C_2_-deuterium of the indole to a single methylene position of [D]_1_-**5a** (90% D). In addition, deuterium crossover experiments between two different substrates ([D]_1_-**1a** and **1i**) suggested that there was no deuterium scrambling distribution (Scheme 5C).

**Figure 2:**
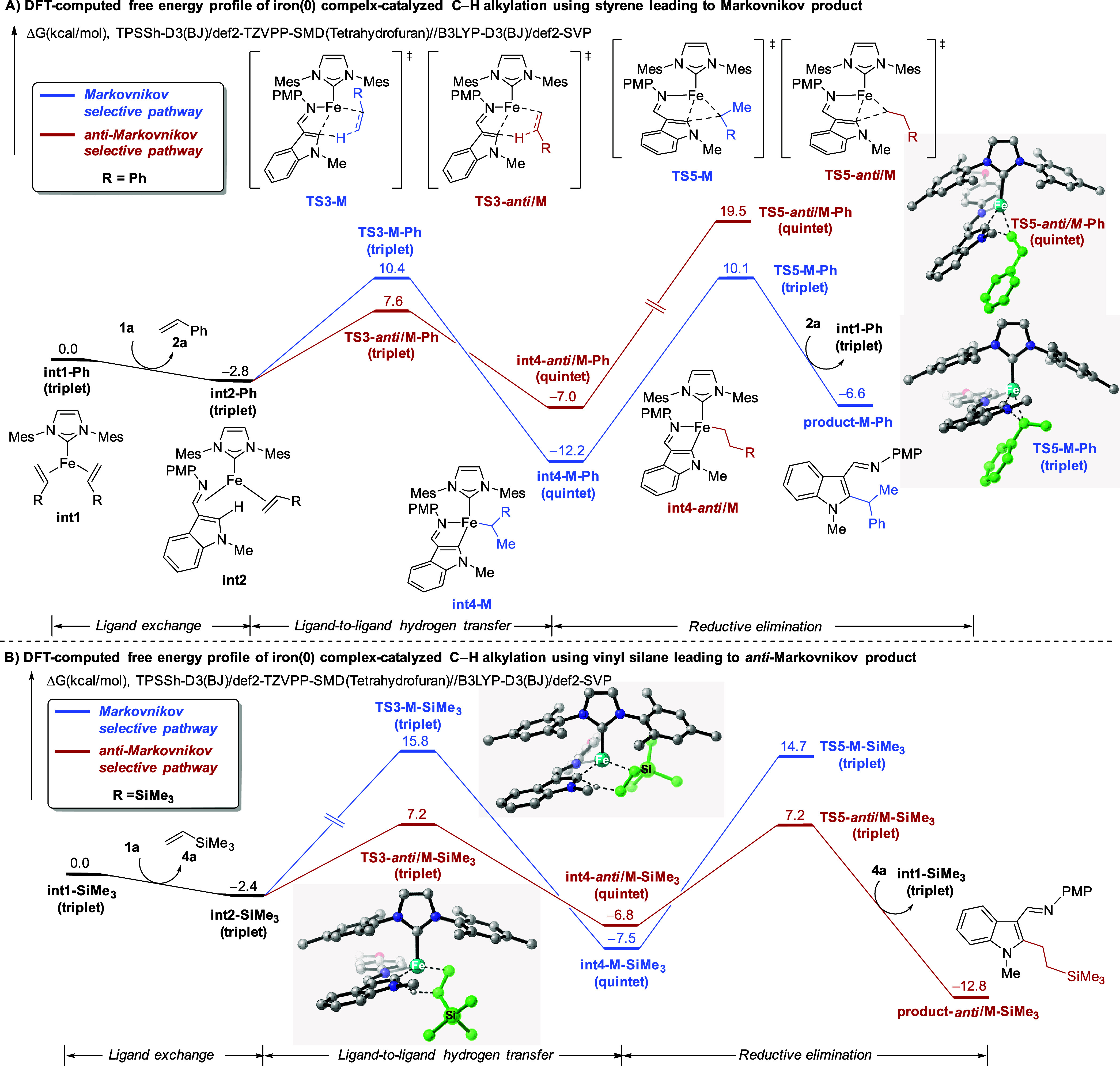
Computational studies on the three-coordinate iron(0) complex-catalyzed C–H alkylation of **1a**. A) Styrene as substrate leading to the Markovnikov product. B) Vinyl silane as substrate leading to the *anti*-Markovnikov product. Nonrelevant hydrogen atoms are absent for clarity in 3D structures.

Given that regioselectivity highly depends on the nature of alkenes, where aryl alkenes yield Markovnikov products and vinyl silanes lead to *anti*-Markovnikov products, density functional theory (DFT) calculations were performed to elaborate the origins of this selectivity.^63^ Two representative alkenes, namely, styrene **2a** and vinylsilane **4a**, were given the corresponding free energy profiles depicted in Figure 2A and Figure 2B, respectively. It is worth mentioning that only the most stable spin state of each species is presented in Figure 2, while all possible spin states are compiled in the Supporting Information as Figures S3 and S4. Initiating from triplet iron(0) complex **int1-Ph**, ligand exchange generates a more stable substrate **1a** coordinated intermediate **int2-Ph**. This species then undergoes ligand-to-ligand hydrogen transfer (LLHT) in a Markovnikov-selective manner *via* a five-membered transition state **TS3-M-Ph**, thus directly forming iron(II) alkyl species **int4-M-Ph**. The subsequent reductive elimination occurs through **TS5-M-Ph**, identified as the rate- and regioselectivity-determining steps for styrenes, thereby giving a branched product. The competing reductive elimination transition state **TS5-***anti***/M-Ph** is disfavored by 9.4 kcal/mol as compared to **TS5-M-Ph**, probably due to the linear alkyl group having an increased distance between the indole motif and the arene of the styrene **2a**, leading to weaker π–π interactions. Moreover, reductive elimination is also the rate-determining step for vinyl silane substrate **4a** which is in good agreement with the KIE findings (*vide supra*). Because of the irreversibility of the LLHT steps for both pathways, we conclude that LLHT is the regioselectivity-determining step for vinyl silane. In the Markovnikov transition state **TS3-M-SiMe**_**3**_, repulsive steric hindrance arises between trimethylsilyl and PMP groups. In contrast, this steric repulsion is absent in the favorable *anti*-Markovnikov transition state **TS3-***anti***/M-SiMe**_**3**_, where the bulky trimethylsilyl is located away from the indole substrate, hence favoring the linear product. Our computational studies provide a general rationale for the alkene-dependent regioselectivity of iron(0) complex catalysis.

## Conclusions

In summary, we have developed a sustainable three-coordinate iron(0) complex-catalyzed regioselective C–H alkylation that proceeds under mild conditions without Grignard reagent and additional additives, providing a series of alkylated indoles with high levels of yield. The regioselectivity of the C–H alkylation was affected by the nature of the alkene substrate, resulting in a variety of alkylated indoles with Markovnikov or *anti*-Markovnikov selectivity. Moreover, mechanistic studies revealed possible reaction pathways, setting the stage for the further application of sustainable 3d metal-catalyzed C–H functionalization.

## Supporting Information Available

Experimental procedures and spectroscopic data for all reactions and characterization data for all products (PDF)
