# Supplementary material for: Three-Coordinate Iron(0) Complex-Catalyzed Regioselective C–H Alkylation of Indole Derivatives
Source: J Am Chem Soc. 2025 Feb 17;147(8):6897–904. doi: 10.1021/jacs.4c17316 (PMC11869306; doi:10.1021/jacs.4c17316)
Supplement: Supplementary file 1 — ja4c17316_si_001.pdf [file ja4c17316_si_001.pdf]

Supporting Information

Three-Coordinate Iron(0) Complex-Catalyzed Regioselective C–H Alkylation of Indole Derivatives

Zi-Jing Zhang, Stéphane Golling, Silvia Cattani, Xinran Chen and Lutz Ackermann*

**Email: Lutz.Ackermann@chemie.unigoettingen.de.*

Table of Contents

1. General information......2
2. Synthesis of three-coordinate iron(0) complexes......3
3. General procedure for regioselective C–H alkylation......12
4. Characterization data of products......14
5. Synthetic transformations......33
6. Mechanistic investigations......37
7. Computational studies......45
8. NMR spectra......100
9. References......145

1. General information

General data:

NMR spectra were recorded on Bruker-300 MHz spectrometer or Bruker-400 MHz spectrometer. Chemical shifts (δ) are given in ppm relative to TMS. The residual solvent signals were used as references and the chemical shifts converted to the TMS scale ($CDCl_3$: δH = 7.26 ppm, δC = 77.16 ppm).

High resolution mass spectra were recorded on a Thermo LTQ Orbitrap XL (ESI+) or a P-SIMS-Gly of Bruker Daltonics Inc (EI+).

Melting point ranges are measured with a Stuart Melting Point Apparatus SMP3 (Barloworld Scientific, Ltd.).

Materials:

All starting materials, reagents and solvents were purchased from commercial suppliers (Aldrich, Alfa, TCI, etc.) and used as supplied unless otherwise stated. Indole substrates[1,2] were synthesized in accordance with the similar procedures in literature. Tetrahydrofuran was dried over Na and distilled prior to use.

2. Synthesis of three-coordinate iron(0) complexes

General Procedure 1: synthesis of three-coordinate iron(0) complexes[3-6]

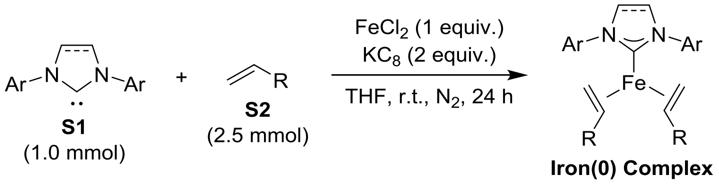

To a colorless solution of NHC ligand **S1** (1.0 mmol) in THF (10 mL) was added $FeCl_2$ (1.0 mmol, 126.7 mg) at room temperature in the glove box. The reaction mixture was stirring at room temperature for 6 hours, then alkene **S2** (2.5 mmol) and KC_8 (2.0 mmol, 270.3 mg) were added subsequently. After stirring at room temperature for 24 hours, the mixture was filtered to give a dark green solution. The solution was subjected to vacuum to remove the volatiles, and the residue was washed with *n*-hexane (10 mL) and dried under vacuum to afford the desired iron(0) complex.

Fe(IMes)(η^2-styrene)$_2$ (**Cat-1**)

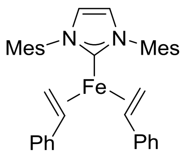

The **general procedure 1** was followed using IMes (1.0 mmol, 304.4 mg) and styrene (2.5 mmol, 287 μL) to afford **Cat-1** (273.1 mg, 48% yield) as a dark green solid. The analytical data are in accordance with these reported in literature.[3]

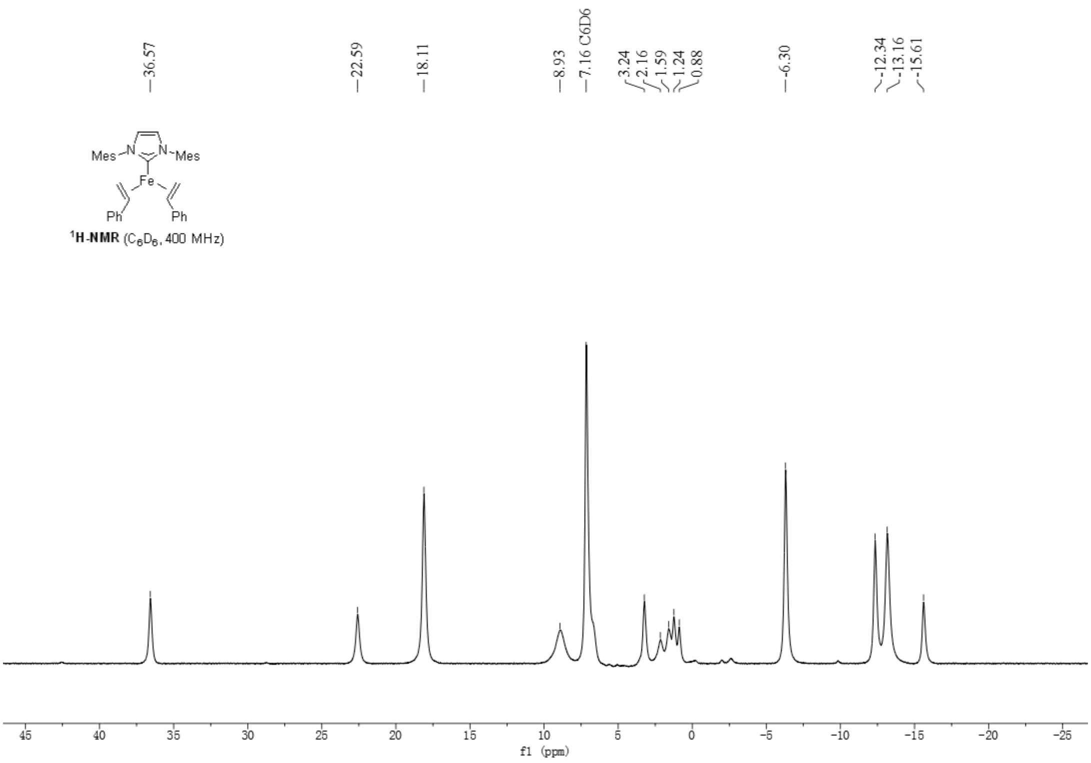

Fe(IPr)(η^2-styrene)$_2$ (**Cat-2**)

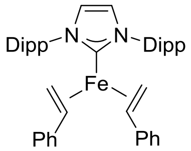

The **general procedure 1** was followed using IPr (1.0 mmol, 388.6 mg) and styrene (2.5 mmol, 287 μL) to afford **Cat-2** (280.9 mg, 43% yield) as a yellow green solid. The analytical data are in accordance with these reported in literature.[3]

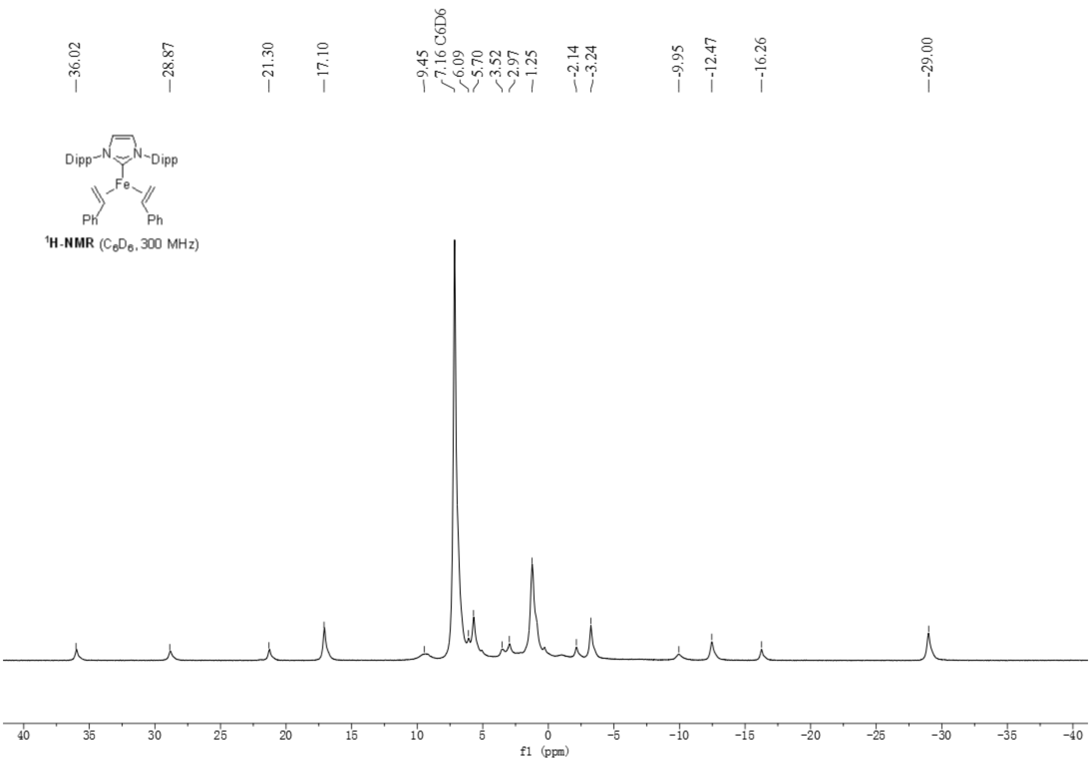

Fe(IXyl)(η^2-styrene)$_2$ (**Cat-3**)

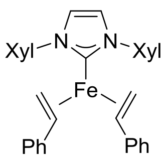

The **general procedure 1** was followed using IXyl (1.0 mmol, 276.4 mg) and styrene (2.5 mmol, 287 μL) to afford **Cat-3** (298.7 mg, 55% yield) as a dark green solid. The analytical data are in accordance with these reported in literature.[3]

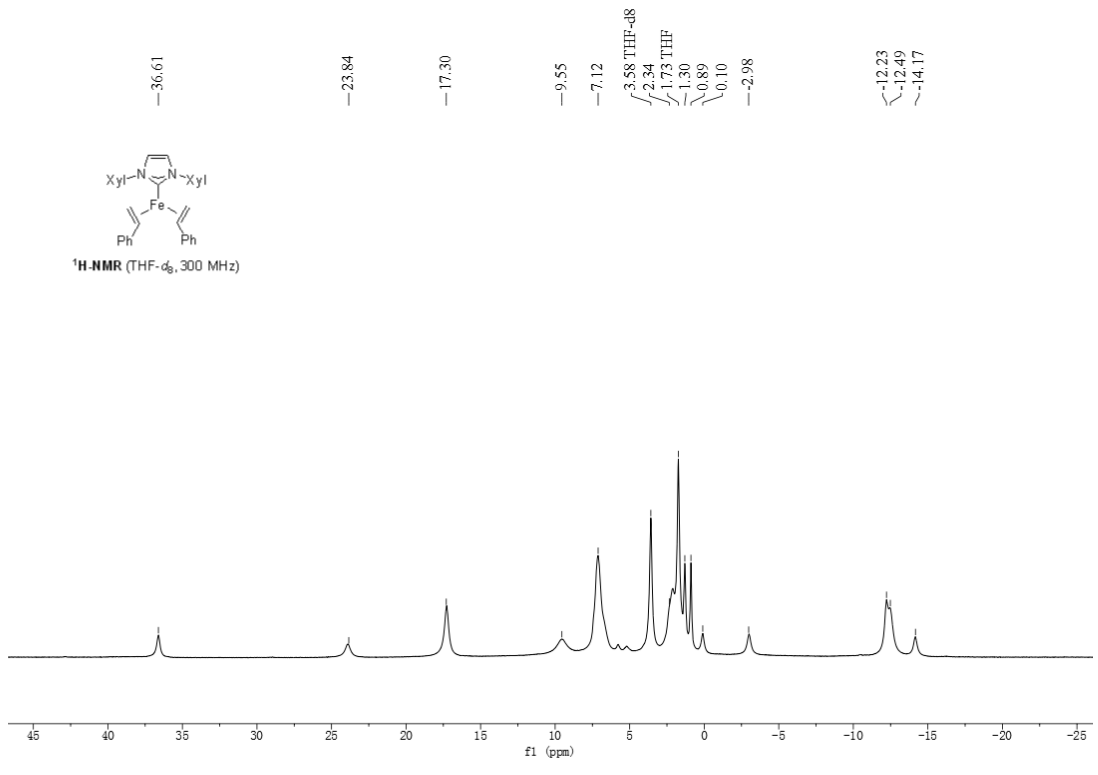

Fe(IMes^{Me})(η^2-styrene)$_2$ (**Cat-4**)

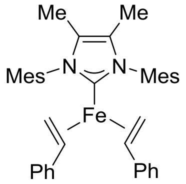

The **general procedure 1** was followed using IMes^{Me} (1.0 mmol, 332.5 mg) and styrene (2.5 mmol, 287 μL) to afford **Cat-4** (341.2 mg, 57% yield) as a brown solid. The analytical data are in accordance with these reported in literature.[4]

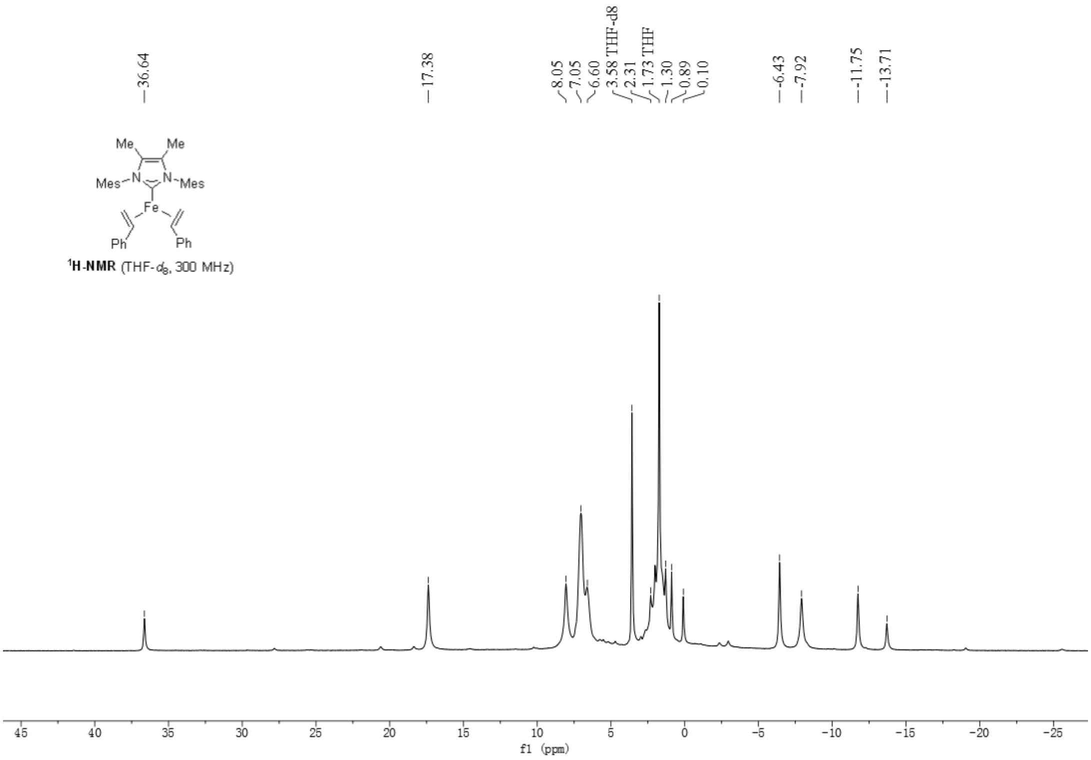

Fe(SIMes)(η^2-styrene)$_2$ (**Cat-5**)

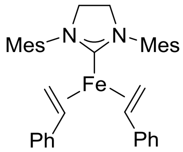

The **general procedure 1** was followed using SIMes (1.0 mmol, 306.5 mg) and styrene (2.5 mmol, 287 μL) to afford **Cat-5** (233.1 mg, 41% yield) as a dark green solid. The analytical data are in accordance with these reported in literature.[4]

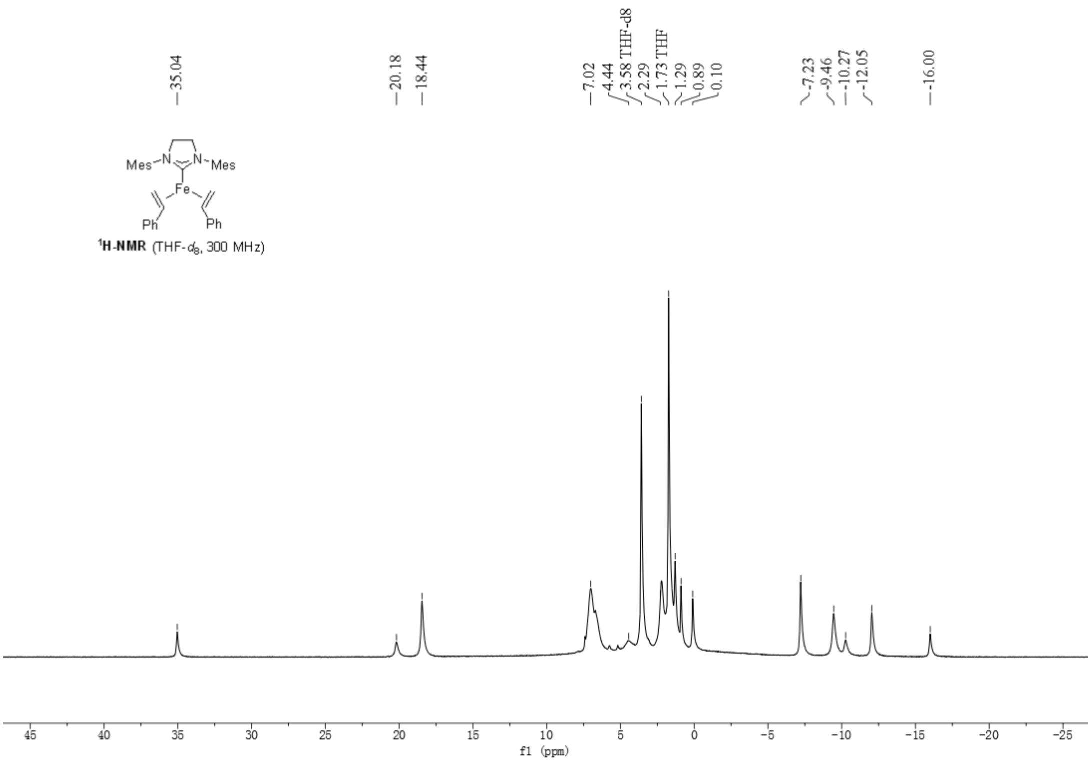

Fe(SIXyl)(η^2-styrene)$_2$ (**Cat-6**)

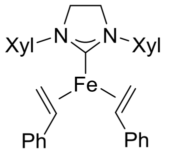

The **general procedure 1** was followed using SIXyl (1.0 mmol, 278.4 mg) and styrene (2.5 mmol, 287 μL) to afford **Cat-6** (240.3 mg, 44% yield) as a dark green solid. The analytical data are in accordance with these reported in literature.[4]

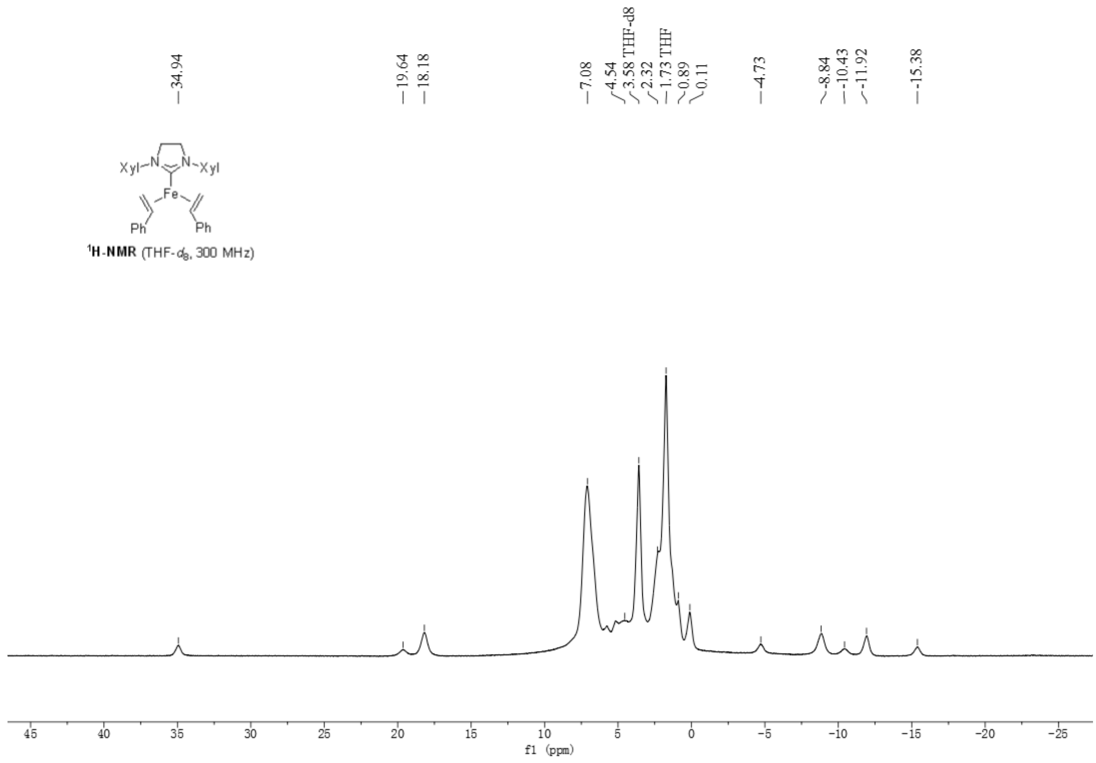

Fe(IMes)(dvtms) (**Cat-7**)

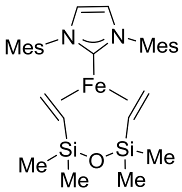

The **general procedure 1** was followed using IMes (1.0 mmol, 304.4 mg) and 1,1,3,3-tetramethyl-1,3-divinyldisiloxane (1.0 mmol, 230 μL) to afford **Cat-7** (431.6 mg, 79% yield) as a green powder. The analytical data are in accordance with these reported in literature.[5]

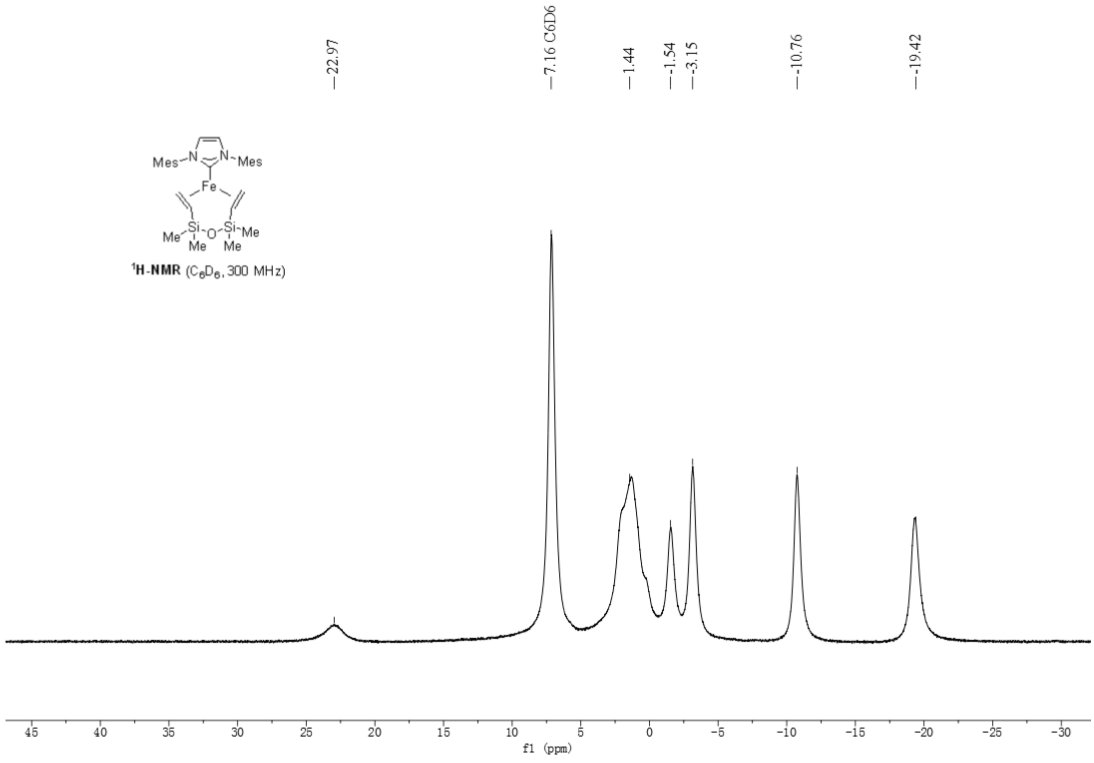

Fe(IPr)(vtms)$_2$ (**Cat-8**)

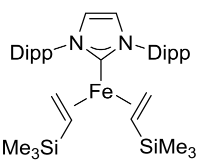

The **general procedure 1** was followed using IPr (1.0 mmol, 388.6 mg) and trimethyl(vinyl)silane (2.5 mmol, 366 μL) to afford **Cat-8** (320.7 mg, 50% yield) as a green powder. The analytical data are in accordance with these reported in literature.[6]

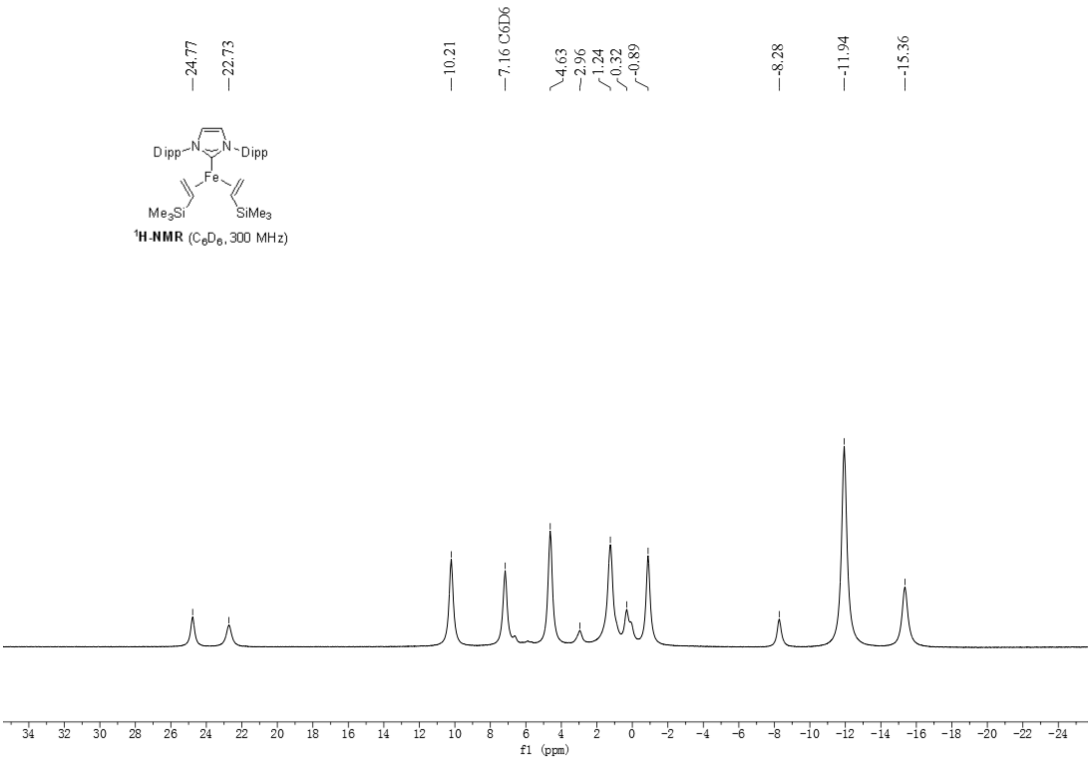

3. General procedure for regioselective C–H alkylation

General Procedure 2: three-coordinate iron(0) complex-catalyzed regioselective C–H alkylation of indole derivatives with aryl alkenes

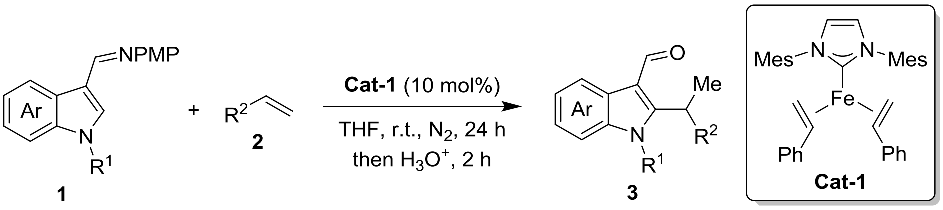

To a flame-dried and N_2-purged Schlenk tube were added indole substrate **1** (0.1 mmol) and **Cat-1** (10 mol%, 0.01 mmol, 5.7 mg) in the glove box. Alkene substrate **2** (0.15 mmol) and tetrahydrofuran (0.3 mL) were added *via* syringe under nitrogen atmosphere, the resulting mixture was stirred at room temperature for 24 hours. Then, the reaction mixture was diluted with tetrahydrofuran (2.0 mL) and quenched with HCl aqueous solution (1 M, 1.0 mL). The resulting mixture was stirred at room temperature for 2 hours. The phases were then separated, the aqueous layer was extracted with ethyl acetate (5.0 mL ×3). The combined organic layer was washed with brine, dried over Na_2SO_4, filtered and concentrated *in vacuo*. The linear and branch ratio was determined by ^{1}H-NMR analysis of the crude reaction mixture. The residue was purified by column chromatography on silica gel (*n*-hexane: ethyl acetate = 5:1) to afford the desired product **3**.

General Procedure 3: three-coordinate iron(0) complex-catalyzed regioselective C–H alkylation of indole derivatives with vinyl silanes

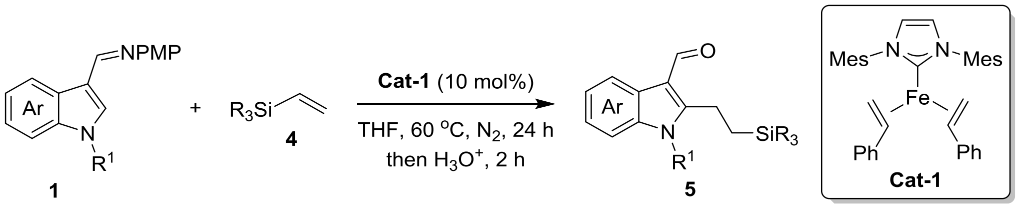

To a flame-dried and N_2-purged Schlenk tube were added indole substrate **1** (0.1 mmol) and **Cat-1** (10 mol%, 0.01 mmol, 5.7 mg) in the glove box. Vinyl silane **4** (0.15 mmol) and tetrahydrofuran (0.3 mL) were added *via* syringe under nitrogen atmosphere, the resulting mixture was stirred at 60 °C for 24 hours. Then, the reaction

mixture was diluted with tetrahydrofuran (2.0 mL) and quenched with HCl aqueous solution (1 M, 1.0 mL). The resulting mixture was stirred at room temperature for 2 hours. The phases were then separated, the aqueous layer was extracted with ethyl acetate (5.0 mL ×3). The combined organic layer was washed with brine, dried over Na_2SO_4, filtered and concentrated *in vacuo*. The linear and branch ratio was determined by ^{1}H-NMR analysis of the crude reaction mixture. The residue was purified by column chromatography on silica gel (*n*-hexane: ethyl acetate = 10:1) to afford the desired product **5**.

4. Characterization data of products

1-Methyl-2-(1-phenylethyl)-1*H*-indole-3-carbaldehyde (3a)

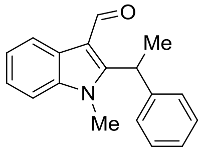

The **general procedure 1** was followed using indole substrate **1a** (0.1 mmol, 26.4 mg) and styrene (**2a**) (0.15 mmol, 17 μL) to afford **3a** (21.1 mg, 80% yield) as a pale-yellow solid (81% yield on 1 mmol scale). **M.p.**: 94-96 °C. **^{1}H-NMR (300 MHz, $CDCl_3$)** δ 10.26 (s, 1H), 8.47 – 8.26 (m, 1H), 7.39 – 7.15 (m, 8H), 5.21 (q, *J* = 7.4 Hz, 1H), 3.44 (s, 3H), 1.87 (d, *J* = 7.4 Hz, 3H). **^{13}C-NMR (75 MHz, $CDCl_3$)** δ 184.9 (CH), 153.6 (C_q), 141.1 (C_q), 137.5 (C_q), 129.0 (CH), 127.1 (CH), 127.0 (CH), 125.9 (C_q), 123.5 (CH), 123.1 (CH), 121.5 (CH), 114.7 (C_q), 109.4 (CH), 34.6 (CH_3), 31.3 (CH), 18.7 (CH_3). **HRMS** (ESI) m/z $(M+H)^+$: calculated for $(C_{18}H_{18}NO)^+$: 264.1383, found: 264.1383. The analytical data are in accordance with these reported in literature.[1]

1-Benzyl-2-(1-phenylethyl)-1*H*-indole-3-carbaldehyde (3b)

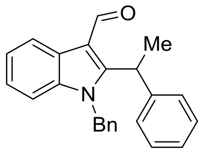

The **general procedure 1** was followed using indole substrate **1b** (0.1 mmol, 34.0 mg) and styrene (**2a**) (0.15 mmol, 17 μL) to afford **3b** (24.7 mg, 73% yield) as a pale-yellow solid. **M.p.**: 145-147 °C. **^{1}H-NMR (300 MHz, $CDCl_3$)** δ 10.23 (s, 1H), 8.42 (d, *J* = 7.9 Hz, 1H), 7.35 – 7.26 (m, 4H), 7.25 – 7.18 (m, 6H), 7.14 (dt, *J* = 8.3, 1.0 Hz, 1H), 6.96 – 6.81 (m, 2H), 5.22 (s, 2H), 5.05 (q, *J* = 7.4 Hz, 1H), 1.74 (d, *J* = 7.4 Hz, 3H). **^{13}C-NMR (75 MHz, $CDCl_3$)** δ 185.6 (CH), 154.0 (C_q), 141.4 (C_q), 137.2 (C_q), 136.1 (C_q), 129.0 (CH), 129.0 (CH), 127.8 (CH), 127.2 (CH), 127.1 (CH), 126.2 (C_q), 125.9 (CH), 123.9 (CH), 123.4 (CH), 121.9 (CH), 115.0 (C_q), 110.4 (CH), 47.7 (CH_2), 35.2 (CH), 20.0 (CH_3). **HRMS** (ESI) m/z $(M+H)^+$: calculated for $(C_{24}H_{22}NO)^+$: 340.1696, found: 340.1709.

1-Phenyl-2-(1-phenylethyl)-1*H*-indole-3-carbaldehyde (3c)

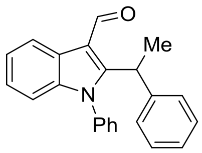

The **general procedure 1** was followed using indole substrate **1c** (0.1 mmol, 32.6 mg) and styrene (**2a**) (0.15 mmol, 17 μL) to afford **3c** (29.4 mg, 90% yield) as a white solid. **M.p.**: 143-145 °C. **^{1}H-NMR (300 MHz, CDCl$_3$)** δ 10.15 (s, 1H), 8.40 (d, *J* = 7.9 Hz, 1H), 7.57 – 7.42 (m, 3H), 7.37 – 7.26 (m, 2H), 7.25 – 7.13 (m, 6H), 7.11 – 7.05 (m, 1H), 6.89 (d, *J* = 8.2 Hz, 1H), 4.63 (q, *J* = 7.3 Hz, 1H), 1.79 (d, *J* = 7.4 Hz, 3H). **^{13}C-NMR (75 MHz, CDCl$_3$)** δ 186.2 (CH), 155.1 (C_q), 142.4 (C_q), 138.6 (C_q), 136.4 (C_q), 130.0 (CH), 130.0 (CH), 129.6 (CH), 128.9 (CH), 128.8 (CH), 128.7 (CH), 127.2 (CH), 126.9 (CH), 125.8 (C_q), 123.8 (CH), 123.5 (CH), 121.9 (CH), 114.7 (C_q), 111.0 (CH), 36.0 (CH), 21.0 (CH_3). **HRMS** (ESI) m/z $(M+H)^+$: calculated for $(C_{23}H_{20}NO)^+$: 326.1539, found: 326.1545.

1-(Methoxymethyl)-2-(1-phenylethyl)-1*H*-indole-3-carbaldehyde (3d)

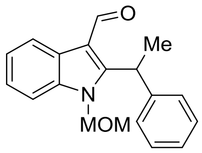

The **general procedure 1** was followed using indole substrate **1d** (0.1 mmol, 29.4 mg) and styrene (**2a**) (0.15 mmol, 17 μL) to afford **3d** (25.5 mg, 87% yield) as a colorless oil. **^{1}H-NMR (300 MHz, CDCl$_3$)** δ 10.22 (s, 1H), 8.43 – 8.33 (m, 1H), 7.50 – 7.39 (m, 1H), 7.39 – 7.26 (m, 6H), 7.28 – 7.20 (m, 1H), 5.32 (d, *J* = 2.7 Hz, 2H), 5.14 (q, *J* = 7.4 Hz, 1H), 3.18 (s, 3H), 1.92 (d, *J* = 7.4 Hz, 3H). **^{13}C-NMR (75 MHz, CDCl$_3$)** δ 186.0 (CH), 154.4 (C_q), 141.7 (C_q), 137.2 (C_q), 129.0 (CH), 127.3 (CH), 127.2 (CH), 126.0 (C_q), 124.0 (CH), 123.5 (CH), 121.9 (CH), 115.6 (C_q), 110.0 (CH), 74.6 (CH_2), 56.2 (CH_3), 35.1 (CH), 20.4 (CH_3). **HRMS** (ESI) m/z $(M+H)^+$: calculated for $(C_{19}H_{20}NO_2)^+$: 294.1489, found: 294.1499.

1-Methyl-2-(1-phenylethyl)-1*H*-pyrrolo[2,3-*b*]pyridine-3-carbaldehyde (3e)

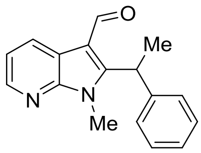

The **general procedure 1** was followed using indole substrate **1e** (0.1 mmol, 26.5 mg) and styrene (**2a**) (0.15 mmol, 17 µL) to afford **3e** (24.4 mg, 92% yield) as a yellow oil. **^{1}H-NMR (300 MHz, CDCl$_3$)** δ 10.22 (s, 1H), 8.60 (dd, *J* = 7.8, 1.6 Hz, 1H), 8.36 (dd, *J* = 4.8, 1.6 Hz, 1H), 7.42 – 7.30 (m, 2H), 7.28 – 7.16 (m, 4H), 5.16 (q, *J* = 7.4 Hz, 1H), 3.61 (s, 3H), 1.92 (d, *J* = 7.4 Hz, 3H). **^{13}C-NMR (75 MHz, CDCl$_3$)** δ 184.9 (CH), 154.3 (C$_q$), 148.6 (C$_q$), 144.4 (CH), 140.7 (C$_q$), 130.0 (CH), 129.1 (CH), 127.3 (CH), 127.2 (CH), 119.2 (CH), 118.6 (C$_q$), 113.1 (C$_q$), 35.2 (CH$_3$), 29.6 (CH), 19.0 (CH$_3$). **HRMS** (ESI) m/z $(M+H)^+$: calculated for $(C_{17}H_{17}N_2O)^+$: 265.1335, found: 265.1335.

5-Fluoro-1-methyl-2-(1-phenylethyl)-1*H*-indole-3-carbaldehyde (3f)

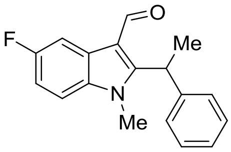

The **general procedure 1** was followed using indole substrate **1f** (0.1 mmol, 28.2 mg) and styrene (**2a**) (0.15 mmol, 17 µL) to afford **3f** (21.7 mg, 77% yield) as a white solid. **M.p.**: 117-119 °C. **^{1}H-NMR (300 MHz, CDCl$_3$)** δ 10.21 (s, 1H), 8.06 (dd, *J* = 9.4, 2.6 Hz, 1H), 7.38 – 7.23 (m, 5H), 7.19 (dd, *J* = 8.9, 4.2 Hz, 1H), 7.03 (td, *J* = 8.9, 2.6 Hz, 1H), 5.16 (q, *J* = 7.4 Hz, 1H), 3.46 (s, 3H), 1.89 (d, *J* = 7.4 Hz, 3H). **^{13}C-NMR (75 MHz, CDCl$_3$)** δ 184.7 (CH), 160.2 (d, *J* = 238.5 Hz, C$_q$), 154.7 (C$_q$), 140.9 (C$_q$), 134.0 (C$_q$), 129.1 (CH), 127.2 (CH), 127.1 (CH), 126.5 (d, *J* = 11.0 Hz, C$_q$), 114.8 (d, *J* = 4.3 Hz, C$_q$), 111.7 (d, *J* = 26.2 Hz, CH), 110.2 (d, *J* = 9.7 Hz, CH), 107.3 (d, *J* = 24.9 Hz, CH), 34.8 (CH$_3$), 31.5 (CH), 18.8 (CH$_3$). **^{19}F-NMR (282 MHz, CDCl$_3$)** δ -120.4. **HRMS** (ESI) m/z $(M+H)^+$: calculated for $(C_{18}H_{17}FNO)^+$: 282.1289, found: 282.1299.

3-Formyl-1-methyl-2-(1-phenylethyl)-1*H*-indole-5-carbonitrile (3g)

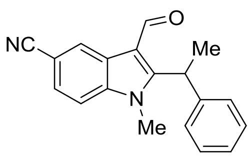

The **general procedure 1** was followed using indole substrate **1g** (0.1 mmol, 28.9 mg) and styrene (**2a**) (0.15 mmol, 17 µL) to afford **3g** (15.5 mg, 54% yield) as a pale-yellow solid. **M.p.**: 130-132 °C. **^{1}H-NMR (300 MHz, CDCl$_3$)** δ 10.25 (s, 1H), 8.74 (dd, *J* = 1.7, 0.7 Hz, 1H), 7.53 (dd, *J* = 8.5, 1.6 Hz, 1H), 7.39 – 7.31 (m, 3H), 7.31 – 7.27 (m, 1H), 7.24 (d, *J* = 6.8 Hz, 2H), 5.17 (q, *J* = 7.4 Hz, 1H), 3.51 (s, 3H), 1.91 (d, *J* = 7.4 Hz, 3H). **^{13}C-NMR (75 MHz, CDCl$_3$)** δ 184.8 (CH), 155.6 (C_q), 140.4 (C_q), 139.1 (C_q), 129.2 (CH), 127.4 (CH), 127.1 (CH), 127.0 (CH), 126.8 (CH), 125.7 (C_q), 120.1 (C_q), 114.9 (C_q), 110.4 (CH), 106.4 (C_q), 34.9 (CH_3), 31.6 (CH), 18.9 (CH_3). **HRMS** (ESI) m/z $(M+H)^+$: calculated for $(C_{19}H_{17}N_2O)^+$: 289.1335, found: 289.1340.

1,5-Dimethyl-2-(1-phenylethyl)-1*H*-indole-3-carbaldehyde (3h)

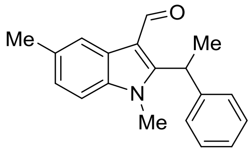

The **general procedure 1** was followed using indole substrate **1h** (0.1 mmol, 27.8 mg) and styrene (**2a**) (0.15 mmol, 17 µL) to afford **3h** (21.4 mg, 77% yield) as a colorless oil. **^{1}H-NMR (300 MHz, CDCl$_3$)** δ 10.24 (s, 1H), 8.21 (s, 1H), 7.37 – 7.21 (m, 5H), 7.18 – 7.09 (m, 2H), 5.19 (q, *J* = 7.4 Hz, 1H), 3.43 (s, 3H), 2.50 (s, 3H), 1.88 (d, *J* = 7.4 Hz, 3H). **^{13}C-NMR (75 MHz, CDCl$_3$)** δ 184.9 (CH), 153.6 (C_q), 141.2 (C_q), 135.9 (C_q), 132.9 (C_q), 129.0 (CH), 127.1 (CH), 127.0 (CH), 126.1 (C_q), 125.0 (CH), 121.4 (CH), 114.4 (C_q), 109.1 (CH), 34.6 (CH_3), 31.3 (CH), 21.6 (CH_3), 18.7 (CH_3). **HRMS** (ESI) m/z $(M+H)^+$: calculated for $(C_{19}H_{20}NO)^+$: 278.1539, found: 278.1535.

5-Methoxy-1-methyl-2-(1-phenylethyl)-1*H*-indole-3-carbaldehyde (3i)

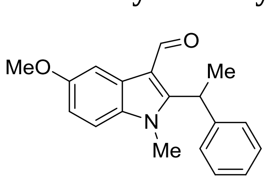

The **general procedure 1** was followed using indole substrate **1i** (0.1 mmol, 29.4 mg) and styrene (**2a**) (0.15 mmol, 17 μL) to afford **3i** (16.1 mg, 55% yield) as a yellow oil. **^{1}H-NMR (300 MHz, CDCl$_3$)** δ 10.21 (s, 1H), 7.91 (d, *J* = 2.6 Hz, 1H), 7.37 – 7.31 (m, 2H), 7.29 – 7.24 (m, 2H), 7.26 – 7.22 (m, 1H), 7.16 (d, *J* = 8.9 Hz, 1H), 6.93 (dd, *J* = 8.9, 2.5 Hz, 1H), 5.13 (q, *J* = 7.4 Hz, 1H), 3.91 (s, 3H), 3.44 (s, 3H), 1.88 (d, *J* = 7.3 Hz, 3H). **^{13}C-NMR (75 MHz, CDCl$_3$)** δ 184.8 (CH), 157.0 (C$_q$), 153.7 (C$_q$), 141.2 (C$_q$), 132.3 (C$_q$), 129.0 (CH), 127.1 (CH), 127.0 (CH), 126.5 (C$_q$), 114.7 (C$_q$), 113.8 (CH), 110.2 (CH), 103.3 (CH), 56.1 (CH$_3$), 34.7 (CH$_3$), 31.3 (CH), 18.9 (CH$_3$). **HRMS** (ESI) m/z (M+H)$^+$: calculated for ($C_{19}H_{20}NO_2$)$^+$: 294.1489, found: 294.1488.

6-Chloro-1-methyl-2-(1-phenylethyl)-1*H*-indole-3-carbaldehyde (3j)

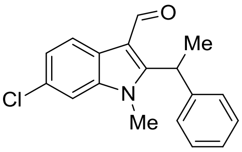

The **general procedure 1** was followed using indole substrate **1j** (0.1 mmol, 29.8 mg) and styrene (**2a**) (0.15 mmol, 17 μL) to afford **3j** (21.2 mg, 71% yield) as a colorless oil. **^{1}H-NMR (300 MHz, CDCl$_3$)** δ 10.23 (s, 1H), 8.29 (d, *J* = 8.9 Hz, 1H), 7.39 – 7.22 (m, 7H), 5.17 (q, *J* = 7.4 Hz, 1H), 3.42 (s, 3H), 1.89 (d, *J* = 7.4 Hz, 3H). **^{13}C-NMR (75 MHz, CDCl$_3$)** δ 184.8 (CH), 154.2 (C$_q$), 140.8 (C$_q$), 138.1 (C$_q$), 129.6 (C$_q$), 129.1 (CH), 127.2 (CH), 127.1 (CH), 124.3 (C$_q$), 123.7 (CH), 122.6 (CH), 114.7 (C$_q$), 109.7 (CH), 34.7 (CH$_3$), 31.4 (CH), 18.8 (CH$_3$). **HRMS** (ESI) m/z (M+H)$^+$: calculated for ($C_{18}H_{17}ClNO$)$^+$: 298.0993, found: 298.0996.

1,6-Dimethyl-2-(1-phenylethyl)-1*H*-indole-3-carbaldehyde (3k)

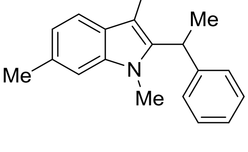

The **general procedure 1** was followed using indole substrate **1k** (0.1 mmol, 27.8 mg) and styrene (**2a**) (0.15 mmol, 17 μL) to afford **3k** (15.8 mg, 57% yield) as a colorless oil. **^{1}H-NMR (300 MHz, CDCl$_3$)** δ 10.24 (s, 1H), 8.25 (d, *J* = 8.1 Hz, 1H), 7.39 – 7.27

(m, 2H), 7.30 – 7.19 (m, 3H), 7.15 (ddd, J = 8.0, 1.4, 0.7 Hz, 1H), 7.07 (s, 1H), 5.20 (q, J = 7.4 Hz, 1H), 3.42 (s, 3H), 2.49 (s, 3H), 1.88 (d, J = 7.4 Hz, 3H). **^{13}C-NMR (75 MHz, $CDCl_3$)** δ 184.8 (CH), 153.2 (C_q), 141.3 (C_q), 137.9 (C_q), 133.6 (C_q), 129.0 (CH), 127.1 (CH), 127.0 (CH), 124.7 (CH), 123.6 (C_q), 121.2 (CH), 114.7 (C_q), 109.5 (CH), 34.5 (CH_3), 31.2 (CH), 22.0 (CH_3), 18.8 (CH_3). **HRMS** (ESI) m/z $(M+H)^+$: calculated for $(C_{19}H_{20}NO)^+$: 278.1539, found: 278.1549.

7-Fluoro-1-methyl-2-(1-phenylethyl)-1*H*-indole-3-carbaldehyde (3l)

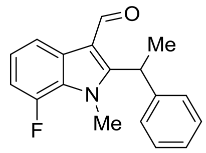

The **general procedure 1** was followed using indole substrate **1l** (0.1 mmol, 28.2 mg) and styrene (**2a**) (0.15 mmol, 17 μL) to afford **3l** (16.1 mg, 57% yield) as a yellow oil. **^{1}H-NMR (300 MHz, $CDCl_3$)** δ 10.24 (s, 1H), 8.14 (dd, J = 7.9, 1.0 Hz, 1H), 7.39 – 7.30 (m, 2H), 7.30 – 7.24 (m, 3H), 7.23 – 7.13 (m, 1H), 6.96 (ddd, J = 12.9, 8.0, 0.9 Hz, 1H), 5.19 (q, J = 7.4 Hz, 1H), 3.70 (d, J = 1.5 Hz, 3H), 1.89 (d, J = 7.4 Hz, 3H). **^{13}C-NMR (75 MHz, $CDCl_3$)** δ 185.0 (CH), 154.3 (C_q), 150.0 (d, J = 245.0 Hz, C_q), 140.9 (C_q), 129.5 (d, J = 4.0 Hz, C_q), 129.1 (CH), 127.1 (CH), 127.0 (CH), 125.2 (d, J = 8.1 Hz, C_q), 123.5 (d, J = 6.6 Hz, CH), 117.3 (d, J = 3.9 Hz, CH), 115.2 (C_q), 109.7 (d, J = 18.5 Hz, CH), 34.4 (CH), 34.1 (d, J = 8.0 Hz, CH_3), 18.6 (CH_3). **^{19}F-NMR (282 MHz, $CDCl_3$)** δ -134.8. **HRMS** (ESI) m/z $(M+H)^+$: calculated for $(C_{18}H_{17}FNO)^+$: 282.1289, found: 282.1291.

7-Ethyl-1-methyl-2-(1-phenylethyl)-1*H*-indole-3-carbaldehyde (3m)

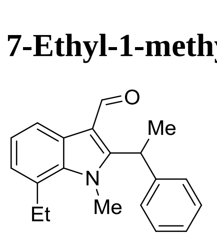

The **general procedure 1** was followed at 60 °C using indole substrate **1m** (0.1 mmol, 29.2 mg) and styrene (**2a**) (0.15 mmol, 17 μL) to afford **3m** (12.9 mg, 44% yield) as a yellow oil. **^{1}H-NMR (300 MHz, $CDCl_3$)** δ 10.28 (s, 1H), 8.30 (dd, J = 7.9, 1.3 Hz, 1H),

7.38 – 7.30 (m, 2H), 7.29 – 7.23 (m, 3H), 7.22 (d, J = 7.7 Hz, 1H), 7.08 (dd, J = 7.4, 1.2 Hz, 1H), 5.30 (q, J = 7.4 Hz, 1H), 3.68 (s, 3H), 3.06 (d, J = 7.5 Hz, 2H), 1.88 (d, J = 7.4 Hz, 3H), 1.31 (t, J = 7.5 Hz, 3H). **^{13}C-NMR (75 MHz, $CDCl_3$)** δ 185.0 (CH), 153.9 (C_q), 141.3 (C_q), 135.6 (C_q), 129.0 (CH), 128.0 (C_q), 127.2 (C_q), 127.0 (CH), 127.0 (CH), 125.3 (CH), 123.2 (CH), 119.3 (CH), 114.7 (C_q), 34.5 (CH), 34.2 (CH_3), 26.0 (CH_2), 18.5 (CH_3), 16.9 (CH_3). **HRMS** (ESI) m/z $(M+H)^+$: calculated for $(C_{20}H_{22}NO)^+$: 292.1696, found: 292.1701.

2-(1-(4-Fluorophenyl)ethyl)-1-methyl-1*H*-indole-3-carbaldehyde (3n)

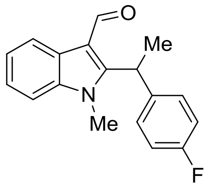

The **general procedure 1** was followed using indole substrate **1a** (0.1 mmol, 26.4 mg) and 1-fluoro-4-vinylbenzene (**2b**) (0.15 mmol, 18 μL) to afford **3n** (21.0 mg, 75% yield) as a colorless oil. **^{1}H-NMR (300 MHz, $CDCl_3$)** δ 10.27 (s, 1H), 8.41 – 8.30 (m, 1H), 7.36 – 7.28 (m, 3H), 7.27 – 7.19 (m, 2H), 7.11 – 6.94 (m, 2H), 5.22 (q, J = 7.3 Hz, 1H), 3.47 (s, 3H), 1.88 (d, J = 7.4 Hz, 3H). **^{13}C-NMR (75 MHz, $CDCl_3$)** δ 184.8 (CH), 161.8 (d, J = 246.2 Hz, C_q), 153.0 (C_q), 137.5 (C_q), 136.9 (d, J = 3.3 Hz, C_q), 128.7 (d, J = 7.9 Hz, CH), 126.0 (C_q), 123.7 (CH), 123.2 (CH), 121.4 (CH), 115.9 (d, J = 21.4 Hz, CH), 114.6 (C_q), 109.5 (CH), 34.0 (CH_3), 31.3 (CH), 18.9 (CH_3). **^{19}F-NMR (282 MHz, $CDCl_3$)** δ -115.7. **HRMS** (ESI) m/z $(M+H)^+$: calculated for $(C_{18}H_{17}FNO)^+$: 282.1289, found: 282.1293.

1-Methyl-2-(1-(4-(trifluoromethyl)phenyl)ethyl)-1*H*-indole-3-carbaldehyde (3o)

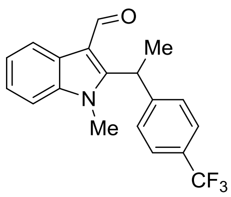

The **general procedure 1** was followed using indole substrate **1a** (0.1 mmol, 26.4 mg) and 1-(trifluoromethyl)-4-vinylbenzene (**2c**) (0.15 mmol, 22 μL) to afford **3o** (11.3 mg,

34% yield) as a colorless oil. **^{1}H-NMR (300 MHz, CDCl$_3$)** δ 10.29 (s, 1H), 8.42 – 8.28 (m, 1H), 7.60 (d, *J* = 8.3 Hz, 2H), 7.39 (d, *J* = 8.2 Hz, 2H), 7.38 – 7.26 (m, 3H), 5.33 (q, *J* = 7.4 Hz, 1H), 3.46 (s, 3H), 1.92 (d, *J* = 7.4 Hz, 3H). **^{13}C-NMR (75 MHz, CDCl$_3$)** δ 184.7 (CH), 151.9 (C$_q$), 145.4 (C$_q$), 137.5 (C$_q$), 129.5 (q, *J* = 32.6 Hz, C$_q$), 127.5 (CH), 126.1 (CH), 126.0 (q, *J* = 3.7 Hz, CH), 124.1 (q, *J* = 272.2 Hz, C$_q$), 123.8 (CH), 123.3 (CH), 121.2 (CH), 114.6 (C$_q$), 109.6 (CH), 34.6 (CH$_3$), 31.4 (CH), 18.6 (CH$_3$). **^{19}F-NMR (282 MHz, CDCl$_3$)** δ -62.5. **HRMS** (ESI) m/z (M+H)$^+$: calculated for $(C_{19}H_{17}F_3NO)^+$: 332.1257, found: 332.1254.

1-Methyl-2-(1-(*p*-tolyl)ethyl)-1*H*-indole-3-carbaldehyde (3p)

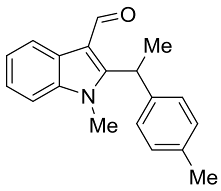

The **general procedure 1** was followed using indole substrate **1a** (0.1 mmol, 26.4 mg) and 1-methyl-4-vinylbenzene (**2d**) (0.15 mmol, 20 μL) to afford **3p** (25.1 mg, 91% yield) as a colorless oil. **^{1}H-NMR (300 MHz, CDCl$_3$)** δ 10.26 (s, 1H), 8.49 – 8.28 (m, 1H), 7.36 – 7.22 (m, 3H), 7.14 (s, 4H), 5.17 (q, *J* = 7.4 Hz, 1H), 3.47 (s, 3H), 2.33 (s, 3H), 1.87 (d, *J* = 7.4 Hz, 3H). **^{13}C-NMR (75 MHz, CDCl$_3$)** δ 185.0 (CH), 153.9 (C$_q$), 138.1 (C$_q$), 137.5 (C$_q$), 136.7 (C$_q$), 129.7 (CH), 127.0 (CH), 125.9 (C$_q$), 123.5 (CH), 123.1 (CH), 121.6 (CH), 114.7 (C$_q$), 109.4 (CH), 34.3 (CH$_3$), 31.3 (CH), 21.1 (CH$_3$), 18.8 (CH$_3$). **HRMS** (ESI) m/z (M+H)$^+$: calculated for $(C_{19}H_{20}NO)^+$: 278.1539, found: 278.1542.

2-(1-(4-(*tert*-Butyl)phenyl)ethyl)-1-methyl-1*H*-indole-3-carbaldehyde (3q)

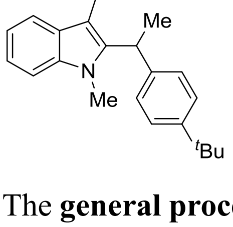

The **general procedure 1** was followed using indole substrate **1a** (0.1 mmol, 26.4 mg) and 1-(*tert*-butyl)-4-vinylbenzene (**2e**) (0.15 mmol, 27 μL) to afford **3q** (19.3 mg, 60%

yield) as a colorless oil. **^{1}H-NMR (300 MHz, $CDCl_3$)** δ 10.26 (s, 1H), 8.48 – 8.31 (m, 1H), 7.38 – 7.25 (m, 5H), 7.19 (d, *J* = 7.7 Hz, 2H), 5.16 (q, *J* = 7.5 Hz, 1H), 3.49 (s, 3H), 1.88 (d, *J* = 7.4 Hz, 3H), 1.30 (s, 9H). **^{13}C-NMR (75 MHz, $CDCl_3$)** δ 185.0 (CH), 154.0 (C_q), 150.0 (C_q), 138.0 (C_q), 137.5 (C_q), 126.8 (CH), 125.9 (C_q), 125.9 (CH), 123.5 (CH), 123.2 (CH), 121.7 (CH), 114.7 (C_q), 109.4 (CH), 34.6 (C_q), 34.2 (CH_3), 31.5 (CH_3), 31.3 (CH), 18.9 (CH_3). **HRMS** (ESI) m/z $(M+H)^+$: calculated for $(C_{22}H_{26}NO)^+$: 320.2009, found: 320.2016.

2-(1-([1,1'-Biphenyl]-4-yl)ethyl)-1-methyl-1*H*-indole-3-carbaldehyde (3r)

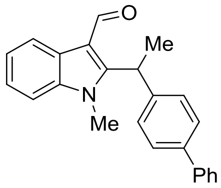

The **general procedure 1** was followed using indole substrate **1a** (0.1 mmol, 26.4 mg) and 4-vinyl-1,1'-biphenyl (**2f**) (0.15 mmol, 27.0 mg) to afford **3r** (29.9 mg, 88% yield) as a colorless oil. **^{1}H-NMR (300 MHz, $CDCl_3$)** δ 10.33 (s, 1H), 8.50 – 8.37 (m, 1H), 7.65 – 7.53 (m, 4H), 7.50 – 7.41 (m, 2H), 7.40 – 7.29 (m, 6H), 5.27 (q, *J* = 7.4 Hz, 1H), 3.52 (s, 3H), 1.93 (d, *J* = 7.4 Hz, 3H). **^{13}C-NMR (75 MHz, $CDCl_3$)** δ 184.8 (CH), 153.4 (C_q), 140.4 (C_q), 140.2 (C_q), 139.8 (C_q), 137.5 (C_q), 128.9 (CH), 127.5 (CH), 127.5 (CH), 127.1 (CH), 125.9 (C_q), 123.6 (CH), 123.1 (CH), 121.5 (CH), 114.6 (C_q), 109.4 (CH), 34.3 (CH_3), 31.3 (CH), 18.8 (CH_3). **HRMS** (ESI) m/z $(M+H)^+$: calculated for $(C_{24}H_{22}NO)^+$: 340.1696, found: 340.1699.

2-(1-(4-(Dimethylamino)phenyl)ethyl)-1-methyl-1*H*-indole-3-carbaldehyde (3s)

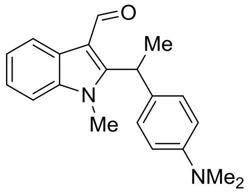

The **general procedure 1** was followed using indole substrate **1a** (0.1 mmol, 26.4 mg) and *N*,*N*-dimethyl-4-vinylaniline (**2g**) (0.15 mmol, 22.1 mg) to afford **3s** (18.2 mg, 59% yield) as a pale-yellow oil. **^{1}H-NMR (300 MHz, $CDCl_3$)** δ 10.26 (s, 1H), 8.45 – 8.30

(m, 1H), 7.36 – 7.23 (m, 3H), 7.11 (dd, J = 9.0, 0.9 Hz, 2H), 6.68 (d, J = 8.8 Hz, 2H), 5.10 (q, J = 7.4 Hz, 1H), 3.49 (s, 3H), 2.93 (s, 6H), 1.84 (d, J = 7.4 Hz, 3H). **^{13}C-NMR (75 MHz, CDCl$_3$)** δ 185.1 (CH), 154.8 (C_q), 149.5 (C_q), 137.6 (C_q), 128.6 (C_q), 127.8 (CH), 125.9 (C_q), 123.4 (CH), 123.1 (CH), 121.8 (CH), 114.7 (C_q), 112.9 (CH), 109.3 (CH), 40.7 (CH_3), 33.7 (CH_3), 31.2 (CH), 19.0 (CH_3). **HRMS** (ESI) m/z $(M+H)^+$: calculated for $(C_{20}H_{23}N_2O)^+$: 307.1805, found: 307.1805.

1-Methyl-2-(1-(4-(4,4,5,5-tetramethyl-1,3,2-dioxaborolan-2-yl)phenyl)ethyl)-1*H*-indole-3-carbaldehyde (3t)

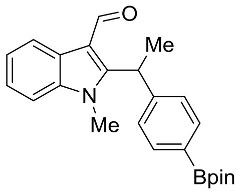

The **general procedure 1** was followed using indole substrate **1a** (0.1 mmol, 26.4 mg) and 4,4,5,5-tetramethyl-2-(4-vinylphenyl)-1,3,2-dioxaborolane (**2h**) (0.15 mmol, 34.5 mg) to afford **3t** (29.6 mg, 76% yield) as a colorless oil. **^{1}H-NMR (300 MHz, CDCl$_3$)** δ 10.27 (s, 1H), 8.47 – 8.30 (m, 1H), 7.77 (d, J = 8.1 Hz, 2H), 7.37 – 7.23 (m, 5H), 5.25 (q, J = 7.3 Hz, 1H), 3.42 (s, 3H), 1.88 (d, J = 7.4 Hz, 3H), 1.34 (s, 12H). **^{13}C-NMR (75 MHz, CDCl$_3$)** δ 184.9 (CH), 153.4 (C_q), 144.3 (C_q), 137.5 (C_q), 135.4 (CH), 129.2 (C_q), 126.5 (CH), 125.9 (C_q), 123.6 (CH), 123.2 (CH), 121.5 (CH), 114.7 (C_q), 109.4 (CH), 84.0 (C_q), 34.8 (CH_3), 31.4 (CH), 25.0 (CH_3), 25.0 (CH_3), 18.6 (CH_3). **HRMS** (ESI) m/z $(M+H)^+$: calculated for $(C_{24}H_{29}BNO_3)^+$: 390.2235, found: 390.2235.

2-(1-(4-Methoxyphenyl)ethyl)-1-methyl-1*H*-indole-3-carbaldehyde (3u)

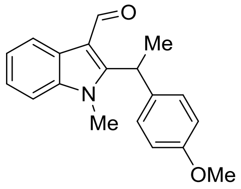

The **general procedure 1** was followed using indole substrate **1a** (0.1 mmol, 26.4 mg) and 1-methoxy-4-vinylbenzene (**2i**) (0.15 mmol, 20 μL) to afford **3u** (24.1 mg, 82%

yield) as a white solid. **M.p.**: 164-166 °C. **^{1}H-NMR (300 MHz, CDCl$_3$)** δ 10.26 (s, 1H), 8.47 – 8.30 (m, 1H), 7.38 – 7.25 (m, 3H), 7.17 (dd, J = 8.9, 1.0 Hz, 2H), 6.86 (d, J = 8.8 Hz, 2H), 5.15 (q, J = 7.4 Hz, 1H), 3.79 (s, 3H), 3.47 (s, 3H), 1.86 (d, J = 7.4 Hz, 3H). **^{13}C-NMR (75 MHz, CDCl$_3$)** δ 184.9 (CH), 158.6 (C$_q$), 154.0 (C$_q$), 137.5 (C$_q$), 133.1 (C$_q$), 128.2 (CH), 125.9 (C$_q$), 123.5 (CH), 123.1 (CH), 121.6 (CH), 114.6 (C$_q$), 114.3 (CH), 109.4 (CH), 55.4 (CH$_3$), 33.8 (CH$_3$), 31.2 (CH), 19.0 (CH$_3$). **HRMS** (ESI) m/z (M+H)$^+$: calculated for $(C_{19}H_{20}NO_2)^+$: 294.1489, found: 294.1497.

1-Methyl-2-(1-(*m*-tolyl)ethyl)-1*H*-indole-3-carbaldehyde (3v)

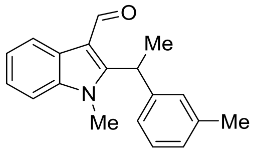

The **general procedure 1** was followed using indole substrate **1a** (0.1 mmol, 26.4 mg) and 1-methyl-3-vinylbenzene (**2j**) (0.15 mmol, 20 µL) to afford **3v** (25.0 mg, 90% yield) as a colorless oil. **^{1}H-NMR (300 MHz, CDCl$_3$)** δ 10.27 (s, 1H), 8.50 – 8.28 (m, 1H), 7.37 – 7.27 (m, 3H), 7.23 (t, J = 7.3 Hz, 1H), 7.10 – 7.03 (m, 3H), 5.17 (q, J = 7.4 Hz, 1H), 3.48 (s, 3H), 2.31 (s, 3H), 1.87 (d, J = 7.4 Hz, 3H). **^{13}C-NMR (75 MHz, CDCl$_3$)** δ 185.0 (CH), 153.9 (C$_q$), 141.0 (C$_q$), 138.7 (C$_q$), 137.5 (C$_q$), 128.9 (CH), 128.0 (CH), 127.8 (CH), 125.9 (C$_q$), 124.0 (CH), 123.5 (CH), 123.1 (CH), 121.6 (CH), 114.7 (C$_q$), 109.4 (CH), 34.5 (CH$_3$), 31.3 (CH), 21.6 (CH$_3$), 18.8 (CH$_3$). **HRMS** (ESI) m/z (M+H)$^+$: calculated for $(C_{19}H_{20}NO)^+$: 278.1539, found: 278.1540.

2-(1-(3-Methoxyphenyl)ethyl)-1-methyl-1*H*-indole-3-carbaldehyde (3w)

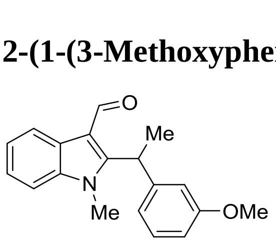

The **general procedure 1** was followed using indole substrate **1a** (0.1 mmol, 26.4 mg) and 1-methoxy-3-vinylbenzene (**2k**) (0.15 mmol, 21 µL) to afford **3w** (18.3 mg, 62% yield) as a colorless oil. **^{1}H-NMR (300 MHz, CDCl$_3$)** δ 10.27 (s, 1H), 8.41 – 8.31 (m, 1H), 7.35 – 7.27 (m, 3H), 7.24 (dd, J = 8.0, 1.1 Hz, 1H), 6.86 – 6.76 (m, 3H), 5.19

(q, J = 7.4 Hz, 1H), 3.76 (s, 3H), 3.48 (s, 3H), 1.86 (d, J = 7.3 Hz, 3H). **^{13}C-NMR (75 MHz, CDCl$_3$)** δ 184.9 (CH), 160.2 (C_q), 153.4 (C_q), 142.8 (C_q), 137.5 (C_q), 130.0 (CH), 125.9 (C_q), 123.6 (CH), 123.2 (CH), 121.5 (CH), 119.6 (CH), 114.7 (C_q), 113.6 (CH), 111.7 (CH), 109.4 (CH), 55.4 (CH_3), 34.5 (CH_3), 31.3 (CH), 18.7 (CH_3). **HRMS** (ESI) m/z $(M+H)^+$: calculated for $(C_{19}H_{20}NO_2)^+$: 294.1489, found: 294.1488.

2-(1-(2-methoxyphenyl)ethyl)-1-methyl-1*H*-indole-3-carbaldehyde (3x)

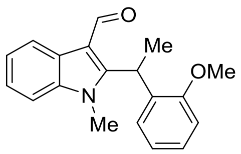

The **general procedure 1** was followed using indole substrate **1a** (0.1 mmol, 26.4 mg) and 1-methoxy-2-vinylbenzene (**2l**) (0.15 mmol, 20 μL) to afford **3x** (22.7 mg, 77% yield) as a white solid. **M.p.**: 165-167 °C. **^{1}H-NMR (300 MHz, CDCl$_3$)** δ 10.20 (s, 1H), 8.45 – 8.27 (m, 1H), 7.44 (d, J = 7.6 Hz, 1H), 7.33 – 7.22 (m, 4H), 7.00 (td, J = 7.5, 1.0 Hz, 1H), 6.82 (dd, J = 8.2, 1.1 Hz, 1H), 5.01 (q, J = 7.4 Hz, 1H), 3.72 (s, 3H), 3.64 (s, 3H), 1.85 (d, J = 7.4 Hz, 3H). **^{13}C-NMR (75 MHz, CDCl$_3$)** δ 185.9 (CH), 157.2 (C_q), 154.9 (C_q), 137.0 (C_q), 130.0 (C_q), 128.6 (CH), 127.1 (CH), 126.0 (C_q), 123.1 (CH), 122.9 (CH), 122.1 (CH), 120.9 (CH), 114.1 (C_q), 110.8 (CH), 109.3 (CH), 55.5 (CH_3), 31.2 (CH_3), 30.5 (CH), 20.1 (CH_3). **HRMS** (ESI) m/z $(M+H)^+$: calculated for $(C_{19}H_{20}NO_2)^+$: 294.1489, found: 294.1491.

2-(1-(3,4-Dimethoxyphenyl)ethyl)-1-methyl-1*H*-indole-3-carbaldehyde (3y)

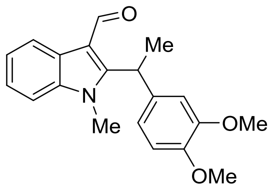

The **general procedure 1** was followed using indole substrate **1a** (0.1 mmol, 26.4 mg) and 1,2-dimethoxy-4-vinylbenzene (**2m**) (0.15 mmol, 22 μL) to afford **3y** (24.4 mg, 76% yield) as a colorless oil. **^{1}H-NMR (300 MHz, CDCl$_3$)** δ 10.27 (s, 1H), 8.43 – 8.33 (m, 1H), 7.35 – 7.25 (m, 3H), 6.83 (d, J = 1.2 Hz, 2H), 6.72 (q, J = 1.1 Hz, 1H), 5.17

(q, J = 7.3 Hz, 1H), 3.87 (s, 3H), 3.77 (s, 3H), 3.49 (s, 3H), 1.85 (d, J = 7.3 Hz, 3H). **^{13}C-NMR (75 MHz, CDCl$_3$)** δ 185.0 (CH), 153.8 (C_q), 149.5 (C_q), 148.2 (C_q), 137.6 (C_q), 133.6 (C_q), 125.9 (C_q), 123.6 (CH), 123.2 (CH), 121.6 (CH), 119.1 (CH), 114.6 (C_q), 111.4 (CH), 110.8 (CH), 109.5 (CH), 56.2 (CH_3), 56.1 (CH_3), 34.2 (CH_3), 31.3 (CH), 19.0 (CH_3). **HRMS** (ESI) m/z $(M+H)^+$: calculated for $(C_{20}H_{22}NO_3)^+$: 324.1594, found: 324.1599.

1-Methyl-2-(1-(naphthalen-2-yl)ethyl)-1*H*-indole-3-carbaldehyde (3z)

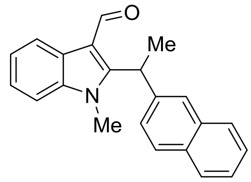

The **general procedure 1** was followed using indole substrate **1a** (0.1 mmol, 26.4 mg) and 2-vinylnaphthalene (**2n**) (0.15 mmol, 23.1 mg) to afford **3z** (28.9 mg, 92% yield) as a colorless oil. **^{1}H-NMR (300 MHz, CDCl$_3$)** δ 10.33 (s, 1H), 8.48 – 8.35 (m, 1H), 7.88 – 7.75 (m, 4H), 7.56 – 7.45 (m, 2H), 7.41 – 7.24 (m, 4H), 5.39 (q, J = 7.2 Hz, 1H), 3.46 (s, 3H), 2.00 (d, J = 7.4 Hz, 3H). **^{13}C-NMR (75 MHz, CDCl$_3$)** δ 185.0 (CH), 153.3 (C_q), 138.6 (C_q), 137.5 (C_q), 133.5 (C_q), 132.4 (C_q), 128.8 (CH), 128.0 (CH), 127.8 (CH), 126.6 (CH), 126.2 (CH), 126.0 (C_q), 125.9 (CH), 125.1 (CH), 123.6 (CH), 123.2 (CH), 121.5 (CH), 114.8 (C_q), 109.5 (CH), 34.8 (CH_3), 31.3 (CH), 18.8 (CH_3). **HRMS** (ESI) m/z $(M+H)^+$: calculated for $(C_{22}H_{20}NO)^+$: 314.1539, found: 314.1537.

1-Methyl-2-(1-ferrocenylethyl)-1*H*-indole-3-carbaldehyde (3aa)

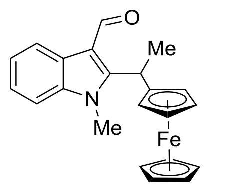

The **general procedure 1** was followed at 60 °C using indole substrate **1a** (0.1 mmol, 26.4 mg) and vinylferrocene (**2o**) (0.15 mmol, 31.8 mg) to afford **3aa** (14.0 mg, 38% yield) as a red solid. **M.p.**: 157-159 °C. **^{1}H-NMR (300 MHz, CDCl$_3$)** δ 10.25 (s, 1H), 8.38 – 8.25 (m, 1H), 7.38 – 7.19 (m, 3H), 5.17 (q, J = 7.4 Hz, 1H), 4.44 (ddt, J =

2.8, 1.8, 0.9 Hz, 1H), 4.19 (s, 5H), 4.16 (td, J = 2.5, 1.3 Hz, 1H), 4.13 (td, J = 2.5, 1.3 Hz, 1H), 4.03 (dt, J = 2.6, 1.3 Hz, 1H), 3.55 (s, 3H), 1.77 (d, J = 7.4 Hz, 3H). **^{13}C-NMR (75 MHz, CDCl$_3$)** δ 184.7 (CH), 154.1 (C$_q$), 137.4 (C$_q$), 125.9 (C$_q$), 123.4 (CH), 123.0 (CH), 121.4 (CH), 113.4 (C$_q$), 109.4 (CH), 89.7 (C$_q$), 69.2 (CH), 68.9 (CH), 68.4 (CH), 67.2 (CH), 66.9 (CH), 31.3 (CH$_3$), 31.1 (CH), 19.0 (CH$_3$). **HRMS** (ESI) m/z $(M+H)^+$: calculated for $(C_{22}H_{22}FeNO)^+$: 372.1045, found: 372.1050.

1-Methyl-2-(1-phenylpropyl)-1*H*-indole-3-carbaldehyde (3ab)

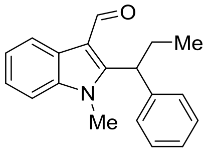

The **general procedure 1** was followed using indole substrate **1a** (0.1 mmol, 26.4 mg) and (*Z*)-prop-1-en-1-ylbenzene (**2p**) (0.15 mmol, 19 μL) to afford **3ab** (14.0 mg, 51% yield) as a white solid. M.p.: 112-114 °C. **^{1}H-NMR (300 MHz, CDCl$_3$)** δ 10.31 (s, 1H), 8.43 – 8.36 (m, 1H), 7.36 – 7.19 (m, 8H), 4.94 (dd, J = 10.1, 6.0 Hz, 1H), 3.49 (s, 3H), 2.63 – 2.44 (m, 1H), 2.29 – 2.12 (m, 1H), 1.01 (t, J = 7.3 Hz, 3H). **^{13}C-NMR (75 MHz, CDCl$_3$)** δ 185.2 (CH), 152.0 (C$_q$), 140.6 (C$_q$), 137.5 (C$_q$), 129.0 (CH), 127.6 (CH), 127.1 (CH), 125.9 (C$_q$), 123.6 (CH), 123.2 (CH), 121.6 (CH), 116.2 (C$_q$), 109.4 (CH), 42.3 (CH), 31.3 (CH$_3$), 25.6 (CH$_2$), 12.8 (CH$_3$). **HRMS** (ESI) m/z $(M+H)^+$: calculated for $(C_{19}H_{20}NO)^+$: 278.1539, found: 278.1541.

1-Methyl-2-(1-((8*R*,9*S*,13*S*,14*S*)-13-methyl-17-oxo-7,8,9,11,12,13,14,15,16,17-decahydro-6*H*-cyclopenta[*a*]phenanthren-3-yl)ethyl)-1*H*-indole-3-carbaldehyde (3ac)

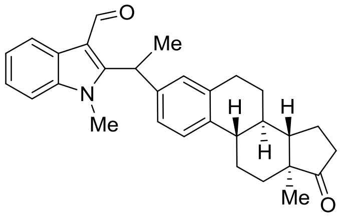

The **general procedure 1** was followed using indole substrate **1a** (0.1 mmol, 26.4 mg) and (8*R*,9*S*,13*S*,14*S*)-13-methyl-3-vinyl-6,7,8,9,11,12,13,14,15,16-decahydro-17*H*-

cyclopenta[*a*]phenanthren-17-one (**2q**) (0.15 mmol, 42.0 mg) to afford **3ac** (34.4 mg, 78% yield) as a white solid. M.p.: 203-205 °C. **^{1}H-NMR (300 MHz, CDCl$_3$)** δ 10.24 (s, 1H), 8.56 – 8.28 (m, 1H), 7.35 – 7.29 (m, 3H), 7.28 – 7.22 (m, 1H), 7.09 – 7.00 (m, 1H), 6.96 (d, *J* = 9.8 Hz, 1H), 5.10 (q, *J* = 7.4 Hz, 1H), 3.53 (s, 3H), 2.93 – 2.75 (m, 2H), 2.57 – 1.92 (m, 7H), 1.86 (d, *J* = 7.4 Hz, 3H), 1.68 – 1.31 (m, 6H), 0.91 (s, 3H). **^{13}C-NMR (75 MHz, CDCl$_3$)** δ 220.9 (C_q), 185.1 (CH), 154.0 (C_q), 138.6 (C_q), 138.6 (C_q), 138.5 (C_q), 137.5 (C_q), 137.2 (C_q), 127.8 (CH), 125.9 (CH), 124.4 (CH), 123.5 (CH), 123.1 (CH), 121.7 (CH), 114.6 (C_q), 109.4 (CH), 50.6 (CH), 48.1 (C_q), 44.4 (CH), 38.2 (CH), 36.0 (CH_2), 34.3 (CH), 31.7 (CH_2), 31.3 (CH_3), 29.6 (CH_2), 26.6 (CH_2), 25.8 (CH_2), 21.7 (CH_2), 19.0 (CH_3), 14.0 (CH_3). **HRMS** (ESI) m/z (M+H)$^+$: calculated for $(C_{30}H_{34}NO_2)^+$: 440.2584, found: 440.2588.

2-(1-((*R*)-2,8-Dimethyl-2-((4*R*,8*R*)-4,8,12-trimethyltridecyl)chroman-6-yl)ethyl)-1-methyl-1*H*-indole-3-carbaldehyde (3ad)

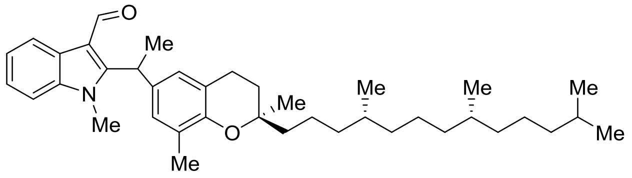

The **general procedure 1** was followed using indole substrate **1a** (0.1 mmol, 26.4 mg) and (*R*)-2,8-dimethyl-2-((4*R*,8*R*)-4,8,12-trimethyltridecyl)-6-vinylchromane (**2r**) (0.15 mmol, 61.9 mg) to afford **3ad** (38.9 mg, 68% yield) as a colorless oil. **^{1}H-NMR (300 MHz, CDCl$_3$)** δ 10.26 (s, 1H), 8.53 – 8.26 (m, 1H), 7.39 – 7.22 (m, 3H), 6.91 – 6.80 (m, 1H), 6.80 – 6.70 (m, 1H), 5.05 (q, *J* = 7.3 Hz, 1H), 3.52 (s, 3H), 2.78 – 2.59 (m, 2H), 2.13 (d, *J* = 1.8 Hz, 3H), 1.89 – 1.68 (m, 5H), 1.60 – 1.01 (m, 24H), 0.98 – 0.73 (m, 12H). **^{13}C-NMR (75 MHz, CDCl$_3$)** δ 185.1 (CH), 154.7 (C_q), 151.1 (C_q), 137.5 (C_q), 130.9 (C_q), 127.0 (CH), 126.8 (C_q), 125.9 (C_q), 125.5 (CH), 123.4 (CH), 123.1 (CH), 121.7 (CH), 120.9 (C_q), 114.6 (C_q), 109.4 (CH), 76.3 (C_q), 40.4 (CH_2), 40.3 (CH_2), 39.5 (CH_2), 37.6 (CH_2), 37.6 (CH_2), 37.4 (CH_2), 33.9 (CH), 32.9 (CH), 32.8 (CH), 31.3 (CH_2), 28.1 (CH), 24.9 (CH_2), 24.9 (CH_2), 24.3 (CH_3), 24.3 (CH_3), 22.9 (CH_3), 22.8 (CH_3), 22.5 (CH_2), 21.1 (CH_2), 19.9 (CH_3), 19.8 (CH_3), 19.1 (CH_3),

16.3 (CH_3). **HRMS** (ESI) m/z $(M+H)^+$: calculated for $(C_{39}H_{58}NO_2)^+$: 572.4462, found: 572.4460.

1-Methyl-2-(2-(trimethylsilyl)ethyl)-1*H*-indole-3-carbaldehyde (5a)

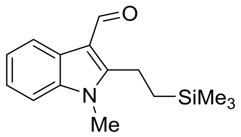

The **general procedure 2** was followed using indole substrate **1a** (0.1 mmol, 26.4 mg) and trimethyl(vinyl)silane (**4a**) (0.15 mmol, 22 µL) to afford **5a** (20.2 mg, 78% yield) as a pale-yellow solid. M.p.: 120-122 °C. **^{1}H-NMR (300 MHz, $CDCl_3$)** δ 10.16 (s, 1H), 8.31 – 8.23 (m, 1H), 7.35 – 7.23 (m, 3H), 3.71 (s, 3H), 3.08 – 2.99 (m, 2H), 0.93 – 0.79 (m, 2H), 0.11 (s, 9H). **^{13}C-NMR (75 MHz, $CDCl_3$)** δ 183.9 (CH), 154.7 (C_q), 137.2 (C_q), 125.9 (C_q), 123.2 (CH), 123.0 (CH), 121.2 (CH), 112.8 (C_q), 109.4 (CH), 29.7 (CH_3), 19.0 (CH_2), 18.2 (CH_2), -1.8 (CH_3). **HRMS** (ESI) m/z $(M+H)^+$: calculated for $(C_{15}H_{22}NOSi)^+$: 260.1465, found: 260.1465.

1-Benzyl-2-(2-(trimethylsilyl)ethyl)-1*H*-indole-3-carbaldehyde (5b)

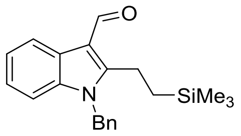

The **general procedure 2** was followed using indole substrate **1b** (0.1 mmol, 34.0 mg) and trimethyl(vinyl)silane (**4a**) (0.15 mmol, 22 µL) to afford **5b** (21.1 mg, 63% yield) as a pale-yellow solid. M.p.: 127-129 °C. **^{1}H-NMR (300 MHz, $CDCl_3$)** δ 10.22 (s, 1H), 8.31 (d, J = 7.6 Hz, 1H), 7.38 – 7.16 (m, 6H), 7.01 (dd, J = 7.3, 2.1 Hz, 2H), 5.35 (s, 2H), 3.09 – 2.89 (m, 2H), 0.88 – 0.69 (m, 2H), 0.04 (s, 9H). **^{13}C-NMR (75 MHz, $CDCl_3$)** δ 184.2 (CH), 154.9 (C_q), 137.0 (C_q), 136.2 (C_q), 129.2 (CH), 128.1 (CH), 126.1 (CH), 123.5 (CH), 123.1 (CH), 121.2 (CH), 113.3 (C_q), 110.1 (CH), 46.8 (CH_2), 19.2 (CH_2), 18.9 (CH_2), -1.9 (CH_3). **HRMS** (ESI) m/z $(M+H)^+$: calculated for $(C_{21}H_{26}NOSi)^+$: 336.1778, found: 336.1780.

1-Phenyl-2-(2-(trimethylsilyl)ethyl)-1*H*-indole-3-carbaldehyde (5c)

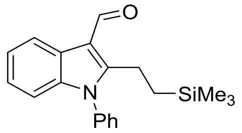

The **general procedure 2** was followed using indole substrate **1c** (0.1 mmol, 32.6 mg) and trimethyl(vinyl)silane (**4a**) (0.15 mmol, 22 µL) to afford **5c** (15.0 mg, 47% yield) as a white solid. M.p.: 105-107 °C. **^{1}H-NMR (300 MHz, $CDCl_3$)** δ 10.28 (s, 1H), 8.32 (d, *J* = 7.8 Hz, 1H), 7.67 – 7.53 (m, 3H), 7.41 – 7.34 (m, 2H), 7.30 (ddd, *J* = 7.8, 7.2, 1.1 Hz, 1H), 7.20 (ddd, *J* = 8.3, 7.2, 1.3 Hz, 1H), 7.00 (d, *J* = 8.1 Hz, 1H), 3.01 – 2.84 (m, 2H), 0.83 – 0.66 (m, 2H), -0.12 (s, 9H). **^{13}C-NMR (75 MHz, $CDCl_3$)** δ 184.6 (CH), 155.3 (C_q), 138.4 (C_q), 136.1 (C_q), 130.0 (CH), 129.4 (CH), 128.3 (CH), 125.7 (C_q), 123.5 (CH), 123.3 (CH), 121.1 (CH), 113.6 (C_q), 110.7 (CH), 19.4 (CH_2), 19.0 (CH_2), -2.1 (CH_3). **HRMS** (ESI) m/z $(M+H)^+$: calculated for $(C_{20}H_{24}NOSi)^+$: 322.1622, found: 322.1629.

1-Methyl-2-(2-(trimethylsilyl)ethyl)-1*H*-pyrrolo[2,3-*b*]pyridine-3-carbaldehyde (5d)

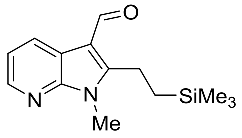

The **general procedure 2** was followed using indole substrate **1e** (0.1 mmol, 26.5 mg) and trimethyl(vinyl)silane (**4a**) (0.15 mmol, 22 µL) to afford **5d** (18.3 mg, 70% yield) as a pale-yellow solid. M.p.: 132-133 °C. **^{1}H-NMR (300 MHz, $CDCl_3$)** δ 10.16 (s, 1H), 8.49 (d, *J* = 7.8 Hz, 1H), 8.34 (d, *J* = 4.8 Hz, 1H), 7.21 (dd, *J* = 7.8, 4.9 Hz, 1H), 3.85 (s, 3H), 3.19 – 3.00 (m, 2H), 1.01 – 0.85 (m, 2H), 0.12 (s, 9H). **^{13}C-NMR (75 MHz, $CDCl_3$)** δ 183.8 (CH), 155.3 (C_q), 148.5 (C_q), 143.9 (CH), 129.4 (CH), 119.0 (CH), 118.6 (C_q), 111.2 (C_q), 28.3 (CH_3), 19.3 (CH_2), 18.1 (CH_2), -1.8 (CH_3). **HRMS** (ESI) m/z $(M+H)^+$: calculated for $(C_{14}H_{21}N_2OSi)^+$: 261.1418, found: 261.1418.

5-Fluoro-1-methyl-2-(2-(trimethylsilyl)ethyl)-1*H*-indole-3-carbaldehyde (5e)

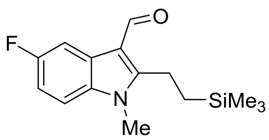

The **general procedure 2** was followed using indole substrate **1f** (0.1 mmol, 28.2 mg) and trimethyl(vinyl)silane (**4a**) (0.15 mmol, 22 μL) to afford **5e** (16.4 mg, 59% yield) as a white solid. M.p.: 124-126 °C. **^{1}H-NMR (300 MHz, $CDCl_3$)** δ 10.11 (s, 1H), 7.94 (dd, J = 9.4, 2.5 Hz, 1H), 7.22 (dd, J = 8.8, 4.2 Hz, 1H), 7.00 (td, J = 9.0, 2.6 Hz, 1H), 3.70 (s, 3H), 3.11 – 2.91 (m, 2H), 0.96 – 0.81 (m, 2H), 0.11 (s, 9H). **^{13}C-NMR (75 MHz, $CDCl_3$)** δ 183.7 (CH), 160.1 (d, J = 238.2 Hz, C_q), 155.7 (C_q), 133.7 (C_q), 126.6 (d, J = 11.0 Hz, C_q), 112.9 (d, J = 4.2 Hz, C_q), 111.2 (d, J = 26.1 Hz, CH), 110.1 (d, J = 9.8 Hz, CH), 106.9 (d, J = 24.9 Hz, CH), 29.9 (CH_3), 19.1 (CH_2), 18.2 (CH_2), -1.8 (CH_3). **^{19}F-NMR (282 MHz, $CDCl_3$)** δ -120.7. **HRMS** (ESI) m/z $(M+H)^+$: calculated for $(C_{15}H_{21}FNOSi)^+$: 278.1371, found: 278.1373.

5-Methoxy-1-methyl-2-(2-(trimethylsilyl)ethyl)-1*H*-indole-3-carbaldehyde (5f)

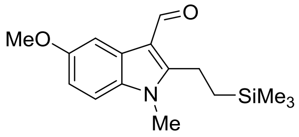

The **general procedure 2** was followed using indole substrate **1i** (0.1 mmol, 29.4 mg) and trimethyl(vinyl)silane (**4a**) (0.15 mmol, 22 μL) to afford **5f** (20.4 mg, 71% yield) as a white solid. M.p.: 117-119 °C. **^{1}H-NMR (300 MHz, $CDCl_3$)** δ 10.10 (s, 1H), 7.80 (d, J = 2.5 Hz, 1H), 7.18 (d, J = 8.8 Hz, 1H), 6.90 (dd, J = 8.8, 2.5 Hz, 1H), 3.89 (s, 3H), 3.67 (s, 3H), 3.05 – 2.86 (m, 2H), 0.98 – 0.75 (m, 2H), 0.10 (s, 9H). **^{13}C-NMR (75 MHz, $CDCl_3$)** δ 183.8 (CH), 156.8 (C_q), 154.8 (C_q), 132.0 (C_q), 126.5 (C_q), 113.2 (CH), 112.8 (C_q), 110.1 (CH), 103.1 (CH), 56.0 (CH_3), 29.8 (CH_3), 19.0 (CH_2), 18.3 (CH_2), -1.8 (CH_3). **HRMS** (ESI) m/z $(M+H)^+$: calculated for $(C_{16}H_{24}NO_2Si)^+$: 290.1571, found: 290.1575.

1-Methyl-2-(2-(triethylsilyl)ethyl)-1*H*-indole-3-carbaldehyde (5g)

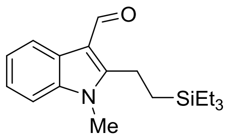

The **general procedure 2** was followed using indole substrate **1a** (0.1 mmol, 26.4 mg) and triethyl(vinyl)silane (**4b**) (0.15 mmol, 28 μL) to afford **5g** (22.6 mg, 75% yield) as a white solid. M.p.: 111-114 °C. **^{1}H-NMR (300 MHz, $CDCl_3$)** δ 10.17 (s, 1H), 8.36 – 8.14 (m, 1H), 7.36 – 7.21 (m, 3H), 3.73 (s, 3H), 3.16 – 2.92 (m, 2H), 1.01 (t, J = 7.9 Hz, 9H), 0.98 – 0.82 (m, 2H), 0.64 (q, J = 7.9 Hz, 6H). **^{13}C-NMR (75 MHz, $CDCl_3$)** δ 183.9 (CH), 154.9 (C_q), 137.2 (C_q), 125.9 (C_q), 123.2 (CH), 123.0 (CH), 121.2 (CH), 112.8 (C_q), 109.4 (CH), 29.7 (CH_3), 19.0 (CH_2), 13.3 (CH_2), 7.6 (CH_3), 3.2 (CH_2). **HRMS** (ESI) m/z $(M+H)^+$: calculated for $(C_{18}H_{28}NOSi)^+$: 302.1935, found: 302.1936.

1-Methyl-2-(2-(triphenylsilyl)ethyl)-1*H*-indole-3-carbaldehyde (5h)

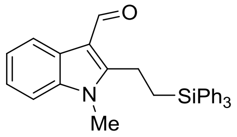

The **general procedure 2** was followed using indole substrate **1a** (0.1 mmol, 26.4 mg) and triphenyl(vinyl)silane (**4c**) (0.15 mmol, 43.0 mL) to afford **5h** (28.9 mg, 65% yield) as a white solid. M.p.: 180-182 °C. **^{1}H-NMR (300 MHz, $CDCl_3$)** δ 10.01 (s, 1H), 8.30 – 8.17 (m, 1H), 7.70 – 7.53 (m, 6H), 7.51 – 7.33 (m, 9H), 7.31 – 7.16 (m, 3H), 3.51 (s, 3H), 3.19 – 3.08 (m, 2H), 1.82 – 1.64 (m, 2H). **^{13}C-NMR (75 MHz, $CDCl_3$)** δ 184.0 (CH), 153.9 (C_q), 137.2 (C_q), 135.7 (CH), 133.9 (C_q), 130.1 (CH), 128.4 (CH), 125.9 (C_q), 123.3 (CH), 123.0 (CH), 121.2 (CH), 113.1 (C_q), 109.4 (CH), 29.6 (CH_3), 19.2 (CH_2), 15.2 (CH_2). **HRMS** (ESI) m/z $(M+H)^+$: calculated for $(C_{30}H_{28}NOSi)^+$: 446.1935, found: 446.1931.

5. Synthetic transformations

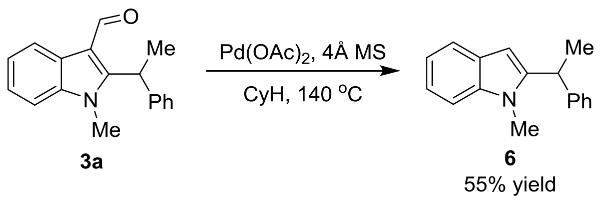

To a solution of **3** (0.15 mmol, 39.5 mg) and 4Å molecular sieves (75 mg) in anhydrous cyclohexane (1.0 mL), was added $Pd(OAc)_2$ (15 mol%, 0.0225 mmol, 5.1 mg). After stirring at 140 °C for 24 hours, the reaction mixture was cooled to room temperature, filtered through a pad of Celite and washed with ethyl acetate (10 mL). The filtrate was concentrated and the crude product was purified by column chromatography on silica gel (*n*-hexane: ethyl acetate = 50:1) to afford the desired product **6** (19.4 mg, 55% yield).

1-Methyl-2-(1-phenylethyl)-1*H*-indole (6)

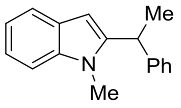

Colorless oil. **^{1}H-NMR (300 MHz, $CDCl_3$)** δ 7.62 (d, J = 7.7 Hz, 1H), 7.31 – 7.24 (m, 1H), 7.27 – 7.16 (m, 3H), 7.19 – 7.11 (m, 3H), 7.09 (td, J = 7.4, 7.0, 1.2 Hz, 1H), 6.52 (t, J = 1.0 Hz, 1H), 4.25 (q, J = 7.1 Hz, 1H), 3.41 (s, 3H), 1.71 (d, J = 7.1 Hz, 3H). **^{13}C-NMR (75 MHz, $CDCl_3$)** δ 145.0 (C_q), 144.0 (C_q), 137.8 (C_q), 128.8 (CH), 127.7 (C_q), 127.5 (CH), 126.6 (CH), 121.1 (CH), 120.3 (CH), 119.4 (CH), 108.9 (CH), 99.3 (CH), 38.3 (CH_3), 29.9 (CH), 22.8 (CH_3). **HRMS** (ESI) m/z $(M+H)^+$: calculated for $(C_{17}H_{18}N)^+$: 236.1434, found: 236.14.

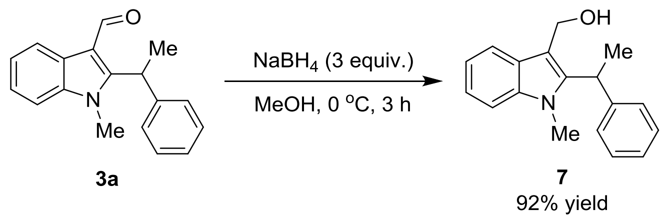

To a solution of **3a** (0.15 mmol, 39.5 mg) in MeOH (1.5 mL) was added $NaBH_4$ (0.45 mmol, 17.3 mg) portionwise at 0 °C under nitrogen. The resulting mixture was

stirred for 3 hours and then quenched by NaOH aqueous solution (2.0 M, 2.0 mL). The solution was stirred for 10 min and then CH_2Cl_2 (10.0 mL) was added. The phases were separated, the aqueous layer was extracted with CH_2Cl_2 (5.0 mL ×3) and the combined organic layer was washed with brine, dried over Na_2SO_4 and concentrated *in vacuo* to give the product **7** (36.4 mg, 92% yield).

(1-Methyl-2-(1-phenylethyl)-1*H*-indol-3-yl)methanol (7)

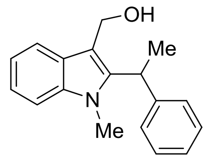

Colorless oil. **^{1}H-NMR (300 MHz, $CDCl_3$)** δ 7.79 – 7.65 (m, 1H), 7.41 – 7.21 (m, 7H), 7.18 (ddd, *J* = 8.1, 6.6, 1.6 Hz, 1H), 4.88 (d, *J* = 1.6 Hz, 2H), 4.74 (q, *J* = 7.4 Hz, 1H), 3.47 (s, 3H), 1.85 (d, *J* = 7.4 Hz, 3H). **^{13}C-NMR (75 MHz, $CDCl_3$)** δ 143.0 (C_q), 142.0 (C_q), 137.0 (C_q), 128.7 (CH), 127.3 (C_q), 127.3 (CH), 126.5 (CH), 121.7 (CH), 119.8 (CH), 118.3 (CH), 111.9 (C_q), 109.0 (CH), 55.8 (CH_2), 35.2 (CH_3), 30.7 (CH), 19.3 (CH_3). **HRMS** (ESI) m/z $(M+Na)^+$: calculated for $(C_{18}H_{19}NONa)^+$: 288.1359, found: 288.1361.

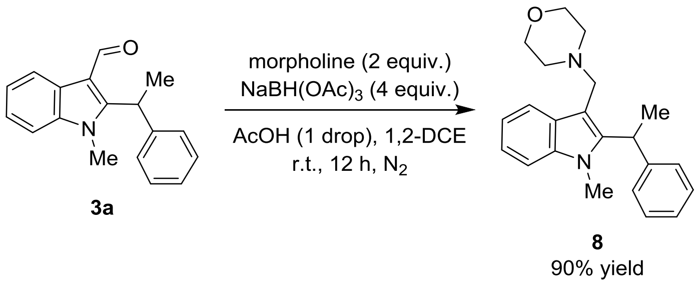

A solution of **3a** (0.15 mmol, 39.5 mg), morpholine (0.3 mmol, 25 μL), $NaBH(OAc)_3$ (0.6 mmol, 127.2 mg) and AcOH (1 drop) in 1,2-dichloroethane (2.0 mL) was stirred at room temperature for 12 hours. The reaction mixture was diluted with CH_2Cl_2 (5.0 mL), quenched by saturated $NaHCO_3$ aqueous solution (5.0 mL). The phases were separated and the aqueous layer was extracted with CH_2Cl_2 (5.0 mL ×3). The combined organic layer was washed with brine, dried over Na_2SO_4, filtered and concentrated *in vacuo*. The residue was purified by column chromatography on silica gel (*n*-hexane:

ethyl acetate = 10:1) to afford the desired product **8** (45.2 mg, 90% yield).

4-((1-Methyl-2-(1-phenylethyl)-1*H*-indol-3-yl)methyl)morpholine (8)

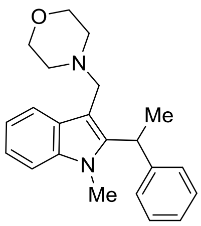

Pale-yellow oil. **^{1}H-NMR (300 MHz, $CDCl_3$)** δ 7.73 (d, *J* = 7.8 Hz, 1H), 7.30 (d, *J* = 4.5 Hz, 4H), 7.25 – 7.17 (m, 3H), 7.13 (ddd, *J* = 8.0, 6.7, 1.5 Hz, 1H), 4.82 (q, *J* = 7.4 Hz, 1H), 3.73 – 3.58 (m, 6H), 3.40 (s, 3H), 2.48 (s, 4H), 1.81 (d, *J* = 7.4 Hz, 3H). **^{13}C-NMR (75 MHz, $CDCl_3$)** δ 143.2 (C_q), 142.1 (C_q), 136.9 (C_q), 128.7 (C_q), 128.5 (CH), 127.4 (CH), 126.3 (CH), 121.1 (CH), 119.3 (CH), 118.8 (CH), 108.7 (CH), 108.2 (C_q), 67.4 (CH_2), 53.7 (CH_2), 52.9 (CH_2), 35.0 (CH_3), 30.8 (CH), 18.4 (CH_3). **HRMS** (ESI) m/z $(M+Na)^+$: calculated for $(C_{22}H_{26}N_2ONa)^+$: 357.1937, found: 357.1937.

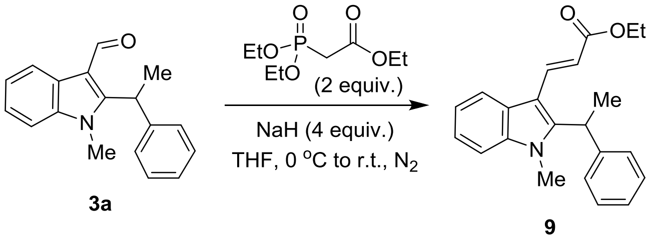

3a

9
84% yield

To a solution of ethyl 2-(diethoxyphosphoryl)acetate (0.3 mmol, 67.2 mg) in anhydrous THF (2.0 mL) under nitrogen, was added portionwise NaH (60% dispersion in mineral oil, 0.6 mmol, 24.0 mg) at 0 °C. After stirring for 1 hours, **3a** (0.15 mmol, 39.5 mg) was added and the resulting mixture was allowed to warm up to room temperature and stirred overnight. The reaction mixture was quenched by saturated NH_4Cl aqueous solution (2.0 mL). Then, diethyl ether (10 mL) and water (10 mL) were added. The phases were separated and the aqueous layer was extracted with diethyl ether (5.0 mL ×3). The combined organic layer was washed with brine, dried over Na_2SO_4, filtered and concentrated *in vacuo*. The crude product was purified by column

chromatography on silica gel (*n*-hexane: ethyl acetate = 10:1) to afford the desired product **9** (42.0 mg, 84% yield).

Ethyl (*E*)-3-(1-methyl-2-(1-phenylethyl)-1*H*-indol-3-yl)acrylate (9)

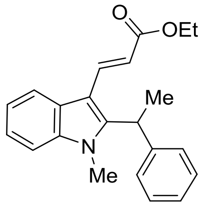

Pale-yellow oil. **^{1}H-NMR (300 MHz, $CDCl_3$)** δ 8.08 (d, *J* = 15.7 Hz, 1H), 8.01 – 7.92 (m, 1H), 7.35 – 7.16 (m, 8H), 6.52 (d, *J* = 15.7 Hz, 1H), 4.94 (q, *J* = 7.3 Hz, 1H), 4.25 (q, *J* = 7.2 Hz, 2H), 3.37 (s, 3H), 1.81 (d, *J* = 7.4 Hz, 3H), 1.34 (t, *J* = 7.1 Hz, 3H). **^{13}C-NMR (75 MHz, $CDCl_3$)** δ 168.8 (C_q), 147.7 (C_q), 141.7 (C_q), 138.2 (C_q), 137.7 (CH), 128.9 (CH), 127.1 (CH), 126.7 (CH), 125.7 (C_q), 122.7 (CH), 121.6 (CH), 120.7 (CH), 113.1 (CH), 109.8 (C_q), 109.5 (CH), 60.2 (CH_2), 34.3 (CH_3), 31.3 (CH), 18.3 (CH_3), 14.6 (CH_3). **HRMS** (ESI) m/z $(M+Na)^+$: calculated for $(C_{22}H_{23}NO_2Na)^+$: 356.1621, found: 356.1633.

6. Mechanistic investigations

6.1 Kinetic isotope effect experiment

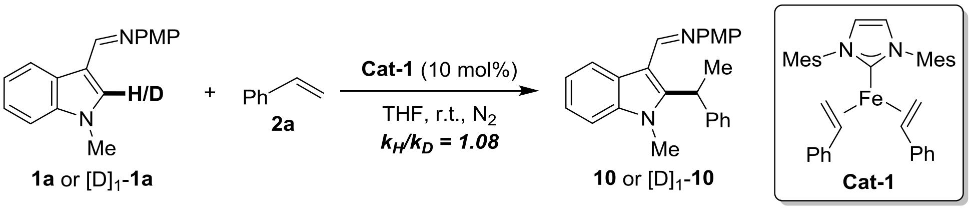

The kinetic isotope effect (KIE) was examined by applying the initial rate method. To a flame-dried and N_2-purged Schlenk tube were added indole substrate **1a** (0.2 mmol), **Cat-1** (10 mol%, 0.02 mmol) and 1,3,5-trimethoxybenzene (0.066 mmol) in the glove box. Styrene (**2a**) (0.3 mmol) and tetrahydrofuran (0.6 mL) were added *via* syringe under nitrogen atmosphere and the resulting mixture was stirred at room temperature (t = 0 min). Aliquots (50 µL) were removed periodically every 20 min. The conversion was determined by ^{1}H-NMR using 1,3,5-trimethoxybenzene as the internal standard. Then the same procedure was applied with indole substrate $[D]_1$-**1a**.

The following results were obtained.

Table S1. Kinetic isotope effect 1

Entry	Time (min)	Yield (%) (with **1a**)	Yield (%) (with $[D]_1$-**1a**)
1	20	7	8
2	40	12	13
3	60	18	20
4	80	24	25
5	100	27	29
6	120	34	32

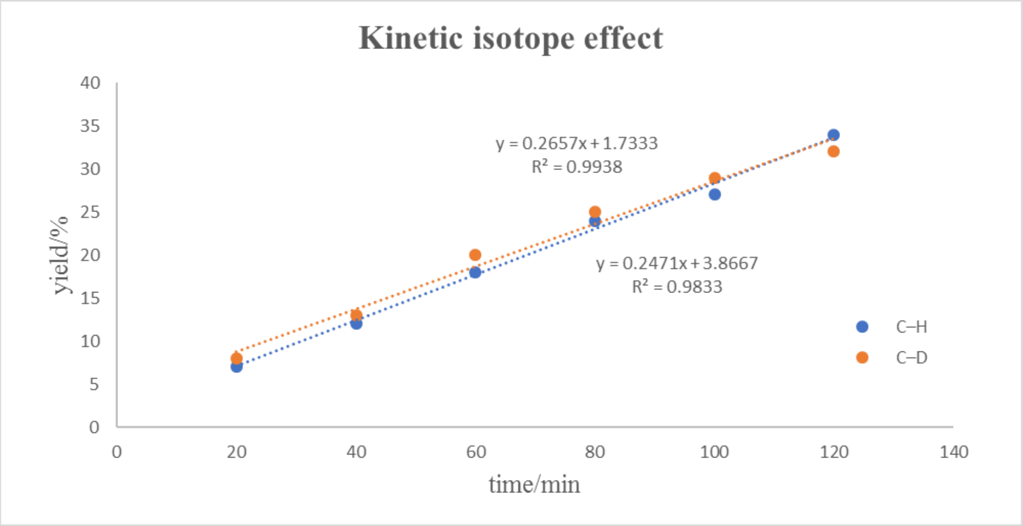

Figure S1. Kinetic isotope effect 1

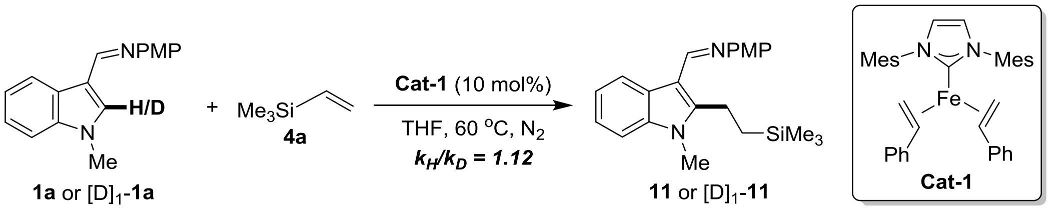

The kinetic isotope effect (KIE) was examined by applying the initial rate method. To a flame-dried and N_2-purged Schlenk tube were added indole substrate **1a** (0.2 mmol), **Cat-1** (10 mol%, 0.02 mmol) and 1,3,5-trimethoxybenzene (0.066 mmol) in the glove box. Trimethyl(vinyl)silane (**4a**) (0.3 mmol) and tetrahydrofuran (0.6 mL) were added *via* syringe under nitrogen atmosphere and the resulting mixture was stirred at 60 °C (t = 0 min). Aliquots (50 µL) were removed periodically every 20 min. The conversion was determined by ^{1}H-NMR using 1,3,5-trimethoxybenzene as the internal standard. Then the same procedure was applied with indole substrate $[D]_1$-**1a**.

The following results were obtained.

Table S2. Kinetic isotope effect 2

Entry	Time (min)	Yield (%) (with **1a**)	Yield (%) (with $[D]_1$-**1a**)
1	20	5	4
2	40	8	7
3	60	11	9
4	80	12	10
5	100	14	12
6	120	16	14

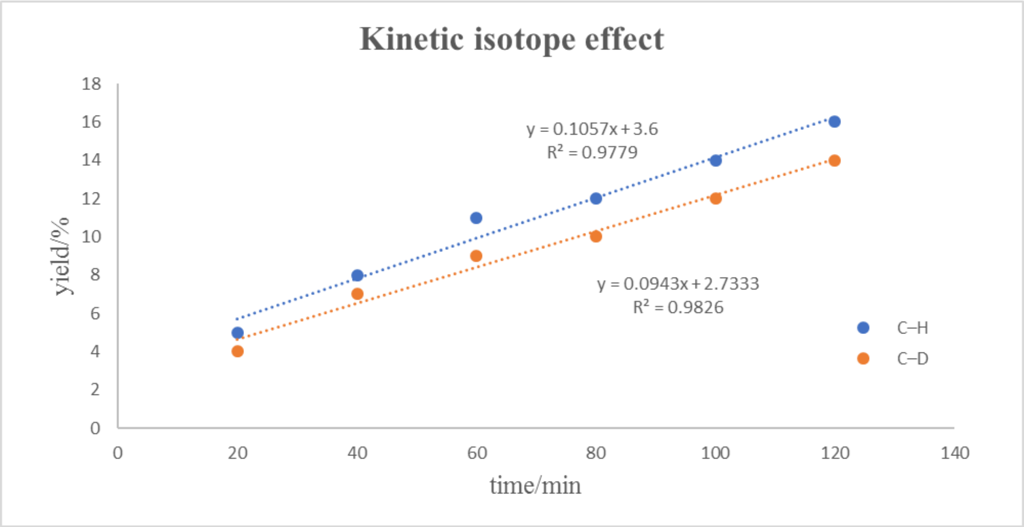

Figure S2. Kinetic isotope effect 2

6.2 Deuterium-labeling experiment

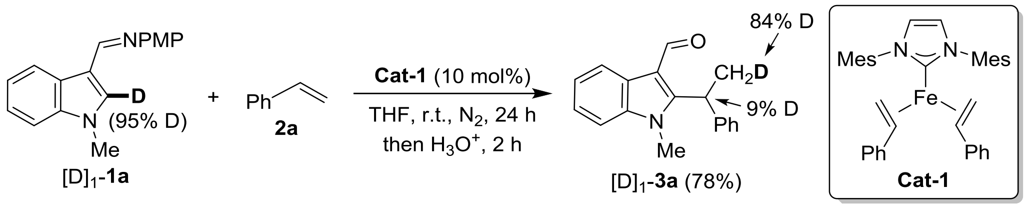

To a flame-dried and N_2-purged Schlenk tube were added indole substrate $[D]_1$-**1a** (0.1 mmol, 26.5 mg) and **Cat-1** (10 mol%, 0.01 mmol, 5.7 mg) in the glove box. Styrene (**2a**) (0.15 mmol, 17 μL) and tetrahydrofuran (0.3 mL) were added *via* syringe under nitrogen atmosphere, the resulting mixture was stirred at room temperature for 24 hours. Then, the reaction mixture was diluted with tetrahydrofuran (2.0 mL) and quenched with HCl aqueous solution (1 M, 1.0 mL). The resulting mixture was stirred at room temperature for 2 hours. The phases were then separated, the aqueous layer was extracted with ethyl acetate (5.0 mL ×3). The combined organic layer was washed with brine, dried over Na_2SO_4, filtered and concentrated *in vacuo*. The residue was purified by column chromatography on silica gel (*n*-hexane: ethyl acetate = 5:1) to afford the desired product $[D]_1$-**3a**. Deuterium contents were determined by ^{1}H-NMR spectroscopic analysis.

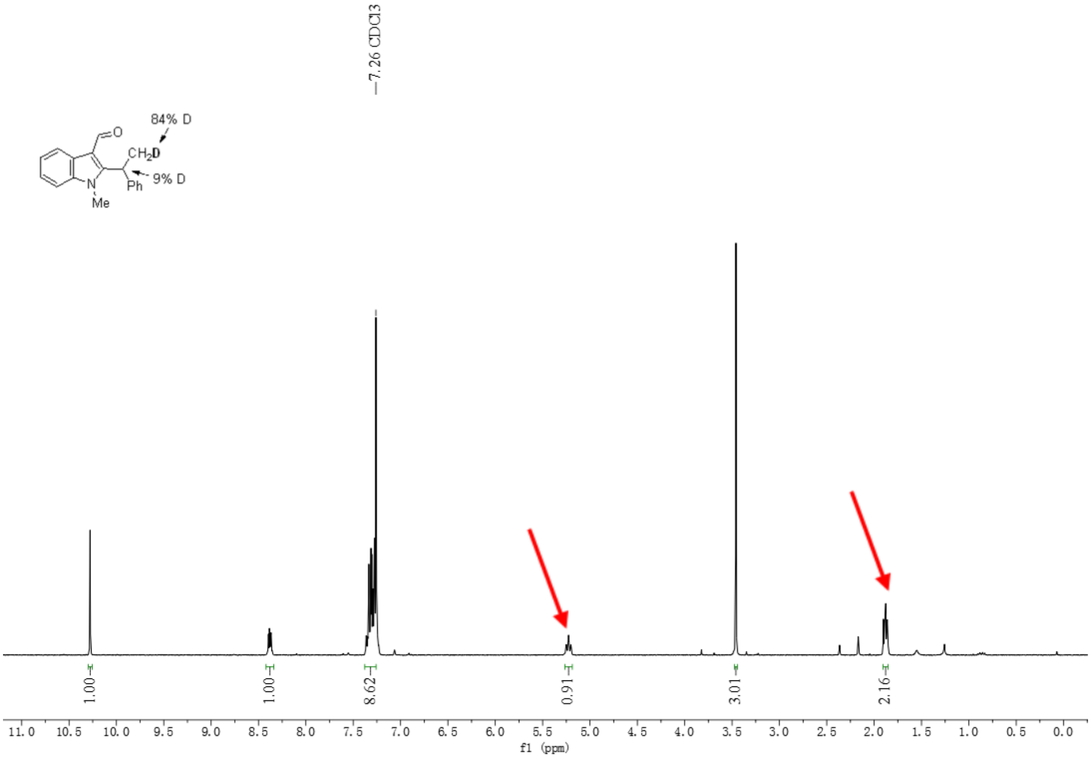

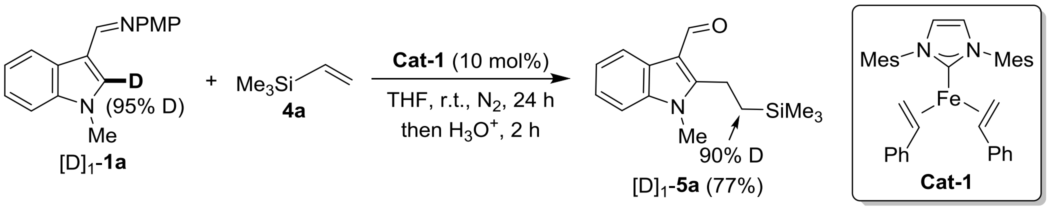

To a flame-dried and N_2-purged Schlenk tube were added indole substrate [D]1-**1a** (0.1 mmol, 26.5 mg) and **Cat-1** (10 mol%, 0.01 mmol, 5.7 mg) in the glove box. Trimethyl(vinyl)silane (**4a**) (0.15 mmol, 22 μL) and tetrahydrofuran (0.3 mL) were added *via* syringe under nitrogen atmosphere, the resulting mixture was stirred at 60 °C for 24 hours. Then, the reaction mixture was diluted with tetrahydrofuran (2.0 mL) and quenched with HCl aqueous solution (1 M, 1.0 mL). The resulting mixture was stirred at room temperature for 2 hours. The phases were then separated, the aqueous layer was extracted with ethyl acetate (5.0 mL ×3). The combined organic layer was washed with brine, dried over Na_2SO_4, filtered and concentrated *in vacuo*. The residue was purified by column chromatography on silica gel (*n*-hexane: ethyl acetate = 10:1) to afford the desired product [D]1-**5a**. Deuterium contents were determined by ^{1}H-NMR spectroscopic analysis.

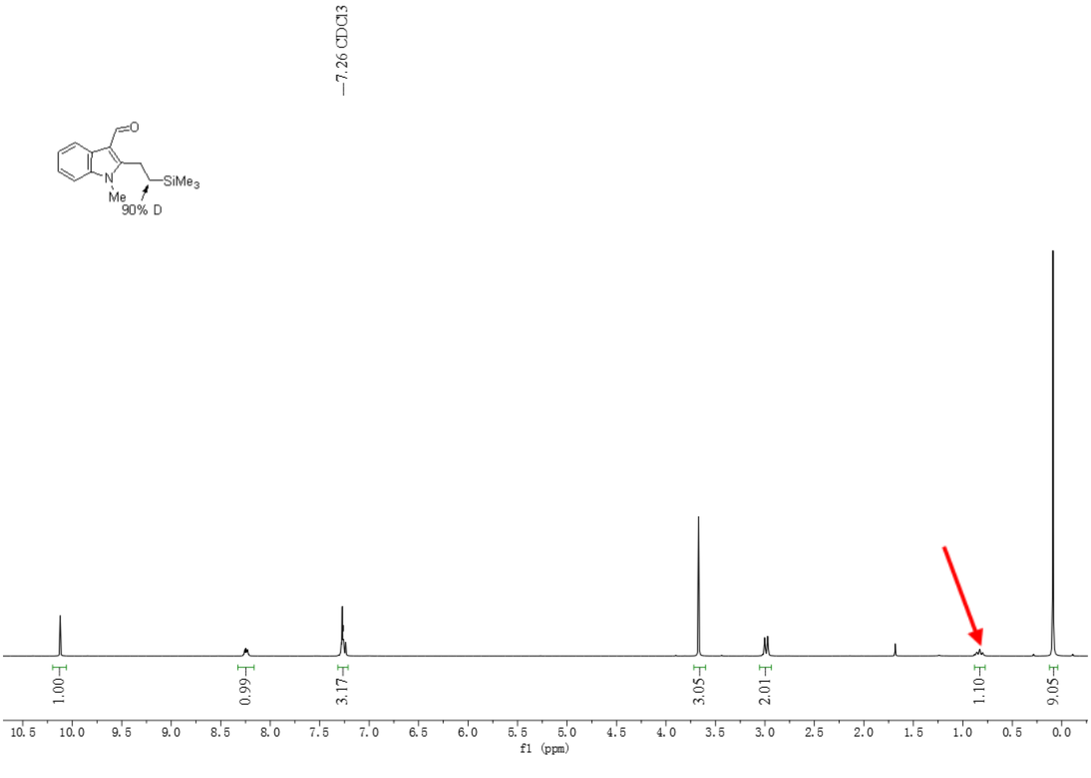

6.3 Deuterium crossover experiment

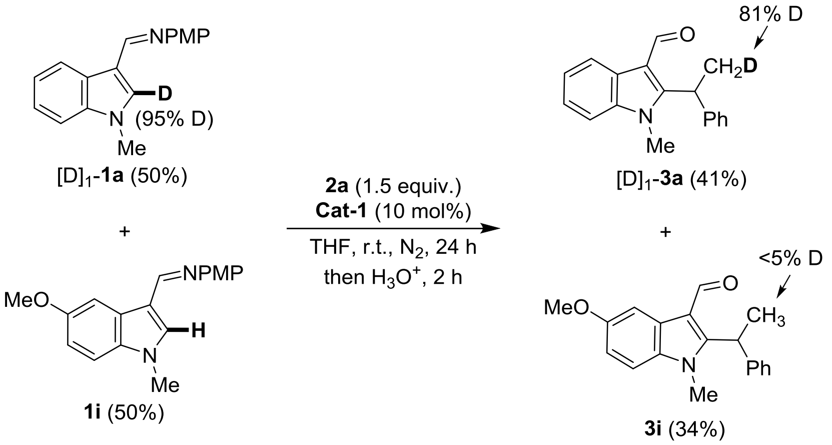

To a flame-dried and N_2-purged Schlenk tube were added indole substrate $[D]_1$-**1a** (0.1 mmol, 26.5 mg), **1i** (0.1 mmol, 29.4 mg) and **Cat-1** (10 mol%, 0.02 mmol, 11.4 mg) in the glove box. Styrene (**2a**) (0.3 mmol, 34 μL) and tetrahydrofuran (0.5 mL) were added *via* syringe under nitrogen atmosphere, the resulting mixture was stirred at room temperature for 24 hours. Then, the reaction mixture was diluted with tetrahydrofuran (3.0 mL) and quenched with HCl aqueous solution (1 M, 2.0 mL). The resulting mixture was stirred at room temperature for 2 hours. The phases were then separated, the aqueous layer was extracted with ethyl acetate (5.0 mL ×3). The combined organic layer was washed with brine, dried over Na_2SO_4, filtered and concentrated *in vacuo*. The residue was purified by column chromatography on silica gel (*n*-hexane: ethyl acetate = 5:1) to afford the desired product. Deuterium contents were determined by ^{1}H-NMR spectroscopic analysis.

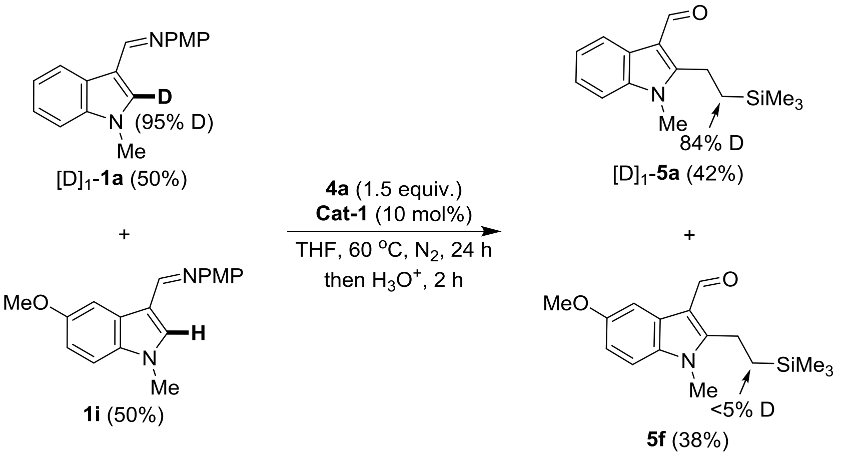

To a flame-dried and N_2-purged Schlenk tube were added indole substrate $[D]_1$-**1a** (0.1 mmol, 26.5 mg), **1i** (0.1 mmol, 29.4 mg) and **Cat-1** (10 mol%, 0.02 mmol, 11.4 mg) in the glove box. Trimethyl(vinyl)silane (**4a**) (0.3 mmol, 44 μL) and tetrahydrofuran (0.5 mL) were added *via* syringe under nitrogen atmosphere, the resulting mixture was stirred at 60 °C for 24 hours. Then, the reaction mixture was diluted with tetrahydrofuran (3.0 mL) and quenched with HCl aqueous solution (1 M, 2.0 mL). The resulting mixture was stirred at room temperature for 2 hours. The phases were then separated, the aqueous layer was extracted with ethyl acetate (5.0 mL ×3). The combined organic layer was washed with brine, dried over Na_2SO_4, filtered and concentrated *in vacuo*. The residue was purified by column chromatography on silica gel (*n*-hexane: ethyl acetate = 10:1) to afford the desired product. Deuterium contents were determined by ^{1}H-NMR spectroscopic analysis.

6.4 Stoichiometric reaction

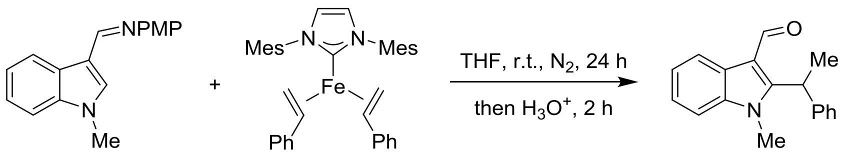

To a flame-dried and N_2-purged Schlenk tube were added indole substrate **1a** (0.1 mmol, 26.4 mg) and **Cat-1** (0.1 mmol, 56.8 mg) in the glove box. Tetrahydrofuran (0.5 mL) were added *via* syringe under nitrogen atmosphere, the resulting mixture was stirred at room temperature for 24 hours. Then, the reaction mixture was diluted with tetrahydrofuran (2.0 mL) and quenched with HCl aqueous solution (1 M, 1.0 mL). The resulting mixture was stirred at room temperature for 2 hours. The phases were then separated, the aqueous layer was extracted with ethyl acetate (5.0 mL ×3). The combined organic layer was washed with brine, dried over Na_2SO_4, filtered and concentrated *in vacuo*. The residue was purified by column chromatography on silica gel (*n*-hexane: ethyl acetate = 5:1) to afford the desired product **3a** (20.5 mg, 78% yield).

7. Computational studies

7.1 Computational details

All DFT calculations were carried out with the Gaussian 16 program.[7] The geometry optimizations were conducted using the B3LYP functional[8] including Grimme's dispersion corrections[9,10] with a Becke-Johnson damping function. Def2-SVP basis set[11] was used for all the atoms. To confirm whether each optimized stationary point is an energy minimum or a transition state as well as to evaluate the zero-point vibrational energy and thermal corrections at 298 K, vibrational frequencies were computed at the same level of theory as for the geometry optimizations. On the basis of the gas-phase optimized structures, the single-point energies and solvent effects were evaluated at the TPSSh level of theory[12] including Grimme's dispersion corrections with a Becke-Johnson damping using def2-TZVP basis set[13] for all the atoms. The solvation energies were calculated using the self-consistent reaction field with the SMD implicit solvent model[14].

The 3D diagrams of computed species were generated by CYLView[15].

7.2 DFT-computed free energy diagram of iron(0) complex-catalyzed C–H alkylation of indole 1a using alkene 2a including all possible spin states

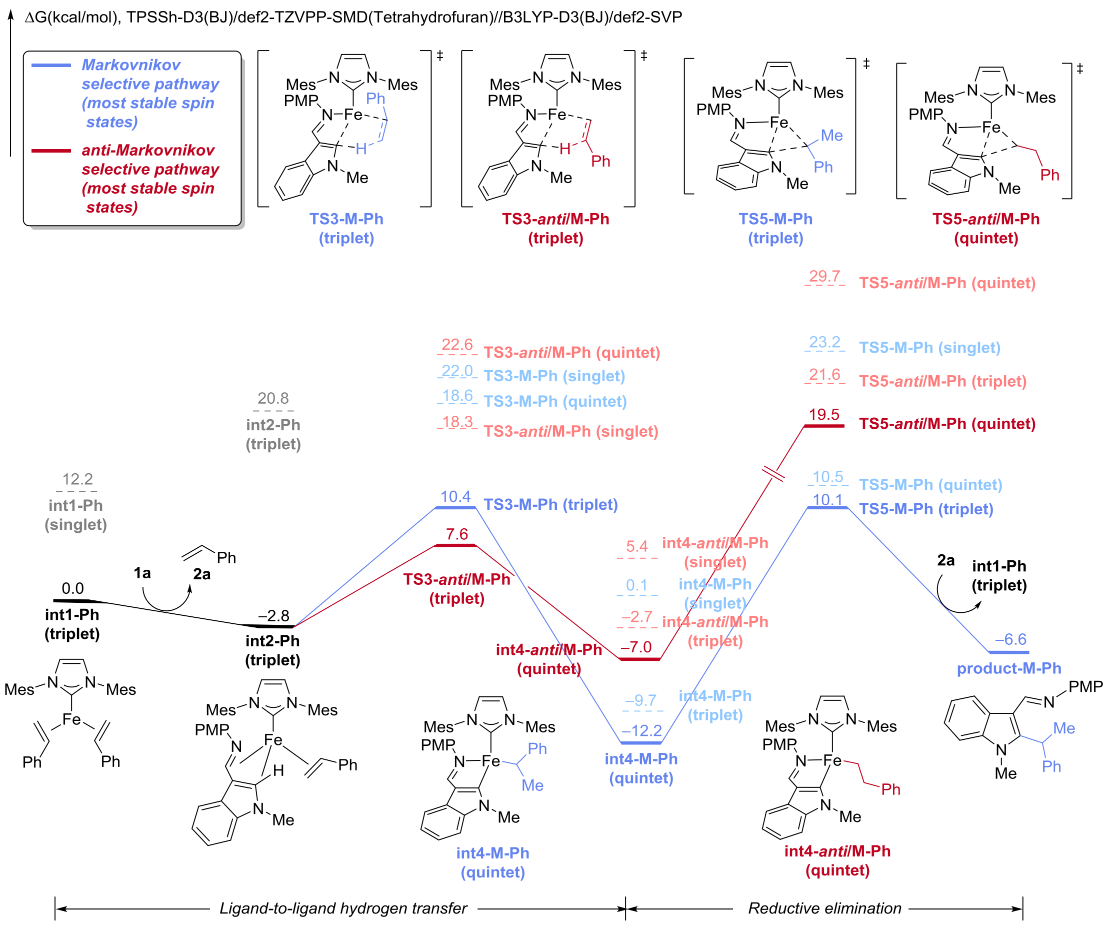

Figure S3. DFT-computed free energy profile of iron(0) compelx-catalyzed C–H alkylation using styrene leading to Markovnikov product. All possible spin states for each species are included with unfavorable spin states presented in dashed line.

7.3 DFT-computed free energy diagram of iron(0) complex-catalyzed C–H alkylation of indole 1a using vinyl silane 4a including all possible spin states

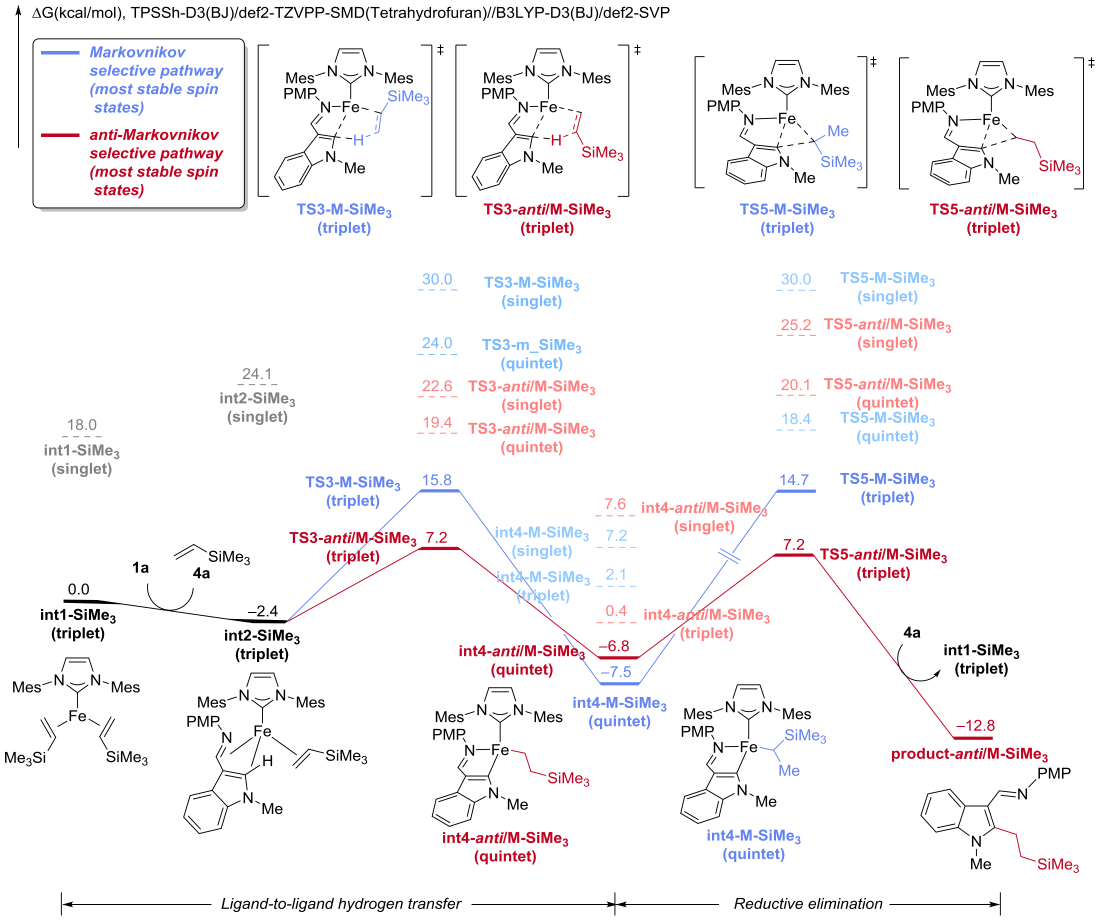

Figure S4. DFT-computed free energy profile of iron(0) compelx-catalyzed C–H alkylation using vinyl silane **4a** leading to *anti*-Markovnikov product. All possible spin states for each species are included with unfavorable spin states presented in dashed line.

7.4 Table of energy

Table S3. Thermal correction to Gibbs free energy (*TCG*), electronic energies (*E*) and Gibbs free energy (*G*) (in Hartree) of the structures calculated at the TPSSh-D3(BJ)/def2-TZVPP-SMD(Tetrahydrofuran)//B3LYP-D3(BJ)/def2-SVP level of theory.

Structures	*TCG*	*E*	*G*	Imaginary Frequency
**1a**	0.246495	–842.594850	–842.3484	
**2a**	0.102196	–309.811017	–309.7088	
**int1-Ph (singlet)**	0.596091	–2808.120944	–2807.5249	
**int1-Ph (triplet)**	0.594749	2808.139046	–2807.5443	
**int2-Ph (singlet)**	0.745132	–3340.89576	–3340.1506	
**int2-Ph (triplet)**	0.737514	–3340.925862	–3340.1883	
**TS3-M-Ph (singlet)**	0.740719	–3340.889494	–3340.1488	616.21*i*
**TS3-M-Ph (triplet)**	0.737683	–3340.905012	–3340.1673	1256.24*i*
**TS3-Ph (quintet)**	0.733407	–3340.887575	–3340.1542	1037.42*i*
**TS3-anti/M-Ph (singlet)**	0.742350	–3340.890114	–3340.1478	176.26*i*
**TS3-anti/M-Ph (triplet)**	0.736025	–3340.907822	–3340.1718	941.24*i*
**TS3-anti/M-Ph (quintet)**	0.731889	–3340.886593	–3340.1547	1084.26*i*
**int4-M-Ph (singlet)**	0.745073	–3340.928754	–3340.1837	
**int4-M-Ph (triplet)**	0.739995	–3340.939235	–3340.1992	
**int4-M-Ph (quintet)**	0.739470	–3340.942708	–3340.2032	
**int4-anti/M-Ph (singlet)**	0.741641	–3340.916742	–3340.1751	
**int4-anti/M-Ph (triplet)**	0.742535	–3340.930682	–3340.1881	

**int4-anti/M-Ph (quintet)**	0.738960	–3340.933923	–3340.1950	
**TS4-M-Ph (singlet)**	0.742249	–3340.889078	–3340.1468	377.36*i*
**TS4-M-Ph (triplet)**	0.736365	–3340.88575	–3340.1494	357.18*i*
**TS4-M-Ph (quintet)**	0.736785	–3340.889618	–3340.1528	379.59*i*
**TS4-anti/M-Ph (singlet)**	0.743246	–3340.879803	–3340.1366	425.00*i*
**TS4-anti/M-Ph (triplet)**	0.736365	–3340.88575	–3340.1494	357.18*i*
**TS4-anti/M-Ph (quintet)**	0.736785	–3340.889618	–3340.1528	379.59*i*
**Product-M-Ph**	0.373722	–1152.441468	–1152.0677	
**4a**	0.11839	–487.408367	–487.28998	
**TS3-M-SiMe$_3$ (singlet)**	0.75977	–3518.482951	–3517.7232	129.93*i*
**TS3-M-SiMe$_3$ (triplet)**	0.757588	–3518.503356	–3517.7458	1387.18*i*
**TS3-M-SiMe$_3$ (quintet)**	0.746413	–3518.479187	–3517.7328	1271.09*i*
**TS3-anti/M-SiMe$_3$ (singlet)**	0.756316	–3518.496462	–3517.7401	214.36*i*
**TS3-anti/M-SiMe$_3$ (triplet)**	0.751863	–3518.511443	–3517.7596	767.85*i*
**TS3-anti/M-SiMe$_3$ (quintet)**	0.746050	–3518.481095	–3517.7350	1372.79*i*
**int4-M-SiMe$_3$ (singlet)**	0.757995	–3518.517603	–3517.7596	
**int4-M-SiMe$_3$ (triplet)**	0.756996	–3518.524687	–3517.7677	
**int4-M-SiMe$_3$ (quintet)**	0.754963	–3518.537988	–3517.7830	
**int4-anti/M-SiMe$_3$ (singlet)**	0.757266	–3518.516185	–3517.7589	
**int4-anti/M-SiMe$_3$ (triplet)**	0.754316	–3518.52473	–3517.7704	

**int4-anti/M-SiMe$_3$ (quintet)**	0.750004	–3518.531907	–3517.7819	
**TS4-M-SiMe$_3$ (singlet)**	0.75977	–3518.482951	–3517.7232	129.93*i*
**TS4-M-SiMe$_3$ (triplet)**	0.754521	–3518.502067	–3517.7475	332.17*i*
**TS4-M-SiMe$_3$ (quintet)**	0.754131	–3518.495836	–3517.7417	388.33*i*
**TS4-anti/M-SiMe$_3$ (singlet)**	0.760424	–3518.491343	–3517.7309	323.49*i*
**TS4-anti/M-SiMe$_3$ (triplet)**	0.751863	–3518.511443	–3517.7596	767.85*i*
**TS4-anti/M-SiMe$_3$ (quintet)**	0.752268	–3518.491179	–3517.7389	308.30*i*
**product-anti/M-SiMe$_3$**	0.389467	–1330.048201	–1329.6587	

7.5 Cartesian coordinates of the computed species

1a

C 3.91431200 -0.12907900 0.04731900
C 2.72007500 0.63462300 -0.09090100
C 2.82219200 2.02414100 -0.27589800
C 4.08543200 2.60873900 -0.31703500
C 5.25518100 1.83290700 -0.17734500
C 5.18643400 0.45339700 0.00658000
C 2.18347700 -1.55141000 0.18879200
C 1.61731900 -0.29915900 0.00251400
H 1.92583100 2.63852600 -0.38699900
H 4.17558800 3.68796200 -0.46066400
H 6.23147900 2.32163400 -0.21431500
H 6.09268700 -0.14584000 0.11417700
H 1.67581400 -2.50679900 0.30238300
N 3.55117600 -1.45745700 0.21514200
C 0.20183700 -0.00742900 -0.08085800
H -0.06313300 1.05991700 -0.21286200
N -0.69953800 -0.91908400 -0.02069800
C -2.05738900 -0.58946900 -0.02994200
C -2.95575800 -1.49040400 -0.63998900
C -2.59150100 0.56450800 0.56910200
C -4.31644600 -1.22571400 -0.69041000
H -2.54692200 -2.39975700 -1.08473300
C -3.96343400 0.83207800 0.53773400
H -1.93006800 1.25237300 1.10014600
C -4.83625200 -0.05848900 -0.10316200
H -5.01187600 -1.91431000 -1.17411000
H -4.33882800 1.73010900 1.02820900
O -6.18209100 0.11022300 -0.19037600
C -6.75997600 1.26105800 0.37579300
H -6.36593300 2.18726300 -0.08128700
H -7.83937300 1.20011900 0.18230200
H -6.59495900 1.31071800 1.46779500
C 4.48032500 -2.55006900 0.38979500
H 5.15028800 -2.64039000 -0.48094000
H 5.09889300 -2.40108700 1.29000900
H 3.92080400 -3.48780800 0.50025900

2a

C 2.97527500 0.33741900 -0.00000300
C 1.95549600 -0.53295500 -0.00000100
H 4.01033500 -0.01187800 0.00001200
H 2.82446300 1.42030600 0.00000200
H 2.19113000 -1.60349000 0.00010400
C 0.51602000 -0.22488900 -0.00005900
C 0.01338100 1.09116600 0.00001500
C -0.40906400 -1.28472500 -0.00000100
C -1.35841400 1.33259600 0.00001800
H 0.70490900 1.93599800 0.00004600
C -1.78442400 -1.04496500 0.00001200
H -0.03754400 -2.31307000 0.00001500
C -2.26550500 0.26592900 -0.00001700
H -1.72649800 2.36157400 0.00002400
H -2.48248700 -1.88566400 0.00007500
H -3.34090100 0.45876200 -0.00006100

int1-Ph (singlet)

Fe -0.20612300 0.35735000 -0.14598600
C 1.34934700 0.99547100 -1.32736600
H 2.11605200 0.22287700 -1.30244900
C 0.67538300 1.00733700 1.53810900
C -0.67403000 0.66300900 1.84236100
H 1.48376900 0.41521300 1.96993200
H 0.95075500 2.06186200 1.42319300
H -0.89859300 -0.17452000 2.50873400
C 1.89790200 2.31830900 -0.99292100
C 1.28308800 3.53116000 -1.36035100
C 3.08690600 2.39276300 -0.23556800
C 1.81997600 4.76044500 -0.97087200
H 0.37782100 3.51162200 -1.97069600
C 3.62579300 3.61840500 0.14817900
H 3.57424400 1.46096400 0.06302300
C 2.99272900 4.81417000 -0.21283100
H 1.32068300 5.68609200 -1.26976400
H 4.54575800 3.64496400 0.73874600
H 3.41200600 5.77643200 0.09042500
C -1.78888900 1.54937800 1.52368400
C -3.08694200 1.35149900 2.05116900
C -1.63333600 2.57386900 0.55108900
C -4.16188600 2.12798100 1.63413600
H -3.23374600 0.57754300 2.80738400
C -2.72468900 3.33018200 0.11501100
H -0.63972300 2.81284600 0.16189400
C -3.99495100 3.11707600 0.65285200
H -5.14896900 1.96087200 2.07385100

H -2.56997800 4.10570900 -0.63977900
H -4.84494000 3.72007600 0.32513700
C 0.16904400 0.74529900 -2.08530100
H -0.41396900 1.58816400 -2.48073700
H 0.15916100 -0.12402600 -2.74859400
N 1.13926300 -2.36669900 -0.13463300
C 0.07200400 -1.50212700 -0.19286900
C 0.73860700 -3.69303200 -0.31263300
C -0.60675100 -3.67673300 -0.48945300
H 1.45183400 -4.51138800 -0.29396900
H -1.32000400 -4.47717900 -0.66115500
C -2.33697500 -1.84383000 -0.45374000
C -2.87182700 -1.34357000 -1.65409300
C -3.08356300 -1.85628400 0.74111200
C -4.15244600 -0.77481300 -1.61142200
C -4.36237700 -1.29797400 0.72436600
C -4.90417700 -0.72743600 -0.43540500
H -4.57393800 -0.36522600 -2.53381600
H -4.94666100 -1.28811500 1.64768500
C -2.52666300 -2.47906600 1.99387500
H -2.59268800 -3.57960900 1.95231900
H -1.46719500 -2.22814800 2.13376500
H -3.08342700 -2.14488800 2.88038600
C -2.16448000 -1.47492700 -2.97694300
H -1.16648900 -1.91455800 -2.87242400
H -2.75369600 -2.11829100 -3.65135900
H -2.05510100 -0.49709600 -3.46731600
C -6.24554500 -0.04534800 -0.40702500
H -6.79207200 -0.18313200 -1.35237200
H -6.87408600 -0.41759400 0.41554600
H -6.11042000 1.03921700 -0.25726800
N -0.99594300 -2.34372000 -0.41841900
C 2.48993900 -1.97322200 0.12814500
C 2.89107200 -1.77840300 1.45856500
C 3.37245000 -1.79406900 -0.95044900
C 4.19019000 -1.31059900 1.68956900
C 4.66387900 -1.33540700 -0.67044800
C 5.08269900 -1.06405400 0.63893700
H 4.51115500 -1.13217300 2.71930200
H 5.35556000 -1.16873300 -1.50057900
C 1.95946100 -2.10090700 2.59773700
H 0.97120300 -1.64440600 2.45358100
H 1.80224200 -3.18995300 2.67590500
H 2.36720000 -1.74727700 3.55452200
C 2.93310800 -2.08229000 -2.36251200
H 2.74774000 -3.15876800 -2.51176400
H 1.99577800 -1.56066700 -2.60604400
H 3.69848900 -1.76589700 -3.08417000
C 6.45289300 -0.49399200 0.90214700
H 7.20734500 -0.92642400 0.22720600
H 6.45702400 0.59733900 0.73667300
H 6.77417100 -0.67149300 1.93899700

int1-Ph (triplet)

Fe -0.00006200 -1.06637600 0.00086600
C -1.22949900 -1.39557400 -1.71168200
H -0.91730000 -0.76462800 -2.54845700
C 0.41440400 -2.50457900 1.35541600
C 1.22922700 -1.39241200 1.71407400
H -0.52084900 -2.67459700 1.90428600
H 0.87776000 -3.42105200 0.97827400
H 0.91697100 -0.75995600 2.54968300
C -2.69035500 -1.43777300 -1.50362400
C -3.27323500 -2.04954200 -0.37431300
C -3.56133100 -0.93130900 -2.48790200
C -4.65585900 -2.17122200 -0.25249900
H -2.62689600 -2.42956900 0.41892800
C -4.94598700 -1.04960900 -2.36567200
H -3.13519900 -0.45874200 -3.37663500
C -5.50425500 -1.67888900 -1.24951300
H -5.07700900 -2.64946600 0.63562000
H -5.59418400 -0.65828800 -3.15466800
H -6.58810800 -1.78217300 -1.15648400
C 2.69011100 -1.43500700 1.50624800
C 3.56097600 -0.92671200 2.48968000
C 3.27313100 -2.04886800 0.37814500
C 4.94564700 -1.04522300 2.36783000
H 3.13474200 -0.45250600 3.37749200
C 4.65577100 -2.17075600 0.25671200
H 2.62689800 -2.43039400 -0.41446000
C 5.50405100 -1.67656500 1.25290600
H 5.59374900 -0.65243300 3.15617500
H 5.07702700 -2.65064000 -0.63047100
H 6.58791600 -1.78000100 1.16018800
C -0.41463800 -2.50705300 -1.35103900
H -0.87794500 -3.42282100 -0.97213300
H 0.52058100 -2.67806700 -1.89965500
N -1.01442600 1.81366400 0.35129600

C 0.00007700 0.96615200 -0.00100800
C -0.63923400 3.14955100 0.22152400
C 0.63947100 3.14914700 -0.22720700
H -1.31832300 3.96137500 0.46180500
H 1.31858300 3.96054100 -0.46886800
C 2.33963900 1.41478900 -0.73049700
C 2.58517800 0.89137200 -2.00732000
C 3.37625500 1.61559200 0.20275400
C 3.90685000 0.55192000 -2.33186800
C 4.67489500 1.27719900 -0.17400900
C 4.96092700 0.73946200 -1.43639500
H 4.11292800 0.13693100 -3.32248200
H 5.48422800 1.41203400 0.54655100
C 3.08745800 2.18047400 1.56847300
H 2.87401900 3.26141900 1.52727100
H 2.20884800 1.69467900 2.01690500
H 3.94326700 2.02828400 2.23818800
C 1.49019200 0.68248400 -3.01929000
H 0.61295500 1.31084400 -2.81762900
H 1.85391100 0.90123700 -4.03425700
H 1.14764000 -0.36414600 -3.01198700
C 6.37318700 0.36022600 -1.79486700
H 6.44720200 -0.00281800 -2.83015200
H 7.05848000 1.21614800 -1.68266400
H 6.73874000 -0.43584200 -1.12608400
N 1.01461700 1.81303000 -0.35471700
C -2.33944500 1.41612600 0.72782800
C -2.58499800 0.89516800 2.00564800
C -3.37607100 1.61523400 -0.20577900
C -3.90669300 0.55643800 2.33087000
C -4.67472900 1.27766400 0.17164900
C -4.96077700 0.74235700 1.43507000
H -4.11278200 0.14338300 3.32229000
H -5.48407200 1.41121900 -0.54913900
C -1.49004800 0.68821800 3.01806100
H -1.14787400 -0.35855100 3.01319200
H -0.61260200 1.31581300 2.81494000
H -1.85367000 0.90944700 4.03252700
C -3.08723600 2.17758500 -1.57253000
H -2.87373600 3.25859300 -1.53330000
H -2.20863500 1.69092800 -2.02004300
H -3.94304600 2.02422400 -2.24197500
C -6.37304800 0.36387200 1.79428500
H -7.05850000 1.21930500 1.67940500
H -6.73824000 -0.43421600 1.12771200
H -6.44725300 0.00380100 2.83059400

int2-Ph (singlet)
N -0.87998100 -0.74036200 2.08739500
C -2.06832700 0.05890300 1.96170700
C -2.05592000 1.41327400 2.31380100
C -3.25759200 -0.60020200 1.59182300
C -3.26471100 2.12106900 2.22790900
C -4.45927000 1.50943400 1.84225900
H -3.26507900 3.18490000 2.48362200
C -0.03357900 -1.15023300 1.07816100
Fe -0.03323400 -0.64602100 -0.81429800
C 2.35208900 -0.85055600 -1.39959100
H 2.71180700 -1.63937600 -0.74726400
C 3.24142500 0.30330900 -1.50852500
C 2.95190300 1.42699700 -2.31012800
C 4.45882400 0.30691100 -0.79575200
C 3.83206300 2.50330000 -2.38095100
H 2.00471300 1.47125200 -2.84985900
C 5.34052500 1.38439000 -0.86892200
H 4.70733600 -0.55882000 -0.17746100
C 5.03228800 2.49362300 -1.66086800
H 3.57062800 3.36978100 -2.99326600
H 6.27394700 1.36115000 -0.30013300
H 5.71262600 3.34686100 -1.70728100
C 0.40892000 -2.24157100 3.06179000
H 0.92224200 -2.94460700 3.71009700
C -0.61945800 -1.39196000 3.28869100
H -1.20442000 -1.18954100 4.18038300
C -4.43618900 0.14315400 1.53194800
H -5.35654600 -0.34981400 1.21023100
C 1.31104700 -1.15449700 -2.27733000
H 1.18857400 -0.61410900 -3.22057900
H 0.98139500 -2.20743500 -2.32055800
C -3.26624600 -2.07943900 1.31394100
H -2.41976000 -2.36886400 0.67996600
H -3.18538200 -2.65658500 2.25128200
H -4.19704000 -2.37446800 0.81090900
C -0.81804800 2.12023200 2.79618700
H 0.05287400 1.45782200 2.83442800
H -0.55819100 2.95783000 2.13238400
H -0.98259500 2.53636200 3.80372500
C -5.75129800 2.28327800 1.79129300

H -6.30962000 2.18005500 2.73816700
H -5.57248300 3.35723900 1.63113500
H -6.39881500 1.91613700 0.98225200
N 0.75870700 -2.08402300 1.72330600
C -4.13173300 -0.77327800 -1.86983000
C -3.89694000 0.60061800 -1.55440200
C -4.97812800 1.48716300 -1.53620100
C -6.26555300 1.00809400 -1.80696300
C -6.48269200 -0.34835600 -2.09170300
C -5.41553600 -1.25771200 -2.12607500
C -1.88377000 -0.59305000 -1.44027200
C -2.48802000 0.70997200 -1.27610100
H -4.82019900 2.54065200 -1.29491100
H -7.11313600 1.69776500 -1.79176500
H -7.49606400 -0.70431800 -2.29252900
H -5.59035700 -2.31316900 -2.34485600
H -0.97594300 -0.56419100 -2.23060600
N -2.92245300 -1.45748500 -1.83405400
C -2.75566100 -2.83935200 -2.18684000
H -3.00431100 -3.52701700 -1.35703300
H -3.39203900 -3.09779100 -3.04829100
H -1.70913800 -3.01632300 -2.47315400
C -1.60391100 1.68490600 -0.83967300
H -1.87985500 2.73143200 -0.68216200
N -0.35924100 1.26234100 -0.57029000
C 0.63798500 2.19484600 -0.23746200
C 1.67660900 1.82498000 0.63834200
C 0.66057200 3.49728800 -0.75959200
C 2.68944600 2.70758500 0.97071700
H 1.67466300 0.81158300 1.03225000
C 1.67861900 4.39770400 -0.42962700
H -0.10285100 3.80449100 -1.47614600
C 2.71058300 4.00228300 0.43007600
H 3.50706300 2.40722500 1.62715700
H 1.67181900 5.38955500 -0.88112600
O 3.77335900 4.78053800 0.77280300
C 3.90225500 6.04437200 0.17262300
H 3.06044300 6.71401400 0.42915200
H 4.83315900 6.48420300 0.55573800
H 3.96740300 5.96983700 -0.92855800
C 1.80721500 -2.84928600 1.12797000
C 3.14090400 -2.56862100 1.47324500
C 1.47427600 -3.86813600 0.22171700
C 4.14768200 -3.32651400 0.86623100
C 2.51682600 -4.58531100 -0.37605100
C 3.85874400 -4.32695600 -0.07145200
H 5.19048300 -3.10928400 1.11267500
H 2.27219100 -5.37032400 -1.09656400
C 3.47998200 -1.43067700 2.39951900
H 3.06410200 -0.48602300 2.01669100
H 3.06777100 -1.57529000 3.40984900
H 4.56792900 -1.31004800 2.48921800
C 0.03401600 -4.16795700 -0.09782000
H -0.53128500 -4.42658400 0.81183900
H -0.47570300 -3.29287700 -0.52888500
H -0.05082800 -4.99991300 -0.81014500
C 4.96943900 -5.07543300 -0.76187200
H 4.62331100 -6.04271400 -1.15486300
H 5.35842200 -4.49215700 -1.61411300
H 5.81476800 -5.26107400 -0.08188600

int2-Ph (triplet)

N -0.61201000 -1.98061800 1.78689600
C -1.96491500 -1.49543300 1.76995900
C -2.27140700 -0.28949600 2.41857800
C -2.94741000 -2.28542700 1.15320200
C -3.60617200 0.12683000 2.41291800
C -4.61836000 -0.62577500 1.80458600
H -3.86167100 1.07412900 2.89481800
C 0.33566300 -1.72863200 0.83235500
Fe 0.18216700 -0.38070000 -0.74655300
C 1.65707500 -0.52124000 -2.28499000
H 2.15241300 -1.49392700 -2.23283300
C 2.55452900 0.62839300 -2.10192500
C 2.15598200 1.96089500 -2.33663900
C 3.86847800 0.40912500 -1.64250600
C 3.02886300 3.02012600 -2.10315000
H 1.14103200 2.17187700 -2.67523000
C 4.74046000 1.47052200 -1.40690200
H 4.19511400 -0.61342300 -1.45813800
C 4.32444400 2.78594800 -1.63069300
H 2.68539100 4.04354800 -2.26874300
H 5.75002600 1.26923800 -1.03805300
H 4.99545500 3.62232800 -1.42305300
C 1.14772600 -3.11868300 2.46091400
H 1.88981200 -3.74194600 2.95006700
C -0.13676200 -2.82200400 2.78438600
H -0.75892500 -3.12634900 3.62060300

C -4.26933700 -1.83307800 1.18645900
H -5.04746000 -2.43170000 0.70611400
C 0.35074700 -0.45666700 -2.81323000
H -0.05009500 0.46878000 -3.23581500
H -0.10017100 -1.37363400 -3.20817000
C -2.57268100 -3.56616400 0.45446200
H -1.85545900 -3.37574100 -0.36091100
H -2.08397500 -4.27908200 1.13822800
H -3.45790300 -4.05638400 0.02707100
C -1.19557000 0.51473900 3.09404400
H -0.74519600 -0.03958300 3.93449700
H -0.38993200 0.75475600 2.38752000
H -1.59674100 1.46003100 3.48336900
C -6.04030200 -0.13194100 1.79800900
H -6.74517300 -0.93348600 1.53385600
H -6.32750400 0.27164500 2.78175100
H -6.16547200 0.67640500 1.06049100
N 1.41748400 -2.44566200 1.27297600
C -4.81310400 0.24754000 -1.62368700
C -4.17607700 1.34775200 -0.97667300
C -4.93699400 2.48050900 -0.66090900
C -6.29686400 2.50290900 -0.98088800
C -6.90773300 1.40562000 -1.61868800
C -6.17504200 0.26450100 -1.94954000
C -2.64846400 -0.30202500 -1.31134300
C -2.77855600 0.98737200 -0.78051500
H -4.47335100 3.33689300 -0.16545000
H -6.89659800 3.38217500 -0.73431800
H -7.97334800 1.44649700 -1.85706100
H -6.65216600 -0.58559300 -2.44153900
H -1.81513100 -1.00496500 -1.29249200
N -3.86896000 -0.73690200 -1.82261000
C -4.11947900 -2.00913800 -2.44394100
H -4.83581300 -2.61357100 -1.86004500
H -4.52670900 -1.88461600 -3.46219600
H -3.17717100 -2.56745400 -2.51696700
C -1.77558000 1.78566900 -0.18088100
H -2.06767600 2.74873800 0.24902200
N -0.49432000 1.37251600 -0.08178700
C 0.48066100 2.26569700 0.38258700
C 1.68466700 1.73959500 0.90484300
C 0.37095000 3.66601800 0.32296600
C 2.72146200 2.55449200 1.32301500
H 1.80198500 0.65594700 0.95481500
C 1.41228500 4.49994200 0.75043100
H -0.52010600 4.12076200 -0.11105200
C 2.59931300 3.95175800 1.24689900
H 3.65540400 2.13188100 1.69558100
H 1.28746600 5.57957200 0.66373100
O 3.68341500 4.67547800 1.65323200
C 3.62389600 6.07542700 1.56450600
H 2.81222300 6.49660200 2.18689400
H 4.58627400 6.45917000 1.93036700
H 3.47582500 6.41783400 0.52312900
C 2.67281000 -2.56773300 0.59379700
C 3.80627300 -1.93653900 1.12998100
C 2.74339600 -3.38184000 -0.55106800
C 5.03330400 -2.13621500 0.48426500
C 3.98798800 -3.53770300 -1.16829400
C 5.14450400 -2.92357300 -0.66645000
H 5.92282700 -1.64160800 0.88339200
H 4.05736200 -4.15895400 -2.06520400
C 3.72111600 -1.08350600 2.36894900
H 2.78231100 -0.51560400 2.41169600
H 3.76651000 -1.69828500 3.28390800
H 4.55623800 -0.37043400 2.40719000
C 1.51140300 -4.05885500 -1.09052600
H 1.00651500 -4.65141000 -0.31093600
H 0.77752200 -3.31614100 -1.44177200
H 1.76170000 -4.72502300 -1.92732000
C 6.46259200 -3.06177800 -1.38315600
H 6.54327200 -4.02959400 -1.90031200
H 6.57122600 -2.27202700 -2.14675800
H 7.31214900 -2.96683900 -0.69067000

TS3-M-Ph (singlet)

N -0.70540400 -0.69264800 2.11339800
C -1.96297500 -0.00213000 2.01383000
C -2.05196600 1.36476000 2.29610000
C -3.10801600 -0.77737300 1.73499400
C -3.31923700 1.96640500 2.21976700
C -4.46948300 1.24163800 1.90742200
H -3.39998900 3.03827500 2.42310700
C 0.10829000 -1.10064300 1.07800300
Fe -0.06371900 -0.72371000 -0.88046200
C 2.04138100 -0.83114100 -1.59590800
H 2.47653600 -1.72316200 -1.14809800
C 0.75964000 -2.01766600 3.09036000

H 1.36699500 -2.63422800 3.74502600
C -0.31807900 -1.23673900 3.33377500
H -0.85993000 -1.01968200 4.24895800
C -4.34364000 -0.13604300 1.67479400
H -5.23245000 -0.72105900 1.42592400
C 1.02323600 -1.03809500 -2.56239900
H 0.78155700 -2.08226400 -2.82023300
C -3.00335100 -2.26838000 1.55798400
H -2.20412900 -2.53043400 0.85423300
H -2.76940500 -2.76336300 2.51603300
H -3.94929500 -2.68313200 1.18399000
C -0.86433800 2.19504800 2.70318800
H -1.02933600 2.63145000 3.70214200
H 0.06355500 1.61491900 2.72877700
H -0.70407200 3.02840200 2.00328900
C -5.82261100 1.90038600 1.84439700
H -6.45726200 1.57966000 2.68809400
H -5.74141000 2.99659600 1.88392800
H -6.34575500 1.62554200 0.91629300
N 1.00993500 -1.92501800 1.72361700
C -4.20391500 -0.88451300 -1.74905900
C -3.95935100 0.49615000 -1.48433500
C -5.02816800 1.39693700 -1.56072400
C -6.30479800 0.91906400 -1.87683000
C -6.52847700 -0.44604200 -2.11785000
C -5.47415000 -1.36780300 -2.05840800
C -1.97739400 -0.72147800 -1.22869700
C -2.55602300 0.58525900 -1.17077200
H -4.86994200 2.45975200 -1.36332000
H -7.14266100 1.61841300 -1.93494400
H -7.53494000 -0.79571100 -2.35950100
H -5.64843300 -2.42872200 -2.24965700
H -0.80778400 -0.65565000 -2.21134700
N -2.99726200 -1.57867800 -1.61317900
C -2.86581600 -2.98610300 -1.88879600
H -3.41308700 -3.60478400 -1.15638400
H -3.24896300 -3.22604500 -2.89478300
H -1.80521900 -3.25992900 -1.84998500
C -1.65082800 1.59577300 -0.83450000
H -1.95249500 2.63888500 -0.69415300
N -0.39100800 1.22746500 -0.64325100
C 0.56900300 2.19947600 -0.29943700
C 1.64251100 1.85548600 0.54293500
C 0.51116800 3.51733100 -0.77430800
C 2.60259500 2.78371600 0.90372000
H 1.71223100 0.82635600 0.88394200
C 1.47784900 4.46440700 -0.41866600
H -0.27582700 3.80798500 -1.47212000
C 2.53787800 4.09985900 0.41951800
H 3.44751000 2.50457600 1.53451200
H 1.40716100 5.47029600 -0.83188900
O 3.55069100 4.92886300 0.79166200
C 3.58633000 6.22881300 0.25909000
H 2.70190800 6.82291100 0.55536200
H 4.48762400 6.71166200 0.66064600
H 3.64697500 6.21816500 -0.84479000
H 0.93632300 -0.37064400 -3.42553300
C 2.97595400 0.30194000 -1.65463100
C 2.71057300 1.48052600 -2.38250600
C 4.21222000 0.22821500 -0.98178900
C 3.61835000 2.53607000 -2.40731500
H 1.76023900 1.58429000 -2.90793800
C 5.12167000 1.28572500 -1.00336200
H 4.45698000 -0.68540500 -0.43922700
C 4.82951100 2.45398300 -1.71135100
H 3.36961800 3.44297600 -2.96416200
H 6.06825500 1.19594900 -0.46282200
H 5.53143000 3.29091100 -1.71765000
C 2.06822800 -2.66711200 1.11337900
C 3.39945000 -2.32633700 1.40090600
C 1.74581600 -3.73740900 0.26222500
C 4.41339300 -3.08002100 0.79691800
C 2.79179400 -4.45053800 -0.33024900
C 4.13356300 -4.13485500 -0.07917400
H 5.45472000 -2.81631900 1.00040900
H 2.55213500 -5.27535700 -1.00669500
C 0.31016600 -4.08275300 -0.02359500
H -0.25437600 -4.26921100 0.90355100
H -0.21209600 -3.25064300 -0.52392800
H 0.23289500 -4.96929200 -0.66752500
C 3.73499100 -1.15687400 2.28971800
H 3.14589200 -0.27106000 2.01310500
H 3.52225000 -1.36818000 3.34988200
H 4.79871300 -0.89684300 2.20594300
C 5.24591200 -4.88299200 -0.76743600
H 4.95287600 -5.91698200 -1.00336000
H 5.51317200 -4.39172800 -1.71891000
H 6.15547900 -4.91398000 -0.14911100

TS3-M-Ph (triplet)

N -0.35661700 -0.13693300 2.28117500
C -1.77034000 -0.22114000 2.05679200
C -2.50186500 0.97374000 1.95083500
C -2.38453500 -1.48040500 2.01767100
C -3.87981000 0.87798200 1.74077300
C -4.52616300 -0.36379200 1.65058200
H -4.46020800 1.79814700 1.63705600
C 0.61519800 -0.02755000 1.31152300
Fe 0.36886300 -0.22517800 -0.65394400
C 2.01513400 0.57237900 -1.77025200
C 1.51567000 0.04546800 3.42043600
H 2.31647000 0.13020700 4.14828200
C 0.17499600 -0.09472400 3.56282000
H -0.45455700 -0.16392600 4.44462600
C -3.76904800 -1.52739800 1.81419100
H -4.25745200 -2.50191400 1.74666800
C 0.96654700 0.11821900 -2.64232600
H 1.20937200 -0.68481600 -3.34913600
C -1.58555300 -2.74150200 2.20736800
H -0.61640800 -2.67498300 1.69686500
H -1.38266200 -2.92370500 3.27686700
H -2.12808100 -3.61022900 1.81096300
C -1.81765500 2.30784400 2.07889100
H -1.50791500 2.49334900 3.12172700
H -0.91349500 2.35204000 1.45833800
H -2.48114700 3.12389600 1.76431600
C -6.00015600 -0.43891400 1.34369600
H -6.18018300 -0.31277600 0.26130600
H -6.42537400 -1.41090600 1.63323300
H -6.56378300 0.35430100 1.85904400
N 1.76426500 0.09062100 2.05078200
C -1.94028500 -3.81679900 -1.07097000
C -2.91534000 -2.78163300 -1.17090100
C -4.27198700 -3.13056800 -1.23498700
C -4.62967200 -4.48090700 -1.20537300
C -3.65287500 -5.48800300 -1.11142400
C -2.29213500 -5.16730000 -1.04409100
C -0.79630200 -1.84008500 -0.96400900
C -2.18147200 -1.54245800 -1.12544800
H -5.04058900 -2.35668500 -1.29827300
H -5.68468500 -4.76116900 -1.25558000
H -3.95867200 -6.53666000 -1.09165300
H -1.53611200 -5.95180600 -0.97268400
H -0.03220100 -1.05510600 -1.94009000
N -0.68433500 -3.21776000 -0.96883200
C 0.53945300 -3.95210500 -0.76406000
H 0.39762500 -4.73497600 -0.00133300
H 0.89684700 -4.43166100 -1.69161800
H 1.30811000 -3.25607600 -0.40699900
C -2.59220100 -0.19323100 -1.22848000
H -3.64909800 0.02865800 -1.43523000
N -1.70102700 0.75609300 -1.09331700
C -2.01664100 2.11196800 -1.17616400
C -0.94516200 3.03041400 -1.23458000
C -3.31892700 2.64307400 -1.14925100
C -1.15983200 4.40017500 -1.26794100
H 0.07166900 2.63675400 -1.23940300
C -3.54626600 4.02202000 -1.17717900
H -4.17980900 1.97787100 -1.08112700
C -2.46673400 4.91527500 -1.23700300
H -0.32560400 5.10280300 -1.31226200
H -4.57303200 4.38668200 -1.14573800
O -2.58461300 6.27009100 -1.26422900
C -3.87059800 6.83833800 -1.23845100
H -4.41979700 6.57482500 -0.31556800
H -3.73580500 7.92784400 -1.27228300
H -4.47727000 6.52589200 -2.10824100
H 0.27363400 0.85617700 -3.05984300
C 3.08636700 0.36809200 1.57148400
C 3.44552600 1.70686300 1.34583600
C 4.01111700 -0.67985900 1.47592600
C 4.78339000 1.98065200 1.04990000
C 5.34046800 -0.35668500 1.18359500
C 5.74670800 0.96522200 0.98279900
H 5.08169800 3.01654400 0.86537300
H 6.07059800 -1.16315600 1.08755000
C 3.58063500 -2.10548800 1.69574600
H 3.42327800 -2.32167400 2.76622900
H 2.62825800 -2.31111400 1.18629300
H 4.33646200 -2.80436500 1.31267000
C 2.41110700 2.79820000 1.41821000
H 1.62745000 2.64090200 0.66085600
H 1.90233400 2.81173500 2.39563200
H 2.86358300 3.78549900 1.25174700
C 7.18628400 1.29499000 0.68631900
H 7.26663600 2.08918700 -0.07180000
H 7.70516600 1.65526000 1.59157800

H 7.73083300 0.41439900 0.31674800
H 2.07284500 1.65222400 -1.59097500
C 3.32701500 -0.10643400 -1.81740100
C 4.51558100 0.62579000 -1.99136100
C 3.43205000 -1.51177400 -1.79291100
C 5.74547900 -0.01335200 -2.14937800
H 4.46395900 1.71658300 -2.02171000
C 4.66117000 -2.15391200 -1.93507300
H 2.52544100 -2.10299100 -1.65896300
C 5.83038200 -1.40765200 -2.11684000
H 6.64827400 0.58422500 -2.29839900
H 4.70703400 -3.24617500 -1.90755900
H 6.79448800 -1.90760400 -2.23700700

TS3-M-Ph (quintet)

N -0.41369100 -1.08969200 2.33746400
C -1.75444500 -0.58099800 2.22854200
C -2.00657300 0.76411000 2.53041200
C -2.77299400 -1.46638900 1.83601000
C -3.32932400 1.21336600 2.43179600
C -4.37543700 0.36376900 2.05559100
H -3.54434100 2.26225000 2.65302800
C 0.43233700 -1.26197900 1.28345300
Fe -0.09416000 -0.71660900 -0.68697800
C 1.15272900 -1.14703600 -2.51190800
H 1.55576700 -2.14188700 -2.29091900
C 1.38437700 -2.00662600 3.21734200
H 2.16087100 -2.45121400 3.83192300
C 0.14842600 -1.53520300 3.52737200
H -0.38598100 -1.48024700 4.47093300
C -4.07519700 -0.97087600 1.75427100
H -4.87716000 -1.63830300 1.43066500
C -0.05896500 -1.07062200 -3.25429700
H -0.51169200 -2.00981200 -3.58944800
C -2.45839400 -2.89824300 1.49871900
H -1.81001400 -2.95048400 0.61064000
H -1.92354900 -3.40347100 2.31919300
H -3.37620700 -3.46258000 1.28568700
C -0.89696100 1.68733000 2.95600300
H -0.58434500 1.48603600 3.99508900
H -0.00957200 1.57527400 2.31965000
H -1.21661400 2.73674700 2.89916900
C -5.79760300 0.85475500 2.00068600
H -5.84203500 1.94044000 1.83441300
H -6.35503200 0.36771500 1.18890700
H -6.31992200 0.63917300 2.94957900
N 1.53663600 -1.83442500 1.84366400
C -4.42652700 -0.71767100 -1.60175600
C -4.07435700 0.63620900 -1.31668200
C -5.08433200 1.60412800 -1.26315500
C -6.41276000 1.22361800 -1.48509600
C -6.74317700 -0.11414100 -1.75327600
C -5.75233600 -1.10367500 -1.81735600
C -2.14256700 -0.69047600 -1.34674200
C -2.64061100 0.64743400 -1.13723700
H -4.83863200 2.64720200 -1.04872200
H -7.20459100 1.97545900 -1.44384000
H -7.78688300 -0.38933200 -1.92406600
H -6.01275600 -2.14055700 -2.03961100
H -1.29597600 -0.77694700 -2.28581000
N -3.27234100 -1.48441500 -1.59561300
C -3.22728200 -2.87488700 -1.95587200
H -3.95487400 -3.46300400 -1.37107300
H -3.44526600 -3.03296200 -3.02828100
H -2.22180300 -3.26265800 -1.74391200
C -1.75530400 1.66062500 -0.75853700
H -2.13447000 2.67195900 -0.56357600
N -0.46359700 1.37974300 -0.58719700
C 0.45020200 2.37264000 -0.22571000
C 1.66800200 1.97925800 0.37083800
C 0.25150800 3.75035100 -0.42758800
C 2.61927100 2.90488100 0.76539500
H 1.86037400 0.91427400 0.49211500
C 1.20775000 4.69303300 -0.03181700
H -0.64917300 4.10449300 -0.93102100
C 2.39987100 4.27868600 0.57405700
H 3.56322500 2.58680700 1.21015300
H 1.01160600 5.74902700 -0.21879700
O 3.39750900 5.11502700 0.98385000
C 3.23522000 6.49649300 0.79130100
H 2.35489800 6.89004300 1.33337600
H 4.13933200 6.98123200 1.18472700
H 3.12853400 6.75579400 -0.27869300
H -0.24054900 -0.21748800 -3.91619100
C 2.12102800 -0.05296500 -2.42773400
C 1.83283500 1.26195300 -2.86017400
C 3.40216900 -0.29035200 -1.87934200
C 2.77682200 2.27974000 -2.73953500

H 0.84825700 1.49551700 -3.26629500
C 4.34029500 0.72902000 -1.75802200
H 3.64870700 -1.29784800 -1.54702900
C 4.03366800 2.02806500 -2.18317700
H 2.51569400 3.29178800 -3.05794800
H 5.31895700 0.50932900 -1.32198800
H 4.75985100 2.83588600 -2.07004600
C 2.69973900 -2.25598800 1.12055500
C 3.90995700 -1.56931200 1.31261700
C 2.60289900 -3.37325000 0.27086300
C 5.04178800 -2.03345100 0.63183700
C 3.75881100 -3.78630200 -0.39976300
C 4.98677800 -3.13120600 -0.23336400
H 5.98818000 -1.50256800 0.76464600
H 3.69866400 -4.64822300 -1.06957300
C 1.29868800 -4.10230800 0.08019900
H 0.78393600 -4.27274300 1.03773300
H 0.60659500 -3.51595700 -0.54724000
H 1.46043300 -5.07262800 -0.40909000
C 4.00152800 -0.36880300 2.21812000
H 4.83766900 0.27744100 1.91716900
H 3.08136200 0.22928100 2.19645800
H 4.17128400 -0.66545400 3.26704700
C 6.20232100 -3.56161900 -1.01231400
H 7.13177800 -3.34416200 -0.46529400
H 6.17778900 -4.63761400 -1.24055800
H 6.25348100 -3.02240800 -1.97402000

TS3-anti/M-Ph (singlet)

N 0.25038100 -1.52333500 -2.07736600
C 1.58713500 -1.00091200 -2.17653700
C 1.84095000 0.21172600 -2.82446200
C 2.63366900 -1.80730900 -1.68119800
C 3.17475000 0.64694800 -2.89931900
C 4.23161200 -0.09921900 -2.37874400
H 3.38447600 1.60115400 -3.39139600
C -0.56679800 -1.49068000 -0.96869700
Fe -0.31343700 -0.60006100 0.78249100
C -2.33108600 -0.08926000 1.12484900
H -2.99993700 -0.94327300 1.22209100
C -1.38843000 -2.90779800 -2.58896300
H -2.07743400 -3.62743000 -3.01790900
C -0.23859000 -2.37581100 -3.06375500
H 0.29272700 -2.52668600 -3.99826800
C 3.94004700 -1.33398200 -1.77968800
H 4.75412400 -1.93587400 -1.36834200
C -1.52696000 0.25962800 2.23261500
H -1.56572500 -0.41044200 3.10421400
C 2.34602700 -3.16570300 -1.10177900
H 1.55957300 -3.10890000 -0.33913300
H 1.99657600 -3.86395300 -1.88089800
H 3.24750100 -3.59272100 -0.64223400
C 0.75917800 1.04509800 -3.45589100
H 0.63958200 2.00475500 -2.93174400
H 1.01447400 1.27180900 -4.50347000
H -0.21544700 0.54558900 -3.43882900
C 5.65592500 0.38218600 -2.46522000
H 6.11476300 0.40893100 -1.46509200
H 6.26513000 -0.29307100 -3.08933000
H 5.71688800 1.39085000 -2.89940800
N -1.58271000 -2.35908800 -1.32243000
C 3.83242000 -0.93816000 1.66306500
C 3.69990000 0.34108900 1.04801900
C 4.83843700 1.14389500 0.90853300
C 6.07259700 0.66584700 1.36376000
C 6.18490900 -0.60226000 1.95573200
C 5.05859100 -1.42192800 2.11524800
C 1.61628700 -0.73672500 1.08958400
C 2.30578400 0.45843200 0.70501100
H 4.76660500 2.12887800 0.44104300
H 6.96438600 1.28849900 1.25595700
H 7.15939400 -0.95425300 2.30237000
H 5.14606700 -2.40510000 2.58229400
H 0.42086100 -0.32776100 2.08725600
N 2.57181900 -1.54638200 1.68546700
C 2.34681300 -2.81404700 2.33123400
H 2.95090900 -3.61534800 1.87320300
H 2.60451000 -2.76359500 3.40341300
H 1.29075900 -3.08274400 2.24114500
C 1.50150000 1.43832200 0.11621100
H 1.90264900 2.38712000 -0.25831800
N 0.21364600 1.16277200 -0.00519600
C -0.64882100 2.14213500 -0.53165000
C -1.70148100 1.78143100 -1.39576200
C -0.50745600 3.49931600 -0.20999100
C -2.55667500 2.73612000 -1.92653700
H -1.83023800 0.72989100 -1.64471000
C -1.35942800 4.47098300 -0.74305800

H 0.24808500 3.79974500 0.51669000
C -2.39453000 4.09555000 -1.60788200
H -3.36905300 2.45526700 -2.59976100
H -1.22262200 5.50967800 -0.44383700
O -3.28506200 4.95815000 -2.17008600
C -3.18171900 6.32638500 -1.86349200
H -2.20959400 6.74812400 -2.17911100
H -3.98522600 6.83574200 -2.41268200
H -3.31038500 6.51746900 -0.78210000
H -2.70497200 0.69502200 0.46680600
C -1.24430500 1.67889400 2.61870300
C -0.02156500 2.07147700 3.19027600
C -2.23221200 2.66189600 2.44701900
C 0.20353800 3.39231700 3.57771900
H 0.77526200 1.33235100 3.29900000
C -2.01175400 3.98651200 2.83245100
H -3.18684900 2.37884900 1.99995300
C -0.79237800 4.35992000 3.39998200
H 1.16764500 3.67117200 4.01112200
H -2.79739500 4.73105400 2.67964500
H -0.61432100 5.39625400 3.69832200
C -2.70043600 -2.72005700 -0.51092700
C -3.99969200 -2.46344500 -0.97922600
C -2.48117500 -3.29810100 0.75247800
C -5.08013700 -2.85265500 -0.17601300
C -3.58854300 -3.66781800 1.51827600
C -4.90001700 -3.46609400 1.06717600
H -6.09462200 -2.64609600 -0.52750200
H -3.42273300 -4.10917300 2.50495200
C -1.09682000 -3.40952000 1.32343000
H -0.35494500 -3.72183700 0.57873500
H -0.75647700 -2.44015100 1.75347600
H -1.07177500 -4.11396600 2.16746000
C -4.24477600 -1.72548400 -2.27127800
H -3.52127000 -0.90752100 -2.40322500
H -4.15496600 -2.37939500 -3.15379300
H -5.25637000 -1.29632500 -2.28162400
C -6.07571700 -3.89516700 1.90650500
H -5.93030600 -3.63025100 2.96536400
H -7.00985500 -3.42835300 1.56192200
H -6.21392500 -4.98921400 1.86205200

TS3-anti/M-Ph (triplet)

N 0.32353800 -1.75047300 -2.14074600
C 1.61311200 -1.12255500 -2.18838500
C 1.74988200 0.13148500 -2.79625100
C 2.71086800 -1.81383800 -1.64583700
C 3.03004300 0.69962400 -2.83728500
C 4.14801500 0.04485700 -2.31019600
H 3.15117700 1.68643300 -3.29303900
C -0.55069900 -1.68875000 -1.08054300
Fe -0.33889700 -0.65770700 0.58645300
C -2.17379800 -0.69433000 1.56763900
H -2.51821700 -1.68667200 1.87012600
C -1.32865800 -3.08280700 -2.73053400
H -2.02744600 -3.77225000 -3.19428500
C -0.13593100 -2.58842300 -3.14890200
H 0.43189200 -2.75163200 -4.05983100
C 3.96833700 -1.21102000 -1.71616100
H 4.82555200 -1.72070700 -1.26980700
C -1.29732300 0.02866600 2.43415300
H -0.98174200 -0.49274900 3.34817200
C 2.52537800 -3.16541300 -1.01115500
H 1.70400800 -3.14115400 -0.28217100
H 2.26975500 -3.93048600 -1.76371900
H 3.44223000 -3.48446800 -0.49677500
C 0.56698300 0.83888300 -3.39912100
H 0.37019300 0.47736600 -4.42364500
H -0.34282900 0.67479100 -2.81057200
H 0.74000000 1.92245000 -3.45224000
C 5.51978300 0.66299400 -2.39305200
H 5.46536100 1.76197200 -2.41352900
H 6.14058300 0.36622100 -1.53594300
H 6.04040300 0.34308800 -3.31271500
N -1.56572900 -2.52716000 -1.47633200
C 3.75470600 -0.97459700 1.85473800
C 3.70718400 0.27594100 1.17042400
C 4.89536900 1.00111100 1.00819300
C 6.09012300 0.47534000 1.50786300
C 6.11744300 -0.76476500 2.16996900
C 4.94401000 -1.50537900 2.35561900
C 1.58076600 -0.67766800 1.20392300
C 2.33040700 0.46173500 0.78163400
H 4.89118100 1.96051300 0.48583200
H 7.01999100 1.03550600 1.38126600
H 7.06507300 -1.15457300 2.54909200
H 4.96406400 -2.46547100 2.87557000

H 0.30268600 -0.20657900 1.97481800
N 2.46297700 -1.50174600 1.87512400
C 2.11660300 -2.74743600 2.51182800
H 2.70189800 -3.58622900 2.09872300
H 2.29501700 -2.70611100 3.59999100
H 1.05100000 -2.94263200 2.33678800
C 1.65582100 1.54186300 0.16213800
H 2.21649800 2.45152400 -0.10170300
N 0.37127900 1.44171400 -0.06885600
C -0.41461300 2.51222000 -0.48560200
C -1.68538000 2.22165100 -1.02543100
C -0.06541800 3.86534400 -0.34442900
C -2.55497100 3.22466100 -1.42381000
H -1.97124100 1.17231900 -1.10575400
C -0.93354300 4.88508400 -0.74152100
H 0.87817500 4.13652900 0.13035700
C -2.18827100 4.57331300 -1.28313100
H -3.53893200 2.99339500 -1.83653200
H -0.63041400 5.92128300 -0.59262100
O -3.10933500 5.49509300 -1.67985600
C -2.80266000 6.85940900 -1.53738100
H -1.91207400 7.14686700 -2.12635500
H -3.67073000 7.42001400 -1.91007700
H -2.62614000 7.13457400 -0.48103100
H -2.89432100 -0.13933900 0.95988100
C -1.32185800 1.51365700 2.59406200
C -0.22878800 2.17375900 3.18327400
C -2.40668800 2.29188800 2.16351800
C -0.22146700 3.55871200 3.33827600
H 0.63906600 1.58790700 3.49865700
C -2.40119300 3.68080900 2.31268200
H -3.26881600 1.80450800 1.70727600
C -1.31079200 4.32125600 2.90187500
H 0.64310000 4.04818100 3.79428700
H -3.25314900 4.26396600 1.95526400
H -1.30230900 5.40839400 3.01156200
C -2.71280300 -2.86076300 -0.68798100
C -3.92095900 -2.18598200 -0.91757500
C -2.59244800 -3.86050300 0.28850300
C -5.02633000 -2.53512800 -0.13471800
C -3.72501400 -4.18029600 1.04545800
C -4.94877600 -3.52708900 0.85156000
H -5.97274800 -2.01141700 -0.29534600
H -3.64591100 -4.95478700 1.81330700
C -1.26312700 -4.51649300 0.54646500
H -0.80752600 -4.89874800 -0.38009600
H -0.55407200 -3.78100800 0.96114000
H -1.35934100 -5.34748300 1.25874700
C -4.00231800 -1.09474300 -1.95103500
H -3.25492700 -0.31236300 -1.74882600
H -3.79196200 -1.47340100 -2.96406400
H -4.99720600 -0.62887300 -1.95897800
C -6.14485500 -3.85206800 1.70954700
H -6.22653600 -3.14175700 2.55043400
H -7.08257800 -3.78638400 1.13687300
H -6.07308900 -4.86255000 2.13841300

TS3-anti/M-Ph (quintet)

N 0.21772000 -2.95598400 -1.12494300
C 1.53893500 -2.48693700 -1.43702800
C 1.69724500 -1.57801800 -2.49212100
C 2.61530400 -2.91401000 -0.64338800
C 2.99142000 -1.11841800 -2.76306500
C 4.09559700 -1.53032400 -2.00970200
H 3.13677500 -0.40241000 -3.57587600
C -0.62580400 -2.28811200 -0.29360600
Fe -0.10430100 -0.59976200 0.85288300
C -1.07240400 0.30710400 2.54750800
H -1.29835800 -0.36982500 3.38171200
C -1.64385000 -4.13261300 -1.16032400
H -2.45312500 -4.84072700 -1.31003400
C -0.38135600 -4.08777600 -1.66605000
H 0.14350000 -4.74564500 -2.35229900
C 3.88536200 -2.42258000 -0.94873700
H 4.73516000 -2.72912800 -0.33320000
C 0.06364500 1.19973900 2.75133000
H 0.69918300 0.98972800 3.62292100
C 2.38288300 -3.83454200 0.52437500
H 1.72368400 -3.35694600 1.26743400
H 1.89090000 -4.77161600 0.21790700
H 3.32902600 -4.09024300 1.02047400
C 0.50575400 -1.07999900 -3.26638000
H -0.09842500 -1.90853800 -3.66931600
H -0.15105500 -0.48282700 -2.61441300
H 0.82006700 -0.44437100 -4.10550200
C 5.48261700 -1.03286100 -2.31466200
H 5.46962500 -0.24569700 -3.08099800

H 5.95500900 -0.61476900 -1.41385900
H 6.12339700 -1.85441500 -2.67789400
N -1.77144900 -3.02659200 -0.32653400
C 4.21369200 0.41569700 1.02103800
C 3.66318600 1.22483300 -0.01818900
C 4.51958200 2.03831200 -0.77080600
C 5.88918900 2.04070900 -0.48475800
C 6.41483900 1.23287000 0.53681100
C 5.58163900 0.40607400 1.30300700
C 1.95099700 0.02713500 1.04081800
C 2.24222300 0.97295700 -0.00399300
H 4.12218200 2.66821700 -1.57040700
H 6.56147800 2.67564700 -1.06681000
H 7.48817400 1.24958800 0.74076900
H 5.99185000 -0.21564200 2.10143900
H 1.14363000 0.58884700 1.88923100
N 3.18051000 -0.29024100 1.62093500
C 3.35444500 -1.17398900 2.74197100
H 4.07054700 -1.98169600 2.51016600
H 3.72452600 -0.63620500 3.63299900
H 2.38376100 -1.62634000 2.98326400
C 1.17349800 1.51837500 -0.72931100
H 1.35691800 2.31261200 -1.46512700
N -0.05268300 1.07245900 -0.49929100
C -1.18888300 1.69302100 -1.01085800
C -2.42605300 1.02902400 -0.86069400
C -1.21309200 2.97309800 -1.59556100
C -3.61638300 1.59830800 -1.28816600
H -2.43701100 0.05417200 -0.37470500
C -2.40458000 3.54731400 -2.04420300
H -0.29695500 3.55911300 -1.66569600
C -3.62045500 2.86369500 -1.89643300
H -4.56708000 1.07991000 -1.15277600
H -2.37293100 4.54481300 -2.48252400
O -4.83281800 3.34440800 -2.28980500
C -4.89330400 4.61868600 -2.88036000
H -4.30230200 4.67026200 -3.81349400
H -5.94896100 4.80828500 -3.11782300
H -4.53384400 5.40880700 -2.19543300
H -1.98507600 0.76085800 2.14045100
C 0.14158000 2.59156900 2.28043800
C 1.39315600 3.24868600 2.24780400
C -0.97668600 3.29917300 1.79310400
C 1.51692800 4.55144200 1.77075700
H 2.28213700 2.71306600 2.59107200
C -0.85203800 4.60376000 1.31706200
H -1.95526700 2.81821000 1.78675700
C 0.39278400 5.24168600 1.30237600
H 2.50043100 5.02845500 1.75540400
H -1.73879200 5.12389600 0.94618900
H 0.48761000 6.26316700 0.92571900
C -2.97201400 -2.61795600 0.34201700
C -4.01177400 -2.06317600 -0.42545900
C -3.04961600 -2.73066600 1.73931300
C -5.14082000 -1.59053400 0.25159900
C -4.19789000 -2.23749400 2.36924000
C -5.24640700 -1.65494200 1.64721400
H -5.95486000 -1.14717300 -0.32821800
H -4.27074800 -2.30756100 3.45775100
C -1.92765500 -3.33957900 2.53705800
H -1.52658800 -4.24234500 2.05229000
H -1.09430000 -2.62424500 2.63399900
H -2.26523300 -3.60401100 3.54875900
C -3.90591500 -1.96321800 -1.92558600
H -2.93177800 -1.55412900 -2.23136900
H -4.00973600 -2.94984300 -2.40620200
H -4.68911300 -1.30831400 -2.32976200
C -6.44471100 -1.07799100 2.35584800
H -7.35167500 -1.14235200 1.73617300
H -6.63861500 -1.59343900 3.30829700
H -6.28087900 -0.01133300 2.58739400

int4-M-Ph (singlet)

N -0.59645400 -0.60404500 2.34239200
C -1.85803000 0.06810900 2.20421200
C -1.94361500 1.45220800 2.38822400
C -2.99552900 -0.71864900 1.93391000
C -3.20374200 2.05320600 2.24002100
C -4.34914500 1.31274900 1.94257200
H -3.28311500 3.13729100 2.36321800
C 0.25553700 -0.94959000 1.31897100
Fe 0.15202400 -0.61592400 -0.63527500
C 0.53148200 -0.82468600 -2.60973200
H 0.71060200 -1.92168200 -2.60628800
C 0.95111000 -1.82538700 3.33147300
H 1.60021500 -2.39212800 3.99182400
C -0.18819600 -1.12807100 3.56380100
H -0.75263600 -0.95384600 4.47470100

C -4.22564000 -0.07674200 1.79939400
H -5.10839100 -0.67077700 1.55050800
C -2.89487900 -2.21707600 1.82971800
H -1.99358700 -2.52330900 1.28538300
H -2.84326500 -2.67841600 2.83117400
H -3.77175900 -2.62933600 1.31216800
C -0.75276100 2.29615600 2.75596200
H -0.86164700 2.68828100 3.78138100
H 0.18721500 1.73756700 2.70109000
H -0.65774700 3.15998400 2.08214100
C -5.69446200 1.97236200 1.78659300
H -5.60784000 3.06851300 1.75782600
H -6.18487700 1.63824300 0.85963200
H -6.36321500 1.70989200 2.62397600
N 1.20975200 -1.70399300 1.96862400
C -3.85580800 -1.20624200 -1.57009200
C -3.74156300 0.20540200 -1.39541900
C -4.88237600 0.99936500 -1.56280000
C -6.10188100 0.38959800 -1.87856200
C -6.19686600 -1.00272100 -2.03190300
C -5.06756000 -1.81990200 -1.88094000
C -1.67228700 -0.81065100 -1.00275800
C -2.36206100 0.43914100 -1.04838100
H -4.82342200 2.08330500 -1.43877200
H -6.99537800 1.00589400 -2.00713400
H -7.16035400 -1.45702600 -2.27473600
H -5.14325600 -2.90278300 -2.00051200
N -2.59257300 -1.77582000 -1.35492700
C -2.31283200 -3.17253600 -1.57227400
H -2.65343000 -3.80629800 -0.73434400
H -2.80774400 -3.52257900 -2.49188200
H -1.23166900 -3.30398100 -1.69746700
C -1.56286200 1.55447100 -0.77052400
H -1.95127200 2.57807900 -0.73946200
N -0.28988600 1.29901700 -0.50352300
C 0.64083400 2.31847400 -0.27300200
C 1.78771100 2.03415700 0.49271100
C 0.50788900 3.61843300 -0.78706000
C 2.74049000 3.00499200 0.75423400
H 1.91511800 1.02383500 0.87427900
C 1.46308000 4.60581500 -0.52691000
H -0.33688100 3.86250700 -1.43278800
C 2.59102400 4.30603000 0.24893000
H 3.63109900 2.77741500 1.34294600
H 1.32451900 5.59809000 -0.95601200
O 3.58449600 5.18931000 0.54622500
C 3.49253600 6.49993800 0.04707400
H 2.59029100 7.02110000 0.41750000
H 4.38294400 7.03728700 0.40079600
H 3.47853500 6.52091300 -1.05844500
C 1.78797300 -0.09604000 -2.43315800
C 1.98914400 1.22342800 -2.92316100
C 2.82601000 -0.64989200 -1.64093000
C 3.14947600 1.92988300 -2.64533900
H 1.20767800 1.68984000 -3.52408100
C 3.99944900 0.06937100 -1.37361300
H 2.75152300 -1.68863100 -1.31308800
C 4.16890200 1.35994200 -1.86546800
H 3.26523000 2.94617500 -3.03019200
H 4.79170400 -0.40336200 -0.79058100
H 5.07920800 1.92323600 -1.64946600
C -0.40410800 -0.40745200 -3.72526000
H -0.81399400 0.60049000 -3.55329300
H 0.08600900 -0.40256100 -4.71790200
H -1.27041100 -1.08275600 -3.78205000
C 2.29846000 -2.37087700 1.32417200
C 2.02200900 -3.54160300 0.59310300
C 3.60353500 -1.87268300 1.45581100
C 3.08173200 -4.18744400 -0.04830300
C 4.63776700 -2.57374100 0.82080500
C 4.39859000 -3.71550000 0.05017200
H 2.87644500 -5.08834700 -0.63257000
H 5.66071400 -2.19919200 0.91677000
C 3.90291500 -0.58924600 2.18393200
H 3.15251500 -0.34733700 2.94687500
H 4.89106000 -0.63143400 2.66482600
H 3.92262400 0.24620000 1.46502900
C 0.61701800 -4.07523700 0.51159100
H 0.22072500 -4.31071200 1.51264000
H -0.06811400 -3.33162700 0.08003900
H 0.57205100 -4.98460400 -0.10273200
C 5.52057200 -4.39922900 -0.68700400
H 5.63709700 -3.96865800 -1.69655800
H 6.48162800 -4.27590400 -0.16582700
H 5.32854000 -5.47560600 -0.80993300

int4-M-Ph (triplet)

N -0.37856300 -0.87327200 2.30022700
C -1.68358300 -0.27934700 2.21608700
C -1.84809600 1.08941500 2.45147400
C -2.77780000 -1.13058900 1.96643100
C -3.14662900 1.61414400 2.36171400
C -4.25313100 0.81073500 2.07912800
H -3.28911200 2.68674500 2.52128900
C 0.48194100 -1.10751200 1.25503400
Fe 0.28896700 -0.59788900 -0.72015900
C 0.48770500 -0.88269400 -2.74898100
H 0.76181600 -1.95123800 -2.75946000
C 1.26589000 -2.01720400 3.21686300
H 1.96244200 -2.56620500 3.84293100
C 0.08315000 -1.41612000 3.49329400
H -0.48059200 -1.32349500 4.41638300
C -4.04966500 -0.56444100 1.89322600
H -4.90286200 -1.20660900 1.66125100
C -2.58290900 -2.61623900 1.82427300
H -1.68817400 -2.85005800 1.23414700
H -2.45532900 -3.09182500 2.81215400
H -3.45364400 -3.07713800 1.33843200
C -0.69826000 1.98002600 2.83567700
H -0.68822800 2.14460300 3.92741700
H 0.26881200 1.55304800 2.55130300
H -0.77978400 2.96363700 2.35312900
C -5.64068500 1.38891100 1.98285500
H -5.62584800 2.48790800 2.02455900
H -6.12228300 1.08365600 1.04128800
H -6.27801900 1.02859500 2.80807800
N 1.49527600 -1.81788300 1.85853400
C -3.75417300 -1.49664900 -1.47613000
C -3.73614000 -0.08341600 -1.29922200
C -4.93572000 0.62705400 -1.43861200
C -6.11319600 -0.06948600 -1.73027800
C -6.11093200 -1.46554100 -1.88919500
C -4.92296300 -2.19830400 -1.76700100
C -1.59173000 -0.94337000 -0.96762900
C -2.36713800 0.25401300 -0.99171700
H -4.95430900 1.71247800 -1.31409700
H -7.05177100 0.48039700 -1.83713100
H -7.04403100 -1.98654900 -2.11612900
H -4.91896800 -3.28275200 -1.89566300
N -2.44948000 -1.96912700 -1.29004300
C -2.07998200 -3.33985700 -1.53680800
H -2.53678800 -4.02849800 -0.80592100
H -2.39649900 -3.65059700 -2.54638400
H -0.99069400 -3.42658800 -1.47373400
C -1.72685800 1.48304200 -0.73275100
H -2.31534800 2.41152700 -0.69245600
N -0.43152800 1.48694700 -0.52004300
C 0.28563600 2.66116000 -0.29057500
C 1.52124300 2.55986200 0.37946600
C -0.12225300 3.94074800 -0.70388600
C 2.29075200 3.67689900 0.66066600
H 1.86696800 1.56479300 0.65953500
C 0.64941200 5.07560400 -0.43168500
H -1.04555800 4.06045400 -1.27334900
C 1.86176700 4.95341200 0.26127900
H 3.24838700 3.58832600 1.17709100
H 0.29853600 6.04698100 -0.78027200
O 2.68502700 5.99414200 0.57126100
C 2.30786000 7.29070000 0.18225600
H 1.34856800 7.59710500 0.63929300
H 3.09905300 7.96902000 0.52969900
H 2.21581300 7.38272500 -0.91594700
C 1.61932200 -0.01517600 -2.50405700
C 1.69702900 1.32801700 -2.98768900
C 2.64985300 -0.44870800 -1.61954800
C 2.74684100 2.15376200 -2.64202400
H 0.91054900 1.69835800 -3.64585100
C 3.72009800 0.40951300 -1.28896300
H 2.71055200 -1.50339200 -1.34215300
C 3.77544300 1.69978600 -1.78855300
H 2.77684000 3.17684200 -3.02514300
H 4.52211900 0.03408900 -0.65330800
H 4.60115100 2.36354500 -1.52437700
C -0.54792600 -0.51813000 -3.78664500
H -1.01988900 0.45191300 -3.56873700
H -0.12116700 -0.46322800 -4.80629900
H -1.36018100 -1.25935900 -3.80571800
C 2.65693900 -2.33625900 1.20422500
C 2.54161700 -3.50892900 0.44068400
C 3.88419900 -1.67715700 1.37466800
C 3.69029100 -3.99988000 -0.18905900
C 5.00981100 -2.21555000 0.74130300
C 4.93208900 -3.36539000 -0.05453200
H 3.61248800 -4.90392600 -0.79885200
H 5.97183500 -1.70878000 0.85697300

C 3.96882500 -0.40579200 2.17646700
H 3.74245400 -0.57490400 3.24117500
H 4.97140700 0.03781700 2.10761600
H 3.23980000 0.33252100 1.80952300
C 1.21014600 -4.19271100 0.29607600
H 0.80048300 -4.48676500 1.27596600
H 0.47764400 -3.50647600 -0.15309400
H 1.28710400 -5.08966700 -0.33328900
C 6.14788100 -3.88208400 -0.77955100
H 6.26875700 -3.36796900 -1.74871300
H 7.06781100 -3.70786000 -0.20125500
H 6.06856000 -4.95950800 -0.98691100

int4-M-Ph (quintet)

N -0.50659400 -1.28315000 2.29840700
C -1.84299200 -0.75932400 2.23473600
C -2.08392900 0.56954100 2.60482000
C -2.86719900 -1.61413100 1.79173200
C -3.39815900 1.04348500 2.49688400
C -4.44470600 0.23192500 2.04697300
H -3.60411200 2.08417900 2.76107600
C 0.32309000 -1.39607200 1.22221900
Fe -0.15039300 -0.69865100 -0.73580500
C 1.03546200 -0.85762900 -2.45724700
H 1.49161300 -1.86255400 -2.44441600
C 1.28745800 -2.27771400 3.09220400
H 2.06487600 -2.77236800 3.66566400
C 0.06153500 -1.81356800 3.44913800
H -0.46133700 -1.81578600 4.40070500
C -4.15986300 -1.09675900 1.70514000
H -4.96516400 -1.73853500 1.33936700
C -2.56416900 -3.03715000 1.40764200
H -1.89760700 -3.07080900 0.53293600
H -2.05373900 -3.57739900 2.22119500
H -3.48494800 -3.58171700 1.15874200
C -0.97313700 1.45488600 3.10233700
H -0.72761300 1.23298200 4.15525100
H -0.05407600 1.32342300 2.51726500
H -1.25836100 2.51422000 3.04268000
C -5.85268600 0.75372800 1.93774900
H -5.88000600 1.85181500 1.97507000
H -6.31666000 0.43761300 0.99237200
H -6.47736700 0.37144500 2.76363500
N 1.42700700 -2.01557200 1.73243800
C -4.42462600 -0.42884100 -1.65913900
C -4.03285000 0.87806000 -1.24723600
C -4.99368000 1.89948100 -1.23334400
C -6.30542100 1.60580100 -1.61156800
C -6.67567200 0.30551300 -2.00227000
C -5.73567700 -0.73008400 -2.03302900
C -2.20055800 -0.56007400 -1.14662500
C -2.62666500 0.77351200 -0.93257500
H -4.72345800 2.91283600 -0.92572800
H -7.06014500 2.39601800 -1.60012700
H -7.70962100 0.10243600 -2.29108900
H -6.02222700 -1.73689600 -2.34413400
N -3.29575100 -1.24870100 -1.59552000
C -3.30241700 -2.63306900 -2.00331900
H -3.98501100 -3.23431100 -1.37943600
H -3.61989400 -2.73196600 -3.05477300
H -2.28425400 -3.02846900 -1.90495700
C -1.69186600 1.75070700 -0.49666300
H -2.04269700 2.77437100 -0.29451900
N -0.43577600 1.42975300 -0.32201600
C 0.53796100 2.36677300 0.04721900
C 1.73622200 1.88266300 0.60761000
C 0.42305400 3.75128700 -0.14967700
C 2.75939700 2.73865300 0.97756500
H 1.85686100 0.80594400 0.71106400
C 1.45153100 4.62461200 0.21817100
H -0.46349400 4.16437500 -0.63360500
C 2.62974500 4.12334300 0.78718600
H 3.69428300 2.35588200 1.38799000
H 1.32844200 5.69133000 0.03157200
O 3.69511200 4.88534400 1.15747100
C 3.63806100 6.27391900 0.94594400
H 2.81137400 6.74448800 1.50985200
H 4.59107400 6.68814300 1.30203200
H 3.51763500 6.52304900 -0.12445400
C 2.09876300 0.16471100 -2.34584500
C 1.92209800 1.50268600 -2.76643700
C 3.34695000 -0.15516600 -1.76420900
C 2.92123700 2.46014000 -2.59054700
H 0.97464100 1.80138900 -3.21851200
C 4.34493800 0.79998200 -1.58899900
H 3.52416600 -1.18343700 -1.45408100
C 4.14099800 2.12328300 -1.99689700

H 2.73821300 3.48950200 -2.91044800
H 5.29390100 0.50628000 -1.12987900
H 4.91482500 2.87972000 -1.84769800
C 0.08594700 -0.71216800 -3.64793700
H -0.56164600 0.17671900 -3.56828600
H 0.62528800 -0.63075100 -4.61305300
H -0.59262100 -1.57760300 -3.71795200
C 2.58389900 -2.40170800 0.97829000
C 2.46194600 -3.42120500 0.01641400
C 3.81489500 -1.78306400 1.25491000
C 3.61291000 -3.80076100 -0.68154200
C 4.93982500 -2.21329800 0.54176700
C 4.85939300 -3.20987400 -0.43605700
H 3.53199700 -4.58269100 -1.44111700
H 5.90126200 -1.73144600 0.73811400
C 3.94193000 -0.68387300 2.27814400
H 4.12248500 -1.08845300 3.28853100
H 4.78708800 -0.02672500 2.03035900
H 3.03533200 -0.06771700 2.33370200
C 1.13948100 -4.08070100 -0.27284300
H 0.57898300 -4.29573500 0.64906800
H 0.50479900 -3.42207600 -0.88864900
H 1.28482700 -5.02084800 -0.82263500
C 6.06827200 -3.59341700 -1.24862400
H 6.14225600 -2.95715900 -2.14739100
H 6.99917500 -3.46299300 -0.67686300
H 6.01341800 -4.63810600 -1.58943300

int4-anti/M-Ph (singlet)

N -0.26590100 -2.31425100 1.69776900
C -1.55715400 -1.78528800 2.02997300
C -1.64136000 -0.72142800 2.93578400
C -2.69142200 -2.33691100 1.41348100
C -2.91050500 -0.19352700 3.20506600
C -4.06359900 -0.69698100 2.59486600
H -2.99544700 0.64694000 3.89913000
C 0.47402300 -1.90457500 0.62687900
Fe 0.24410200 -0.65707100 -0.89278500
C 0.57302800 0.08274800 -2.71387800
H 0.54110600 -0.82122000 -3.36482400
C 1.57195700 -3.53683700 1.80676300
H 2.37727400 -4.22877200 2.03208700
C 0.38344200 -3.30863200 2.42590600
H -0.06518600 -3.75871100 3.30641600
C -3.93381600 -1.77224500 1.70503900
H -4.82457000 -2.16646500 1.20947700
C -2.55622500 -3.50103700 0.46996200
H -1.83010800 -3.28038200 -0.32448800
H -2.19581600 -4.40202800 0.99417200
H -3.52056900 -3.74261700 0.00281800
C -0.40188100 -0.16137700 3.58063300
H 0.05022500 -0.88302700 4.28168100
H 0.36317000 0.07955000 2.82950200
H -0.62996600 0.75444000 4.14341900
C -5.41912900 -0.09696800 2.85897500
H -5.36653000 0.70953300 3.60473000
H -5.83645700 0.32440700 1.93117100
H -6.12721500 -0.85773800 3.22669900
N 1.60874500 -2.67317200 0.71710200
C -3.82603600 -0.08419500 -1.29396000
C -3.41691700 0.94771100 -0.39570600
C -4.36519400 1.88290100 0.03413700
C -5.68793800 1.77173000 -0.41209600
C -6.07476200 0.73961500 -1.28121500
C -5.14062200 -0.20258600 -1.73782100
C -1.58473600 -0.39073300 -0.95187300
C -2.00616400 0.74259600 -0.18688500
H -4.07547700 2.69065500 0.71021900
H -6.43198500 2.49986800 -0.07920200
H -7.11285900 0.67242700 -1.61523400
H -5.43963300 -0.99673700 -2.42531500
N -2.70427900 -0.86432400 -1.60607000
C -2.72265100 -1.89604300 -2.61143700
H -3.41715200 -2.71091500 -2.34766500
H -3.02557300 -1.48921600 -3.59167300
H -1.71290100 -2.31170100 -2.71074000
C -0.96603000 1.44064200 0.43661600
H -1.10987800 2.37174800 0.99270200
N 0.24427300 0.93903000 0.23112200
C 1.41428400 1.49965100 0.73739700
C 2.63737100 1.13862400 0.13407200
C 1.46461500 2.40056000 1.81523100
C 3.84551300 1.65495000 0.57877000
H 2.62930100 0.46268600 -0.72095500
C 2.67784400 2.92654300 2.26851500
H 0.54496000 2.69523600 2.32125400
C 3.88269200 2.55582100 1.65448700
H 4.78507700 1.37393100 0.10009000

H 2.66776700 3.61909300 3.11008600
O 5.11444400 3.00126400 2.02993900
C 5.20314100 3.93036900 3.08123300
H 4.81453000 3.51855700 4.03119400
H 6.26860400 4.16622600 3.20687800
H 4.65467800 4.86325300 2.85469000
C -0.26553900 1.17268500 -3.39316200
H 0.13945400 1.41613300 -4.39795900
H -1.28457700 0.79209400 -3.56941400
C 2.67815100 -2.47812200 -0.21001200
C 2.43465800 -2.74377400 -1.57260800
C 3.88703100 -1.91865300 0.23438000
C 3.43171000 -2.41194100 -2.49411700
C 4.86362800 -1.62746400 -0.72655900
C 4.65075500 -1.84915000 -2.09177800
H 3.24921700 -2.59523100 -3.55600500
H 5.80606400 -1.18239900 -0.39715900
C 4.10562200 -1.58702800 1.68686200
H 3.23171700 -1.06508200 2.10351300
H 4.27061500 -2.49179400 2.29446600
H 4.97836200 -0.93247900 1.80909200
C 1.15018600 -3.39310000 -2.02436900
H 0.98233400 -4.34633300 -1.49836200
H 0.25523000 -2.78124500 -1.81144000
H 1.16565500 -3.58101200 -3.10588300
C 5.68887600 -1.46950600 -3.11612000
H 5.35056000 -0.60560900 -3.71259900
H 6.64381100 -1.19756400 -2.64401100
H 5.87685300 -2.29463200 -3.82130300
H 1.64358600 0.38263600 -2.71103100
C -0.37918800 2.46069200 -2.60800900
C -1.62899800 3.04659400 -2.36401500
C 0.75632400 3.08560100 -2.07215200
C -1.74816600 4.20383300 -1.59143300
H -2.52916500 2.56517200 -2.75319000
C 0.64700300 4.23801300 -1.29243800
H 1.74095400 2.64425900 -2.23690700
C -0.60903200 4.80023800 -1.04267100
H -2.73844200 4.62592800 -1.40138300
H 1.54580500 4.68803400 -0.86332800
H -0.69823200 5.69875300 -0.42624200

int4-anti/M-Ph (triplet)

N -0.52477300 -1.50424900 2.32554300
C -1.87295100 -1.01441000 2.26578900
C -2.14096400 0.28217800 2.72596900
C -2.87409100 -1.83887600 1.72722700
C -3.45174000 0.75833000 2.61038700
C -4.47633800 -0.02200200 2.06340200
H -3.67598600 1.77382000 2.94720000
C 0.36752100 -1.36737400 1.29719400
Fe -0.30666500 -0.66960900 -0.49450300
C 0.01773900 -2.29586800 -1.59020200
H -0.65238300 -3.16284600 -1.43425400
C 1.32758400 -2.41349300 3.08999600
H 2.12990000 -2.89471800 3.64088600
C 0.03984700 -2.14089000 3.42547700
H -0.52706300 -2.33667900 4.33059100
C -4.16775600 -1.32051400 1.63549800
H -4.95758700 -1.94257100 1.20576200
C -2.54077500 -3.22237800 1.23839500
H -1.81580700 -3.17455200 0.41178900
H -2.08089900 -3.83316800 2.03198700
H -3.43996100 -3.74310100 0.88189300
C -1.04252700 1.13092200 3.30711200
H -0.66111600 0.70872700 4.25164400
H -0.19038600 1.19762600 2.61663200
H -1.39967500 2.14958800 3.51298900
C -5.87284800 0.51801600 1.90991300
H -6.00748000 1.45478500 2.46955900
H -6.09233900 0.72773100 0.85155300
H -6.62260100 -0.20711600 2.26471700
N 1.50733900 -1.93722100 1.79378900
C -4.33795900 0.26815500 -1.54175600
C -3.80854900 1.49646800 -1.04819200
C -4.66107100 2.60379600 -0.93585500
C -5.99937700 2.47561700 -1.31816000
C -6.50188900 1.25591400 -1.80522800
C -5.67345900 0.13335500 -1.92213100
C -2.13280100 -0.13622200 -1.08538000
C -2.42205400 1.22064500 -0.75919600
H -4.28636400 3.55680800 -0.55397300
H -6.66874500 3.33538400 -1.23507100
H -7.55260600 1.18155600 -2.09512800
H -6.06490200 -0.81351100 -2.29923100
N -3.30523100 -0.67395600 -1.55286300
C -3.50301800 -2.03141200 -2.00167200
H -4.28948200 -2.52608300 -1.40741200

H -3.80450800 -2.05988500 -3.06222900
H -2.56682800 -2.58259300 -1.88056800
C -1.34579700 1.99436000 -0.27837100
H -1.43321300 3.08204500 -0.14003100
N -0.20282900 1.38076300 -0.05678300
C 0.96381400 2.09530400 0.24867200
C 2.17207900 1.66386300 -0.33177200
C 0.99884300 3.21791100 1.08992600
C 3.35612500 2.34793500 -0.10933100
H 2.16706200 0.78448900 -0.97470200
C 2.19617300 3.89897700 1.34000200
H 0.08295000 3.55988900 1.57527800
C 3.38521800 3.46833800 0.73641800
H 4.28565900 2.01920100 -0.57582800
H 2.18532100 4.75634400 2.01273700
O 4.60346000 4.04825700 0.92628300
C 4.68874100 5.18549100 1.74876000
H 4.37810800 4.96850000 2.78763600
H 5.74225800 5.49635100 1.75144500
H 4.07089300 6.01852900 1.36594400
C 0.01981700 -1.86570400 -3.06664800
H 0.36111800 -2.68817400 -3.72638200
H -0.99646700 -1.60246000 -3.39878600
C 2.75861600 -1.94693500 1.09586800
C 3.07558000 -3.02001700 0.24947800
C 3.63602600 -0.86646500 1.29715000
C 4.25502100 -2.92886000 -0.50137600
C 4.80618300 -0.82628200 0.53378200
C 5.11540000 -1.83026100 -0.39438100
H 4.50614400 -3.74250500 -1.18755100
H 5.49009300 0.01625000 0.66390300
C 3.33683200 0.17973200 2.33802200
H 2.34337500 0.62524500 2.19059400
H 3.34938900 -0.26296300 3.34799600
H 4.07676200 0.98874200 2.30622100
C 2.22492100 -4.26313600 0.20241500
H 2.56319800 -4.98117800 0.97011800
H 1.16688200 -4.04785100 0.39398900
H 2.30311200 -4.76127800 -0.77398900
C 6.33857800 -1.71589200 -1.26715600
H 6.12368800 -1.09445300 -2.15394400
H 7.17452600 -1.23930000 -0.73281300
H 6.67414300 -2.69910300 -1.62881500
H 1.03241500 -2.64416300 -1.33946600
C 0.92606400 -0.67174100 -3.27875400
C 0.40897800 0.62329300 -3.44194400
C 2.32306600 -0.83143000 -3.27170400
C 1.25844600 1.71963900 -3.61652500
H -0.67313700 0.77049800 -3.42337400
C 3.17405900 0.26106200 -3.45050100
H 2.74528500 -1.82909900 -3.12467800
C 2.64361500 1.54280200 -3.62956900
H 0.83356200 2.71923900 -3.73535400
H 4.25709300 0.11204600 -3.44701400
H 3.30712500 2.40071400 -3.75973700

int4-anti/M-Ph (quintet)

N -0.16171300 -2.67426100 1.36069100
C -1.52164900 -2.30051500 1.62774400
C -1.76901600 -1.31559800 2.59630300
C -2.54830300 -2.88586400 0.87524100
C -3.09243800 -0.91383900 2.79175300
C -4.14972600 -1.46339400 2.05460200
H -3.30363100 -0.12962400 3.52352700
C 0.60173700 -2.08105200 0.40003000
Fe -0.02042300 -0.60259400 -0.98830500
C 0.95653400 -0.25406900 -2.78707800
H 1.06038500 -1.19095200 -3.36624100
C 1.81730800 -3.61552600 1.56903600
H 2.70093200 -4.19193800 1.82529600
C 0.55952800 -3.61605200 2.08595700
H 0.11058900 -4.19160300 2.88984700
C -3.85837000 -2.45592100 1.11204200
H -4.67269800 -2.89383300 0.52885000
C -2.23194000 -3.90473500 -0.18637500
H -1.65403300 -3.44348000 -1.00372700
H -1.61924900 -4.73059800 0.20816900
H -3.15025500 -4.32952000 -0.61446800
C -0.63335600 -0.68714800 3.35897200
H -0.11684900 -1.41985200 4.00059200
H 0.11905800 -0.27387800 2.67173900
H -0.99554000 0.12803400 4.00045000
C -5.55447500 -0.95767400 2.24455700
H -5.78940200 -0.81535700 3.31100000
H -5.67996700 0.01648800 1.74517300
H -6.29521600 -1.64796100 1.81570100
N 1.82010800 -2.67376600 0.54613500

C -4.24990700 0.50431500 -0.89916100
C -3.60141900 1.38963700 0.01132200
C -4.36232400 2.38386600 0.64379400
C -5.73014800 2.47205100 0.37100700
C -6.35221400 1.58231100 -0.52469200
C -5.61639000 0.58466600 -1.17231400
C -2.05181500 -0.11920500 -0.85322900
C -2.21664000 0.98225800 0.02232700
H -3.89376200 3.08182300 1.34214200
H -6.32932100 3.24358500 0.86084200
H -7.42386300 1.67179200 -0.71727200
H -6.09946700 -0.10500100 -1.86754700
N -3.28221100 -0.36938500 -1.39814100
C -3.56784400 -1.39497600 -2.37136100
H -4.22225000 -2.17809200 -1.95171200
H -4.06577900 -0.96730400 -3.25700800
H -2.62014700 -1.84945200 -2.68434400
C -1.07328700 1.59205500 0.60222800
H -1.19560700 2.48998200 1.22663700
N 0.12111200 1.12104900 0.34887800
C 1.27219800 1.67350800 0.91991900
C 2.48416700 1.57355700 0.20620000
C 1.30286000 2.30073500 2.17621100
C 3.65955000 2.10845000 0.70854200
H 2.47953300 1.06393400 -0.75542400
C 2.48826700 2.83207200 2.69689800
H 0.39302700 2.35953600 2.77596500
C 3.67755800 2.74330000 1.96165000
H 4.59439300 2.03184500 0.15083600
H 2.46986600 3.29838500 3.68182900
O 4.88522800 3.20940200 2.38388600
C 4.95793200 3.87260200 3.62180200
H 4.66864900 3.21349200 4.46126700
H 6.00410900 4.17914300 3.75596000
H 4.31542500 4.77185700 3.64568000
C 0.21780500 0.78245800 -3.65899400
H 0.79082000 1.01427800 -4.58079500
H -0.73993600 0.35625900 -4.00357700
C 2.97327900 -2.29121800 -0.21548400
C 3.08483100 -2.72818400 -1.54568100
C 3.93710000 -1.47162200 0.39182600
C 4.18458000 -2.28492800 -2.28498600
C 5.02507500 -1.06145300 -0.38871600
C 5.15811500 -1.44362700 -1.72847400
H 4.28054200 -2.60171100 -3.32692800
H 5.78065100 -0.41412700 0.06357500
C 3.80344400 -1.04658300 1.83085100
H 3.94409200 -1.89809500 2.51696000
H 4.54628500 -0.27904800 2.08162100
H 2.80685900 -0.62624500 2.03080000
C 2.04786900 -3.63912800 -2.14614400
H 1.89014800 -4.53361500 -1.52251100
H 1.07982100 -3.12197200 -2.22465800
H 2.34524400 -3.96361500 -3.15254000
C 6.30604100 -0.94494800 -2.56860300
H 5.96872100 -0.15301900 -3.25907500
H 7.11027500 -0.52403800 -1.94769900
H 6.73318600 -1.75064100 -3.18606600
H 1.99604900 0.07974600 -2.60284200
C -0.07608800 2.08378700 -2.94389400
C -1.39006600 2.50442000 -2.69833900
C 0.97152200 2.89076900 -2.47013300
C -1.65488500 3.67449500 -1.98219200
H -2.22181400 1.88947100 -3.04960600
C 0.71739300 4.05433500 -1.74510100
H 2.00442500 2.58815700 -2.65806000
C -0.60129700 4.44863400 -1.49160900
H -2.69010700 3.96360500 -1.78537600
H 1.55123600 4.65062100 -1.36609300
H -0.80389800 5.35717000 -0.91851400

TS5-M-Ph (singlet)

Fe -0.57105500 -0.70636500 -0.38151700
C -1.08830500 0.33646200 -2.23164600
H -0.54398900 -0.63415800 -2.33934900
N -1.56675900 -2.36070300 1.70984000
C -0.61597000 -1.42172800 1.35900300
C -1.46730400 -2.72472300 3.04990700
C -0.42822000 -2.01159800 3.55902900
H -2.12338000 -3.45952100 3.50630400
H 0.00516000 -1.99171400 4.55440900
C 1.06438500 -0.19694400 2.69954400
C 0.60821100 1.11140800 2.95003800
C 2.42634500 -0.50315200 2.58531700
C 1.55865300 2.12861300 3.05286100
C 3.34180100 0.55438000 2.67873800
C 2.92807800 1.87205400 2.89867700
H 1.21527000 3.15266000 3.21854100

H 4.40663400 0.33678400 2.56051300
C 2.91154700 -1.91362700 2.38209700
H 3.19342300 -2.37051700 3.34711100
H 2.14437200 -2.54774800 1.92275600
H 3.79606100 -1.93313000 1.73044100
C -0.86155400 1.40432700 3.08862500
H -1.40404300 1.08265800 2.18937100
H -1.29823300 0.86964900 3.94899900
H -1.03660400 2.47981400 3.21878800
C 3.92066100 3.00625300 2.93479200
H 3.84212000 3.62374700 2.02322700
H 3.73653800 3.67533600 3.79020200
H 4.95550700 2.63971000 3.00123800
N 0.07922900 -1.22614000 2.52601900
C -1.82930600 3.18216700 0.18054500
C -0.40609400 3.25405400 0.18995500
C 0.21122400 4.45192700 0.56318800
C -0.58272400 5.55224100 0.91821000
C -1.98032200 5.46252400 0.90791800
C -2.62320100 4.26769800 0.54000800
C -1.09542800 1.07603900 -0.41093800
C 0.05865200 1.93799200 -0.16264000
H 1.30115700 4.52735600 0.58145100
H -0.10510100 6.49160600 1.20785700
H -2.58217600 6.33000800 1.18948600
H -3.71301500 4.20018900 0.53811700
N -2.21890500 1.88480000 -0.17389600
C -3.56827800 1.42465200 0.03156000
H -3.90638600 1.64974000 1.05925300
H -4.27965500 1.89848800 -0.66655600
H -3.60973800 0.34171600 -0.11692400
C 1.28995900 1.31546900 -0.22307100
H 2.23576700 1.85031000 -0.10350000
N 1.25499800 -0.01038000 -0.42258200
C 2.36630200 -0.77995800 -0.71196400
C 2.18170300 -2.17132500 -0.87841200
C 3.67588800 -0.28489100 -0.87357700
C 3.23578500 -3.02217800 -1.17883100
H 1.18158000 -2.58041800 -0.72835800
C 4.74599500 -1.13811000 -1.15623000
H 3.87058500 0.78360500 -0.78480800
C 4.53880500 -2.51705200 -1.31135900
H 3.07908000 -4.09578100 -1.30119500
H 5.74151700 -0.70781200 -1.26704500
O 5.51876600 -3.42399400 -1.59139300
C 6.83942900 -2.96548200 -1.72762100
H 7.20889400 -2.48593800 -0.80171800
H 7.46069900 -3.84558700 -1.94295400
H 6.94402800 -2.24350100 -2.55886900
C -0.32025500 1.35025600 -2.99721200
C -0.86461500 2.61572200 -3.28475700
C 1.00650200 1.09963200 -3.38950700
C -0.10779700 3.59346300 -3.92964200
H -1.88259600 2.84972000 -2.96796400
C 1.76542600 2.07746800 -4.03409000
H 1.45051000 0.12542900 -3.17532900
C 1.21355300 3.33237700 -4.30686000
H -0.55005000 4.57299900 -4.12918700
H 2.79552100 1.85493800 -4.32528600
H 1.80665700 4.10072600 -4.80894500
C -2.52975000 0.11170800 -2.66399300
H -2.57946000 -0.20224300 -3.72123100
H -3.01308200 -0.67626000 -2.06361100
H -3.13657800 1.02209900 -2.56700900
C -2.62479300 -2.64510800 0.79225200
C -3.88246000 -2.04115800 1.00929300
C -2.36933500 -3.41837800 -0.35430300
C -4.87258600 -2.21023000 0.03894100
C -3.39360100 -3.54544300 -1.30513900
C -4.64212000 -2.94255000 -1.13568200
H -5.84569200 -1.73562500 0.19158000
H -3.20244900 -4.13759000 -2.20435200
C -4.13660000 -1.21266900 2.24137400
H -5.06639300 -0.63661900 2.14049200
H -4.22684100 -1.84225900 3.14109900
H -3.31025300 -0.51156200 2.42689300
C -1.06031200 -4.13353000 -0.57431900
H -0.51338600 -3.70727700 -1.43023800
H -0.40708400 -4.08102200 0.30451200
H -1.24336400 -5.19359500 -0.81017200
C -5.70314600 -3.04017700 -2.20070900
H -5.66369400 -2.16092600 -2.86685900
H -5.56660500 -3.93320100 -2.82802900
H -6.71282900 -3.07411100 -1.76444700

TS5-M-Ph (triplet)

Fe -0.61700400 -0.57621700 -0.31773600

C -1.03778000 0.41094200 -2.23175400
H -0.66742200 -0.64019800 -2.31262800
N -2.17843200 -2.07646000 1.70361200
C -1.06876200 -1.32116700 1.40100400
C -2.24721200 -2.36872000 3.06403500
C -1.15671500 -1.78993200 3.63515600
H -3.04769900 -2.96433900 3.49179800
H -0.81159300 -1.77509900 4.66465100
C 0.71407400 -0.34049700 2.77585000
C 0.54241500 1.03245200 3.01383100
C 1.98097100 -0.92298500 2.62203200
C 1.68904100 1.82969000 3.09509100
C 3.09796000 -0.08648000 2.70833700
C 2.97009700 1.29105700 2.93060500
H 1.57004600 2.90471800 3.24954900
H 4.09051600 -0.52155600 2.57061100
C 2.12158100 -2.39414900 2.34118700
H 1.79229000 -3.00291000 3.20011000
H 1.50562800 -2.68537800 1.47850600
H 3.16311100 -2.65322600 2.10906700
C -0.83349200 1.62817300 3.14327100
H -1.45151100 1.35126100 2.27776700
H -1.34965900 1.26409700 4.04750000
H -0.78482500 2.72384200 3.18570900
C 4.18412500 2.18454100 2.92747300
H 4.02967400 3.08206800 3.54470900
H 5.07765800 1.65868400 3.29664500
H 4.40899500 2.52993700 1.90248100
N -0.45392000 -1.15843600 2.61398800
C -1.27989900 3.49071600 0.17450400
C 0.13317900 3.31104400 0.19422500
C 0.94716500 4.38399500 0.57704800
C 0.35300200 5.60183800 0.93131800
C -1.04089600 5.75733200 0.91302700
C -1.87800700 4.69581500 0.53533100
C -0.91035700 1.29742100 -0.38848900
C 0.37105900 1.93563400 -0.16697900
H 2.03351600 4.27014600 0.60587900
H 0.98374400 6.44325800 1.22899700
H -1.48346500 6.71535700 1.19614500
H -2.96314000 4.81711000 0.52582400
N -1.87897300 2.27830000 -0.18681400
C -3.28578500 2.03329900 0.01878000
H -3.58382800 2.31754500 1.04395100
H -3.91168000 2.60533400 -0.68649900
H -3.48656000 0.96658300 -0.12006600
C 1.54523900 1.18418200 -0.28370500
H 2.52111400 1.66984300 -0.16834900
N 1.43429200 -0.11489500 -0.53534700
C 2.52729500 -0.94797500 -0.73403300
C 2.27327700 -2.26836900 -1.16910000
C 3.87372600 -0.59447200 -0.52533000
C 3.29392500 -3.18485400 -1.37601200
H 1.23409600 -2.56317500 -1.33627200
C 4.90991200 -1.51178500 -0.72368200
H 4.12870000 0.41035100 -0.18939400
C 4.63070700 -2.81841000 -1.14875100
H 3.08360900 -4.20220700 -1.71155600
H 5.93581600 -1.19090700 -0.54186300
O 5.57007700 -3.78209700 -1.36486600
C 6.92187000 -3.46421600 -1.15112700
H 7.11716800 -3.17127800 -0.10254500
H 7.50081900 -4.36933200 -1.37997000
H 7.26390800 -2.64542500 -1.81103600
C -0.12423800 1.26783300 -3.03126800
C -0.44225900 2.61034400 -3.31037300
C 1.11833800 0.78090700 -3.47198600
C 0.45000100 3.43255800 -3.99747200
H -1.38676100 3.02601900 -2.95492400
C 2.01188400 1.60108000 -4.16193300
H 1.39080300 -0.25430200 -3.25798500
C 1.68379400 2.93359500 -4.42772600
H 0.18424600 4.47533200 -4.18925200
H 2.97208100 1.19520000 -4.49102500
H 2.38385600 3.57918800 -4.96382400
C -2.50269900 0.44267200 -2.63643700
H -2.61850300 0.11771400 -3.68549800
H -3.10880200 -0.23085900 -2.01090000
H -2.93130800 1.45160000 -2.56520700
C -3.17166400 -2.32654200 0.70767500
C -4.37844900 -1.60609400 0.75265900
C -2.87369700 -3.20726300 -0.34882800
C -5.28281300 -1.77052100 -0.30373500
C -3.80656700 -3.33220400 -1.38357100
C -5.01022000 -2.61487100 -1.38655200
H -6.21969300 -1.20703000 -0.28663600
H -3.58451800 -4.00892900 -2.21325400
C -4.68329500 -0.67061300 1.89414500

H -5.49961900 0.01612700 1.63039500
H -4.99145800 -1.22266100 2.79734800
H -3.80217600 -0.07472200 2.17149400
C -1.58248900 -3.98330800 -0.37335600
H -0.72765400 -3.32789800 -0.60963900
H -1.36012100 -4.42740800 0.60844300
H -1.61617200 -4.78272000 -1.12615300
C -5.96722000 -2.72543800 -2.54541700
H -5.65327700 -2.06593300 -3.37289800
H -6.00242500 -3.75103400 -2.94343500
H -6.98751800 -2.43146300 -2.25857900

TS5-M-Ph (quintet)

Fe -0.38485000 -0.37666000 0.23582400
C -1.85258200 0.71876700 -1.44069500
H -1.10218900 0.01367400 -1.84234400
N -0.65724700 -3.45427400 0.31437700
C 0.11195600 -2.34102900 0.47425400
C -0.03405200 -4.58626700 0.82764700
C 1.16291700 -4.17195100 1.32539900
H -0.49761400 -5.56750400 0.79093000
H 1.96123000 -4.71803800 1.81810000
C 2.34682600 -1.96490900 1.45777500
C 2.18647900 -0.99513500 2.46466900
C 3.55096400 -2.11342500 0.75047500
C 3.27461700 -0.16246500 2.74044100
C 4.61366100 -1.26400200 1.07518800
C 4.49136800 -0.27778100 2.05740000
H 3.16164500 0.60816400 3.50784200
H 5.54511700 -1.34826700 0.51134600
C 3.69337700 -3.12745000 -0.35413700
H 3.80088300 -4.15305000 0.03675200
H 2.81289400 -3.12372200 -1.01406400
H 4.57853600 -2.90698800 -0.96514500
C 0.88855500 -0.81435700 3.20828400
H 0.20821200 -0.13701600 2.65904300
H 0.35770400 -1.76612800 3.35491300
H 1.06622700 -0.35934500 4.19298900
C 5.63011800 0.65697600 2.36809400
H 5.96490600 0.54692800 3.41296300
H 6.49193800 0.47256700 1.71121600
H 5.32044700 1.70494700 2.23114300
N 1.23485200 -2.80278100 1.10214200
C -3.00388200 2.68455700 1.57922200
C -1.71786700 3.26763000 1.40198700
C -1.51682000 4.59844000 1.77800300
C -2.58035900 5.32285400 2.33427700
C -3.83316200 4.72583500 2.52461300
C -4.05962200 3.39080200 2.15268000
C -1.70678400 1.03604800 0.62427100
C -0.87615800 2.23408000 0.83015100
H -0.54059400 5.07001700 1.64114800
H -2.42890600 6.36447000 2.62820100
H -4.64774300 5.30414900 2.96781400
H -5.03784800 2.92898600 2.30096800
N -2.96953200 1.36103000 1.12762400
C -3.97761900 0.40314300 1.50822100
H -4.05328500 0.31440400 2.60942900
H -4.97208000 0.68518300 1.12317900
H -3.71849300 -0.57554100 1.09268500
C 0.45349100 2.24246900 0.43878200
H 1.03872500 3.16846600 0.46910200
N 0.99170700 1.10365200 -0.05147100
C 2.27163800 1.04216900 -0.57987700
C 2.57136200 0.01251900 -1.50268800
C 3.31529900 1.93044800 -0.25493300
C 3.83576000 -0.12974500 -2.05430200
H 1.77495200 -0.68404200 -1.77646900
C 4.59354900 1.78991300 -0.80266500
H 3.13906300 2.73120800 0.46454200
C 4.87062500 0.74750900 -1.69563800
H 4.05525300 -0.92853100 -2.76529600
H 5.37185300 2.49237400 -0.50439400
O 6.09615400 0.50129300 -2.24934100
C 7.15477900 1.37117700 -1.94217300
H 7.38691200 1.37635600 -0.86023400
H 8.03488300 1.00845600 -2.49078100
H 6.94185100 2.41019500 -2.25530300
C -1.61095900 2.06439800 -1.98750800
C -2.57414800 3.08930700 -1.87779600
C -0.36909500 2.39853100 -2.56247700
C -2.30092900 4.38561700 -2.31024400
H -3.53971500 2.87592300 -1.41716600
C -0.09638000 3.69450000 -2.99996700
H 0.39798000 1.62770800 -2.65604800
C -1.05963800 4.70000100 -2.87382200
H -3.06202400 5.16191700 -2.19607000

H 0.87887300 3.91959700 -3.44004900
H -0.84607900 5.71777700 -3.20963000
C -3.23672000 0.13314300 -1.61285500
H -3.49152000 0.02266600 -2.68271100
H -3.30881100 -0.86036500 -1.15215500
H -4.01066000 0.76828900 -1.15975600
C -1.96521200 -3.42361500 -0.27111600
C -3.07832000 -3.30076400 0.57532400
C -2.08735500 -3.49532100 -1.66786200
C -4.34660600 -3.26820400 -0.01600000
C -3.37543000 -3.46264100 -2.21158000
C -4.51472800 -3.34685700 -1.40440100
H -5.22534800 -3.16539400 0.62603100
H -3.49052500 -3.50997400 -3.29758500
C -2.89705600 -3.16782300 2.06469700
H -3.86459800 -3.05109700 2.57115800
H -2.38700900 -4.04419300 2.49538500
H -2.27770100 -2.28746000 2.30242700
C -0.86296700 -3.55814300 -2.54134400
H -0.28654700 -2.62175100 -2.46101600
H -0.18685000 -4.37343100 -2.23941800
H -1.13489000 -3.70387600 -3.59543100
C -5.88427400 -3.24522900 -2.02340100
H -6.09531800 -2.20294300 -2.31879300
H -5.96523900 -3.86341900 -2.93015500
H -6.67180800 -3.55521900 -1.32094300

TS5-anti/M-Ph (singlet)

Fe -0.01494000 -0.74443800 -0.31469100
C -1.17605700 -0.25616200 -2.04400200
H -0.37445500 -0.97824100 -2.31493800
N -0.03619800 -2.12501800 2.17759200
C 0.55770400 -1.11717600 1.43834500
C 0.47294600 -2.18569000 3.47304000
C 1.41170600 -1.20706900 3.55810500
H 0.13829400 -2.92076200 4.19846500
H 2.06103800 -0.90963300 4.37598900
C 2.18420400 0.63891700 2.05191400
C 1.50752700 1.86563600 2.19932700
C 3.51696500 0.56942500 1.62711200
C 2.19048000 3.03509700 1.86193200
C 4.15824600 1.77018200 1.29196200
C 3.50946000 3.00499600 1.38733100
H 1.66516700 3.99038000 1.93997900
H 5.18843200 1.72938800 0.92863200
C 4.25466500 -0.73951900 1.53773500
H 4.82854000 -0.92707000 2.46240900
H 3.57067900 -1.58374700 1.39165200
H 4.96347100 -0.73234500 0.69825200
C 0.08976200 1.91121600 2.70187300
H -0.56336700 1.29261800 2.07272300
H 0.01972200 1.52622700 3.73338000
H -0.30033100 2.93691000 2.68438400
C 4.19225200 4.28117700 0.96481900
H 3.73507900 4.68473800 0.04491800
H 4.09824400 5.06225000 1.73614600
H 5.26229400 4.12328200 0.76546600
N 1.45381200 -0.56623500 2.32113100
C -1.98238400 2.84641400 -0.00276300
C -0.68360400 3.19888000 -0.47625000
C -0.32567700 4.55050200 -0.53006800
C -1.25313100 5.52367400 -0.12944000
C -2.52653700 5.15874800 0.32573100
C -2.90641100 3.80736100 0.39770400
C -0.92572300 0.84973900 -0.44642700
C -0.00834900 1.95259900 -0.73918400
H 0.66849300 4.84454900 -0.87621800
H -0.97843600 6.58065400 -0.17233000
H -3.23543800 5.93178200 0.63221100
H -3.89691700 3.52344000 0.75757300
N -2.09762500 1.45441300 0.02013800
C -3.18470800 0.79391400 0.69338300
H -3.20728100 1.06668200 1.76402000
H -4.15527600 1.05839200 0.24832100
H -3.05588100 -0.28980800 0.61225100
C 1.28263500 1.57291400 -1.05844800
H 2.06586100 2.29074800 -1.31871800
N 1.53173600 0.25598700 -1.00077000
C 2.71839100 -0.32441300 -1.41682600
C 2.82590800 -1.73211500 -1.35076200
C 3.84063200 0.37637800 -1.90293400
C 3.97452900 -2.40338400 -1.74508900
H 1.98501700 -2.29522600 -0.94544600
C 5.00704900 -0.29034200 -2.28728300
H 3.81698100 1.46303200 -1.98028000
C 5.08780700 -1.68896000 -2.21387200
H 4.04181500 -3.49156800 -1.68563400

H 5.84920700 0.29889100 -2.65079800
O 6.18079400 -2.42487800 -2.56861400
C 7.32074300 -1.75110600 -3.03649300
H 7.73239800 -1.05779800 -2.27900600
H 8.07420600 -2.51866500 -3.26040300
H 7.11341500 -1.17570300 -3.95801100
H -1.04483000 0.56876700 -2.75177500
C -3.76460000 -0.08811400 -2.21066500
C -5.00477100 -0.65472900 -1.87502500
C -3.73106500 1.26808600 -2.56066900
C -6.17407500 0.10756600 -1.87697400
H -5.04657200 -1.70970500 -1.59069900
C -4.89633000 2.03932100 -2.55350500
H -2.78275600 1.74590600 -2.80634000
C -6.12311800 1.46464800 -2.21090800
H -7.12668300 -0.35548800 -1.60629300
H -4.83837500 3.10091200 -2.80509200
H -7.03272500 2.07006100 -2.20123200
C -2.52346900 -0.95567800 -2.17770800
H -2.51943300 -1.55734000 -3.10889900
H -2.64456400 -1.70115900 -1.37225900
C -1.20926700 -2.75818900 1.66257600
C -2.46538700 -2.37179800 2.17687700
C -1.10481800 -3.65579100 0.58303300
C -3.61557700 -2.88890500 1.57384900
C -2.28924400 -4.13624700 0.00667300
C -3.55042400 -3.76155900 0.47905700
H -4.59276500 -2.57605900 1.95086000
H -2.21719500 -4.82355000 -0.84104400
C -2.56683200 -1.39648200 3.31999700
H -3.59374300 -1.02018500 3.42133600
H -2.28382000 -1.86336200 4.27706200
H -1.89418100 -0.53968600 3.17327100
C 0.22399900 -4.12217900 0.04506900
H 0.42605000 -3.68322500 -0.94540000
H 1.05543800 -3.85333300 0.70725600
H 0.21872100 -5.21502600 -0.08788700
C -4.80432600 -4.29525000 -0.16435600
H -5.67972200 -3.67951500 0.08802600
H -4.70990300 -4.32528400 -1.26068400
H -5.01424500 -5.32494300 0.17230700

TS5-anti/M-Ph (triplet)

Fe -0.01426100 0.82432800 -0.36054100
C 1.14331000 0.25994600 -2.05608600
H 0.61112700 1.22127400 -2.27957400
N -0.26439200 2.86614100 1.77985700
C -0.55415900 1.60040800 1.32676000
C -0.68295200 3.05573400 3.09460600
C -1.25695600 1.88602500 3.48403700
H -0.54547500 3.99658800 3.61864800
H -1.72280600 1.59407500 4.42049000
C -1.60099400 -0.35294900 2.40284600
C -0.68813600 -1.34598000 2.79300200
C -2.90033700 -0.65636800 1.96976000
C -1.10632400 -2.67913300 2.73023500
C -3.27446900 -2.00330000 1.92516700
C -2.38753300 -3.02602300 2.28683200
H -0.39640500 -3.46479100 2.99955600
H -4.27869100 -2.25704200 1.57768100
C -3.84351100 0.43640900 1.54565100
H -4.11385900 1.08782600 2.39365900
H -3.38048500 1.07362700 0.77877800
H -4.76491300 0.01832500 1.11930300
C 0.70051000 -0.98405300 3.24576500
H 1.18207200 -0.32142800 2.51389200
H 0.68350900 -0.45242200 4.21217600
H 1.32461000 -1.88079700 3.35183000
C -2.78250700 -4.47409000 2.14623300
H -2.46455200 -4.87014900 1.16529000
H -2.30672700 -5.10093400 2.91544500
H -3.87257100 -4.60754600 2.21557800
N -1.17158800 1.01548100 2.40279400
C 2.68847000 -2.15082800 0.58534000
C 1.47474800 -2.83684700 0.28633200
C 1.40079700 -4.21439400 0.52611200
C 2.51923200 -4.87985900 1.04417800
C 3.70456200 -4.18598100 1.32962900
C 3.80119900 -2.80479100 1.10477900
C 1.23966300 -0.55923000 -0.19220100
C 0.55855700 -1.83682900 -0.20775300
H 0.48073200 -4.76437300 0.31271800
H 2.46836300 -5.95579700 1.22945800
H 4.56587900 -4.72649500 1.72926700
H 4.72646700 -2.26599700 1.31610800
N 2.51586800 -0.79537100 0.29415100
C 3.45948200 0.21930700 0.68326100

H 3.66678400 0.16940600 1.76729200
H 4.41394400 0.10897600 0.14362400
H 3.02913100 1.20193700 0.45514400
C -0.77253600 -1.87241300 -0.64106800
H -1.31016900 -2.82706000 -0.70309600
N -1.35076200 -0.72314400 -0.97321100
C -2.65568100 -0.63142900 -1.44152500
C -3.07007200 0.60988000 -1.97577000
C -3.61524400 -1.65993600 -1.39824800
C -4.36368500 0.81981700 -2.42914700
H -2.33434800 1.41675000 -2.02625000
C -4.92410500 -1.45772300 -1.84801200
H -3.35184200 -2.63506300 -0.98912600
C -5.31323800 -0.21400800 -2.36420700
H -4.67211400 1.78333000 -2.83964600
H -5.63357000 -2.28316100 -1.78531900
O -6.56388200 0.08143400 -2.81879500
C -7.54900600 -0.91930400 -2.77655800
H -7.74633500 -1.26239600 -1.74364900
H -8.46818100 -0.47748400 -3.18487200
H -7.27289900 -1.79799600 -3.38862700
H 0.62284700 -0.51507800 -2.63259200
C 3.44457900 -0.84072600 -2.49665000
C 4.83758000 -0.73420000 -2.35665400
C 2.89580000 -2.12118800 -2.65338000
C 5.65482100 -1.86684100 -2.35066200
H 5.28780000 0.25695100 -2.24399100
C 3.70775400 -3.25714100 -2.64071500
H 1.81698100 -2.24240000 -2.74592800
C 5.09005800 -3.13767800 -2.48638600
H 6.73582200 -1.75622700 -2.23063600
H 3.25014600 -4.24504200 -2.72965700
H 5.72177100 -4.02867600 -2.46197800
C 2.59245600 0.41508500 -2.49163900
H 2.59865500 0.82270400 -3.52234100
H 3.09354400 1.18570300 -1.88386000
C 0.48708900 3.78017900 0.97958700
C 1.84811600 3.97517300 1.26618100
C -0.13892000 4.40205500 -0.11730000
C 2.58633300 4.81001100 0.41659000
C 0.64048000 5.22178800 -0.93846400
C 2.00460300 5.43548400 -0.69145300
H 3.64908100 4.96416000 0.62224100
H 0.16880700 5.70917900 -1.79645000
C 2.49426900 3.29753000 2.44665800
H 3.58964400 3.31602400 2.35983000
H 2.22578600 3.79529200 3.39329300
H 2.16824500 2.25147100 2.53517600
C -1.60261500 4.18071100 -0.39590500
H -1.79829000 3.14169000 -0.70554200
H -2.21199200 4.34780800 0.50569500
H -1.96007800 4.84933400 -1.19092400
C 2.81416900 6.31987700 -1.60529800
H 2.79587400 5.94292000 -2.64102500
H 2.40831600 7.34470700 -1.62902100
H 3.86437700 6.37951600 -1.28552200

TS5-anti/M-Ph (quintet)

Fe -0.17916400 0.60408900 0.17753900
C 1.12456100 1.48449700 -1.31847400
H 0.09587600 1.94635400 -1.30927200
N -2.81430400 1.44833400 1.65956800
C -1.79641600 0.57959400 1.39848600
C -3.58215200 1.02537100 2.74257000
C -3.02620700 -0.13965900 3.17587600
H -4.43698500 1.58947400 3.10250400
H -3.29744700 -0.80195300 3.99240500
C -1.04163300 -1.51282400 2.38468500
C 0.25407800 -1.31112100 2.89336600
C -1.43465400 -2.71935400 1.78813100
C 1.19118900 -2.32895700 2.71068900
C -0.46045300 -3.71377100 1.63854500
C 0.86144100 -3.52279400 2.05628500
H 2.21683100 -2.17041200 3.05316000
H -0.73848900 -4.64797100 1.14423500
C -2.84554900 -2.92012300 1.30385300
H -3.55890400 -2.93894300 2.14486800
H -3.15429000 -2.11070800 0.62708900
H -2.94061600 -3.86350000 0.75244700
C 0.62436900 -0.02788000 3.58867500
H 0.61837400 0.81609300 2.88279800
H -0.08516300 0.21492900 4.39584500
H 1.63248900 -0.09528300 4.01901800
C 1.93030500 -4.54559400 1.77648900
H 2.66645600 -4.13537300 1.06480900
H 2.48339500 -4.81254700 2.69134000
H 1.51091300 -5.46691000 1.34674000

N -1.94236400 -0.39265800 2.34705900
C 3.98958500 -0.27225900 0.46876900
C 3.44821100 -1.34389500 -0.30085700
C 4.29524400 -2.38350500 -0.70212600
C 5.64675200 -2.35022100 -0.33144400
C 6.15622400 -1.29387700 0.43609900
C 5.32818500 -0.23974900 0.85016200
C 1.76556100 0.22538700 0.18050900
C 2.04187500 -1.04833200 -0.46932500
H 3.90862700 -3.21139700 -1.30237900
H 6.31206900 -3.15900100 -0.64411000
H 7.21358200 -1.28508500 0.71162700
H 5.73112600 0.59167200 1.43083800
N 2.97268100 0.64324500 0.74259500
C 3.14216800 1.72758400 1.67217200
H 3.43697300 1.36157300 2.67312100
H 3.91494200 2.43363500 1.32554000
H 2.19270400 2.26865500 1.76910400
C 0.98549600 -1.71178900 -1.07963300
H 1.14327800 -2.64749400 -1.63077600
N -0.23317000 -1.14803900 -1.01306900
C -1.36444100 -1.75607600 -1.52763500
C -2.51092900 -0.95691700 -1.76171300
C -1.48329500 -3.13360200 -1.80587400
C -3.69869500 -1.50072300 -2.23075900
H -2.44110300 0.11542500 -1.56517600
C -2.67520000 -3.68656100 -2.28471600
H -0.64065000 -3.79828400 -1.61246300
C -3.79865400 -2.87661400 -2.49688900
H -4.57503600 -0.87452300 -2.40984900
H -2.71639500 -4.75971800 -2.47357200
O -5.00726300 -3.32214100 -2.95067900
C -5.15141400 -4.68848400 -3.23931300
H -4.98952700 -5.32058400 -2.34571000
H -6.18187800 -4.83256600 -3.59231200
H -4.45361100 -5.02124400 -4.03039100
H 1.15591100 0.83025500 -2.19722900
C 3.56883400 2.22368800 -1.68052000
C 4.61908900 3.03549200 -1.22367900
C 3.89615500 1.07921500 -2.42082600
C 5.95224800 2.70860200 -1.48015900
H 4.38545900 3.93873400 -0.65153500
C 5.22800400 0.74626900 -2.67681600
H 3.10757100 0.41559800 -2.77437400
C 6.26267000 1.55593500 -2.20657500
H 6.75154100 3.35253000 -1.10378900
H 5.45616100 -0.16933600 -3.22672800
H 7.30415300 1.28376400 -2.39262800
C 2.13305100 2.61636400 -1.38821400
H 1.81416200 3.32516300 -2.17942000
H 2.10828900 3.20709600 -0.45754000
C -3.05390400 2.61088700 0.85840800
C -2.16094300 3.69547900 0.94354100
C -4.14348400 2.61057700 -0.02773300
C -2.38996400 4.79724100 0.11382700
C -4.33165200 3.74024900 -0.83475800
C -3.46822500 4.83975900 -0.78107700
H -1.70492100 5.64803500 0.16798200
H -5.17149100 3.75209300 -1.53443000
C -0.97782500 3.66556200 1.87632700
H -0.56413100 4.67329500 2.01972700
H -1.24178200 3.24826200 2.85932900
H -0.17396200 3.02543300 1.47228600
C -5.06791800 1.42453100 -0.12366900
H -4.50543600 0.48032100 -0.16481200
H -5.73965400 1.35959300 0.74808300
H -5.69623000 1.49094700 -1.02258600
C -3.67393400 6.04021500 -1.66922300
H -2.83328300 6.15960500 -2.37274300
H -4.59713700 5.95382700 -2.25979600
H -3.73283900 6.96821800 -1.07766600

4a

C 2.51821500 0.15040700 -0.00070800
C 1.35053500 0.80821100 -0.00172100
H 3.48520500 0.66733100 -0.00162400
H 2.56540100 -0.94472100 0.00118600
H 1.39239600 1.90780300 -0.00356000
Si -0.34775900 -0.00034600 -0.00005200
C -1.29317600 0.55270300 -1.53922900
H -0.77355300 0.23718100 -2.45845300
H -2.30774800 0.12095000 -1.55590300
H -1.39479500 1.65007100 -1.56961800
C -0.15918100 -1.87710900 0.00322000
H 0.38674800 -2.22464400 0.89528200
H -1.14801900 -2.36436600 0.00431600
H 0.38627300 -2.22792900 -0.88786600

C -1.29182400 0.55821200 1.53796000
H -1.39259400 1.65576900 1.56478800
H -2.30670100 0.12730700 1.55733200
H -0.77139700 0.24555000 2.45771400

TS3-M-SiMe$_3$ (singlet)

N -0.74741500 -0.54991700 2.21170200
C -2.05015800 0.02057600 2.00481200
C -2.25532300 1.39549500 2.15604600
C -3.11578900 -0.86582800 1.74704400
C -3.55872400 1.88505200 1.97224600
C -4.63374500 1.04488000 1.67965500
H -3.73046900 2.96103500 2.06986200
C 0.15537000 -0.93342200 1.24388900
Fe 0.01526100 -0.69085900 -0.77495400
C 2.40061700 -0.71772400 -1.44359600
C 0.79990400 -1.63394900 3.34508200
H 1.43892100 -2.12967100 4.06890100
C -0.36836500 -0.96385400 3.48357800
H -0.97617400 -0.74584200 4.35615400
C -4.39258300 -0.33313900 1.57800100
H -5.22041900 -1.00629600 1.34206800
C 1.42842100 -1.23403800 -2.30402200
H 1.13804700 -2.29851500 -2.21980200
C -2.88910100 -2.35333200 1.70599300
H -1.99570000 -2.60770500 1.12282900
H -2.73951900 -2.75592400 2.72267100
H -3.75382200 -2.86572600 1.26298700
C -1.15132400 2.34528100 2.53624600
H -1.33997600 2.76986100 3.53673200
H -0.16990600 1.86089300 2.54883700
H -1.09062200 3.18569900 1.82948800
C -6.02782500 1.58497200 1.49565500
H -6.03163300 2.68307900 1.43206200
H -6.48158000 1.18340300 0.57718500
H -6.67815100 1.29492600 2.33854400
N 1.10555400 -1.60703200 1.98796200
C -4.00756300 -1.38521600 -1.74845800
C -3.93890500 0.03276300 -1.60777300
C -5.09806300 0.78680500 -1.82536500
C -6.28976300 0.13154500 -2.15711000
C -6.33932300 -1.26613800 -2.27731800
C -5.18998600 -2.04421500 -2.07639000
C -1.84739800 -0.91456800 -1.14245100
C -2.57536200 0.31516500 -1.23617800
H -5.07609000 1.87480800 -1.72746200
H -7.19744000 0.71691800 -2.32483600
H -7.28156000 -1.75518300 -2.53552700
H -5.22728900 -3.13121000 -2.17509600
H -0.38189200 -0.67006600 -2.25391600
N -2.73663700 -1.91261400 -1.48162000
C -2.42253800 -3.30975300 -1.63416900
H -2.95832100 -3.93692400 -0.90093900
H -2.68511600 -3.66315800 -2.64551000
H -1.34483800 -3.44802700 -1.49044200
C -1.80958100 1.45411600 -0.97544000
H -2.23743000 2.46211900 -0.95412100
N -0.52402800 1.25576700 -0.71153500
C 0.31721500 2.34047300 -0.43169500
C 1.43472500 2.15136100 0.40524200
C 0.09452900 3.63526100 -0.93074400
C 2.24850800 3.21208000 0.77340300
H 1.63968100 1.14274900 0.75627100
C 0.91713400 4.70896100 -0.57463800
H -0.72010000 3.81293000 -1.63398300
C 1.99951300 4.50765100 0.29239100
H 3.10262800 3.06622900 1.43708200
H 0.71041700 5.69243800 -0.99632700
O 2.85497600 5.48258000 0.70488000
C 2.66419300 6.79208900 0.22991500
H 1.67909900 7.19888500 0.52459000
H 3.45268200 7.41025800 0.68008200
H 2.75083400 6.84809300 -0.87087800
H 1.31414100 -0.85088800 -3.32290600
C 2.27304200 -2.24655600 1.46865500
C 3.53358400 -1.67985300 1.71858900
C 2.12429600 -3.42691000 0.72179900
C 4.65402700 -2.30516600 1.15995800
C 3.27127300 -4.00835600 0.17286700
C 4.54325800 -3.45492200 0.36872100
H 5.63995300 -1.86396100 1.32753100
H 3.16751800 -4.91435300 -0.43012300
C 0.76163100 -4.02707300 0.50342100
H 0.26011200 -4.24400600 1.46019300
H 0.09879800 -3.32971300 -0.03095400
H 0.82421800 -4.95717400 -0.07762400
C 3.67533600 -0.41605900 2.52499300

H 2.92763400 0.33168100 2.22536400
H 3.52617900 -0.60030800 3.60146600
H 4.67309200 0.02206900 2.39240800
C 5.75665400 -4.05183100 -0.29560100
H 6.66403800 -3.90355500 0.30886400
H 5.63218300 -5.13007500 -0.47526300
H 5.93320300 -3.57319200 -1.27453300
Si 3.62039700 0.57152000 -2.02287300
C 2.82357400 2.06359700 -2.85785400
H 3.56167200 2.58255800 -3.49185800
H 1.98798800 1.74733800 -3.50345900
H 2.42643900 2.78385700 -2.13104600
C 4.70461000 -0.28681800 -3.32915900
H 4.09885500 -0.60831000 -4.19262000
H 5.49971500 0.38126600 -3.70218800
H 5.18439000 -1.18575400 -2.90672000
C 4.79261400 1.08330600 -0.63164400
H 5.52437200 1.82271400 -0.99682800
H 4.26335900 1.52703700 0.22064500
H 5.35166800 0.20418000 -0.27267000
H 2.76608800 -1.39076300 -0.67234100

TS3-M-$SiMe_3$ (triplet)

N -0.59852400 -1.12691700 2.21998600
C -1.90218900 -0.54069300 2.09911200
C -2.10715900 0.79840200 2.45328900
C -2.96007100 -1.36959000 1.67907700
C -3.40803500 1.31048000 2.34523900
C -4.48422400 0.52032800 1.93156000
H -3.57969400 2.36036000 2.60034800
C 0.35925400 -1.17662600 1.22837200
Fe 0.19317400 -0.48082400 -0.60690400
C 1.99230900 -0.57761100 -1.68666800
C 0.99148000 -2.38070600 3.08481500
H 1.64605500 -3.01150300 3.67791200
C -0.22835200 -1.85179000 3.34619300
H -0.87156100 -1.91991900 4.21811900
C -4.24057200 -0.81997000 1.60361500
H -5.06433500 -1.44349300 1.24832000
C 0.92739400 -0.49598400 -2.66144600
H 0.70072000 -1.38749100 -3.26412600
C -2.71267700 -2.81335800 1.33283300
H -1.81510900 -2.91840800 0.70887800
H -2.55178400 -3.42061200 2.23986800
H -3.56972400 -3.23428400 0.78939300
C -0.99254300 1.66642200 2.97139600
H -1.05007400 1.75446800 4.07021200
H -0.00663200 1.26210900 2.71839000
H -1.05536700 2.68084700 2.55382700
C -5.87934000 1.08578500 1.85933400
H -5.86670900 2.15944600 1.61705000
H -6.47832800 0.56792400 1.09698900
H -6.39942000 0.97526300 2.82697300
N 1.33828300 -1.96112900 1.80262600
C -3.83366500 -1.51078400 -1.89550800
C -3.91451400 -0.14051200 -1.51084100
C -5.16645000 0.48961500 -1.50885300
C -6.29955700 -0.24383500 -1.86805800
C -6.20140000 -1.59937300 -2.23224200
C -4.96345000 -2.25003000 -2.25506500
C -1.71196100 -0.87036000 -1.33572100
C -2.56972600 0.25493500 -1.17616100
H -5.25738000 1.53811100 -1.21670500
H -7.27920600 0.24049000 -1.86538700
H -7.10364300 -2.15136400 -2.50609700
H -4.88794600 -3.30041300 -2.54362300
H -0.54470000 -0.58631800 -2.09903600
N -2.50329400 -1.90671200 -1.80547200
C -2.03943400 -3.22390500 -2.15957000
H -2.52066700 -4.00000200 -1.54016800
H -2.24940600 -3.44962600 -3.21873100
H -0.95689900 -3.27200900 -1.99705500
C -2.01340100 1.49580800 -0.78424800
H -2.65550100 2.38425600 -0.69138100
N -0.72878100 1.55751900 -0.52875600
C -0.08716900 2.76986800 -0.27116700
C 1.07459600 2.74576500 0.52557700
C -0.48833300 4.00615800 -0.80402100
C 1.79258900 3.89949500 0.79563900
H 1.39460100 1.78015200 0.91556800
C 0.22727200 5.17789800 -0.53606300
H -1.35030300 4.05397200 -1.47204700
C 1.37405600 5.13384900 0.26941600
H 2.69785900 3.87389100 1.40475500
H -0.10852400 6.11414000 -0.98166300
O 2.14169100 6.21320500 0.57946900
C 1.78200000 7.47045900 0.06246300

H 0.77935000 7.78627700 0.40524500
H 2.52617700 8.18895600 0.43178300
H 1.79343500 7.48025600 -1.04298700
H 0.79281000 0.43247600 -3.22963600
C 2.57762000 -2.34126300 1.19743300
C 3.76401400 -1.74442000 1.64781300
C 2.57955500 -3.34093500 0.21018500
C 4.96754200 -2.14379900 1.05296100
C 3.80302300 -3.70236200 -0.36050300
C 5.00742300 -3.10703800 0.03905100
H 5.89884200 -1.67690300 1.38481500
H 3.81544300 -4.46619600 -1.14275300
C 1.28912100 -3.96934200 -0.23618900
H 0.72506000 -4.38397600 0.61440600
H 0.63808500 -3.20899400 -0.69604200
H 1.46552100 -4.77146900 -0.96596600
C 3.74338100 -0.70815600 2.74066200
H 2.90658300 -0.00746100 2.60908100
H 3.61804400 -1.17326100 3.73267000
H 4.67817800 -0.13224000 2.75479100
C 6.30647700 -3.46753400 -0.63444700
H 6.30729100 -4.51211300 -0.98049600
H 6.47399500 -2.82925900 -1.51955000
H 7.16528800 -3.32505800 0.03831800
Si 3.33017900 0.73462600 -1.74504200
C 2.70215800 2.30135200 -2.59779900
H 3.48181200 3.08024400 -2.56940600
H 2.46497200 2.10548500 -3.65616200
H 1.80375700 2.71105300 -2.11540200
C 4.73685300 0.02097600 -2.80579300
H 4.38490200 -0.22320300 -3.82142300
H 5.58027300 0.72667300 -2.89521500
H 5.11951500 -0.90804900 -2.35030300
C 4.14449600 1.19607000 -0.09966900
H 4.70499100 2.13794700 -0.21989400
H 3.42561700 1.33487500 0.71619000
H 4.85660900 0.41523100 0.20210600
H 2.41443300 -1.58173400 -1.55511600

TS3-M-SiMe$_3$ (quintet)

N -0.40017200 -0.24013600 2.54874500
C -1.71798700 0.24551500 2.23636400
C -1.94377700 1.62907200 2.17864100
C -2.73840000 -0.69338900 2.01140100
C -3.23393800 2.05937400 1.84931700
C -4.27985000 1.15790600 1.61372300
H -3.42336100 3.13406100 1.77547000
C 0.52890500 -0.60124800 1.62089800
Fe 0.27135600 -0.70227900 -0.50424600
C 1.77336000 -1.29890200 -1.96279900
C 1.32278700 -0.93787800 3.73144800
H 2.04744400 -1.23960400 4.48161700
C 0.06318000 -0.43663500 3.84281800
H -0.54502900 -0.20501900 4.71205200
C -4.01537900 -0.21278800 1.70621600
H -4.81376300 -0.92861300 1.50048400
C 0.67965600 -1.72338300 -2.79769200
H 0.47201800 -2.79458100 -2.92460000
C -2.46284000 -2.17050300 2.10055100
H -1.63840100 -2.45213600 1.43078900
H -2.17500400 -2.46542400 3.12370800
H -3.35053300 -2.74830800 1.81069600
C -0.85854300 2.62439900 2.49250600
H -0.86421300 2.87988000 3.56679900
H 0.13720900 2.23936900 2.24567600
H -1.00158200 3.55343000 1.92393500
C -5.66072800 1.65922500 1.27687400
H -5.62165300 2.43882300 0.49946600
H -6.30262000 0.84678700 0.91026100
H -6.14486000 2.10900100 2.16074400
N 1.58291900 -1.03258800 2.36863000
C -3.77966000 -2.29114500 -1.35717900
C -3.86887300 -0.87067500 -1.46298600
C -5.12833300 -0.27776900 -1.60401100
C -6.27125400 -1.08693000 -1.61897300
C -6.16633700 -2.48188000 -1.50210800
C -4.91680400 -3.10424100 -1.37428300
C -1.62933600 -1.49950900 -1.16013000
C -2.51859100 -0.37322000 -1.33020700
H -5.22077900 0.80720700 -1.68984100
H -7.25745100 -0.62800600 -1.72424800
H -7.07091600 -3.09487100 -1.51712700
H -4.83947500 -4.19017100 -1.29041300
H -0.69600100 -1.56714600 -2.02018000
N -2.44900800 -2.63838300 -1.19246700
C -1.98537200 -3.99012000 -1.04142900
H -2.49327300 -4.49937900 -0.20257300

H -2.15486500 -4.58862000 -1.95431700
H -0.90817400 -3.97275600 -0.83159100
C -2.02208800 0.92641900 -1.21656300
H -2.71377600 1.77604100 -1.26649500
N -0.72351500 1.11695000 -0.97640300
C -0.18280700 2.38466900 -0.79961800
C 1.11618500 2.47586300 -0.24838400
C -0.81730100 3.59787700 -1.13294100
C 1.74099200 3.69296800 -0.02957400
H 1.62008400 1.54627400 0.01607200
C -0.20052900 4.83215100 -0.90422800
H -1.80475600 3.59113700 -1.59571000
C 1.08483700 4.89380400 -0.34675100
H 2.74677900 3.74362400 0.39259900
H -0.73218600 5.74232200 -1.18255600
O 1.76701000 6.04628200 -0.09141800
C 1.15730600 7.27244400 -0.40514200
H 0.21646300 7.42266500 0.15686500
H 1.86791600 8.06164000 -0.12392200
H 0.93565000 7.36018000 -1.48500900
H 0.38563100 -1.11446900 -3.66122500
C 2.79945100 -1.54654800 1.81091800
C 3.95306500 -0.74811800 1.84767500
C 2.79717700 -2.83391000 1.24811600
C 5.13148600 -1.27844000 1.31185700
C 3.99511600 -3.31079300 0.70552900
C 5.17081400 -2.54981700 0.72690700
H 6.03720200 -0.66667000 1.32637200
H 4.00895700 -4.30598800 0.25319600
C 1.53887000 -3.65859900 1.19841400
H 1.00939500 -3.65705200 2.16324100
H 0.83417000 -3.24730300 0.45528300
H 1.75870300 -4.69823100 0.91944800
C 3.91145400 0.64783800 2.41272600
H 3.05183100 1.20979900 2.01642100
H 3.80955900 0.64534600 3.51044400
H 4.82748500 1.19839600 2.15997500
C 6.43430800 -3.06169100 0.08499300
H 6.49926400 -2.72146600 -0.96334400
H 7.33122700 -2.69160600 0.60397400
H 6.46825900 -4.16118800 0.07546800
Si 2.93884000 -0.01191700 -2.66702000
C 1.98266500 1.35139000 -3.55597000
H 2.68511800 2.08049000 -3.99279800
H 1.37952400 0.93778700 -4.38057200
H 1.30744200 1.89298100 -2.87981900
C 4.04879700 -0.86550100 -3.95194200
H 3.44072100 -1.31740200 -4.75281300
H 4.75416400 -0.15623300 -4.41782300
H 4.63905800 -1.67280000 -3.48587200
C 4.11581100 0.72822200 -1.38638100
H 4.74730500 1.49584700 -1.86396500
H 3.59628500 1.20043700 -0.54233100
H 4.77723900 -0.05170300 -0.98132800
H 2.28893400 -2.11485300 -1.43173000

TS3-anti/M-SiMe$_3$ (singlet)

N -0.40385200 -0.12254200 -2.45138100
C -1.80147500 -0.37728500 -2.22889900
C -2.28760200 -1.68587800 -2.14801600
C -2.66801500 0.73563600 -2.20649900
C -3.66970300 -1.86097500 -1.96716300
C -4.55250800 -0.78407800 -1.89201000
H -4.06049200 -2.87975700 -1.88943400
C 0.52339400 0.30954300 -1.52442100
Fe 0.31243500 0.43642700 0.46548600
C 2.31780500 0.00976000 0.90608700
H 2.98768700 0.81360900 0.58872500
C 1.35185400 0.40479500 -3.67395400
H 2.09474900 0.61359000 -4.43748800
C 0.08739000 -0.07264600 -3.75178700
H -0.51956700 -0.37386900 -4.59989800
C -4.03059700 0.51157000 -2.02268500
H -4.70608500 1.36905700 -1.97075700
C 1.57686000 0.18714100 2.09832700
C -2.13586900 2.12563700 -2.42577300
H -1.28165500 2.33146300 -1.77022800
H -1.78506200 2.25462800 -3.46390700
H -2.91407900 2.87687500 -2.23504800
C -1.40132300 -2.89702800 -2.26002000
H -1.35160400 -3.44225600 -1.30555000
H -1.79826100 -3.59397300 -3.01525900
H -0.37320000 -2.63923200 -2.53628600
C -6.03176500 -0.98739800 -1.69618000
H -6.27708100 -2.04761300 -1.53676100
H -6.38730000 -0.41749100 -0.82435100
H -6.59939400 -0.63650300 -2.57448700

N 1.60461300 0.62592300 -2.32312200
C -3.63919800 1.78219400 1.16743500
C -3.73492100 0.36218500 1.26594300
C -4.97173500 -0.20832200 1.59004900
C -6.07716600 0.62483900 1.79716700
C -5.96466900 2.01962000 1.68518800
C -4.73564100 2.61694800 1.37054600
C -1.54542800 0.97275200 0.69169500
C -2.41198000 -0.13138100 0.97704200
H -5.07622900 -1.29301200 1.67090100
H -7.04459700 0.18317200 2.04943200
H -6.84158700 2.64957500 1.85195200
H -4.64667000 3.70270200 1.29490200
H -0.26644500 0.77668600 1.81781100
N -2.31779100 2.10813100 0.83148300
C -1.85243000 3.46727700 0.72593700
H -2.49228300 4.05657200 0.04922100
H -1.83522800 3.96805600 1.70971700
H -0.83447800 3.46530000 0.32057300
C -1.77776100 -1.37769400 0.96090400
H -2.30425500 -2.32120800 1.15004300
N -0.48185400 -1.37898400 0.68651900
C 0.25316000 -2.57335200 0.75389100
C 1.23819200 -2.85404000 -0.21459900
C 0.05318500 -3.51897500 1.77116300
C 1.96986500 -4.03130700 -0.17770300
H 1.40464300 -2.12738500 -1.00659200
C 0.78386500 -4.71243500 1.81626200
H -0.67171400 -3.30869000 2.55995000
C 1.75042300 -4.97816900 0.83844300
H 2.72570300 -4.25051100 -0.93459100
H 0.59956100 -5.41329800 2.63022700
O 2.51829100 -6.10031600 0.79296200
C 2.34427700 -7.07842100 1.78833300
H 1.31723500 -7.48789400 1.78907600
H 3.04992600 -7.88916900 1.56209200
H 2.56461400 -6.68385700 2.79730300
H 2.64897400 -0.98692400 0.60858700
C 2.83749600 1.21060000 -1.89028800
C 4.00722500 0.43655400 -1.87158800
C 2.84046000 2.56706800 -1.52204300
C 5.19176600 1.05067600 -1.44353700
C 4.04286700 3.13286800 -1.09116700
C 5.22981800 2.38841600 -1.03800700
H 6.10867500 0.45613700 -1.41134900
H 4.05401700 4.18416600 -0.79079900
C 1.57086400 3.37326900 -1.56618600
H 1.10277300 3.33561300 -2.56233300
H 0.82699200 2.97054200 -0.86229300
H 1.75698700 4.42289700 -1.30263400
C 3.99365700 -1.02988500 -2.21417400
H 5.01019400 -1.39410500 -2.41820000
H 3.59349100 -1.61225800 -1.36890100
H 3.36300900 -1.25588000 -3.08523300
C 6.50402800 3.01826500 -0.53649500
H 6.67823400 4.00022500 -1.00435400
H 6.45659600 3.18343700 0.55328800
H 7.37761100 2.38200200 -0.73909100
Si 1.77284100 1.69593200 3.18733500
H 1.26182500 -0.71916200 2.63353600
C 0.28311400 1.88271700 4.33282500
H 0.13590900 0.97286100 4.93815600
H 0.41523600 2.72928700 5.02683600
H -0.64210600 2.05059500 3.75883900
C 1.92131700 3.24395500 2.10928000
H 2.76455700 3.16526700 1.40580400
H 1.00342200 3.40929500 1.52449000
H 2.08079200 4.13946000 2.73224000
C 3.34844200 1.53996400 4.22337200
H 3.50470700 2.42309000 4.86532800
H 3.30623100 0.65129200 4.87463900
H 4.22855700 1.43138200 3.56849200

TS3-anti/M-$SiMe_3$ (triplet)

N 0.22897400 0.10902000 -2.54628300
C 1.60950500 -0.15360800 -2.25908200
C 2.46361900 0.93667000 -2.02675700
C 2.06923900 -1.47792400 -2.26584100
C 3.80736800 0.66619800 -1.75635000
C 4.29945100 -0.64660800 -1.71485700
H 4.48143200 1.50298500 -1.55906400
C -0.77138500 0.25138400 -1.61454000
Fe -0.68608700 -0.07661700 0.35462500
C -2.17556600 1.20449400 1.10183800
H -3.18017800 0.95135300 0.75726100
C -1.56519800 0.52631900 -3.75315100
H -2.31924600 0.71805100 -4.51033900

C -0.23553500 0.27404300 -3.84528000
H 0.42664600 0.19691200 -4.70217400
C 3.42372200 -1.70086900 -1.99029800
H 3.79010000 -2.72935200 -1.95508200
C -1.60960900 0.50608100 2.20270600
C 1.14170300 -2.62338300 -2.56982800
H 0.15699200 -2.46485600 -2.11178100
H 0.98716700 -2.73132400 -3.65736900
H 1.55165900 -3.56802700 -2.18806800
C 1.93768900 2.34554100 -2.07100600
H 1.64524400 2.62997700 -3.09611100
H 1.04753900 2.45094300 -1.43620600
H 2.69067800 3.05946000 -1.71253300
C 5.73320100 -0.91466100 -1.33406200
H 6.41040200 -0.14201000 -1.72971400
H 5.85040600 -0.91725100 -0.23569700
H 6.07365400 -1.89470300 -1.69924700
N -1.87208400 0.51433600 -2.39610500
C 1.05073400 -3.96658600 0.66322400
C 2.15668400 -3.09150500 0.86087100
C 3.43995200 -3.64093400 0.98955700
C 3.59667000 -5.02880500 0.92036000
C 2.49218600 -5.87506100 0.72400900
C 1.19968400 -5.35095800 0.59164500
C 0.21104800 -1.83568700 0.56461900
C 1.61298500 -1.75598300 0.82930900
H 4.30858300 -2.99352900 1.13230000
H 4.59432800 -5.46426300 1.01746800
H 2.64175700 -6.95596800 0.67031600
H 0.34318700 -6.00906500 0.43167700
H -0.52773700 -0.86192600 1.67250900
N -0.09779900 -3.18086600 0.52371300
C -1.39546200 -3.73665200 0.23676400
H -1.43032600 -4.18929100 -0.77049300
H -1.66328500 -4.51116800 0.97337200
H -2.14015700 -2.93672400 0.29070900
C 2.21509300 -0.50099900 1.06287000
H 3.28135700 -0.47071200 1.33111100
N 1.49565100 0.59110100 0.98476300
C 2.04006500 1.86041300 1.18169000
C 1.14886600 2.95005000 1.29986100
C 3.41651900 2.15237700 1.21998400
C 1.60289900 4.25223500 1.45035600
H 0.07945100 2.74777600 1.25722400
C 3.88460900 3.46125400 1.36885300
H 4.14841900 1.35242200 1.11062400
C 2.98023200 4.52614200 1.48497200
H 0.90537000 5.08717300 1.54076200
H 4.96052100 3.63604400 1.38580200
O 3.33610900 5.83243400 1.62844400
C 4.70250300 6.15882900 1.67310100
H 5.22596400 5.87476200 0.74120400
H 4.76421700 7.24861000 1.79628300
H 5.21579100 5.67347500 2.52370400
H -1.88791600 2.24182900 0.90076500
C -3.17608900 0.82725000 -1.89733600
C -3.49627600 2.17289000 -1.65926800
C -4.08607300 -0.21096400 -1.65543400
C -4.76382900 2.46231000 -1.14435300
C -5.33966200 0.12418600 -1.13153400
C -5.69381400 1.45253200 -0.86294100
H -5.02494000 3.50400600 -0.93903700
H -6.05439700 -0.67558600 -0.92012800
C -3.70812000 -1.63850900 -1.94515800
H -3.63002200 -1.81893300 -3.03045200
H -2.72580900 -1.87990400 -1.51627400
H -4.45101500 -2.33730400 -1.53659200
C -2.47671800 3.25280100 -1.90272300
H -1.57074000 3.06845800 -1.30389800
H -2.15711100 3.28205800 -2.95670200
H -2.87492600 4.24133500 -1.63613100
C -7.02787900 1.78381700 -0.24421700
H -7.79957800 1.05338400 -0.53001400
H -6.95932400 1.77362700 0.85752500
H -7.37385400 2.78551800 -0.54015600
Si -2.63075000 -0.72980200 3.18186200
H -0.83167600 1.02744000 2.77786000
C -1.55491500 -2.13712600 3.83279500
H -0.69809800 -1.73747700 4.40015200
H -2.12716900 -2.79514800 4.50758800
H -1.14577800 -2.75264500 3.01744900
C -4.00509700 -1.41389200 2.07687200
H -4.74851100 -0.63565800 1.84387500
H -3.61019100 -1.78044100 1.11792200
H -4.52814200 -2.24818200 2.57229100
C -3.42852700 0.16188500 4.64657200
H -4.07294300 -0.51732600 5.22953600
H -2.66265400 0.56693600 5.32845400

H -4.04726800 1.00497500 4.29815500

TS3-anti/M-SiMe$_3$ (quintet)

N 0.21011600 -1.31007700 -2.45571100
C -1.15734000 -1.61730500 -2.13380200
C -1.44662200 -2.84984200 -1.52526000
C -2.14432400 -0.65098200 -2.38183900
C -2.76840900 -3.08386600 -1.13535800
C -3.78248700 -2.13900000 -1.34824200
H -3.00798500 -4.02791700 -0.63823900
C 0.99388600 -0.54293300 -1.65075900
Fe 0.45485400 0.50900800 0.07984700
C 1.83276300 1.35944800 1.48670600
H 2.58862200 1.98084100 0.99022600
C 2.16839200 -1.24880500 -3.46879400
H 3.03476700 -1.33993100 -4.11704100
C 0.90729200 -1.75322400 -3.57344000
H 0.44533500 -2.37674900 -4.33309900
C -3.45434800 -0.93606400 -1.98070800
H -4.22903200 -0.18212700 -2.13495000
C 0.72479300 2.00918800 2.14910700
C -1.80063400 0.65722200 -3.04221800
H -1.09283400 1.22668700 -2.42317500
H -1.32783400 0.50210600 -4.02578600
H -2.70034600 1.27177500 -3.17968400
C -0.36863500 -3.87571400 -1.29844200
H -0.05454800 -4.34258200 -2.24746500
H 0.52324500 -3.42528600 -0.84211000
H -0.71817400 -4.66917900 -0.62565600
C -5.19480300 -2.42030800 -0.90337700
H -5.21795700 -2.77499000 0.13967800
H -5.82139200 -1.52035700 -0.96800700
H -5.65710600 -3.20845800 -1.52197400
N 2.19434900 -0.51004400 -2.29048000
C -3.52582500 2.38091800 -0.03340700
C -3.68732000 1.13865200 0.64782300
C -4.98082000 0.67782100 0.92295700
C -6.08189500 1.44111100 0.51693800
C -5.90432600 2.66065900 -0.15518500
C -4.62069600 3.14929500 -0.43575900
C -1.40264100 1.54972300 0.29024200
C -2.35684000 0.61116500 0.83669900
H -5.13027600 -0.27148400 1.44238500
H -7.09343100 1.08561200 0.72844800
H -6.77761200 3.24256400 -0.45972200
H -4.48723900 4.10275000 -0.95067600
H -0.51446300 1.92091200 1.20030300
N -2.16740700 2.59926900 -0.23408600
C -1.64768700 3.69126600 -1.01283400
H -2.24394300 3.83313600 -1.92984800
H -1.64067300 4.64435600 -0.45530400
H -0.61664300 3.45502900 -1.30430100
C -1.91963400 -0.62934600 1.31010000
H -2.65683000 -1.34478300 1.69520800
N -0.62660300 -0.93529900 1.25524200
C -0.13136200 -2.17451400 1.64718800
C 1.26265300 -2.39351400 1.53357900
C -0.90604500 -3.25400200 2.11727800
C 1.84347700 -3.61127600 1.85526700
H 1.88497700 -1.57343200 1.17419700
C -0.32893600 -4.48575500 2.44081300
H -1.98590800 -3.15056800 2.22418600
C 1.05289900 -4.68014400 2.30892400
H 2.92035400 -3.76499500 1.76065000
H -0.97498200 -5.28969800 2.79432000
O 1.70554500 -5.84305100 2.59208600
C 0.95744500 -6.93824900 3.05527700
H 0.20147200 -7.26509300 2.31680200
H 1.66724500 -7.75978500 3.22290800
H 0.44034600 -6.71360000 4.00661000
H 2.25775700 0.46813000 1.96648100
C 3.31263200 0.21327600 -1.75714000
C 4.31526200 -0.49391400 -1.07437200
C 3.32772600 1.61356400 -1.86729400
C 5.35568800 0.24229000 -0.49649000
C 4.38916300 2.30459700 -1.27406400
C 5.40722000 1.63939500 -0.57953000
H 6.13971600 -0.29179600 0.04692700
H 4.41123900 3.39543500 -1.34465100
C 2.20463000 2.35394900 -2.54229200
H 1.89597300 1.87181100 -3.48167000
H 1.31585200 2.37149000 -1.88772700
H 2.48691600 3.39443900 -2.75282400
C 4.25325300 -1.99403600 -0.94853000
H 3.26761900 -2.32418200 -0.58736900
H 4.41817500 -2.49147500 -1.91843200
H 5.01693000 -2.36115000 -0.24920000

C 6.50341400 2.41519000 0.10416200
H 6.18068900 2.73530300 1.11002000
H 7.41304800 1.80923300 0.22830400
H 6.76682800 3.32383800 -0.45805800
Si 0.68135200 3.87035000 2.34625400
H 0.26297200 1.43747700 2.96740400
C -1.08663200 4.46538900 2.63737700
H -1.48216400 4.02696800 3.56870200
H -1.13128200 5.56231400 2.73958800
H -1.76393800 4.16335500 1.82489700
C 1.43732200 4.66823200 0.80399600
H 2.50101600 4.39294000 0.71629100
H 0.93435500 4.34688200 -0.11869900
H 1.37799400 5.76769300 0.85873500
C 1.74555400 4.38093300 3.82770700
H 1.77771500 5.47806900 3.94122100
H 1.35050100 3.95522500 4.76459200
H 2.77903800 4.01864600 3.70354200

int4-M-SiMe$_3$ (singlet)

N 0.69475000 -0.24632000 -2.48856600
C 1.97870800 0.33365100 -2.21336300
C 2.11525200 1.72470800 -2.14960700
C 3.07633200 -0.53283700 -2.04910100
C 3.38942800 2.24152900 -1.86867500
C 4.49829400 1.41586400 -1.67349500
H 3.51021700 3.32644800 -1.79877200
C -0.21168500 -0.66263800 -1.54780100
Fe -0.18240300 -0.65410200 0.44447600
C -0.71702300 -1.22718700 2.28041800
H -0.95912600 -2.28425200 1.98896400
C -0.94142000 -1.12185900 -3.67953700
H -1.62410600 -1.50419000 -4.43172000
C 0.27002200 -0.52092500 -3.78384400
H 0.87364100 -0.26309700 -4.64865300
C 4.32222700 0.02798300 -1.77158400
H 5.17497000 -0.63386800 -1.60157700
C 2.91475500 -2.02156500 -2.20054200
H 1.98730700 -2.37525200 -1.73303200
H 2.87156500 -2.30677800 -3.26600700
H 3.76071000 -2.55234900 -1.74300600
C 0.96135200 2.66013800 -2.39469200
H 1.05084100 3.13281400 -3.38791700
H -0.00640400 2.15009500 -2.34816600
H 0.94175200 3.46659200 -1.64762400
C 5.85936100 1.98537200 -1.37021300
H 5.81226900 3.06707200 -1.17663500
H 6.29643300 1.49390500 -0.48781100
H 6.55245500 1.82393200 -2.21325300
N -1.22234200 -1.19795700 -2.31786800
C 3.76408700 -1.50460700 1.34297700
C 3.68801600 -0.08234800 1.42576500
C 4.83974900 0.63554500 1.76720100
C 6.03444800 -0.05547800 2.00092200
C 6.09263200 -1.45429500 1.90019100
C 4.95056100 -2.19833200 1.57098100
C 1.60958700 -0.95374500 0.80378600
C 2.32488300 0.25082600 1.09103200
H 4.80952900 1.72524200 1.84132900
H 6.93663700 0.50177200 2.26578500
H 7.03680400 -1.97239100 2.08403500
H 4.99723800 -3.28663100 1.49403700
N 2.49703000 -1.99289700 0.99291700
C 2.17563400 -3.39815500 0.97762300
H 2.52360900 -3.90075800 0.05821100
H 2.63598800 -3.90251100 1.84162100
H 1.08920500 -3.51578400 1.05721400
C 1.56244700 1.41741400 0.98760200
H 1.97379400 2.41990800 1.14287600
N 0.29214000 1.24039700 0.64001000
C -0.60729600 2.29978300 0.51349500
C -1.77581700 2.10030800 -0.24822400
C -0.43618500 3.55984200 1.11287000
C -2.71677800 3.10454400 -0.42007600
H -1.92170100 1.12967100 -0.71908200
C -1.37471600 4.58138900 0.94062000
H 0.42714700 3.74620900 1.75259800
C -2.52632100 4.36398800 0.17047300
H -3.61877100 2.93734100 -1.01149900
H -1.20334100 5.53862700 1.43269000
O -3.49865800 5.29043000 -0.04866900
C -3.36616200 6.56043300 0.54063800
H -2.45775200 7.08500800 0.19098700
H -4.24885600 7.14164000 0.24155900
H -3.33393300 6.50155700 1.64411000
C 0.27861100 -1.24873300 3.45262900
H 0.68632200 -0.23978000 3.62751400
H -0.15856900 -1.58961200 4.41138100

H 1.14643600 -1.89673600 3.25626700
C -2.41940400 -1.78301600 -1.79471000
C -2.33142800 -3.00792100 -1.10641100
C -3.65377200 -1.15021900 -2.02030600
C -3.51101500 -3.57775800 -0.61987800
C -4.80940700 -1.77150500 -1.53122600
C -4.76022900 -2.97519400 -0.82149800
H -3.45286400 -4.52237000 -0.07277300
H -5.77441600 -1.28234000 -1.68816100
C -3.75285000 0.15176700 -2.77367800
H -2.87252800 0.78853500 -2.61625300
H -3.83388700 -0.01827700 -3.86064400
H -4.64582700 0.71184300 -2.46190300
C -1.00622800 -3.70012700 -0.93564000
H -0.56625300 -3.96041500 -1.91252200
H -0.26956600 -3.05429300 -0.43583800
H -1.11200700 -4.61933400 -0.34413500
C -6.00932100 -3.57804800 -0.23454900
H -6.17041400 -3.20243200 0.79090500
H -6.90122100 -3.31692800 -0.82323800
H -5.94131200 -4.67460500 -0.17472200
Si -2.34744200 -0.48577300 2.86479300
C -2.08507700 1.22650300 3.62004400
H -1.68592300 1.93557800 2.88085300
H -3.03562500 1.63342000 4.00353800
H -1.37565000 1.17927900 4.46210000
C -3.12413000 -1.59092100 4.20633400
H -2.45178100 -1.72202100 5.06945500
H -4.06748900 -1.15676000 4.57966500
H -3.35109100 -2.59302900 3.80415000
C -3.65043300 -0.38041000 1.49713900
H -3.80025800 -1.36502600 1.03199900
H -4.61688700 -0.06005600 1.92155100
H -3.39054700 0.33428100 0.70544000

int4-M-SiMe$_3$ (triplet)

N 0.69555900 -1.46696900 -2.22238500
C 1.92785400 -0.72948300 -2.17235600
C 1.93974600 0.61762600 -2.55647500
C 3.09752900 -1.39922200 -1.76680400
C 3.15243100 1.31324700 -2.45539600
C 4.32527600 0.69738600 -2.01318700
H 3.17225400 2.37194300 -2.72846500
C -0.29558200 -1.41688800 -1.27900900
Fe -0.31697700 -0.45117200 0.55528600
C -0.73864200 -0.73677400 2.54296100
H -1.02567200 -1.80814100 2.57393800
C -0.85615800 -2.82502800 -2.99767100
H -1.48448700 -3.52639400 -3.53812900
C 0.37274600 -2.31693400 -3.27171100
H 1.04796100 -2.47725800 -4.10655900
C 4.28001400 -0.66420100 -1.68367500
H 5.18569700 -1.15980800 -1.32541800
C 3.09292800 -2.87308800 -1.46251600
H 2.13678400 -3.20078800 -1.03780700
H 3.26140600 -3.46350700 -2.38009200
H 3.89508400 -3.12340400 -0.75491400
C 0.72259500 1.30733100 -3.10977000
H 0.75797900 1.32323900 -4.21310900
H -0.20611900 0.80804100 -2.81351600
H 0.66633800 2.34786200 -2.76362600
C 5.61598000 1.46253000 -1.88516300
H 5.47883000 2.53055100 -2.10873500
H 6.01682600 1.36988000 -0.86382400
H 6.38240900 1.06743400 -2.57277700
N -1.25067300 -2.26100000 -1.78853000
C 3.60978200 -1.21498500 1.65165900
C 3.60650000 0.18868900 1.39993800
C 4.79272300 0.90956100 1.58533700
C 5.94742800 0.23520600 1.99820000
C 5.93159500 -1.14843100 2.23557600
C 4.75396700 -1.89103700 2.07066100
C 1.48889300 -0.69501800 0.96902700
C 2.26230500 0.50555100 0.97778500
H 4.81833200 1.98745200 1.40729800
H 6.87540500 0.79417800 2.14317200
H 6.84366400 -1.65285000 2.56323400
H 4.73992500 -2.96434900 2.27110500
N 2.32596800 -1.70640600 1.38030900
C 1.91440900 -3.05550000 1.67288600
H 2.68925300 -3.77527800 1.37074800
H 1.70971700 -3.19248000 2.74922600
H 0.99830700 -3.27079300 1.11457800
C 1.61849600 1.69081400 0.59290100
H 2.16844300 2.64306600 0.56814800
N 0.34430300 1.64149400 0.24471900
C -0.36090400 2.81958200 -0.02173100

C -1.44288600 2.79349400 -0.92752700
C -0.08301000 4.04819100 0.60588000
C -2.18927500 3.93017700 -1.20495400
H -1.68660200 1.85501500 -1.42232700
C -0.82627800 5.20026900 0.33199400
H 0.70975500 4.10252400 1.35399400
C -1.88880300 5.15157300 -0.57980600
H -3.02158900 3.90065700 -1.91097600
H -0.57745300 6.12351800 0.85518600
O -2.67790600 6.21138300 -0.90930900
C -2.42531600 7.45380700 -0.30196300
H -1.41057200 7.82776800 -0.53267500
H -3.16395700 8.15969100 -0.70546000
H -2.53898100 7.40711700 0.79688700
C 0.24020400 -0.42948200 3.67789900
H 0.62742400 0.59919000 3.59875800
H -0.21233700 -0.52640600 4.68488100
H 1.12408000 -1.08765400 3.66552100
C -2.56143900 -2.44634300 -1.23613000
C -2.74508600 -3.29666000 -0.13466900
C -3.63293400 -1.75484300 -1.82739800
C -4.03910900 -3.43030900 0.38174500
C -4.91136100 -1.93719600 -1.28962600
C -5.13348100 -2.76003000 -0.17857600
H -4.19437600 -4.08041400 1.24698600
H -5.75133200 -1.39574500 -1.73243800
C -3.39897300 -0.79129300 -2.96079700
H -3.02368700 -1.29368200 -3.86602600
H -4.32453300 -0.26134800 -3.22288200
H -2.64185600 -0.04129300 -2.68085900
C -1.58667000 -4.01717500 0.49673600
H -0.88938800 -4.41035300 -0.25761800
H -1.01736700 -3.32577500 1.13623900
H -1.93383700 -4.84916100 1.12467200
C -6.50430300 -2.88612900 0.43395300
H -6.59382800 -2.23062900 1.31728000
H -7.29390500 -2.59694400 -0.27480800
H -6.70233100 -3.91478200 0.77212300
Si -2.30040900 0.27522100 2.53886100
C -1.99265800 2.03898800 3.13083500
H -1.17465600 2.50528000 2.56403000
H -2.89074100 2.66602400 3.00513700
H -1.71863800 2.04819400 4.19850200
C -3.72383700 -0.46374300 3.55652200
H -3.46615200 -0.51846700 4.62718100
H -4.64467600 0.13768800 3.46059200
H -3.94965500 -1.48675000 3.21045400
C -2.93166300 0.29492800 0.73755500
H -3.66313600 -0.50848900 0.58565100
H -3.41533700 1.25680600 0.50886200
H -2.17459800 0.17134500 -0.06597400

int4-M-SiMe$_3$ (quintet)

N 0.40526300 -0.76878900 -2.61638800
C 1.78477000 -0.41502700 -2.41711800
C 2.21765100 0.90373500 -2.59887800
C 2.66704100 -1.43755200 -2.01142900
C 3.56416700 1.19176700 -2.32669100
C 4.46196200 0.21394100 -1.89550200
H 3.91309300 2.22114300 -2.44833400
C -0.46302000 -0.99650800 -1.58782400
Fe -0.11236800 -0.66841200 0.45425400
C -1.59273200 -1.30731000 1.74500100
H -2.47145200 -1.59581300 1.14362800
C -1.43468800 -1.49107000 -3.59183000
H -2.23053300 -1.82445100 -4.25107100
C -0.16791500 -1.06762800 -3.84835600
H 0.37802700 -0.95284900 -4.77996600
C 3.99430800 -1.10057200 -1.75478600
H 4.68372900 -1.87897800 -1.41833100
C 2.18044600 -2.85052400 -1.83804900
H 1.51760200 -2.92498000 -0.96363800
H 1.60114300 -3.19422200 -2.70897500
H 3.02293300 -3.53940200 -1.68850100
C 1.30086100 2.00876700 -3.05199700
H 1.73282700 2.54030000 -3.91465600
H 0.30804200 1.64235100 -3.33733300
H 1.15454600 2.74789700 -2.25012900
C 5.90178600 0.53765700 -1.59891600
H 6.07088500 1.62269600 -1.55304100
H 6.20851000 0.10793800 -0.63416300
H 6.56592400 0.12275700 -2.37656700
N -1.59416000 -1.44125300 -2.20959800
C 4.09939800 -0.85783800 1.69830900
C 3.83462000 0.52192600 1.46323100
C 4.86851200 1.45124300 1.64847000
C 6.12674400 0.99794500 2.04980100
C 6.37184200 -0.37107300 2.26550600

C 5.35715300 -1.31828600 2.09305400
C 1.90954100 -0.72882900 1.05659700
C 2.44908600 0.58077000 1.05898200
H 4.69600600 2.51675500 1.47717500
H 6.93813400 1.71556100 2.19400800
H 7.36680400 -0.69891500 2.57571300
H 5.54684700 -2.37963700 2.26605100
N 2.91819900 -1.56297300 1.45614000
C 2.80300900 -2.99071300 1.64070700
H 3.45383100 -3.54006000 0.93974500
H 3.08436200 -3.27611100 2.66778000
H 1.76051900 -3.28269000 1.47063700
C 1.63248100 1.68280100 0.69256700
H 2.05652800 2.69995300 0.70074900
N 0.38950900 1.49740600 0.33077300
C -0.45116900 2.58533100 0.06817300
C -1.38186300 2.49196900 -0.98447100
C -0.45257600 3.75483100 0.84400900
C -2.25607000 3.53107700 -1.26641800
H -1.40001800 1.57820600 -1.57548100
C -1.33458600 4.80582700 0.57198400
H 0.21693700 3.82982300 1.70285500
C -2.24298800 4.70300400 -0.49021000
H -2.97592900 3.45976700 -2.08393500
H -1.31558700 5.68853900 1.21073800
O -3.14354500 5.66199100 -0.83259500
C -3.19315000 6.84569900 -0.07362800
H -2.23610600 7.39798700 -0.10841600
H -3.98064100 7.47110800 -0.51511800
H -3.44523100 6.64601900 0.98390300
C -1.03144600 -2.55093300 2.45665200
H -0.16591600 -2.30260500 3.09588400
H -1.76927500 -3.05702800 3.11090400
H -0.68094500 -3.32654600 1.74803900
C -2.73788900 -1.94620400 -1.50065900
C -2.65873600 -3.25008600 -0.97609700
C -3.86649800 -1.13791800 -1.30754700
C -3.73239600 -3.72275300 -0.21947400
C -4.91826100 -1.65712400 -0.53956200
C -4.86490800 -2.93412300 0.02546700
H -3.67472100 -4.72721000 0.20798600
H -5.79868500 -1.03167300 -0.36843000
C -3.95548800 0.26763200 -1.83765600
H -3.29470900 0.43712800 -2.69807800
H -4.98603700 0.50503800 -2.14011600
H -3.67039800 0.98585000 -1.05390100
C -1.42379200 -4.08735700 -1.17108700
H -1.08074800 -4.08145600 -2.21685700
H -0.59644300 -3.69149100 -0.56169500
H -1.60163900 -5.12779800 -0.86674300
C -5.97262200 -3.43449900 0.91547000
H -5.74331600 -3.21365900 1.97237900
H -6.93270200 -2.95192000 0.67980900
H -6.10128700 -4.52410400 0.82974500
Si -2.19821600 0.02363500 2.90075500
C -0.76754700 0.95591500 3.72385900
H -0.14629100 1.46261800 2.97047000
H -1.13184300 1.71156800 4.43980000
H -0.11305300 0.25890200 4.27327700
C -3.26844800 -0.70265000 4.29922400
H -2.69059100 -1.40227600 4.92584900
H -3.66618000 0.08697300 4.95932000
H -4.12617000 -1.25934900 3.88366300
C -3.29642800 1.24517400 1.95663400
H -4.14507300 0.71075600 1.49711300
H -3.70809100 2.01605900 2.62895800
H -2.74530800 1.76009000 1.15735700

int4-anti/M-SiMe$_3$ (singlet)

N -1.18137200 -2.52822700 -1.14984600
C -2.45395400 -2.30212900 -0.52852900
C -2.62732300 -2.64334900 0.81766000
C -3.47293000 -1.70398600 -1.28688700
C -3.86244700 -2.35284300 1.41076900
C -4.89598100 -1.73658500 0.69833400
H -4.01155800 -2.59707500 2.46591200
C -0.22474400 -1.56599900 -1.27201500
Fe 0.03098300 0.32263600 -0.80016500
C 0.84487300 2.15254000 -0.70684400
H 0.49109500 2.77570300 -1.55386700
C 0.46742700 -3.52811900 -2.23412700
H 1.13599800 -4.19054900 -2.77397600
C -0.78003300 -3.73013600 -1.73174300
H -1.42177800 -4.60606900 -1.73935300
C -4.68382800 -1.42577100 -0.65210300
H -5.47872400 -0.93347000 -1.21809900
C -3.24260600 -1.36786500 -2.73490100
H -2.38740800 -0.68599100 -2.84065100

H -3.01089200 -2.26926100 -3.32590700
H -4.12743100 -0.88844000 -3.17507500
C -1.50979400 -3.28408100 1.59571500
H -1.33430500 -4.32347400 1.27024100
H -0.56547500 -2.74055900 1.45423000
H -1.73952900 -3.30537800 2.67025000
C -6.20360500 -1.38401800 1.35577100
H -6.24568500 -1.74603300 2.39323300
H -6.33806400 -0.29131300 1.37300600
H -7.05576100 -1.81639100 0.80616200
N 0.78857700 -2.20547500 -1.94281700
C -3.76408300 1.82684700 -0.09635300
C -3.33225800 1.36677400 1.18356800
C -4.18222800 1.53596800 2.28292000
C -5.42824800 2.14746100 2.09853800
C -5.83799700 2.58710000 0.83031600
C -5.00366300 2.43212100 -0.28708200
C -1.68308600 0.92496900 -0.42104000
C -2.01954100 0.80879500 0.96821600
H -3.87784700 1.19435700 3.27534500
H -6.09409000 2.28247000 2.95464200
H -6.81524400 3.06066200 0.70984600
H -5.32071400 2.78165800 -1.27188300
N -2.75316100 1.55369100 -1.02741700
C -2.80903200 2.03291800 -2.38482900
H -3.68327200 1.62883500 -2.92165200
H -2.86693600 3.13467900 -2.41420900
H -1.90074000 1.72334400 -2.91355500
C -0.99929200 0.28244600 1.76789100
H -1.07591400 0.16659800 2.85517500
N 0.11498600 -0.03341900 1.12050300
C 1.24775700 -0.56386200 1.73606600
C 2.50237700 -0.35486400 1.12289800
C 1.22349900 -1.31731600 2.92391900
C 3.66758100 -0.87602000 1.66601000
H 2.55643400 0.24994100 0.21906400
C 2.39477600 -1.84407800 3.47718000
H 0.27333000 -1.51597700 3.42101000
C 3.62947000 -1.63311600 2.84726600
H 4.63281100 -0.70313000 1.18736400
H 2.32566000 -2.42937400 4.39405500
O 4.82110000 -2.12060100 3.29265200
C 4.84317100 -2.85791500 4.48949400
H 4.23652000 -3.77989400 4.41865200
H 5.89020800 -3.13583500 4.67126500
H 4.47702300 -2.26365900 5.34686600
C 0.82041800 2.99188600 0.57668500
H -0.14560300 3.51858400 0.67291100
C 1.99811500 -1.49577100 -2.20685900
C 1.90347800 -0.26248000 -2.88526500
C 3.21665600 -1.95302100 -1.67285400
C 3.06725800 0.50254000 -3.02908900
C 4.35155500 -1.15675100 -1.85827900
C 4.29815200 0.07776600 -2.51861800
H 3.00212000 1.46553100 -3.54107500
H 5.30138800 -1.49574400 -1.43653200
C 3.29179700 -3.21529300 -0.85481200
H 2.46072500 -3.26302800 -0.13566300
H 3.24196900 -4.12173800 -1.47999000
H 4.23027800 -3.24940800 -0.28595500
C 0.59659900 0.23938200 -3.45035300
H 0.00804800 -0.56850700 -3.90609500
H -0.08381800 0.74257000 -2.72293900
H 0.78508300 1.01599800 -4.20532000
C 5.52047700 0.95230400 -2.62482800
H 5.57679400 1.64105200 -1.76419000
H 6.44561900 0.35721800 -2.63169100
H 5.49809300 1.56959100 -3.53528600
H 1.89734200 1.90111600 -0.96750800
H 0.88073000 2.34650900 1.47127500
Si 2.24860800 4.21578200 0.69841400
C 2.24251900 5.39673000 -0.78185300
H 2.29808300 4.83207000 -1.72684400
H 1.31655200 5.99551100 -0.80481100
H 3.09563400 6.09483800 -0.75136200
C 3.88190900 3.25024200 0.69156300
H 3.91340600 2.52513100 1.52152700
H 3.99559400 2.68341300 -0.24699100
H 4.75058400 3.92224900 0.78987400
C 2.14893400 5.22360300 2.29860600
H 2.99348800 5.92686100 2.39192400
H 1.21584300 5.81009600 2.33820800
H 2.16352800 4.56087800 3.18001200

int4-anti/M-$SiMe_3$ (triplet)

N -0.66770800 -1.01937100 -2.32157300
C -2.02379800 -0.66960400 -2.01167500

C -2.89541000 -1.67682000 -1.56891600
C -2.43804600 0.66483600 -2.16929000
C -4.19363200 -1.30003800 -1.20641300
C -4.62954400 0.02851800 -1.29419100
H -4.87905800 -2.06722500 -0.83579500
C 0.40712800 -0.72167100 -1.51879500
Fe 0.22898700 0.41692600 0.16064600
C 2.04489400 1.14649800 0.37434100
H 2.38040000 1.83459900 -0.42104500
C 1.06087100 -1.82226800 -3.42070700
H 1.77146900 -2.27349100 -4.10650300
C -0.28820200 -1.68394500 -3.48066100
H -1.01002200 -1.98786000 -4.23239800
C -3.74383900 0.99099900 -1.79339600
H -4.06878200 2.03131900 -1.86806600
C -1.51063200 1.70525100 -2.73906900
H -0.59349500 1.77724600 -2.13771400
H -1.21189000 1.44829800 -3.76902400
H -1.99330200 2.69162900 -2.75145100
C -2.44668500 -3.11271100 -1.49304800
H -2.34066000 -3.55029500 -2.50008600
H -1.47217400 -3.20529800 -0.99382200
H -3.16943500 -3.72058900 -0.93463500
C -6.02733400 0.42018400 -0.88755600
H -6.47221400 -0.31886100 -0.20439400
H -6.03188500 1.40142900 -0.38979900
H -6.68941600 0.49643000 -1.76748000
N 1.46496200 -1.23126400 -2.22761600
C -2.25732100 3.84551500 0.50459600
C -3.03804500 2.78834300 1.05927800
C -4.37254100 3.04401600 1.40599000
C -4.89658200 4.32397200 1.20231400
C -4.10840400 5.35363900 0.65869700
C -2.77341800 5.12605700 0.30476700
C -0.90114700 2.00570800 0.53549100
C -2.16676900 1.63819700 1.06848600
H -4.99584900 2.25470100 1.83349500
H -5.93519600 4.53063300 1.47247000
H -4.54227400 6.34545100 0.51131400
H -2.16188100 5.92648000 -0.11683200
N -0.98601900 3.33403500 0.21846900
C 0.07407000 4.12271600 -0.36413300
H -0.25174100 4.57169200 -1.31756700
H 0.38571600 4.93668800 0.31175000
H 0.92998300 3.46518900 -0.55058100
C -2.35386500 0.28098600 1.41078000
H -3.28264200 -0.04829500 1.89732200
N -1.38859700 -0.57106700 1.14509800
C -1.45731900 -1.92181100 1.49049300
C -0.24579700 -2.64298300 1.53212200
C -2.64146500 -2.62466400 1.76848000
C -0.21668700 -3.99945900 1.82187300
H 0.68005900 -2.10039900 1.33161200
C -2.62286500 -3.99062900 2.06508900
H -3.60403100 -2.11458200 1.72108300
C -1.40948500 -4.69314200 2.08829300
H 0.72412100 -4.55231300 1.85166600
H -3.56688200 -4.49859000 2.26230700
O -1.28989000 -6.02212500 2.35365400
C -2.45144300 -6.76262800 2.63612600
H -3.16042400 -6.75791800 1.78749900
H -2.13006400 -7.79590400 2.82418400
H -2.97540700 -6.38236800 3.53225200
C 2.17473300 1.81169300 1.75280800
H 1.68670400 2.80176500 1.76416200
C 2.84297600 -1.06796400 -1.87036100
C 3.51435800 0.07772700 -2.32737600
C 3.47247800 -2.03441200 -1.07359600
C 4.84269400 0.25896900 -1.93361800
C 4.80067300 -1.80278300 -0.69305400
C 5.49647600 -0.65813000 -1.09985800
H 5.37488400 1.15358500 -2.26760300
H 5.30063200 -2.53440100 -0.05281900
C 2.74525800 -3.28325300 -0.65100800
H 1.72226100 -3.06586400 -0.31760200
H 2.65871800 -3.99288300 -1.49151800
H 3.27699600 -3.79422500 0.16383400
C 2.80124000 1.09224300 -3.18071400
H 2.48840200 0.66195600 -4.14611900
H 1.88807100 1.44704700 -2.67934500
H 3.44547600 1.95853500 -3.38396800
C 6.89384200 -0.38415600 -0.60914800
H 6.86446400 0.29217700 0.26231400
H 7.40308400 -1.30680500 -0.29440800
H 7.50619500 0.10525900 -1.38169400
H 2.71121500 0.26753200 0.31161400
H 1.64691200 1.22394800 2.53006000
Si 3.97497500 1.98789300 2.31178300

C 4.97893500 2.85193600 0.96026700
H 4.93894300 2.27090600 0.02595500
H 4.57868200 3.85777400 0.74940000
H 6.03649700 2.96488300 1.25158200
C 4.70700100 0.26905500 2.61299800
H 4.16933200 -0.24947600 3.42430400
H 4.63068900 -0.35261400 1.70742000
H 5.77040300 0.32795800 2.89922900
C 4.08671800 2.99396900 3.91135200
H 5.12915500 3.08672200 4.25972500
H 3.68821300 4.01216500 3.76783600
H 3.50394600 2.51860400 4.71788700

int4-anti/M-SiMe$_3$ (quintet)

N 1.13450000 0.90371800 -2.63360700
C 2.47408200 0.94764300 -2.12178500
C 2.84749900 2.02507700 -1.30390200
C 3.34611900 -0.10969600 -2.41255100
C 4.13717000 2.01878900 -0.76800300
C 5.04152600 0.98034400 -1.02839500
H 4.44043500 2.83782100 -0.11040700
C 0.11385400 0.26337200 -1.99679000
Fe 0.21413800 -0.85540000 -0.20633000
C -1.42513300 -2.02852200 0.20581100
H -1.44992900 -2.91587800 -0.45367000
C -0.62740500 1.28341700 -3.89671400
H -1.36120500 1.57562200 -4.64176500
C 0.70570000 1.53377300 -3.79645500
H 1.38492500 2.09090200 -4.43471200
C 4.63028500 -0.06824100 -1.85872600
H 5.32406600 -0.88668300 -2.06780500
C 2.88713800 -1.26767300 -3.25756800
H 2.08640600 -1.82582900 -2.74598100
H 2.47269100 -0.93168200 -4.22118100
H 3.71337100 -1.96273800 -3.46048400
C 1.86703200 3.12379400 -0.99316400
H 1.59727500 3.69688700 -1.89552300
H 0.93326400 2.71170900 -0.58425300
H 2.28456200 3.82654000 -0.25890500
C 6.40565300 0.98393200 -0.39331800
H 6.32779200 0.75298600 0.68073800
H 7.06613400 0.23158600 -0.84752100
H 6.88767600 1.97005700 -0.48635000
N -0.96531000 0.50831100 -2.79317000
C 4.24355700 -1.45486000 1.41177800
C 3.74686200 -0.27964600 2.04706100
C 4.56915400 0.39016800 2.96496200
C 5.84470300 -0.11454100 3.23230900
C 6.31503800 -1.27763500 2.59501400
C 5.51746400 -1.96231100 1.67258600
C 2.14117200 -1.12059200 0.57763700
C 2.41678900 -0.08986300 1.51331700
H 4.21922700 1.29481400 3.46879300
H 6.48973700 0.40151400 3.94773100
H 7.31719600 -1.64931400 2.82103500
H 5.88377400 -2.86309800 1.17590500
N 3.24774800 -1.92500800 0.55414000
C 3.39346400 -3.11843000 -0.24310900
H 4.19401100 -3.00369200 -0.99340900
H 3.63652000 -3.98745300 0.39040800
H 2.44430800 -3.31020500 -0.75758600
C 1.40513800 0.85539300 1.83359000
H 1.60516600 1.63332500 2.58836900
N 0.22982800 0.77965100 1.26289000
C -0.78939400 1.70077700 1.53301600
C -2.12177300 1.23861700 1.53612800
C -0.56815800 3.06703100 1.77056900
C -3.17996900 2.09866200 1.78232200
H -2.30739200 0.18611400 1.33870400
C -1.63276900 3.94541300 2.00557400
H 0.44818200 3.46376700 1.74765900
C -2.94923800 3.46567000 2.01206700
H -4.20795400 1.73224900 1.78960100
H -1.41899300 5.00158500 2.16879200
O -4.05251500 4.23822500 2.20718200
C -3.88092500 5.61063400 2.46178400
H -3.38941000 6.12882100 1.61759300
H -4.88445800 6.03433300 2.60225900
H -3.28591500 5.78876400 3.37626500
C -1.53529700 -2.47480800 1.67858400
H -0.82641800 -3.29634100 1.88800500
C -2.29065700 0.05190700 -2.48740700
C -2.63840100 -1.27355100 -2.79268700
C -3.17773400 0.93654000 -1.85644200
C -3.91138800 -1.71424900 -2.42298200
C -4.43710900 0.44302500 -1.49118400
C -4.81755100 -0.87791900 -1.75655200
H -4.19431400 -2.74831600 -2.63736400

H -5.13310000 1.10992100 -0.97620100
C -2.78240200 2.36366500 -1.58238400
H -2.64099300 2.92530100 -2.52062300
H -3.54831100 2.87606800 -0.98717200
H -1.83568900 2.41578200 -1.02511900
C -1.64497000 -2.19854100 -3.44199500
H -1.19019500 -1.74689300 -4.33756600
H -0.82492200 -2.42442000 -2.74171600
H -2.11885700 -3.14662100 -3.73090500
C -6.15348700 -1.41285000 -1.31000800
H -6.79221400 -0.61784500 -0.89942400
H -6.69229600 -1.89302500 -2.14254100
H -6.02063500 -2.17562800 -0.52523500
H -2.31066300 -1.43472300 -0.08450000
H -1.23968000 -1.66413000 2.37251200
Si -3.28917600 -3.01550500 2.13798200
C -3.93448300 -4.25313600 0.85882800
H -3.92610900 -3.79948100 -0.14497300
H -3.30365700 -5.15698700 0.82248400
H -4.96593400 -4.56967900 1.08704700
C -4.43959500 -1.50850200 2.13062300
H -4.14780000 -0.78366300 2.90781100
H -4.40719000 -0.99190200 1.15863800
H -5.48417200 -1.80568300 2.32156100
C -3.34660100 -3.80529900 3.85855900
H -4.37322500 -4.09399300 4.14033700
H -2.71881000 -4.71105500 3.90002400
H -2.97242100 -3.10521800 4.62424900

TS5-M-SiMe$_3$ (singlet)

N -0.74741500 -0.54991700 2.21170200
C -2.05015800 0.02057600 2.00481200
C -2.25532300 1.39549500 2.15604600
C -3.11578900 -0.86582800 1.74704400
C -3.55872400 1.88505200 1.97224600
C -4.63374500 1.04488000 1.67965500
H -3.73046900 2.96103500 2.06986200
C 0.15537000 -0.93342200 1.24388900
Fe 0.01526100 -0.69085900 -0.77495400
C 2.40061700 -0.71772400 -1.44359600
C 0.79990400 -1.63394900 3.34508200
H 1.43892100 -2.12967100 4.06890100
C -0.36836500 -0.96385400 3.48357800
H -0.97617400 -0.74584200 4.35615400
C -4.39258300 -0.33313900 1.57800100
H -5.22041900 -1.00629600 1.34206800
C 1.42842100 -1.23403800 -2.30402200
H 1.13804700 -2.29851500 -2.21980200
C -2.88910100 -2.35333200 1.70599300
H -1.99570000 -2.60770500 1.12282900
H -2.73951900 -2.75592400 2.72267100
H -3.75382200 -2.86572600 1.26298700
C -1.15132400 2.34528100 2.53624600
H -1.33997600 2.76986100 3.53673200
H -0.16990600 1.86089300 2.54883700
H -1.09062200 3.18569900 1.82948800
C -6.02782500 1.58497200 1.49565500
H -6.03163300 2.68307900 1.43206200
H -6.48158000 1.18340300 0.57718500
H -6.67815100 1.29492600 2.33854400
N 1.10555400 -1.60703200 1.98796200
C -4.00756300 -1.38521600 -1.74845800
C -3.93890500 0.03276300 -1.60777300
C -5.09806300 0.78680500 -1.82536500
C -6.28976300 0.13154500 -2.15711000
C -6.33932300 -1.26613800 -2.27731800
C -5.18998600 -2.04421500 -2.07639000
C -1.84739800 -0.91456800 -1.14245100
C -2.57536200 0.31516500 -1.23617800
H -5.07609000 1.87480800 -1.72746200
H -7.19744000 0.71691800 -2.32483600
H -7.28156000 -1.75518300 -2.53552700
H -5.22728900 -3.13121000 -2.17509600
H -0.38189200 -0.67006600 -2.25391600
N -2.73663700 -1.91261400 -1.48162000
C -2.42253800 -3.30975300 -1.63416900
H -2.95832100 -3.93692400 -0.90093900
H -2.68511600 -3.66315800 -2.64551000
H -1.34483800 -3.44802700 -1.49044200
C -1.80958100 1.45411600 -0.97544000
H -2.23743000 2.46211900 -0.95412100
N -0.52402800 1.25576700 -0.71153500
C 0.31721500 2.34047300 -0.43169500
C 1.43472500 2.15136100 0.40524200
C 0.09452900 3.63526100 -0.93074400
C 2.24850800 3.21208000 0.77340300
H 1.63968100 1.14274900 0.75627100
C 0.91713400 4.70896100 -0.57463800

H -0.72010000 3.81293000 -1.63398300
C 1.99951300 4.50765100 0.29239100
H 3.10262800 3.06622900 1.43708200
H 0.71041700 5.69243800 -0.99632700
O 2.85497600 5.48258000 0.70488000
C 2.66419300 6.79208900 0.22991500
H 1.67909900 7.19888500 0.52459000
H 3.45268200 7.41025800 0.68008200
H 2.75083400 6.84809300 -0.87087800
H 1.31414100 -0.85088800 -3.32290600
C 2.27304200 -2.24655600 1.46865500
C 3.53358400 -1.67985300 1.71858900
C 2.12429600 -3.42691000 0.72179900
C 4.65402700 -2.30516600 1.15995800
C 3.27127300 -4.00835600 0.17286700
C 4.54325800 -3.45492200 0.36872100
H 5.63995300 -1.86396100 1.32753100
H 3.16751800 -4.91435300 -0.43012300
C 0.76163100 -4.02707300 0.50342100
H 0.26011200 -4.24400600 1.46019300
H 0.09879800 -3.32971300 -0.03095400
H 0.82421800 -4.95717400 -0.07762400
C 3.67533600 -0.41605900 2.52499300
H 2.92763400 0.33168100 2.22536400
H 3.52617900 -0.60030800 3.60146600
H 4.67309200 0.02206900 2.39240800
C 5.75665400 -4.05183100 -0.29560100
H 6.66403800 -3.90355500 0.30886400
H 5.63218300 -5.13007500 -0.47526300
H 5.93320300 -3.57319200 -1.27453300
Si 3.62039700 0.57152000 -2.02287300
C 2.82357400 2.06359700 -2.85785400
H 3.56167200 2.58255800 -3.49185800
H 1.98798800 1.74733800 -3.50345900
H 2.42643900 2.78385700 -2.13104600
C 4.70461000 -0.28681800 -3.32915900
H 4.09885500 -0.60831000 -4.19262000
H 5.49971500 0.38126600 -3.70218800
H 5.18439000 -1.18575400 -2.90672000
C 4.79261400 1.08330600 -0.63164400
H 5.52437200 1.82271400 -0.99682800
H 4.26335900 1.52703700 0.22064500
H 5.35166800 0.20418000 -0.27267000
H 2.76608800 -1.39076300 -0.67234100

TS5-M-SiMe$_3$ (triplet)

Fe 0.56215100 -0.54697500 0.20082700
C 1.29778200 0.02377400 2.12339100
H 0.70673000 -0.92455600 2.17370800
N 1.74005800 -1.77701600 -2.22229400
C 0.72653500 -1.03307700 -1.65960600
C 1.62096100 -1.85125300 -3.60818400
C 0.50622200 -1.14435400 -3.93413200
H 2.32397100 -2.40435900 -4.22342300
H 0.03638200 -0.95116900 -4.89388500
C -1.15897300 0.21349600 -2.63505800
C -0.95019000 1.60047900 -2.69680900
C -2.42740100 -0.34535900 -2.41865900
C -2.05955200 2.43663800 -2.53212500
C -3.50568700 0.52952200 -2.25237200
C -3.33834700 1.92009800 -2.29456800
H -1.90836600 3.51832700 -2.55180200
H -4.49660100 0.11001500 -2.06315500
C -2.60880000 -1.83715800 -2.34855400
H -2.45399300 -2.30729400 -3.33461700
H -1.88481900 -2.28613600 -1.65458600
H -3.61555000 -2.09555000 -1.99429400
C 0.42919300 2.16486400 -2.90603700
H 1.12751900 1.75109300 -2.16492100
H 0.82225700 1.91317100 -3.90537700
H 0.42767200 3.25690000 -2.79573300
C -4.50091800 2.84198000 -2.02775100
H -4.59363500 3.04839800 -0.94638900
H -4.37423400 3.81148500 -2.53218000
H -5.45404900 2.40121600 -2.35715800
N -0.02346800 -0.65561800 -2.74417300
C 1.23867300 3.53915400 0.21190900
C -0.17064900 3.36364100 0.32916100
C -1.00636000 4.47901100 0.19194600
C -0.43733900 5.73471700 -0.05544000
C 0.95252900 5.88746400 -0.16757900
C 1.81073700 4.78507000 -0.03431800
C 0.90321400 1.28891000 0.51173400
C -0.38628200 1.95158700 0.53426500
H -2.09087500 4.36985100 0.26885900
H -1.08474500 6.60858800 -0.16282700
H 1.37543800 6.87649900 -0.35905700

H 2.89226400 4.90669100 -0.12045900
N 1.85314800 2.28909300 0.33217600
C 3.24521300 2.08551800 0.01763900
H 3.50327900 2.59146000 -0.92904500
H 3.90941700 2.47923400 0.80612200
H 3.43334800 1.01385600 -0.09722200
C -1.55905900 1.19278000 0.65122200
H -2.53292900 1.69692700 0.66909700
N -1.45201500 -0.12571800 0.73261600
C -2.53976200 -0.96916700 0.92089900
C -2.27555200 -2.34485500 1.10680400
C -3.89074400 -0.57378500 0.92614000
C -3.29211900 -3.27318500 1.27638500
H -1.23300600 -2.67220200 1.10423500
C -4.92389200 -1.50146100 1.08903400
H -4.15411700 0.47500200 0.79071600
C -4.63537300 -2.86250500 1.26263000
H -3.07432400 -4.33364700 1.41727300
H -5.95432300 -1.14562200 1.07991300
O -5.57112600 -3.83989900 1.42509600
C -6.92918300 -3.47957800 1.41874500
H -7.23014800 -3.01773400 0.45980200
H -7.50370400 -4.40483800 1.56225300
H -7.17465600 -2.77660300 2.23631100
C 2.79296200 -0.30207400 2.15942800
H 3.02945900 -0.99294900 2.98726000
H 3.15019400 -0.78978300 1.23713800
H 3.40720200 0.59806900 2.31465800
C 2.83417000 -2.23898000 -1.42921100
C 4.06170700 -1.55727100 -1.50236100
C 2.63066500 -3.30523600 -0.53439300
C 5.09131000 -1.96144400 -0.64400100
C 3.68729900 -3.66727100 0.30765700
C 4.92079100 -3.00361700 0.27506100
H 6.04738700 -1.43232400 -0.68262500
H 3.54054300 -4.49046300 1.01224200
C 4.25431300 -0.41118600 -2.46159400
H 5.14435900 0.17642200 -2.19682700
H 4.38465100 -0.76700200 -3.49687100
H 3.38196200 0.25815800 -2.46458000
C 1.30821100 -4.02377200 -0.47502000
H 0.53017300 -3.38537200 -0.02539600
H 0.94419700 -4.28165800 -1.48102000
H 1.38323700 -4.94299800 0.12183100
C 6.01877400 -3.37630500 1.23752700
H 5.86923000 -2.87482400 2.20940200
H 6.03659000 -4.45976900 1.43036000
H 7.00777800 -3.07638700 0.86099300
Si 0.72341900 1.00463800 3.63558200
C -1.14780900 0.97421400 3.87304300
H -1.56411600 -0.02451200 3.66906800
H -1.39333700 1.24350800 4.91394900
H -1.65011400 1.68791800 3.20592600
C 1.35874200 2.78175100 3.60292900
H 2.43710900 2.81991300 3.37877000
H 0.83687300 3.38865000 2.84958600
H 1.20623900 3.25359700 4.58804000
C 1.48739100 0.16359400 5.16049700
H 1.14506600 0.66047200 6.08400600
H 1.19337000 -0.89772900 5.22362600
H 2.58850100 0.20828400 5.14794200

TS5-M-$SiMe_3$ (quintet)

Fe 0.39195200 -0.26873500 0.03712500
C 1.78234900 0.74140300 1.43706900
H 1.07132300 0.00857100 1.90742400
N 0.88859000 -3.19728700 -0.79991700
C 0.04403700 -2.12737100 -0.72925700
C 0.35706200 -4.21836600 -1.58041300
C -0.85690200 -3.77970900 -2.00976200
H 0.88644300 -5.15083900 -1.75092500
H -1.60699000 -4.24849700 -2.63903100
C -2.19101400 -1.68430000 -1.69117900
C -2.08917100 -0.54071200 -2.50409900
C -3.38626400 -2.04069900 -1.04652200
C -3.23281500 0.24940100 -2.65123500
C -4.50561400 -1.22456300 -1.23911500
C -4.44513000 -0.07336000 -2.02873900
H -3.16901000 1.15114500 -3.26633400
H -5.43445600 -1.47420600 -0.72194500
C -3.46138500 -3.24788500 -0.14895600
H -3.47748700 -4.18944800 -0.72329500
H -2.59353300 -3.29549500 0.52581000
H -4.36974100 -3.21282200 0.46680500
C -0.79911000 -0.14857700 -3.17462100
H -0.14758500 0.41478900 -2.48429200
H -0.23034000 -1.02614700 -3.51626800

H -0.99395100 0.49910700 -4.04086500
C -5.64792300 0.81647900 -2.19882200
H -5.41520300 1.84867100 -1.89303900
H -5.97407400 0.85188700 -3.25171900
H -6.49422200 0.46851400 -1.58979900
N -1.03018700 -2.50548000 -1.48345700
C 2.57114900 3.07653300 -1.46119000
C 1.25185100 3.54132800 -1.19448700
C 0.91272300 4.85521500 -1.53193200
C 1.87285700 5.67949900 -2.13715500
C 3.15996500 5.20025100 -2.40905200
C 3.52468600 3.88529800 -2.07516900
C 1.48005000 1.29959700 -0.48022100
C 0.54519200 2.42927900 -0.58953000
H -0.09226800 5.23586000 -1.33234900
H 1.61174800 6.70674100 -2.40335300
H 3.89385400 5.85581200 -2.88429200
H 4.53127100 3.51664200 -2.28254300
N 2.68013000 1.74438200 -1.05059600
C 3.72130800 0.88864500 -1.56565500
H 3.81749800 1.00488200 -2.66145500
H 4.70441200 1.10777200 -1.11462600
H 3.46831600 -0.15277100 -1.34485900
C -0.77269300 2.26838900 -0.19438700
H -1.47380700 3.10998200 -0.24364000
N -1.15307600 1.07687700 0.31358200
C -2.41437000 0.84217200 0.83354800
C -2.61168700 -0.33192000 1.59982400
C -3.53713200 1.67524300 0.65203800
C -3.84936400 -0.66446900 2.12884700
H -1.75351300 -0.98939000 1.76138600
C -4.78828600 1.34527800 1.17963300
H -3.44837200 2.58910600 0.06375100
C -4.96181000 0.16228000 1.90860700
H -3.98734800 -1.57649700 2.71303600
H -5.62747500 2.01607100 0.99489800
O -6.15225900 -0.26578500 2.42859300
C -7.28732500 0.53962000 2.24695400
H -7.53186300 0.67713000 1.17635500
H -8.12537500 0.02560700 2.73765800
H -7.16484700 1.53921900 2.70426100
C 3.17713500 0.11893200 1.45456600
H 3.43109100 -0.27863500 2.45110500
H 3.27672300 -0.71809700 0.75002800
H 3.94615000 0.86194200 1.19438600
C 2.17680800 -3.21553000 -0.17528400
C 3.31345100 -2.99144700 -0.96937200
C 2.26136000 -3.42687200 1.21125400
C 4.56260000 -2.98990500 -0.33776500
C 3.53198200 -3.41807200 1.79524400
C 4.69166500 -3.19724000 1.04114700
H 5.45696000 -2.80607400 -0.93892200
H 3.61631300 -3.57506200 2.87387600
C 3.18037800 -2.73509900 -2.44818200
H 4.13397600 -2.39619200 -2.87519800
H 2.86952400 -3.64141800 -2.99311100
H 2.41903000 -1.96512900 -2.64737500
C 1.02000100 -3.61686100 2.04144300
H 0.45563300 -2.67213400 2.10833400
H 0.34004100 -4.35835200 1.59438800
H 1.27104200 -3.94019800 3.06075100
C 6.03922900 -3.12768400 1.71117200
H 6.21432800 -2.11750500 2.11994900
H 6.11003200 -3.83524900 2.55107500
H 6.85529100 -3.34418300 1.00626000
Si 1.65802400 2.24353200 2.58717600
C -0.11641700 2.77341900 2.94037400
H -0.77792300 1.90521000 3.08465700
H -0.14145100 3.38145400 3.86009400
H -0.53327200 3.37393600 2.12090300
C 2.69087800 3.69158000 1.96225700
H 3.70081600 3.36666800 1.66550900
H 2.23100600 4.18202900 1.09362300
H 2.80229500 4.44111900 2.76367900
C 2.40268800 1.70467600 4.25383900
H 2.30139300 2.51458600 4.99604000
H 1.88762800 0.81663100 4.65781900
H 3.47480000 1.46450000 4.16933900

TS5-anti/M-SiMe$_3$ (singlet)

Fe 0.18122800 -0.41653700 -0.34464500
C -0.09141500 -1.35466100 -2.20935700
H 0.14421800 -0.45654600 -2.80045500
N 1.41439600 -0.46691700 2.37752700
C 0.33436800 -0.23583800 1.54041300
C 1.10617400 -0.23157800 3.71598200
C -0.19142400 0.16028000 3.74422300
H 1.83713900 -0.36637900 4.50720400

H -0.83950000 0.44016900 4.56907900
C -1.99210000 0.50293300 2.08544300
C -2.94965800 -0.52223400 2.01028700
C -2.32405800 1.84744300 1.87045300
C -4.25938700 -0.17807800 1.66569800
C -3.64800300 2.14377200 1.52279300
C -4.62588400 1.14866800 1.41196900
H -5.00446000 -0.97029600 1.56151800
H -3.91857000 3.18574200 1.32814200
C -1.30663400 2.94517900 2.03223400
H -1.29947600 3.32083000 3.07061000
H -0.29289100 2.60326100 1.79514800
H -1.53245700 3.79325100 1.37136500
C -2.58229400 -1.94514300 2.33363000
H -1.60446900 -2.20966200 1.91341400
H -2.52111400 -2.09473500 3.42586500
H -3.33499400 -2.64047000 1.93828900
C -6.04704700 1.49535400 1.04889600
H -6.09027500 2.37857900 0.39330300
H -6.53757300 0.65919200 0.53085400
H -6.64022400 1.72807100 1.95072800
N -0.64407000 0.16031600 2.43090400
C -3.49689600 -2.17439800 -1.13613000
C -3.69770400 -0.79333800 -1.43341600
C -4.98226100 -0.34653900 -1.75217000
C -6.04605200 -1.26044400 -1.77387000
C -5.83267700 -2.61307000 -1.48124300
C -4.54903500 -3.08854900 -1.16248600
C -1.42448700 -1.17922500 -0.91000000
C -2.41388600 -0.15858700 -1.26103600
H -5.15849900 0.70928500 -1.97077300
H -7.05169900 -0.91369700 -2.02463600
H -6.67118800 -3.31335900 -1.50742700
H -4.38881000 -4.14623000 -0.94398200
N -2.16008800 -2.37585300 -0.79390400
C -1.61732100 -3.66435100 -0.47333100
H -2.31584400 -4.23125900 0.16271800
H -1.40511200 -4.27719500 -1.37106900
H -0.68225200 -3.53506100 0.08518000
C -1.94851300 1.14025100 -1.23250500
H -2.57186200 2.00235100 -1.48831200
N -0.67111800 1.28631400 -0.83404200
C 0.02936600 2.47785900 -0.93294300
C 1.35803000 2.50292200 -0.44889400
C -0.46672100 3.67330300 -1.49095400
C 2.13782500 3.64924600 -0.50082400
H 1.76424000 1.59095300 -0.00840900
C 0.30938600 4.83579500 -1.53521200
H -1.47352900 3.70679700 -1.90808200
C 1.62010400 4.83801200 -1.03833900
H 3.16156600 3.65345100 -0.12223100
H -0.12297300 5.73573000 -1.97323900
O 2.45276700 5.91982300 -1.03934500
C 1.97571500 7.13230400 -1.56311300
H 1.09278000 7.50391200 -1.00990200
H 2.78947600 7.86379400 -1.46389500
H 1.70436200 7.04431000 -2.63184500
H -0.72804400 -1.99271700 -2.82844200
C 1.12106400 -2.08969300 -1.64849500
H 1.33063700 -1.86609400 -0.53865200
C 2.69578200 -0.92679800 1.94737000
C 2.87786400 -2.30687500 1.72961900
C 3.75534400 -0.01497000 1.81937300
C 4.14141200 -2.75163000 1.33554400
C 5.00336600 -0.50792200 1.40981300
C 5.21497700 -1.86547700 1.15559900
H 4.29587000 -3.82143900 1.17079800
H 5.83167500 0.19531700 1.28807100
C 1.74029400 -3.26686500 1.95228700
H 2.02837600 -4.29381100 1.68883200
H 1.41374200 -3.25634200 3.00472200
H 0.86321300 -2.98018300 1.35381900
C 3.58615800 1.44324400 2.15655800
H 4.25022200 2.06831200 1.54276800
H 2.55507100 1.78677600 2.01274000
H 3.84559400 1.62802700 3.21314600
C 6.54901400 -2.37147200 0.67244200
H 6.83779400 -3.30093400 1.18758500
H 6.50904400 -2.59942100 -0.40652700
H 7.34524800 -1.62930600 0.82767800
H 0.93932200 -3.17631400 -1.61467000
Si 2.74307700 -1.74963700 -2.58971900
C 3.42849200 -0.08982800 -2.02880100
H 4.37146700 0.14494900 -2.54943900
H 2.72194600 0.73030200 -2.23116600
H 3.63633000 -0.10109200 -0.94770500
C 3.97692200 -3.13601000 -2.25110600
H 3.59764900 -4.09962000 -2.62947300

H 4.93958800 -2.93543700 -2.75006300
H 4.16587500 -3.24147600 -1.17377300
C 2.36865700 -1.71941100 -4.43801800
H 3.29676300 -1.58986400 -5.01930800
H 1.89707600 -2.66163500 -4.76268500
H 1.68741400 -0.89500300 -4.69950700

TS5-anti/M-SiMe$_3$ (triplet)

N 0.22897400 0.10902000 -2.54628300
C 1.60950500 -0.15360800 -2.25908200
C 2.46361900 0.93667000 -2.02675700
C 2.06923900 -1.47792400 -2.26584100
C 3.80736800 0.66619800 -1.75635000
C 4.29945100 -0.64660800 -1.71485700
H 4.48143200 1.50298500 -1.55906400
C -0.77138500 0.25138400 -1.61454000
Fe -0.68608700 -0.07661700 0.35462500
C -2.17556600 1.20449400 1.10183800
H -3.18017800 0.95135300 0.75726100
C -1.56519800 0.52631900 -3.75315100
H -2.31924600 0.71805100 -4.51033900
C -0.23553500 0.27404300 -3.84528000
H 0.42664600 0.19691200 -4.70217400
C 3.42372200 -1.70086900 -1.99029800
H 3.79010000 -2.72935200 -1.95508200
C -1.60960900 0.50608100 2.20270600
C 1.14170300 -2.62338300 -2.56982800
H 0.15699200 -2.46485600 -2.11178100
H 0.98716700 -2.73132400 -3.65736900
H 1.55165900 -3.56802700 -2.18806800
C 1.93768900 2.34554100 -2.07100600
H 1.64524400 2.62997700 -3.09611100
H 1.04753900 2.45094300 -1.43620600
H 2.69067800 3.05946000 -1.71253300
C 5.73320100 -0.91466100 -1.33406200
H 6.41040200 -0.14201000 -1.72971400
H 5.85040600 -0.91725100 -0.23569700
H 6.07365400 -1.89470300 -1.69924700
N -1.87208400 0.51433600 -2.39610500
C 1.05073400 -3.96658600 0.66322400
C 2.15668400 -3.09150500 0.86087100
C 3.43995200 -3.64093400 0.98955700
C 3.59667000 -5.02880500 0.92036000
C 2.49218600 -5.87506100 0.72400900
C 1.19968400 -5.35095800 0.59164500
C 0.21104800 -1.83568700 0.56461900
C 1.61298500 -1.75598300 0.82930900
H 4.30858300 -2.99352900 1.13230000
H 4.59432800 -5.46426300 1.01746800
H 2.64175700 -6.95596800 0.67031600
H 0.34318700 -6.00906500 0.43167700
H -0.52773700 -0.86192600 1.67250900
N -0.09779900 -3.18086600 0.52371300
C -1.39546200 -3.73665200 0.23676400
H -1.43032600 -4.18929100 -0.77049300
H -1.66328500 -4.51116800 0.97337200
H -2.14015700 -2.93672400 0.29070900
C 2.21509300 -0.50099900 1.06287000
H 3.28135700 -0.47071200 1.33111100
N 1.49565100 0.59110100 0.98476300
C 2.04006500 1.86041300 1.18169000
C 1.14886600 2.95005000 1.29986100
C 3.41651900 2.15237700 1.21998400
C 1.60289900 4.25223500 1.45035600
H 0.07945100 2.74777600 1.25722400
C 3.88460900 3.46125400 1.36885300
H 4.14841900 1.35242200 1.11062400
C 2.98023200 4.52614200 1.48497200
H 0.90537000 5.08717300 1.54076200
H 4.96052100 3.63604400 1.38580200
O 3.33610900 5.83243400 1.62844400
C 4.70250300 6.15882900 1.67310100
H 5.22596400 5.87476200 0.74120400
H 4.76421700 7.24861000 1.79628300
H 5.21579100 5.67347500 2.52370400
H -1.88791600 2.24182900 0.90076500
C -3.17608900 0.82725000 -1.89733600
C -3.49627600 2.17289000 -1.65926800
C -4.08607300 -0.21096400 -1.65543400
C -4.76382900 2.46231000 -1.14435300
C -5.33966200 0.12418600 -1.13153400
C -5.69381400 1.45253200 -0.86294100
H -5.02494000 3.50400600 -0.93903700
H -6.05439700 -0.67558600 -0.92012800
C -3.70812000 -1.63850900 -1.94515800
H -3.63002200 -1.81893300 -3.03045200
H -2.72580900 -1.87990400 -1.51627400

H -4.45101500 -2.33730400 -1.53659200
C -2.47671800 3.25280100 -1.90272300
H -1.57074000 3.06845800 -1.30389800
H -2.15711100 3.28205800 -2.95670200
H -2.87492600 4.24133500 -1.63613100
C -7.02787900 1.78381700 -0.24421700
H -7.79957800 1.05338400 -0.53001400
H -6.95932400 1.77362700 0.85752500
H -7.37385400 2.78551800 -0.54015600
Si -2.63075000 -0.72980200 3.18186200
H -0.83167600 1.02744000 2.77786000
C -1.55491500 -2.13712600 3.83279500
H -0.69809800 -1.73747700 4.40015200
H -2.12716900 -2.79514800 4.50758800
H -1.14577800 -2.75264500 3.01744900
C -4.00509700 -1.41389200 2.07687200
H -4.74851100 -0.63565800 1.84387500
H -3.61019100 -1.78044100 1.11792200
H -4.52814200 -2.24818200 2.57229100
C -3.42852700 0.16188500 4.64657200
H -4.07294300 -0.51732600 5.22953600
H -2.66265400 0.56693600 5.32845400
H -4.04726800 1.00497500 4.29815500

TS5-anti/M-SiMe$_3$ (quintet)

Fe 0.06293800 0.47587600 0.01861800
C 0.12414800 1.92712500 1.67942900
H 0.16671800 1.11460000 2.42018600
N 1.81785100 -0.25529600 -2.53354200
C 0.64692300 -0.23072800 -1.84115500
C 1.64324700 -0.75434300 -3.81919300
C 0.31838300 -1.05449200 -3.93525300
H 2.46666900 -0.84811900 -4.52094200
H -0.25423000 -1.46555000 -4.76132200
C -1.64864400 -0.90606700 -2.35973300
C -2.52444300 0.18760800 -2.46889800
C -2.04310200 -2.13867300 -1.82070900
C -3.82365600 0.02941400 -1.98387200
C -3.35801600 -2.24817700 -1.35270200
C -4.25316600 -1.17405100 -1.40936500
H -4.50723100 0.88119900 -2.01887100
H -3.67858700 -3.19375500 -0.90838500
C -1.07609300 -3.28903200 -1.73414200
H -0.75678000 -3.62206400 -2.73583200
H -0.17147500 -3.00594500 -1.17679200
H -1.52515600 -4.14268300 -1.21158100
C -2.05999900 1.49084700 -3.05845500
H -1.20122600 1.87903400 -2.49197600
H -1.74117700 1.37110100 -4.10728900
H -2.85957600 2.24266800 -3.02273200
C -5.64309000 -1.28034600 -0.83937900
H -5.83026200 -2.27381500 -0.40655900
H -5.79070700 -0.52392800 -0.05183000
H -6.40761400 -1.09476500 -1.61174400
N -0.26657400 -0.72826100 -2.72241400
C -3.37827000 2.91355500 0.63875400
C -3.66203400 1.64848600 1.23731800
C -4.93026000 1.42730800 1.78315300
C -5.89560400 2.44462100 1.72532300
C -5.59998900 3.67836000 1.13262700
C -4.33147700 3.93044200 0.58387900
C -1.46583200 1.65475400 0.37936200
C -2.46153300 0.84940300 1.08550600
H -5.16801900 0.47016000 2.25476500
H -6.88769800 2.27309900 2.15031700
H -6.36241400 4.46072300 1.09984400
H -4.10327400 4.89812500 0.13208300
N -2.08101700 2.88527500 0.13028300
C -1.46115400 3.99045800 -0.54353100
H -2.13616500 4.42727200 -1.29993800
H -1.16573400 4.79815500 0.15292000
H -0.55667800 3.63003200 -1.05292000
C -2.14896200 -0.44962500 1.45581100
H -2.86651200 -1.05897000 2.01884200
N -0.92062100 -0.93603300 1.15892100
C -0.54099500 -2.23664200 1.43366500
C 0.84021900 -2.55358500 1.39671200
C -1.42821900 -3.30106300 1.69840000
C 1.30188300 -3.84798900 1.58768200
H 1.54849800 -1.74156100 1.21519200
C -0.96790700 -4.60677400 1.90057600
H -2.50341800 -3.11911600 1.71184400
C 0.40148400 -4.89669900 1.84015900
H 2.36919600 -4.07711000 1.55435600
H -1.69708500 -5.39478200 2.09173000
O 0.94568600 -6.13845100 2.01297800
C 0.08470500 -7.21378200 2.28101600

H -0.63415900 -7.38573700 1.45745300
H 0.71494500 -8.10702800 2.39207500
H -0.48868600 -7.06380500 3.21513800
H -0.42629100 2.76357400 2.12041900
C 1.49663000 2.37441500 1.15872500
H 1.90528400 1.72240300 0.34879000
C 3.06696300 0.14212600 -1.95387300
C 3.42383200 1.49835700 -1.97673900
C 3.87292000 -0.83904700 -1.35124300
C 4.63596900 1.86402400 -1.37853500
C 5.06889900 -0.42219100 -0.75792100
C 5.46679100 0.92202300 -0.75951400
H 4.93338300 2.91548600 -1.39319700
H 5.70539600 -1.16895400 -0.27575500
C 2.51023500 2.52282400 -2.59563100
H 2.97892800 3.51620600 -2.60546200
H 2.23785700 2.25542200 -3.62849300
H 1.56733600 2.59294400 -2.02883700
C 3.44622900 -2.28274000 -1.33525300
H 2.45931200 -2.40148700 -0.86274300
H 3.36173800 -2.69057000 -2.35543500
H 4.16539700 -2.90018500 -0.78029000
C 6.73948500 1.33931800 -0.06980500
H 6.59689400 1.35889900 1.02395100
H 7.56013000 0.63613400 -0.27941900
H 7.05780700 2.34537400 -0.37835300
H 1.42584500 3.37333900 0.69750200
Si 2.84208800 2.32927700 2.50467300
C 3.38692100 0.52856700 2.66277700
H 4.14920400 0.39769100 3.44819500
H 2.53297400 -0.12179300 2.91380800
H 3.81269200 0.17188500 1.71031800
C 4.30868500 3.41440400 2.01011700
H 4.01157300 4.47531500 1.96552600
H 5.13281100 3.32818600 2.73782900
H 4.69651200 3.12992400 1.02158700
C 2.14125900 2.95788200 4.13813000
H 2.91058500 2.94515900 4.92787600
H 1.77739500 3.99404300 4.03881400
H 1.29630400 2.33707300 4.47397200

product-M-Ph

C 2.78047000 -2.11450400 0.12446100
C 1.40060800 -2.40573200 -0.04071100
C 1.02014500 -3.70675800 -0.40903400
C 2.00818400 -4.7030600 -0.60156900
C 3.37235300 -4.35997900 -0.43090700
C 3.77800800 -3.07730700 -0.06431000
C 0.68267700 -1.17868100 0.23899800
H -0.03303700 -3.96445500 -0.54185500
H 1.72294100 -5.68526200 -0.88796600
H 4.12437200 -5.13684800 -0.58690500
H 4.83468800 -2.83957400 0.07004300
N 2.89619500 -0.77901800 0.48515200
C 4.17464800 -0.14888700 0.73931200
H 4.66178100 -0.60001500 1.61932600
H 4.83791600 -0.27293400 -0.13115500
H 4.04163900 0.92225000 0.91103200
C -0.75485000 -1.03637700 0.17813900
H -1.29358500 -1.93977100 -0.16825600
N -1.40637200 0.02422900 0.50247800
C -2.79135100 0.09678500 0.32663100
C -3.54160400 0.85663600 1.24856700
C -3.47907200 -0.50810900 -0.73916700
C -4.91965000 0.96668100 1.13559900
H -3.00932100 1.34754100 2.06568300
C -4.86626100 -0.39086900 -0.86970400
H -2.91967900 -1.05371700 -1.50204700
C -5.59907000 0.34117500 0.07496700
H -5.50444900 1.54286500 1.85512500
H -5.35949700 -0.86247500 -1.71951200
O -6.94634300 0.51454400 0.03916600
C -7.68127000 -0.08871000 -0.99781000
H -7.57576300 -1.18907700 -0.99112400
H -8.73544900 0.16869500 -0.82861800
H -7.37662000 0.28736600 -1.99170300
C 1.64057500 -0.20892900 0.55326000
C 1.39412000 1.22847100 0.92889500
H 0.31956600 1.36556500 0.72987900
C 2.14498900 2.20214600 0.02570400
C 2.82867100 3.32249200 0.51739300
C 2.13477500 1.98737600 -1.36321700
C 3.48691900 4.20036600 -0.35140700
H 2.85669900 3.52006600 1.58979000
C 2.79064300 2.85964700 -2.23117800
H 1.60379900 1.11941100 -1.76233400
C 3.47330600 3.97243000 -1.72788800
H 4.01415100 5.06706700 0.05523900

H 2.76851300 2.67154200 -3.30754600
H 3.98918800 4.65642700 -2.40585500
C 1.57826800 1.44984000 2.43830900
H 1.24257200 2.45588300 2.73116600
H 0.96900400 0.72041700 2.99079300
H 2.62517000 1.33281900 2.76006900

product-anti/M-SiMe$_3$

C -3.77514100 -1.46968100 0.06270800
C -2.52997100 -2.12765000 -0.12519800
C -2.52821100 -3.48848200 -0.47271800
C -3.74583600 -4.15144100 -0.61604700
C -4.96921400 -3.47993200 -0.42204800
C -5.00072900 -2.12808100 -0.07955500
C -1.49940200 -1.13634300 0.10253500
H -1.58833000 -4.02255200 -0.62992600
H -3.75451500 -5.21041500 -0.88455000
H -5.90794000 -4.02576800 -0.54122100
H -5.94947900 -1.60995800 0.07221200
N -3.51223100 -0.14509700 0.39073400
C -4.55155100 0.82759600 0.64224500
H -5.26692300 0.84384500 -0.19509000
H -5.10490800 0.59137800 1.56660400
H -4.11534100 1.82777600 0.73999000
C -0.07744100 -1.35376000 -0.00560400
H 0.23141500 -2.40866500 -0.14370800
N 0.80154100 -0.41296400 0.02301600
C 2.16659800 -0.70760200 -0.00435700
C 3.02687800 0.16821100 -0.70089900
C 2.74381000 -1.80256600 0.66042300
C 4.39326200 -0.06356700 -0.76116900
H 2.58784600 1.02751500 -1.21153100
C 4.12213900 -2.03621200 0.61740000
H 2.11020700 -2.46682400 1.25207500
C 4.95732300 -1.17044700 -0.10205100
H 5.05887400 0.60484700 -1.31057500
H 4.53140100 -2.88826100 1.16002400
O 6.30558100 -1.30821000 -0.20544400
C 6.92692100 -2.39833700 0.43056300
H 6.55229100 -3.36598100 0.04926500
H 8.00046400 -2.32143800 0.21191000
H 6.78269000 -2.37322000 1.52634800
C -2.14831200 0.05909600 0.43085100
C -1.52105400 1.37610200 0.74871900
H -0.59088700 1.16148700 1.29216000
C -1.16719500 2.19591600 -0.50899900
H -2.07912300 2.43350400 -1.08575600
H -0.53728600 1.56408000 -1.15595200
H -2.16891100 1.95834400 1.42395100
Si -0.20189200 3.78692400 -0.14849000
C -1.33781100 5.03915600 0.70048800
H -1.73133000 4.64240600 1.65118400
H -0.79951800 5.97358200 0.93045500
H -2.19780700 5.29714400 0.06012900
C 1.25665200 3.39281300 0.98134200
H 1.85897500 2.56432600 0.57676500
H 1.91293800 4.27032700 1.10252900
H 0.91478000 3.09100900 1.98467700
C 0.41795200 4.49820500 -1.78372300
H 0.97250800 5.43800500 -1.62612400
H 1.09450300 3.78912400 -2.28880800
H -0.41792400 4.71158600 -2.47031500

8. NMR spectra

^{1}H-NMR spectrum of **3a**

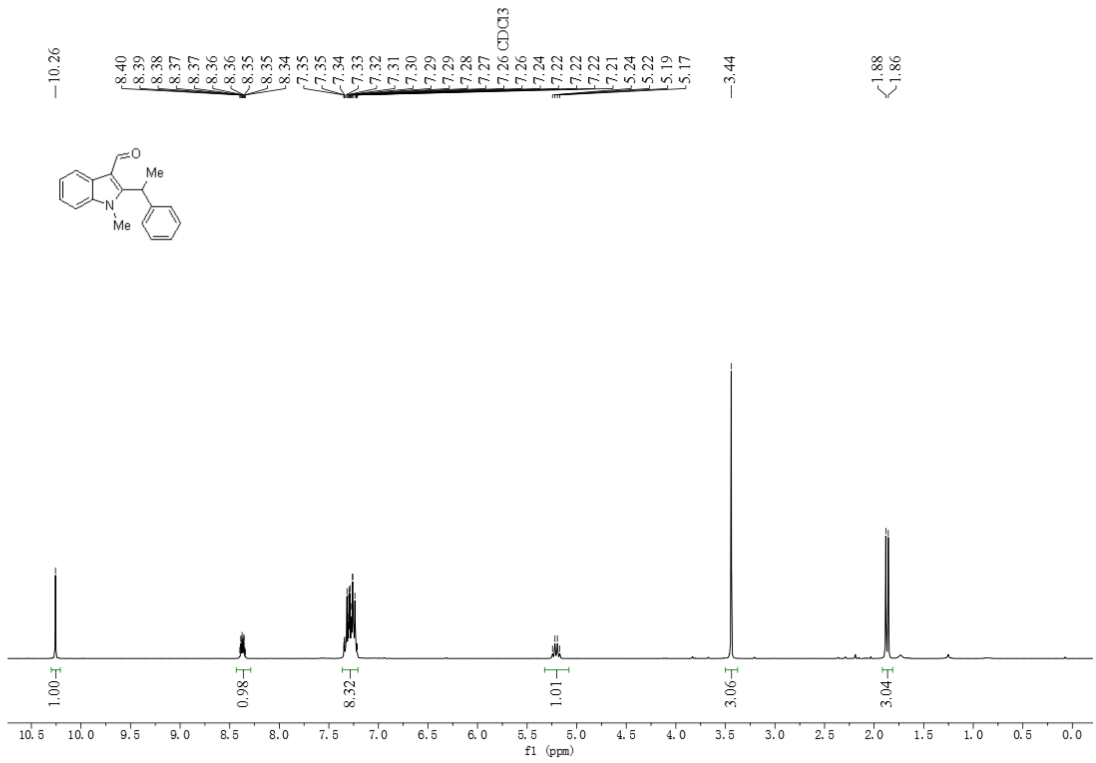

^{13}C-NMR spectrum of **3a**

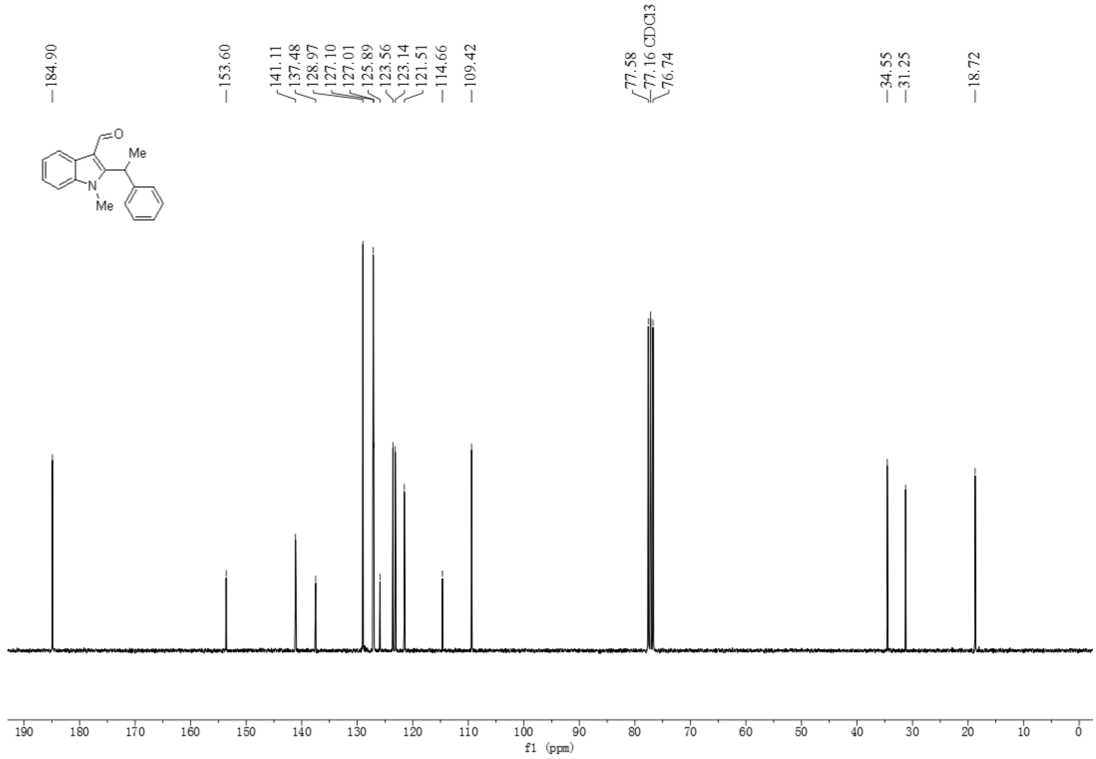

^{1}H-NMR spectrum of **3b**

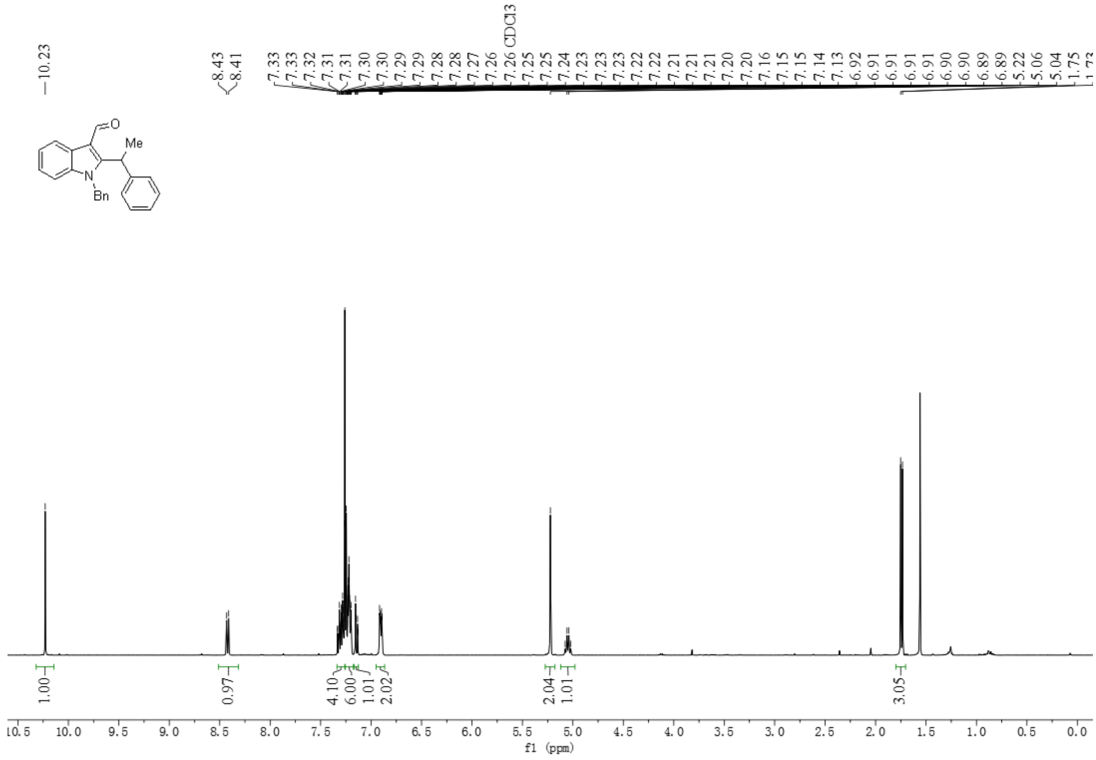

^{13}C-NMR spectrum of **3b**

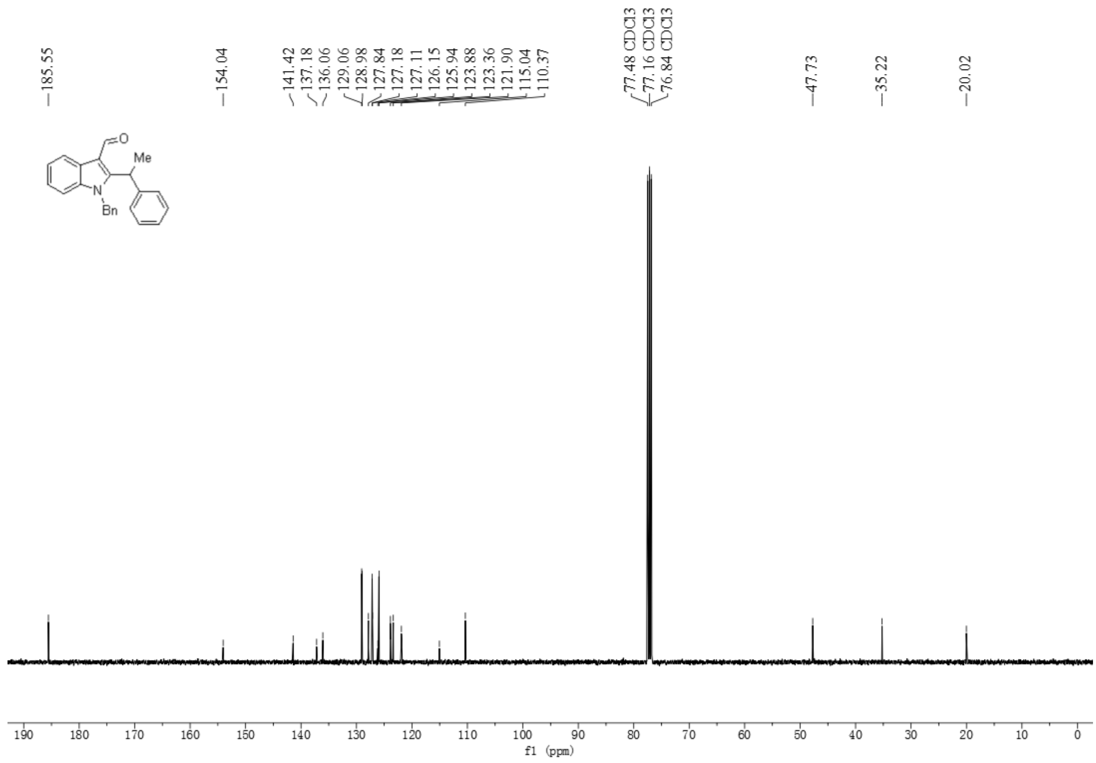

^{1}H-NMR spectrum of **3c**

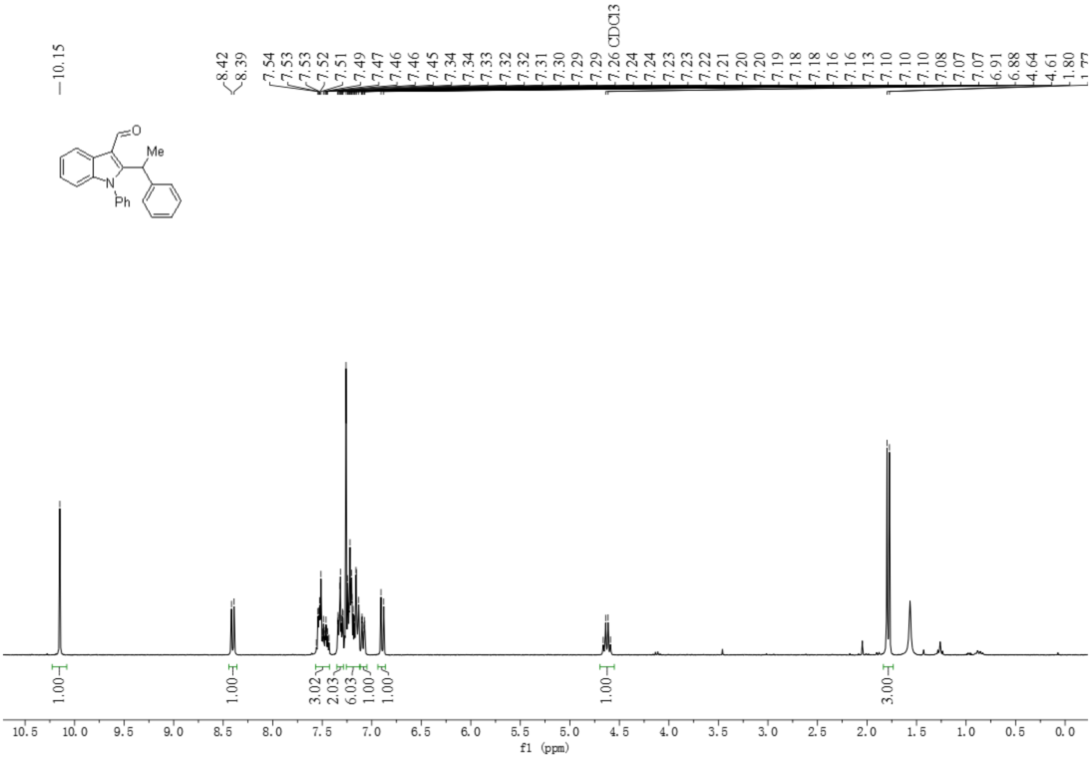

^{13}C-NMR spectrum of **3c**

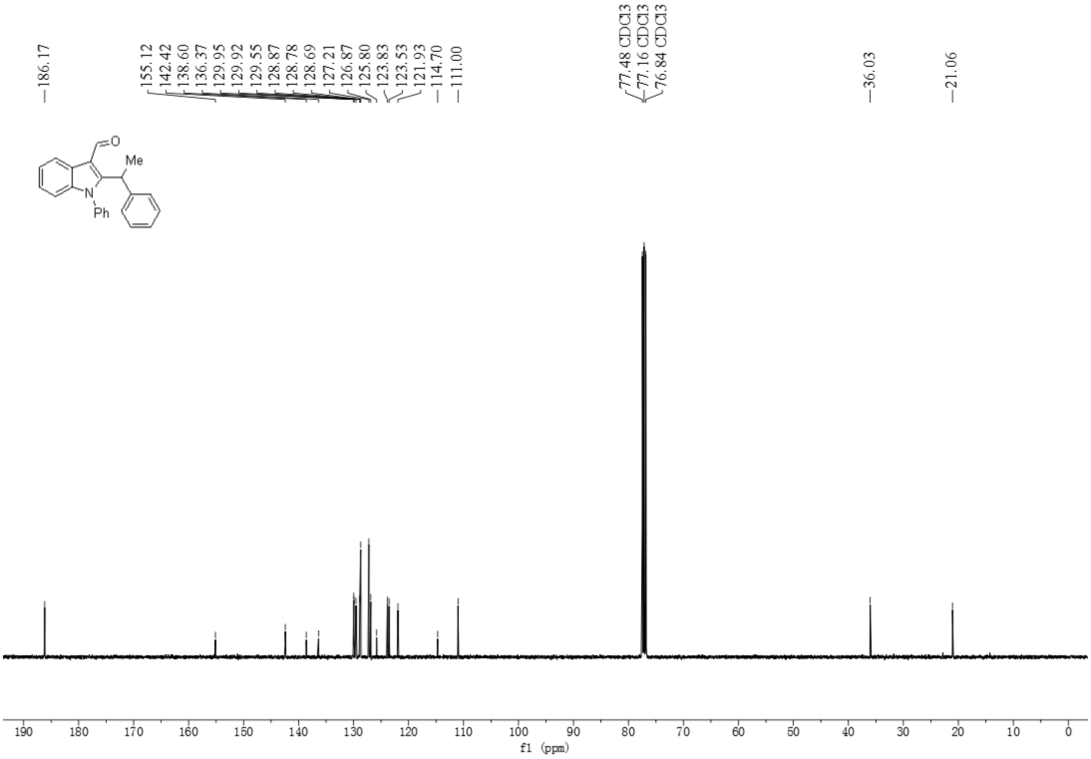

^{1}H-NMR spectrum of **3d**

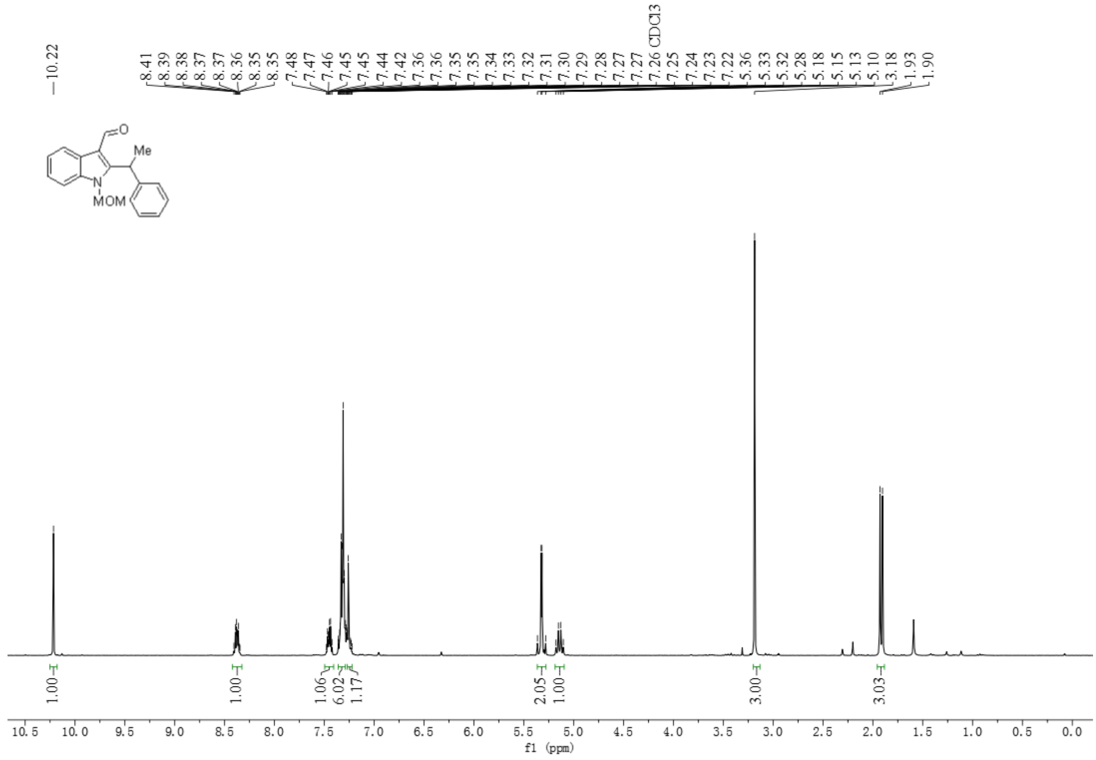

^{13}C-NMR spectrum of **3d**

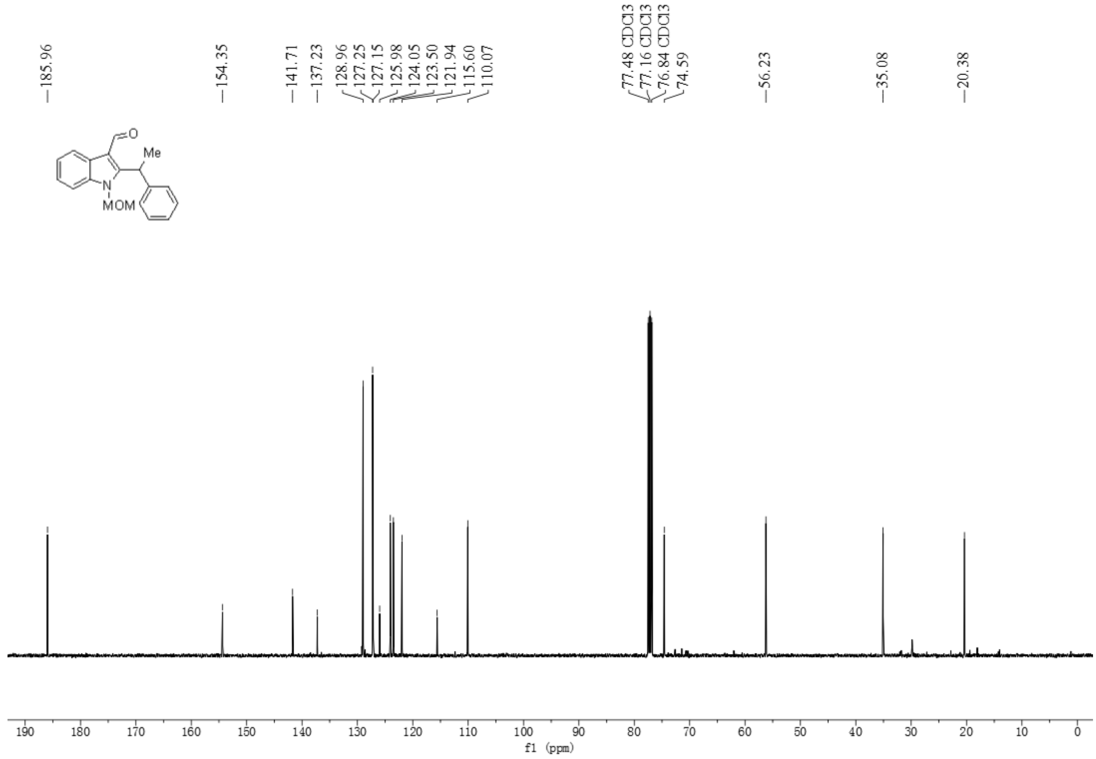

^{1}H-NMR spectrum of **3e**

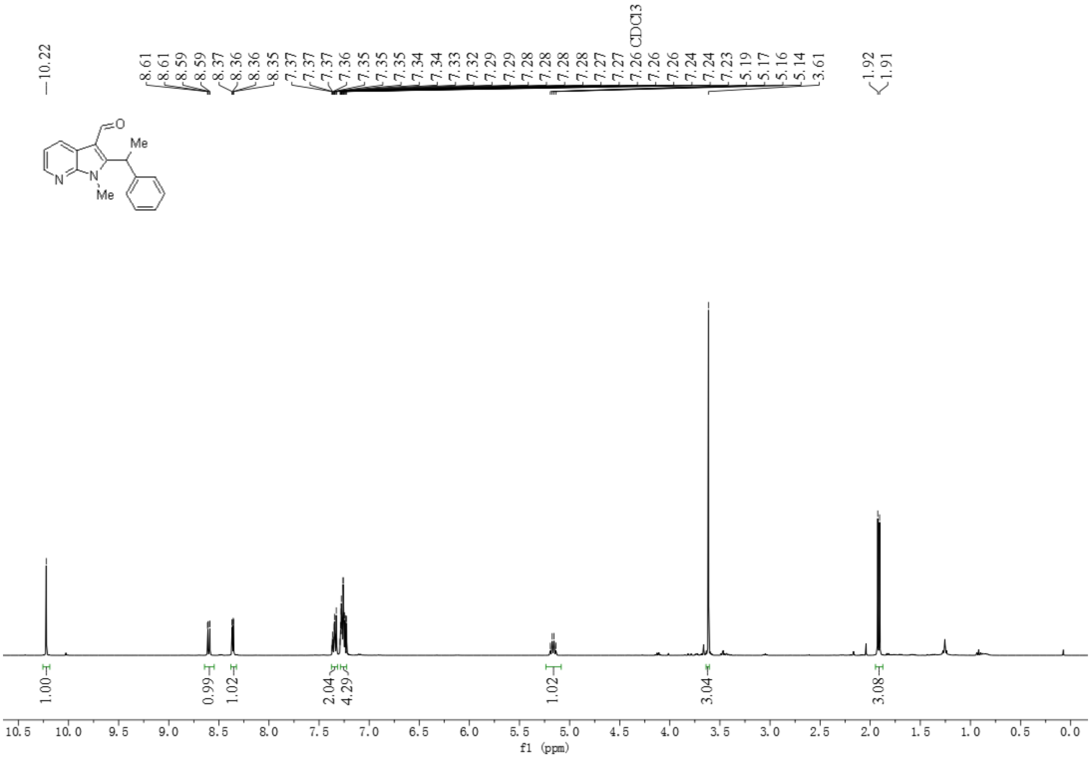

^{13}C-NMR spectrum of **3e**

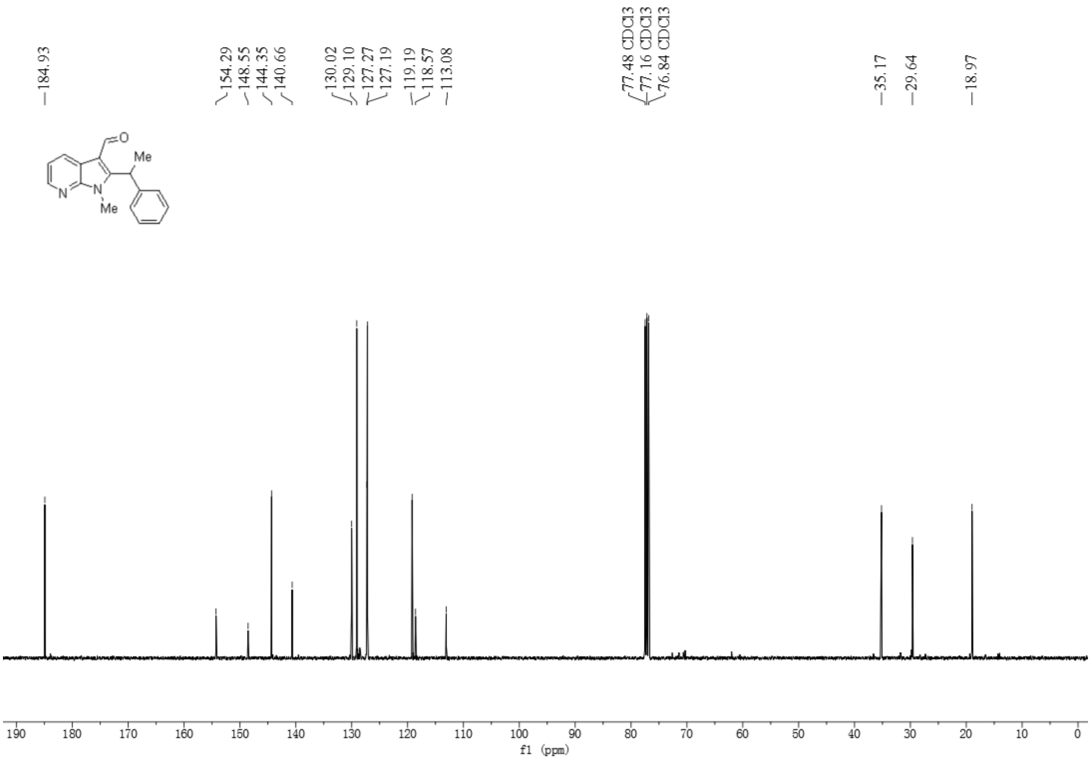

^{1}H-NMR spectrum of **3f**

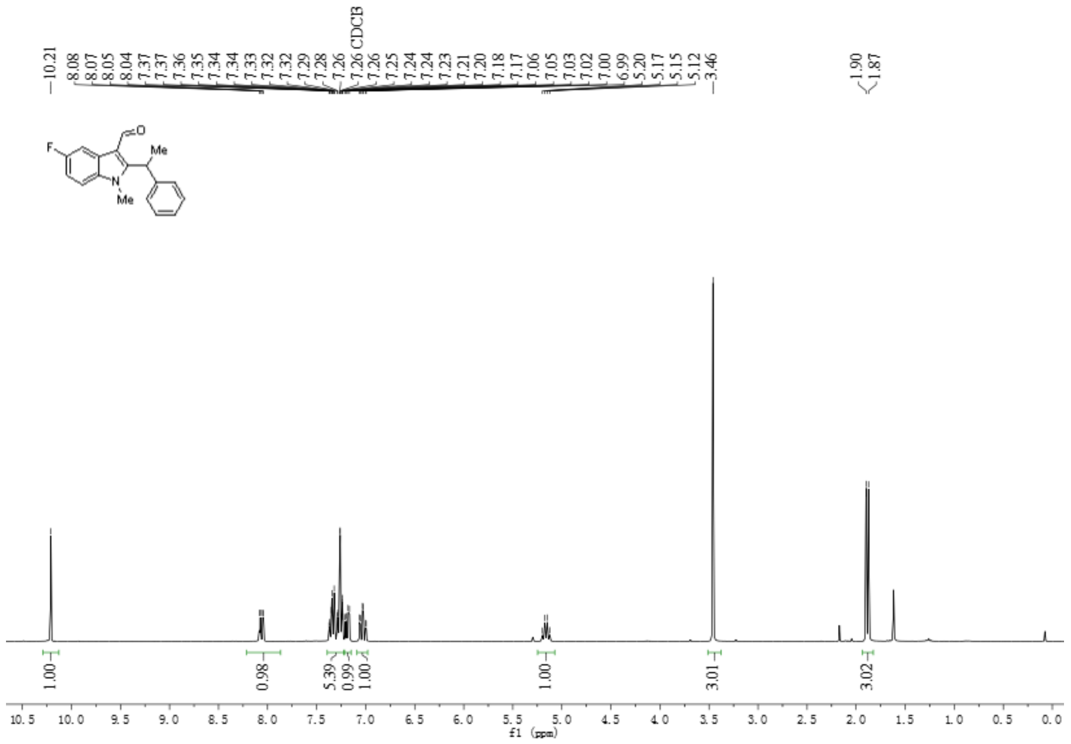

^{13}C-NMR spectrum of **3f**

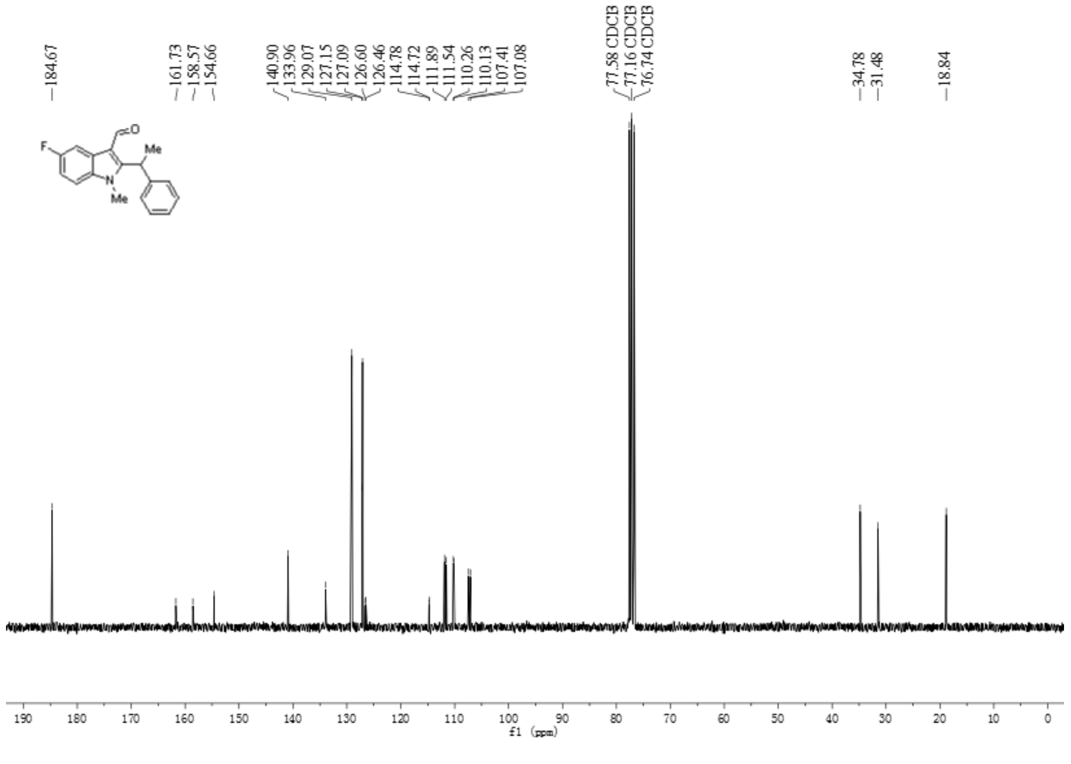

^{19}F-NMR spectrum of **3f**

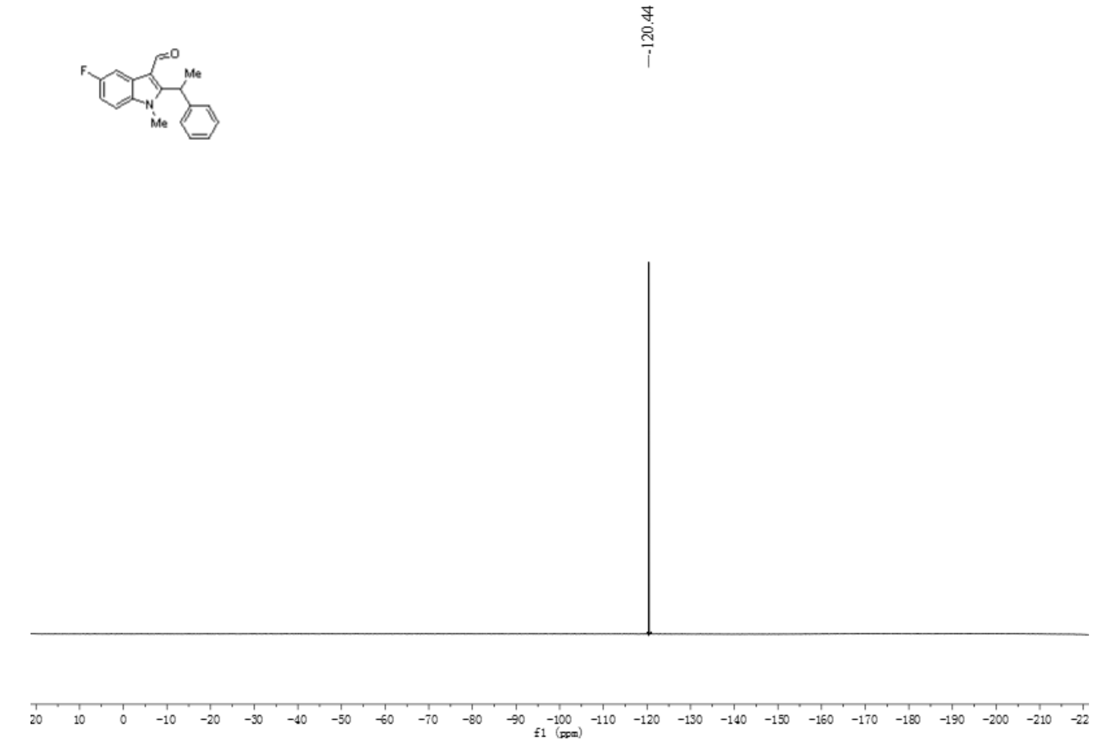

^{1}H-NMR spectrum of **3g**

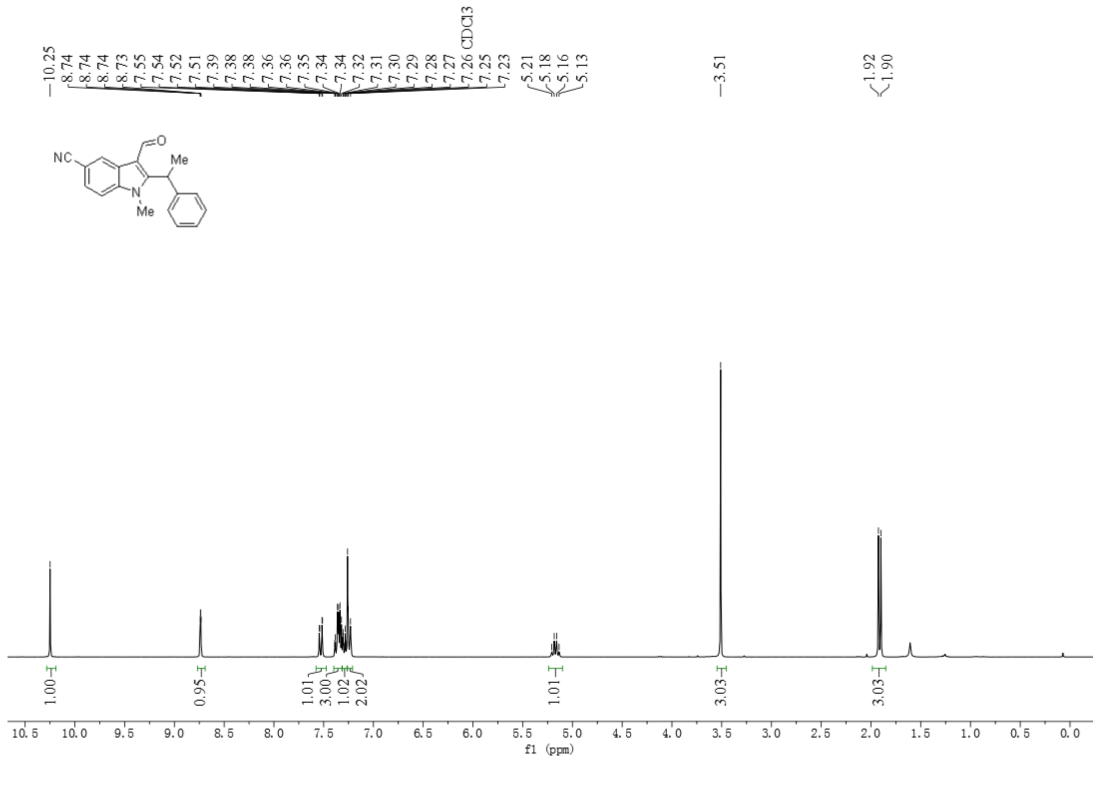

^{13}C-NMR spectrum of **3g**

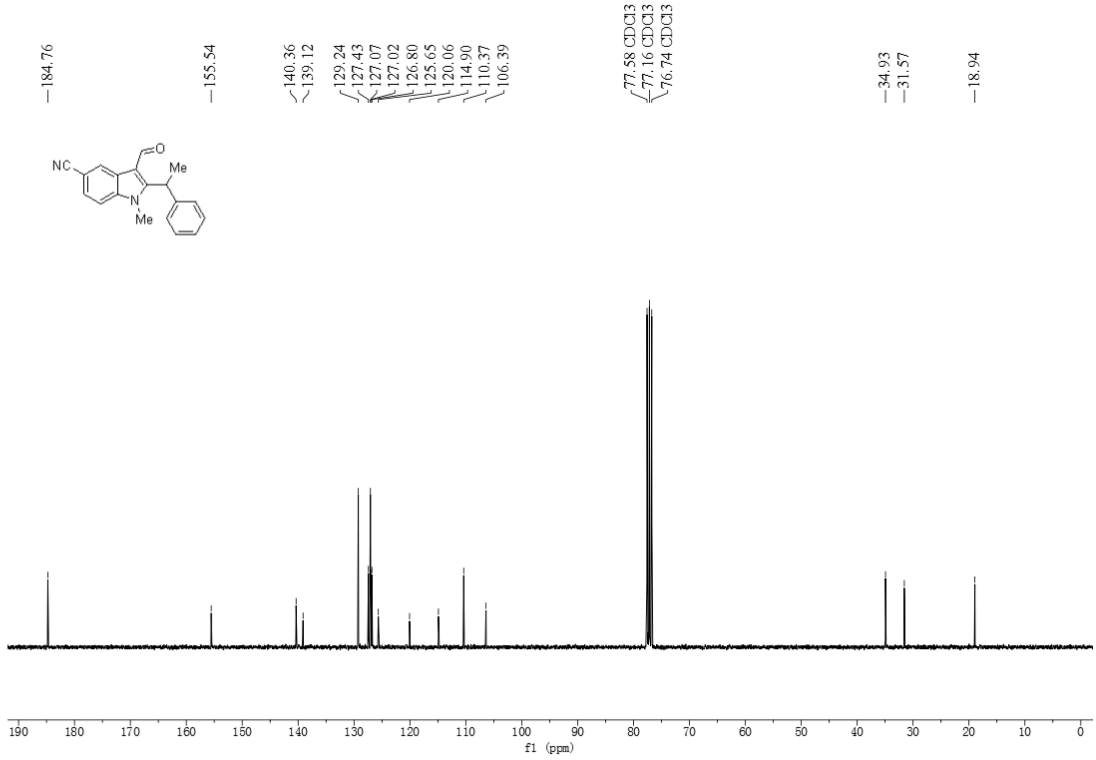

^{1}H-NMR spectrum of **3h**

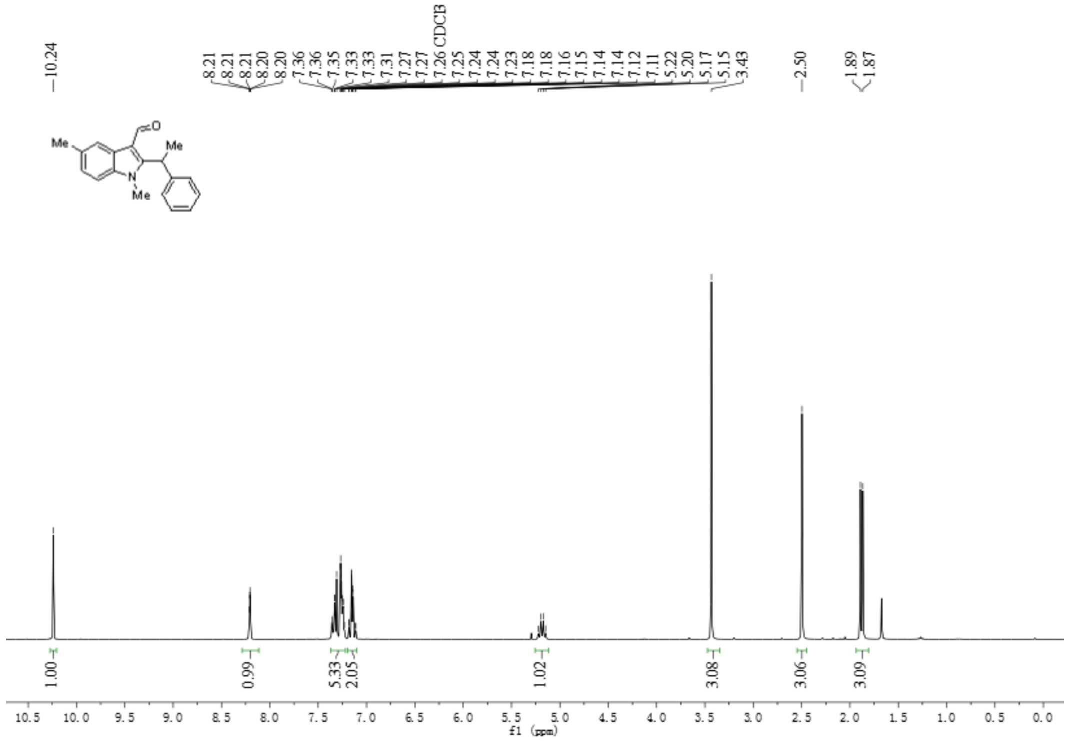

^{13}C-NMR spectrum of **3h**

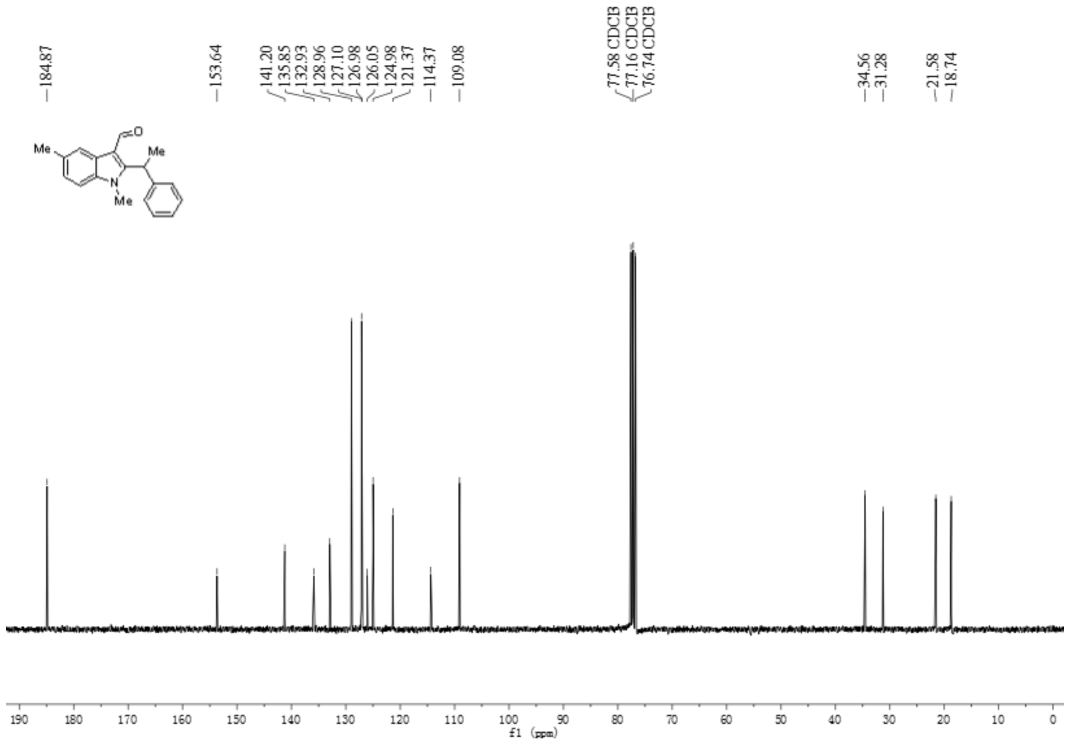

^{1}H-NMR spectrum of **3i**

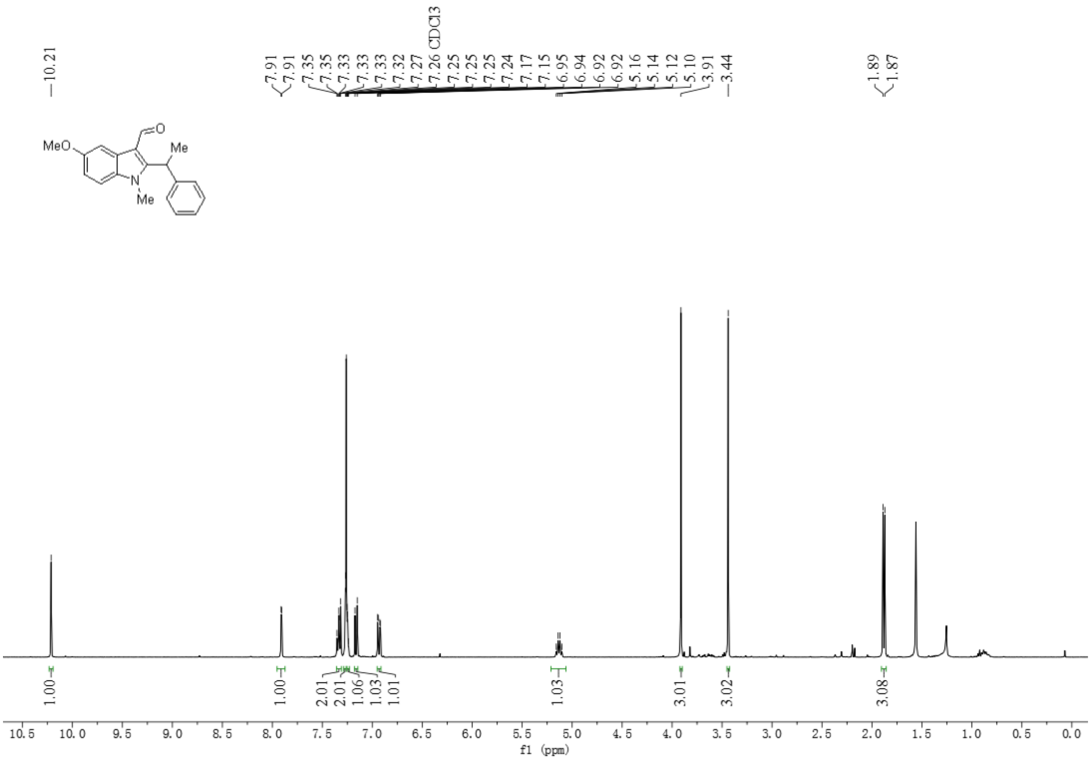

^{13}C-NMR spectrum of **3i**

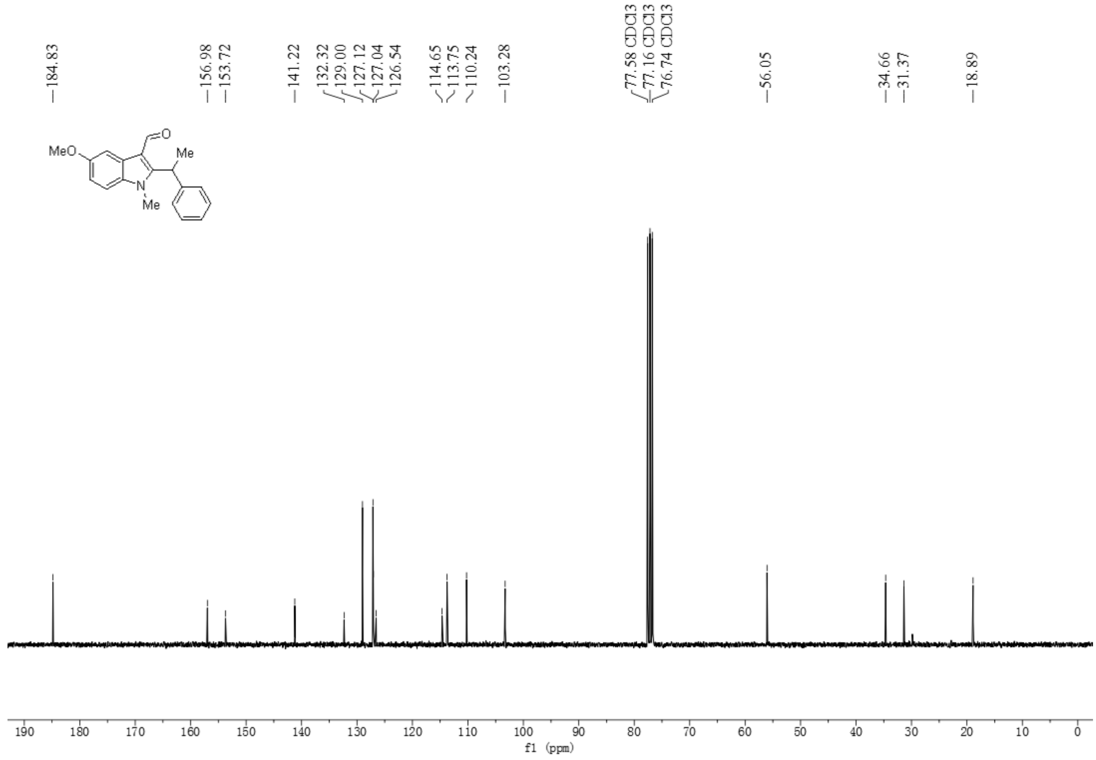

^{1}H-NMR spectrum of **3j**

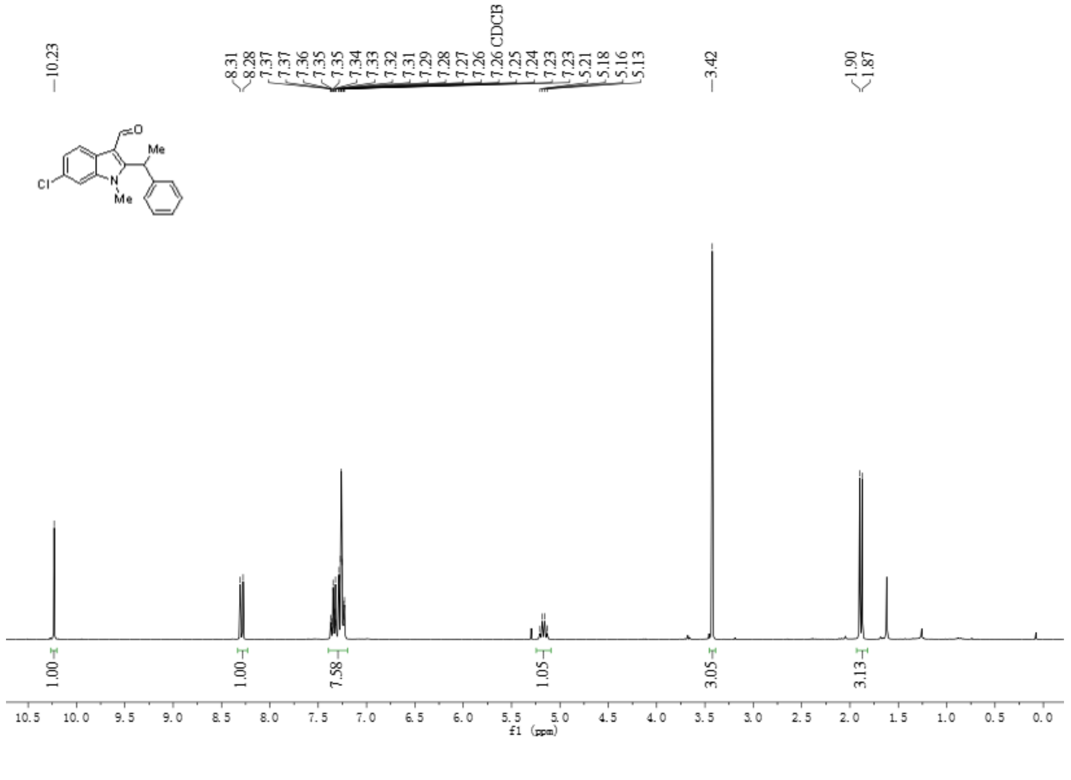

^{13}C-NMR spectrum of **3j**

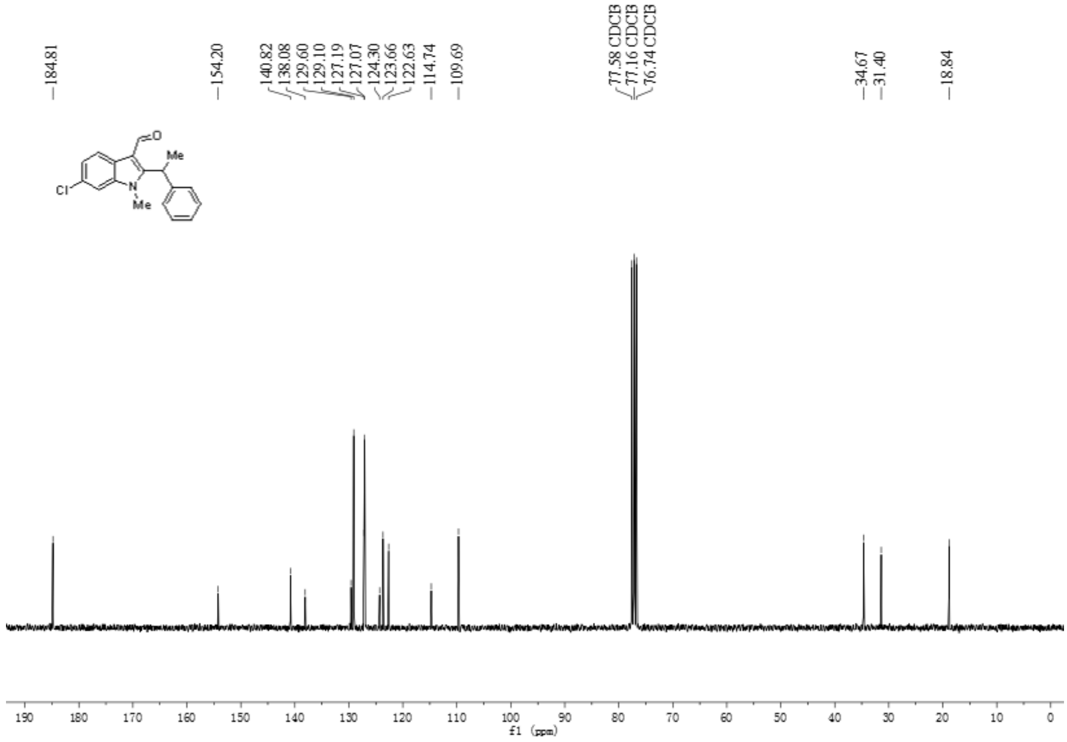

^{1}H-NMR spectrum of **3k**

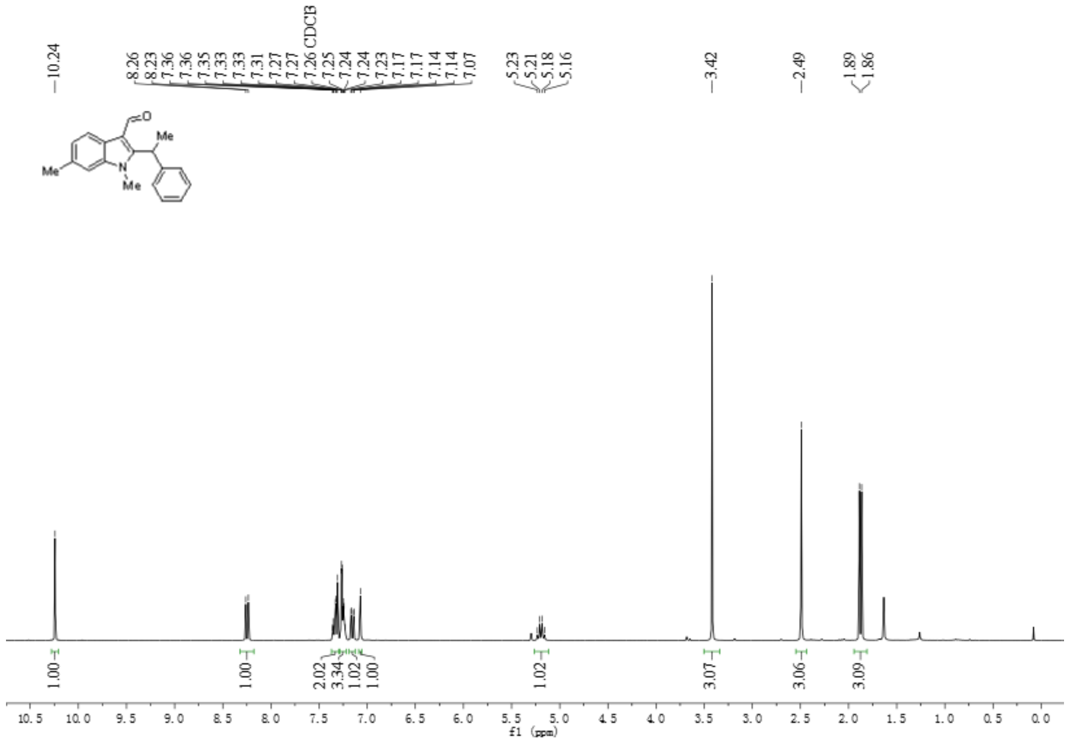

^{13}C-NMR spectrum of **3k**

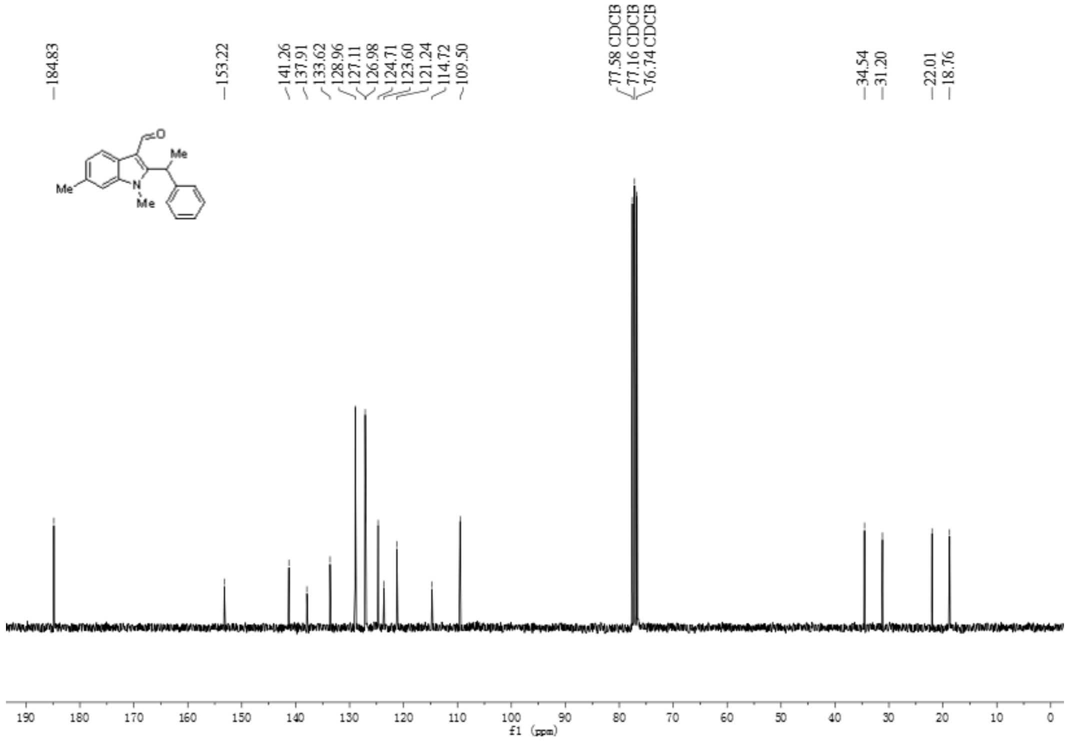

^{1}H-NMR spectrum of **3l**

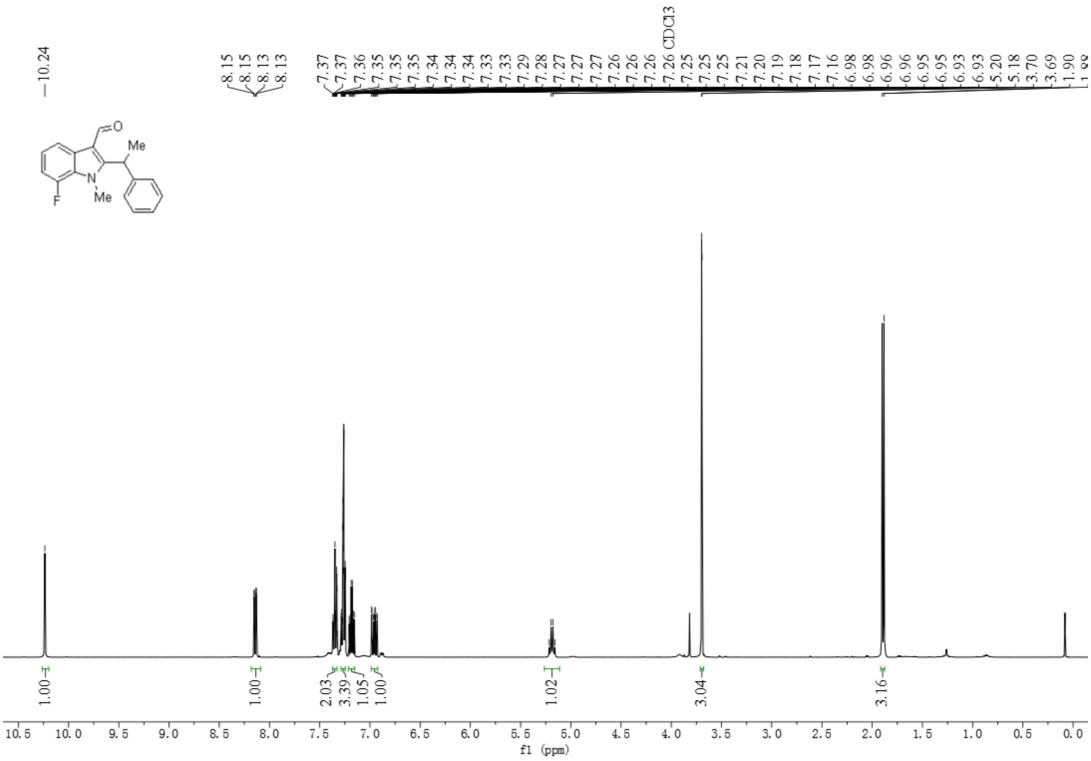

^{13}C-NMR spectrum of **3l**

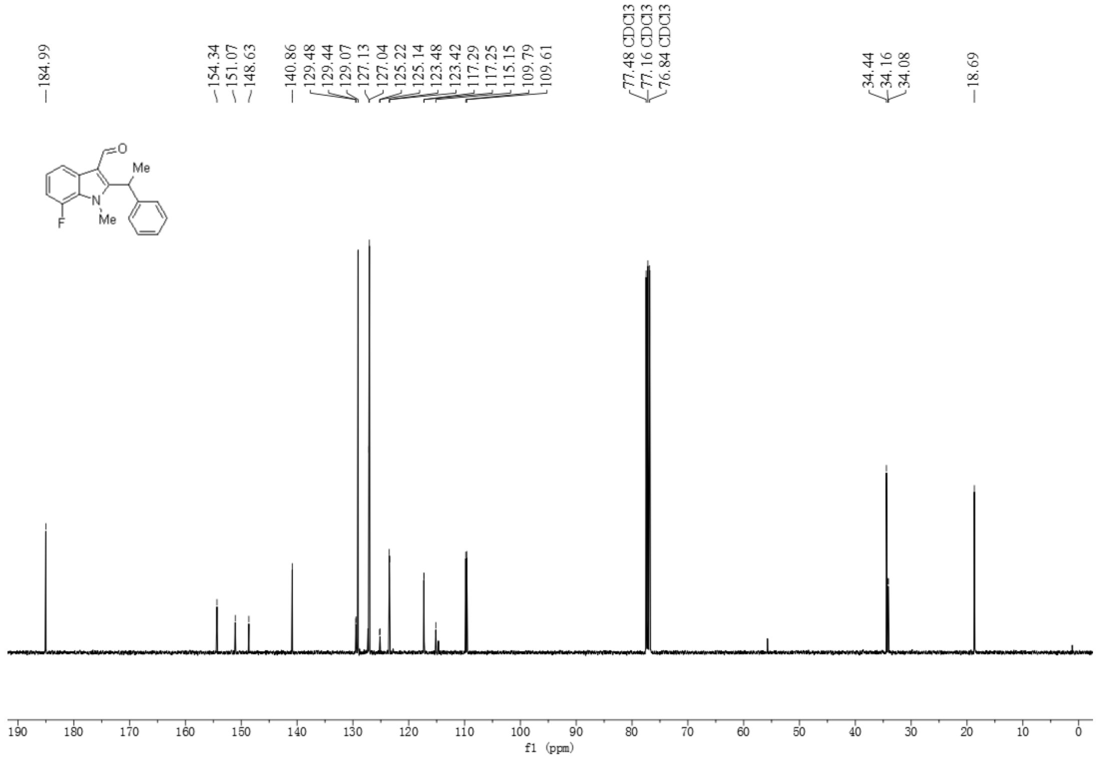

^{19}F-NMR spectrum of **3l**

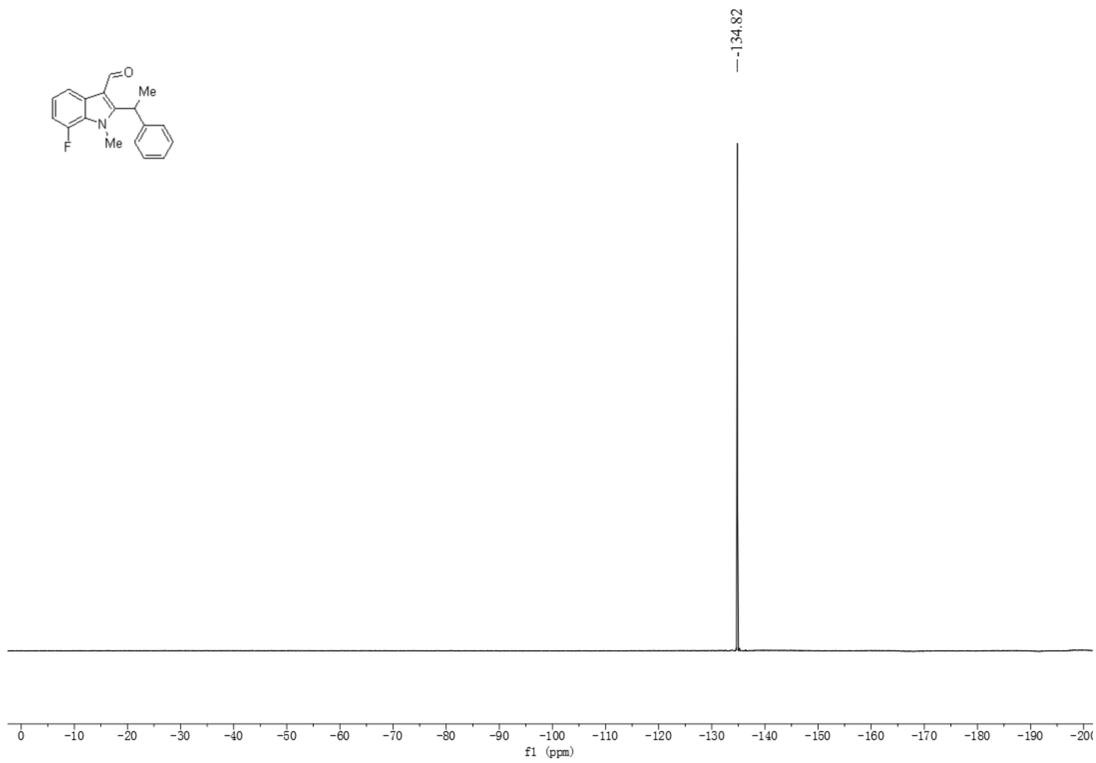

^{1}H-NMR spectrum of **3m**

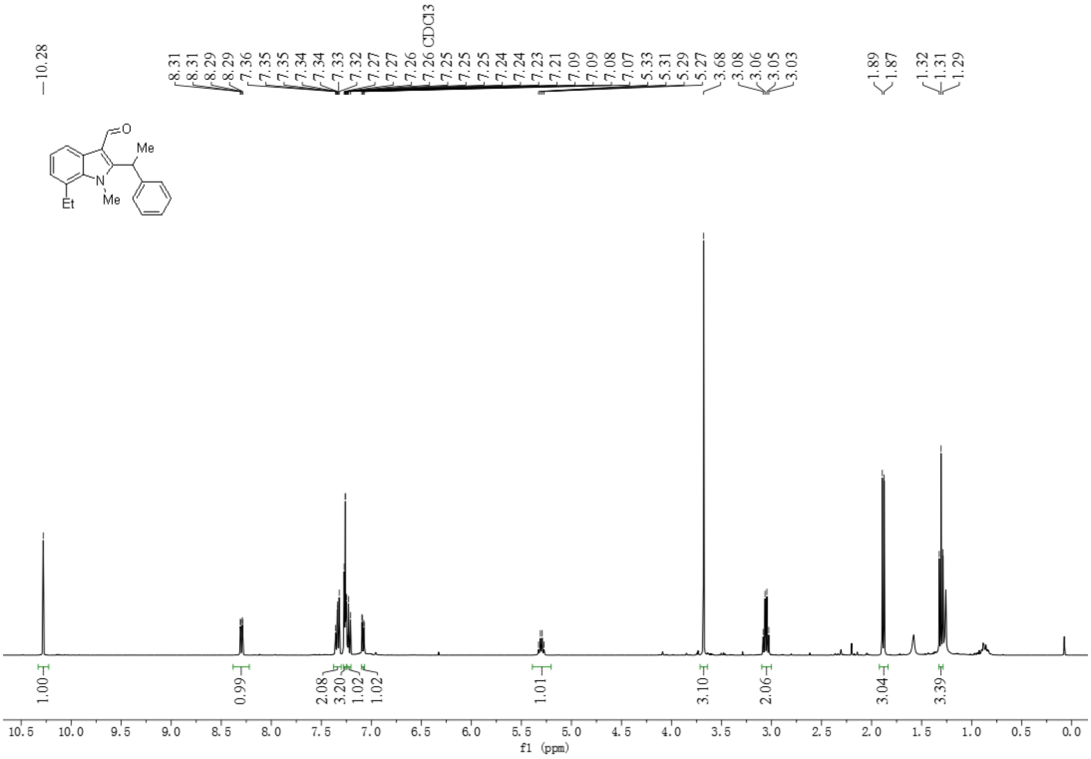

^{13}C-NMR spectrum of **3m**

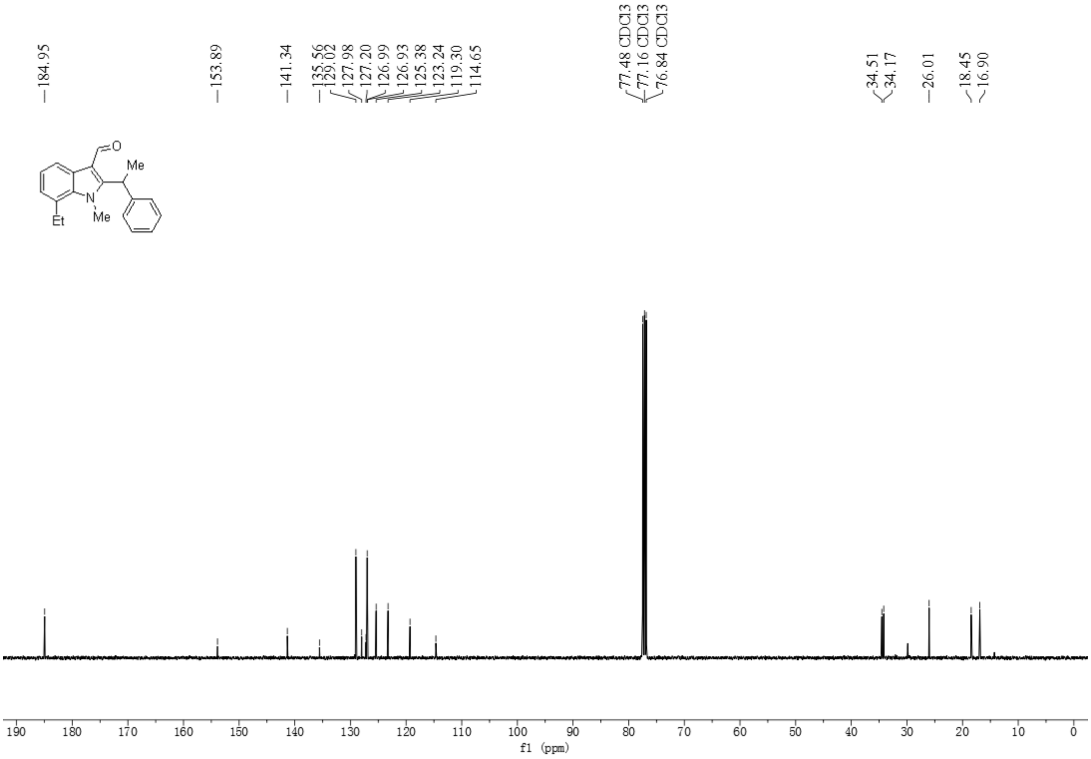

^{1}H-NMR spectrum of **3n**

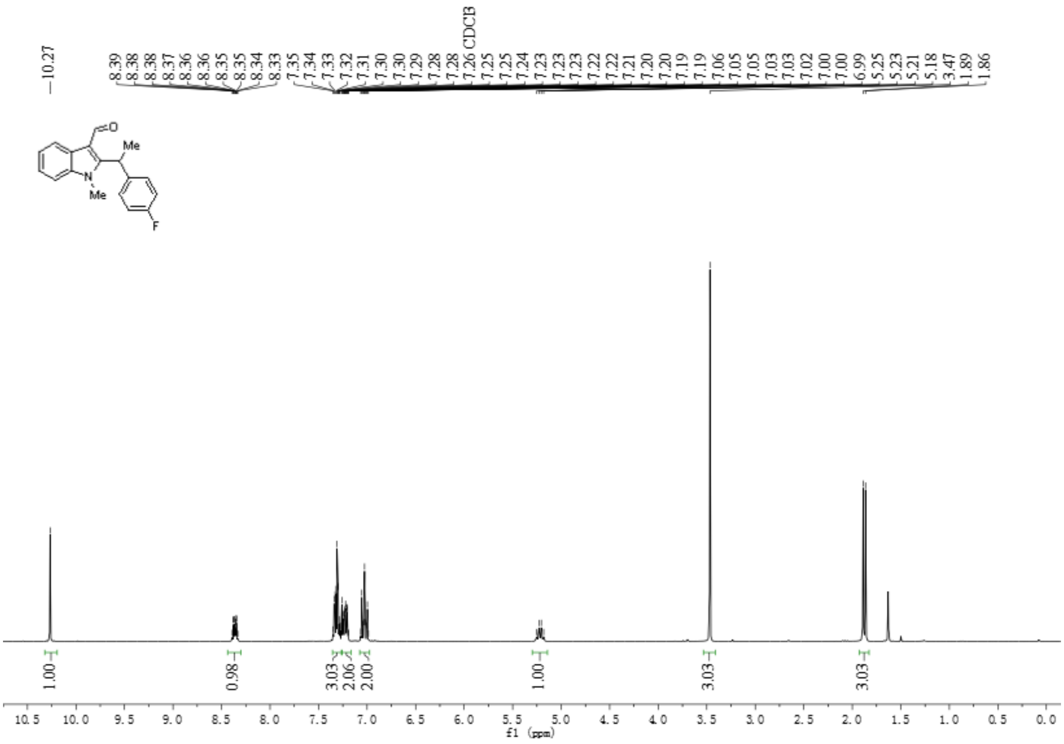

^{13}C-NMR spectrum of **3n**

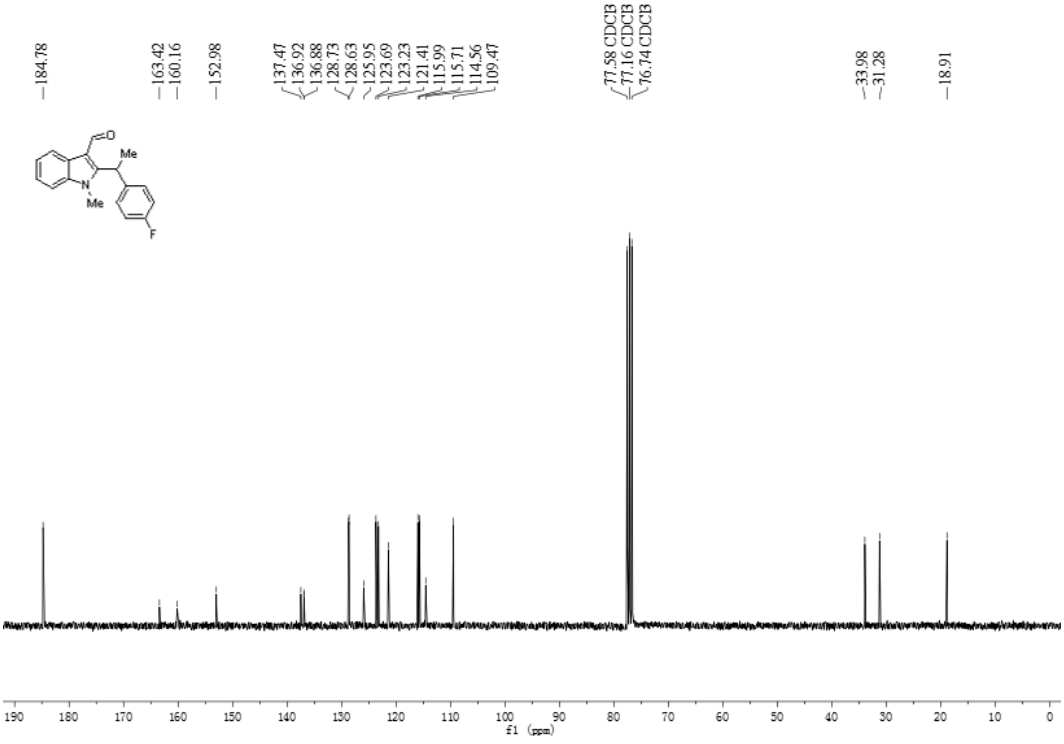

^{19}F-NMR spectrum of **3n**

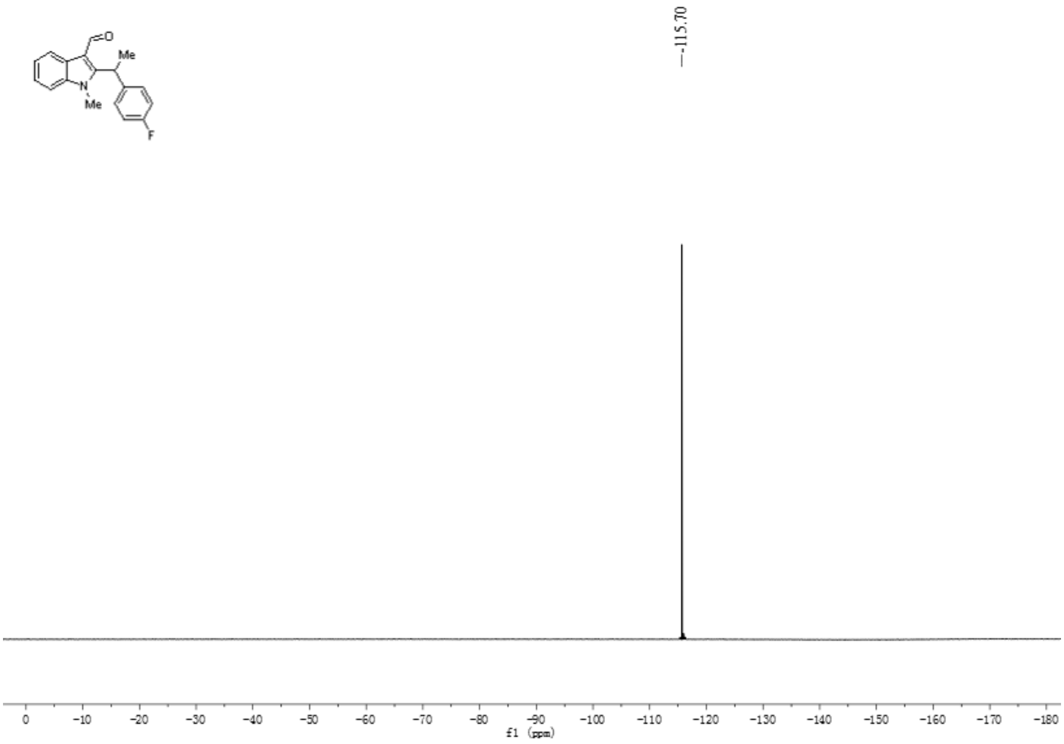

^{1}H-NMR spectrum of **3o**

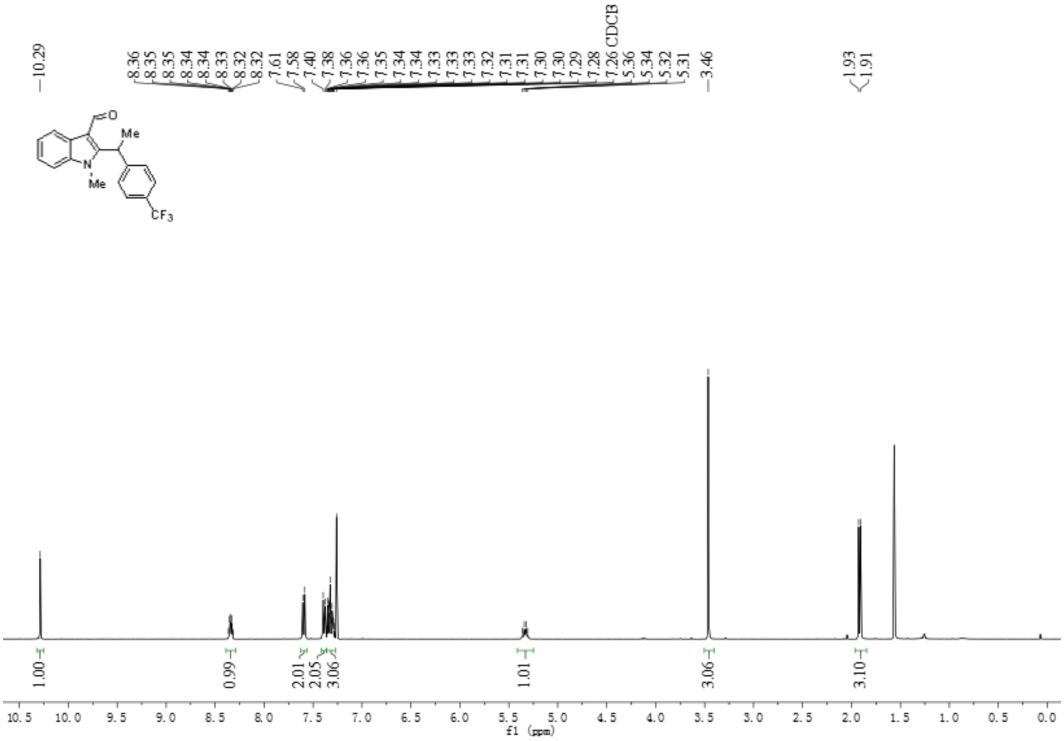

^{13}C-NMR spectrum of **3o**

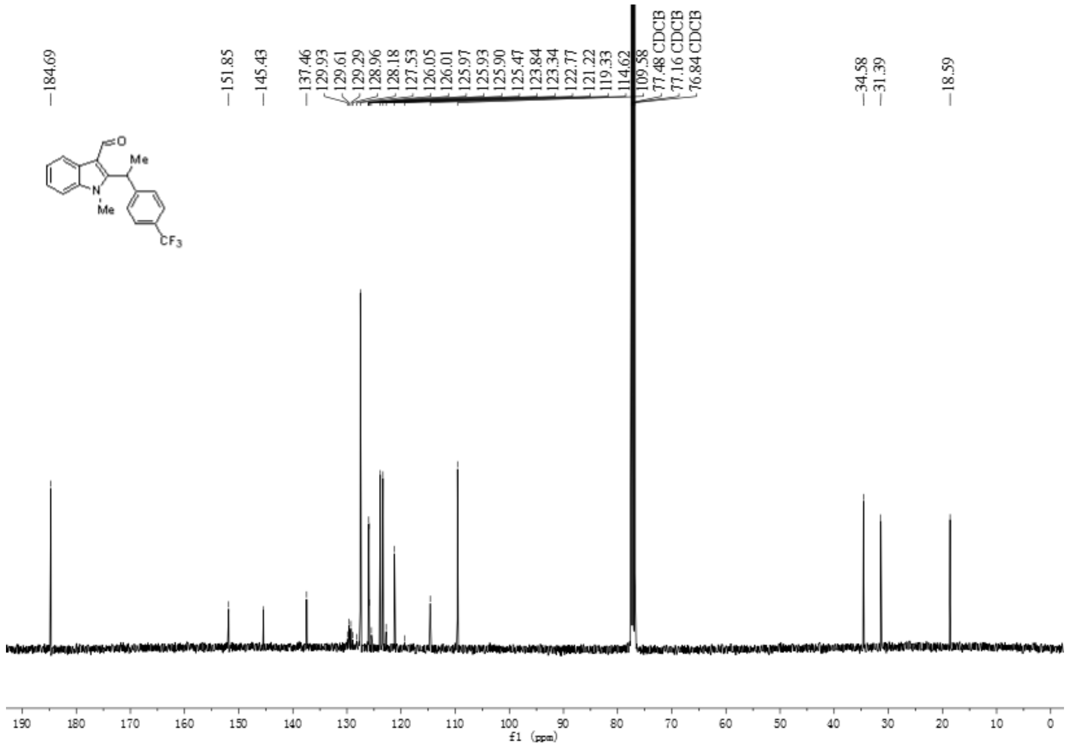

^{19}F-NMR spectrum of **3o**

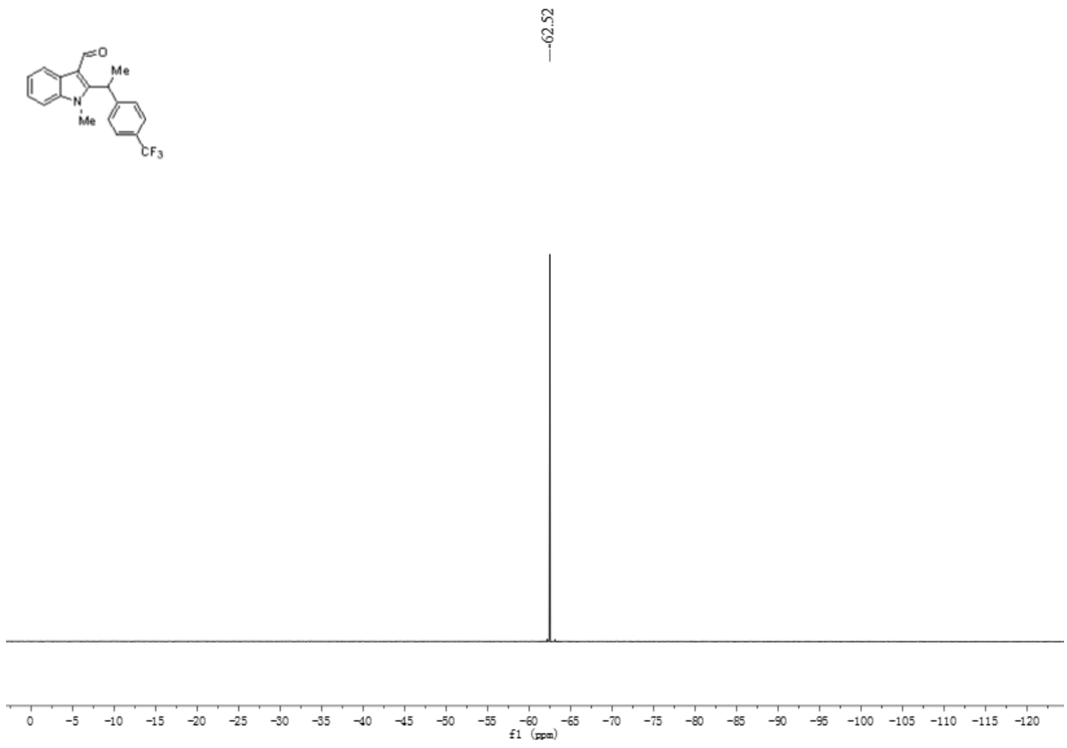

^{1}H-NMR spectrum of **3p**

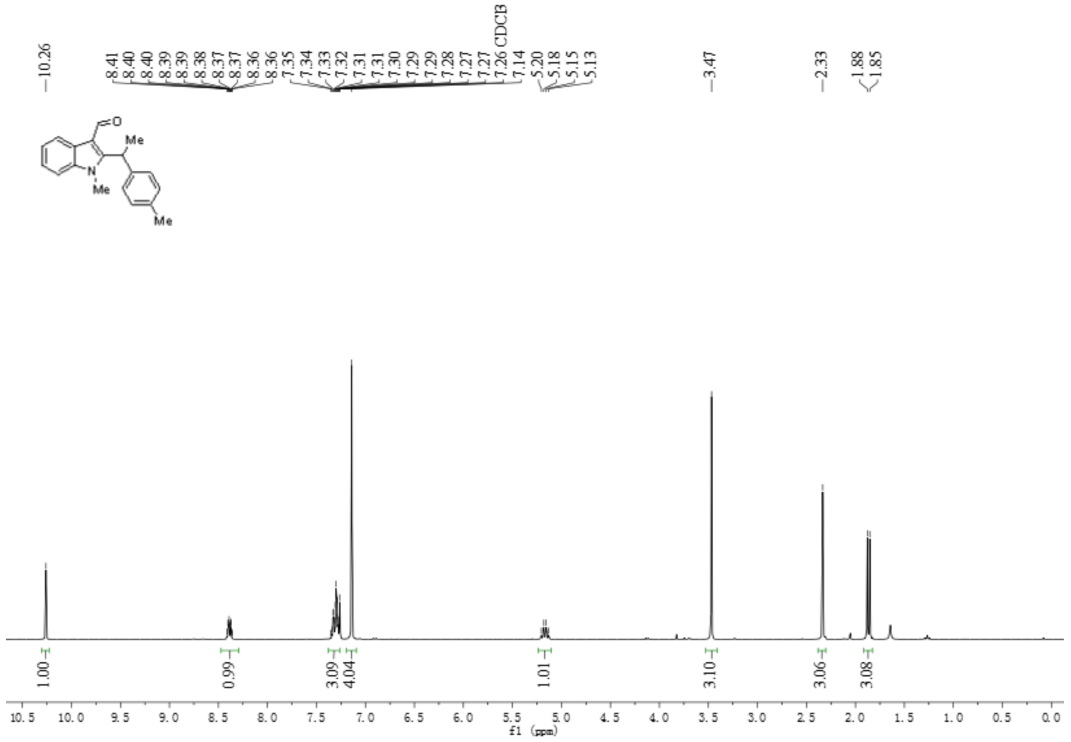

^{13}C-NMR spectrum of **3p**

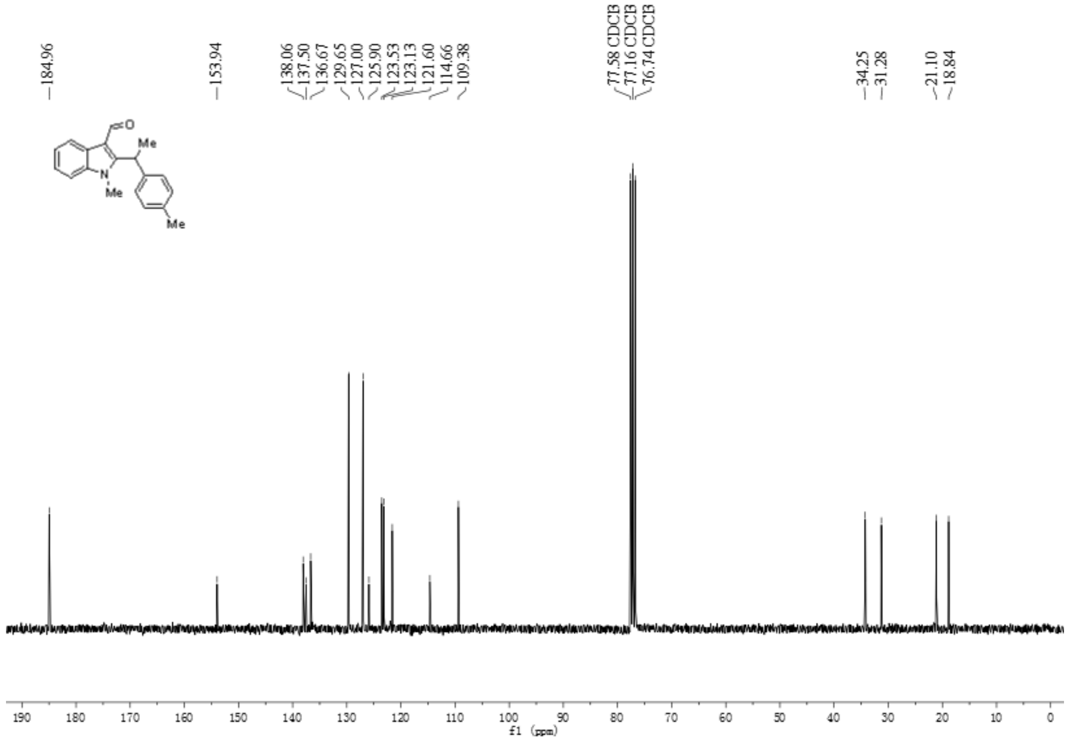

^{1}H-NMR spectrum of **3q**

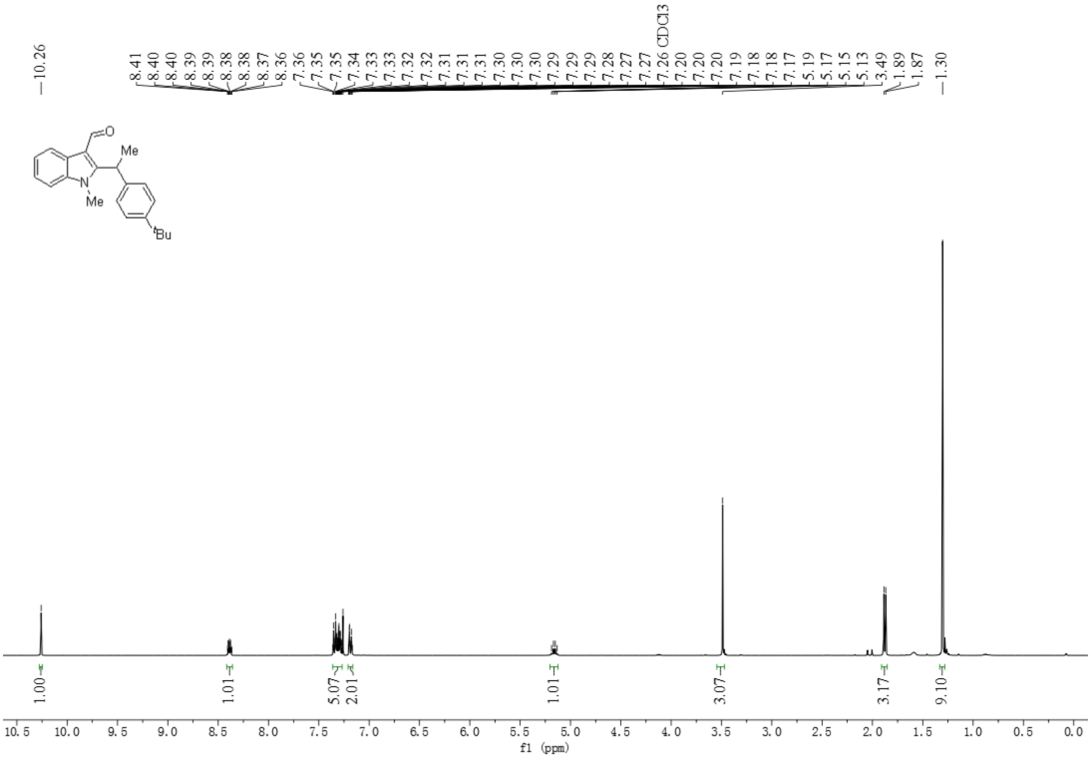

^{13}C-NMR spectrum of **3q**

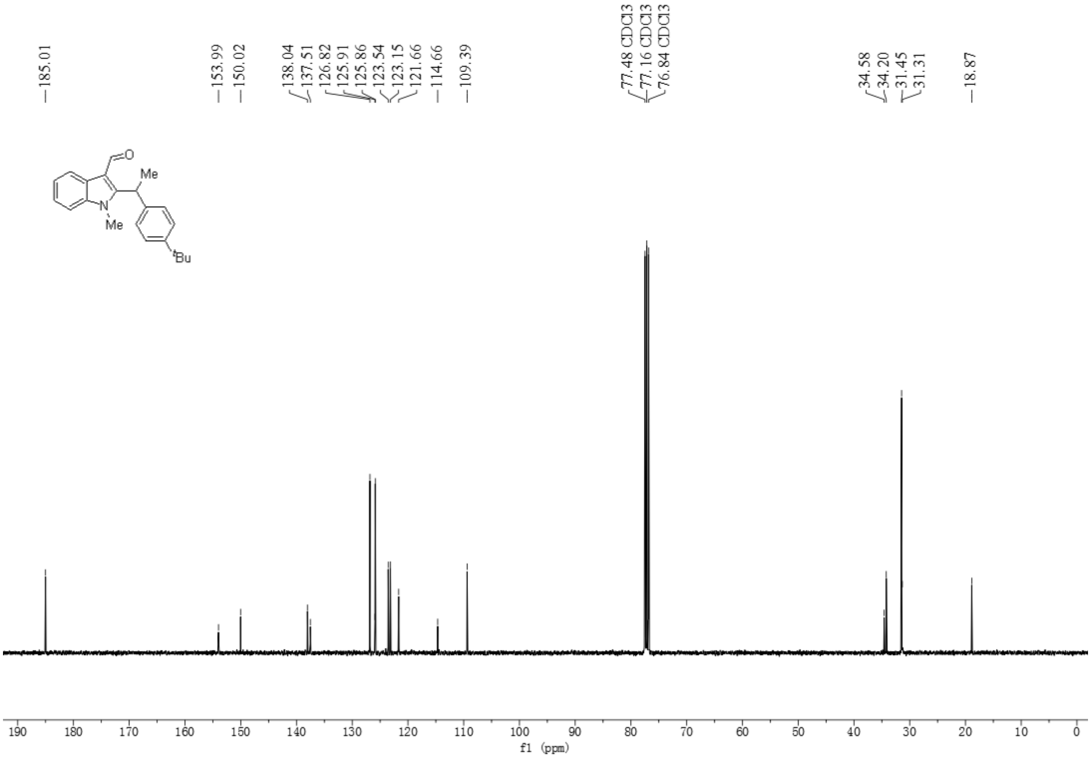

^{1}H-NMR spectrum of **3r**

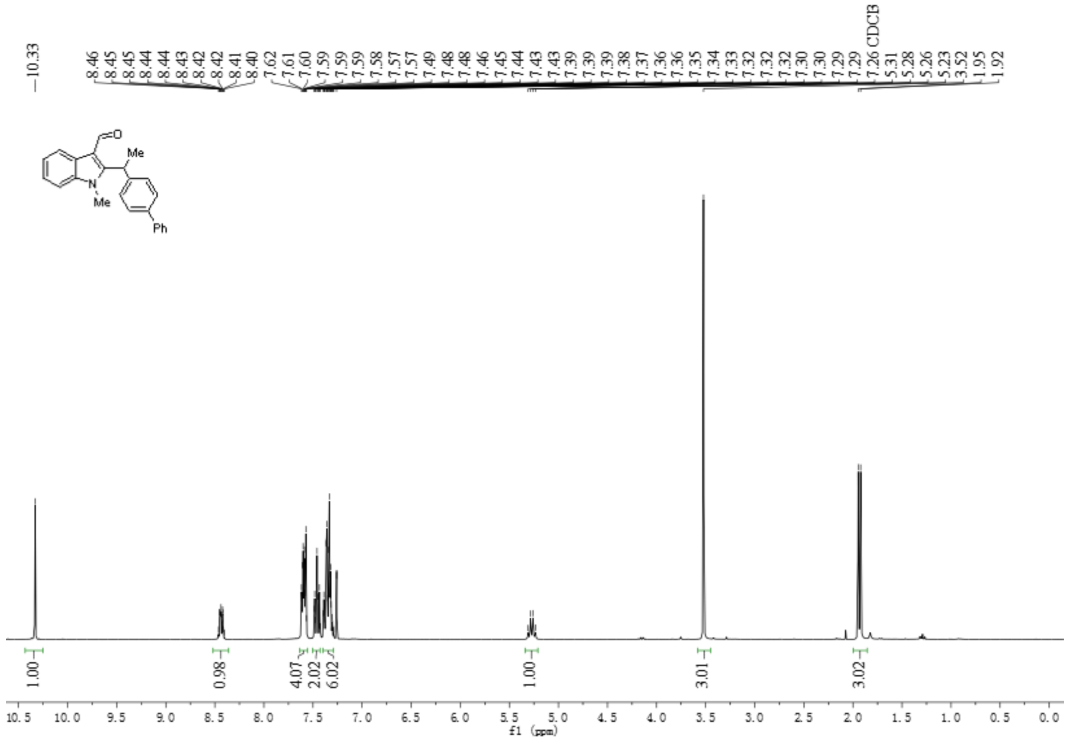

^{13}C-NMR spectrum of **3r**

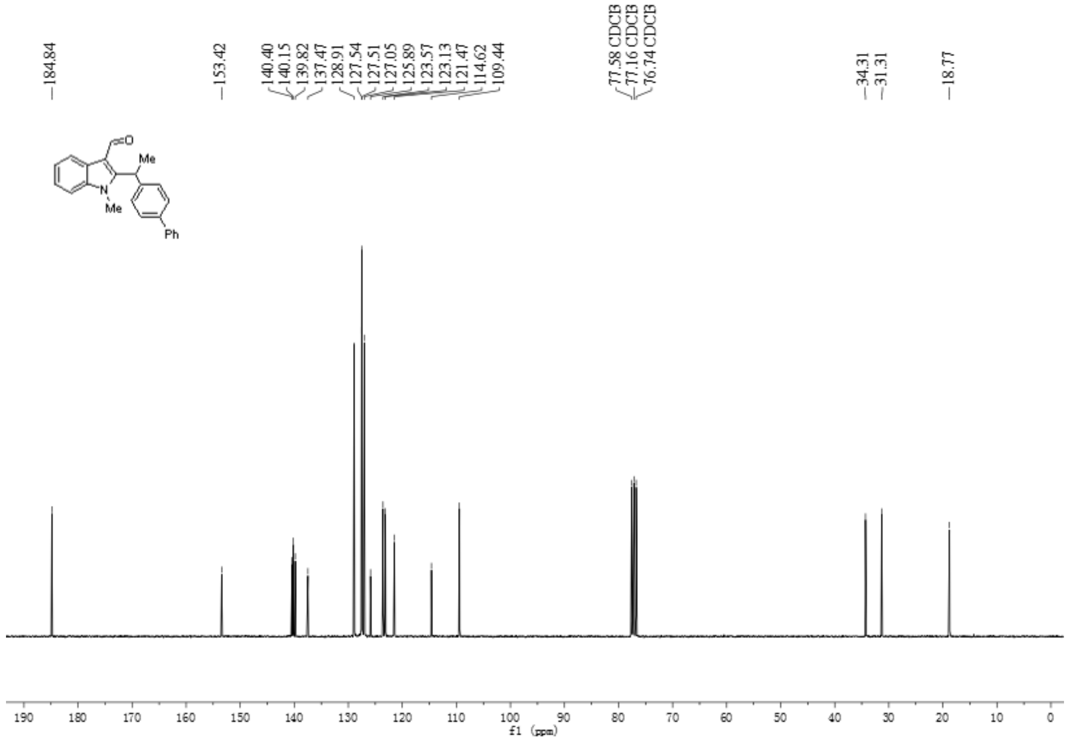

^{1}H-NMR spectrum of **3s**

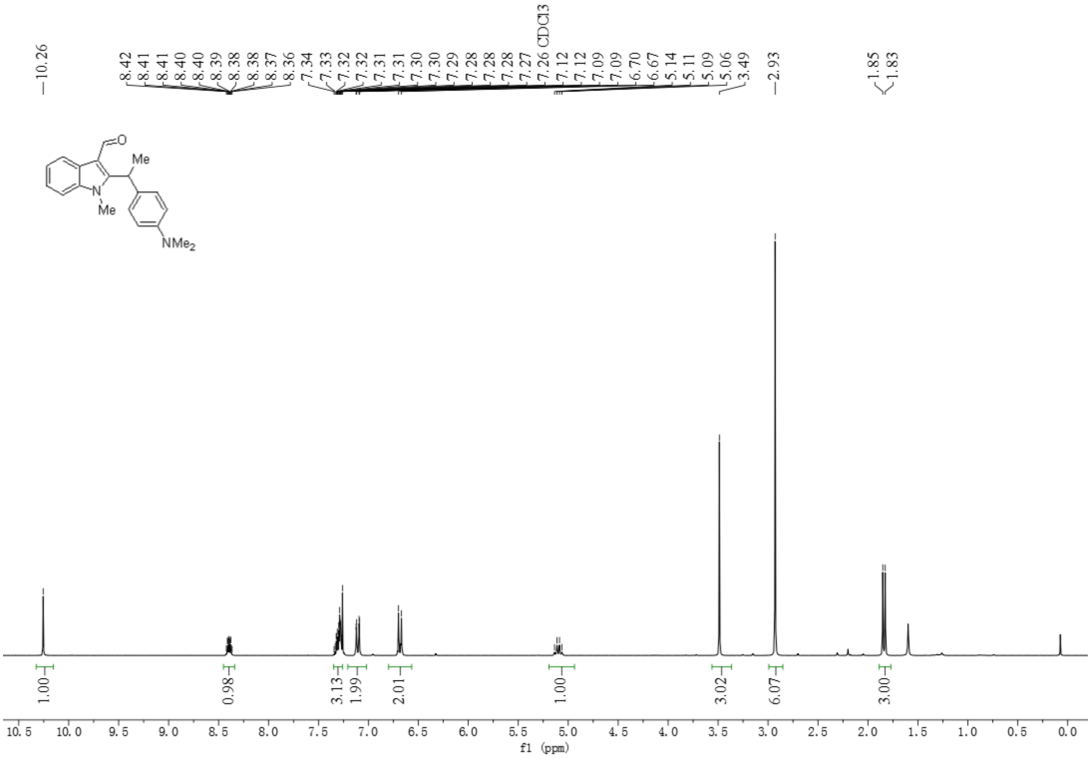

^{13}C-NMR spectrum of **3s**

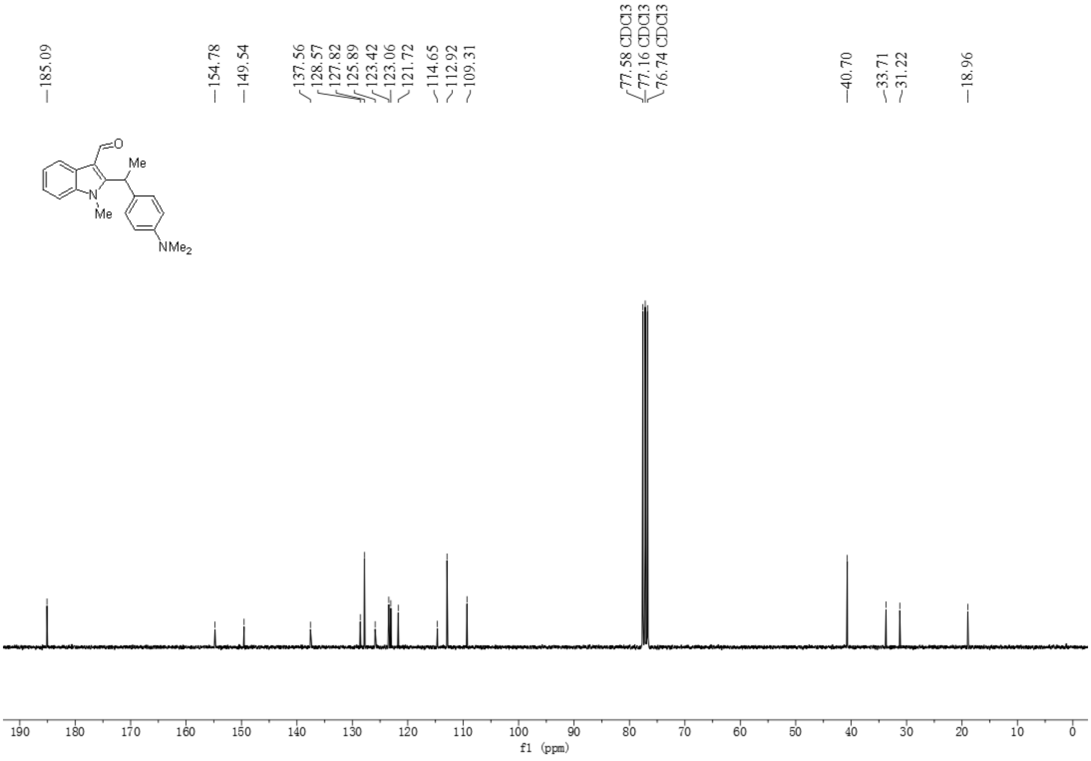

^{1}H-NMR spectrum of **3t**

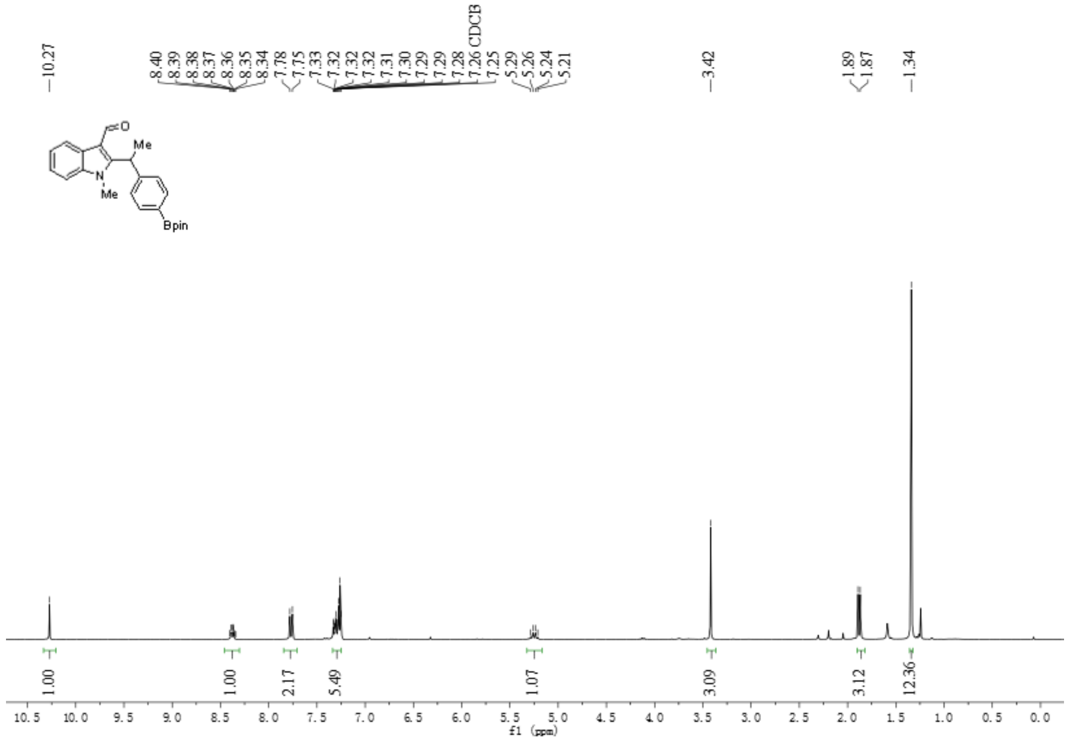

^{13}C-NMR spectrum of **3t**

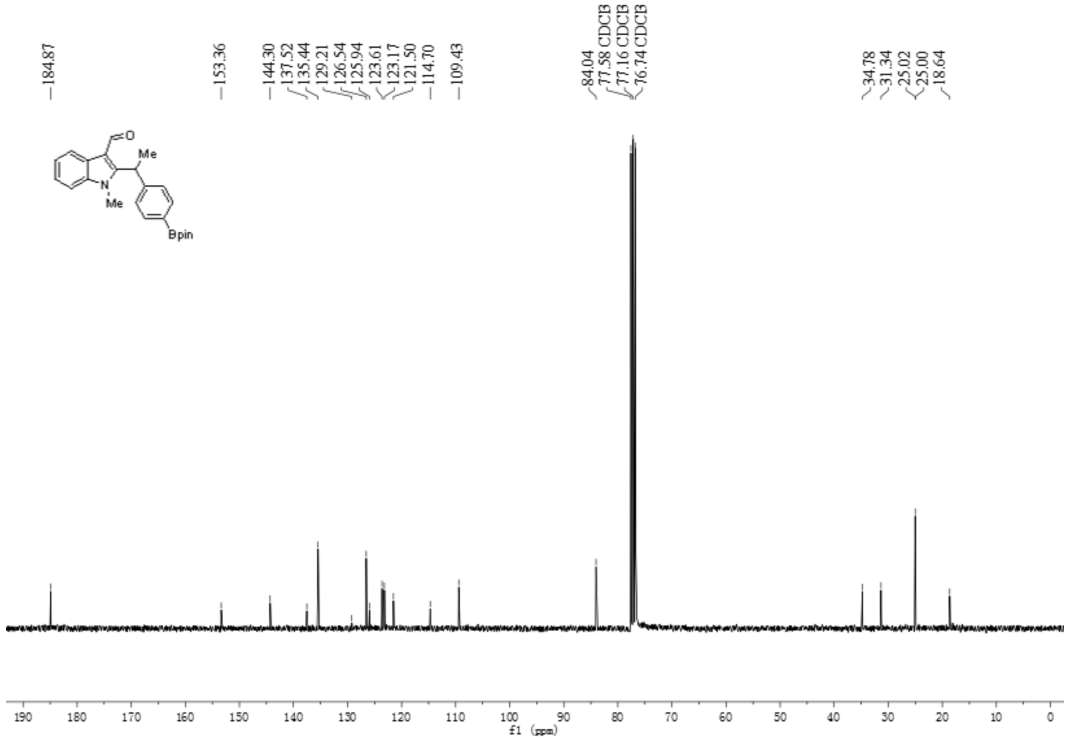

^{1}H-NMR spectrum of **3u**

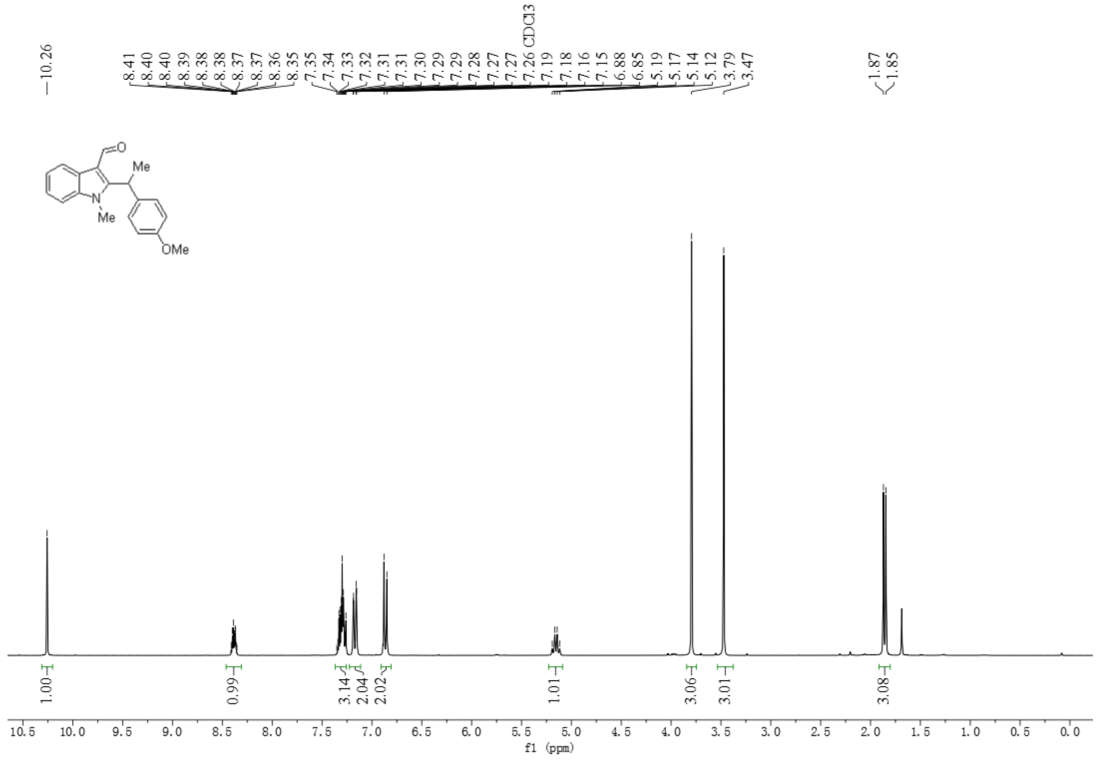

^{13}C-NMR spectrum of **3u**

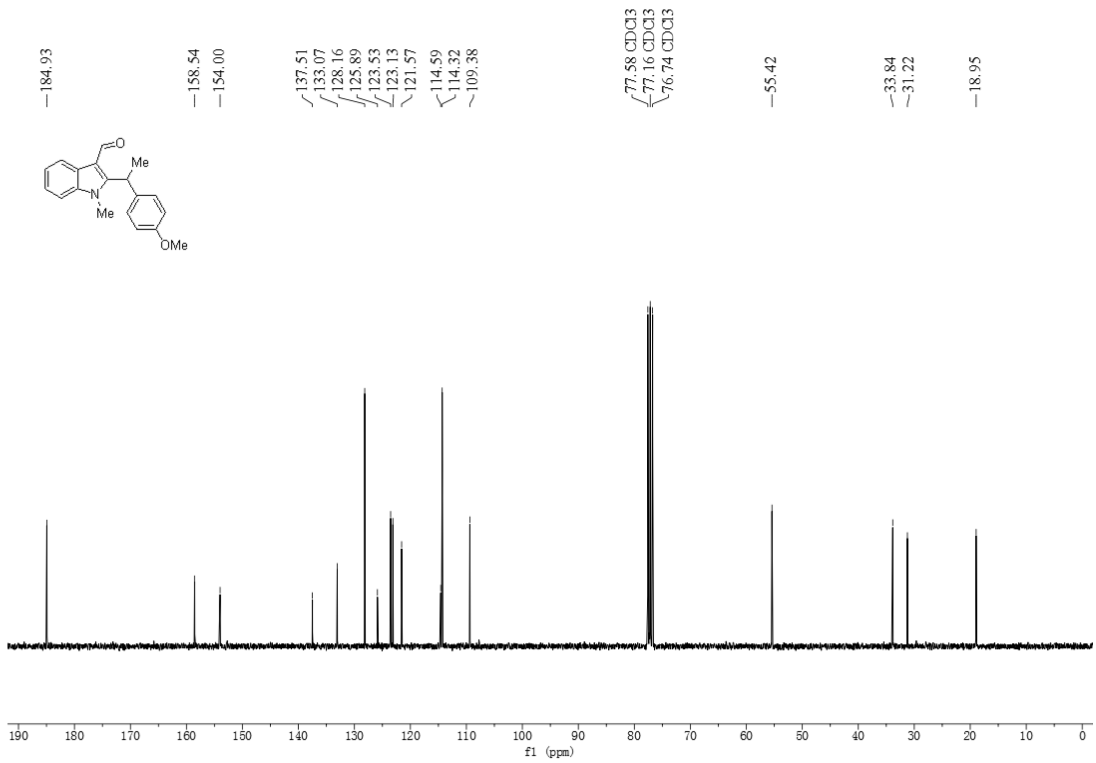

^{1}H-NMR spectrum of **3v**

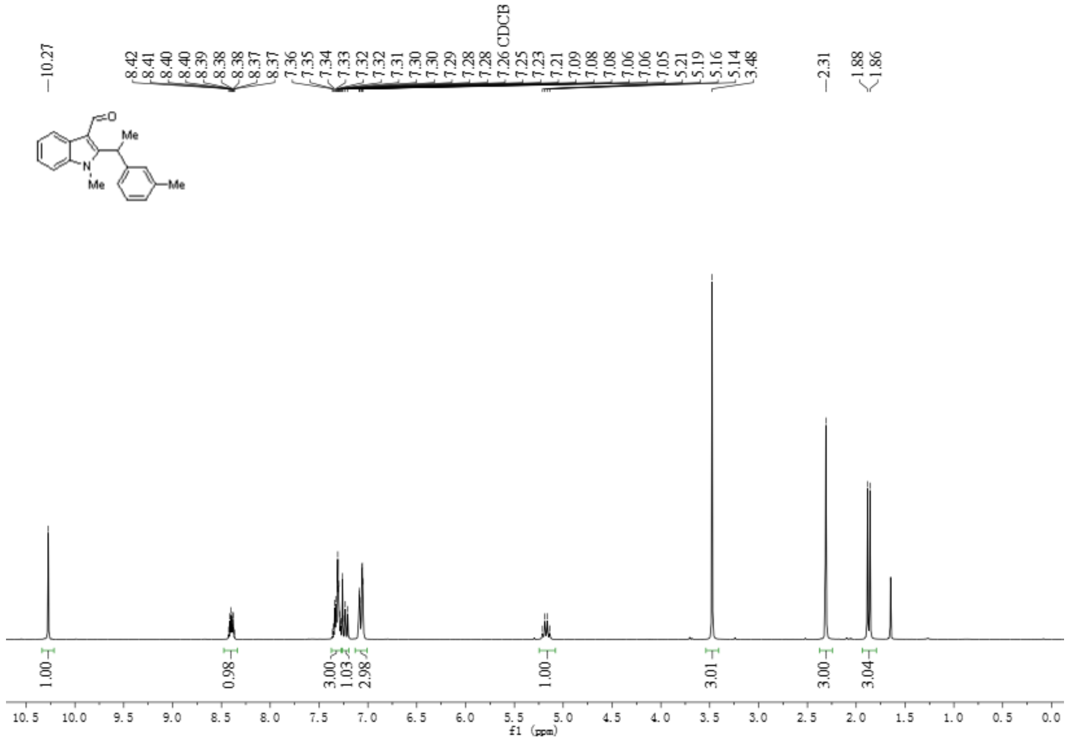

^{13}C-NMR spectrum of **3v**

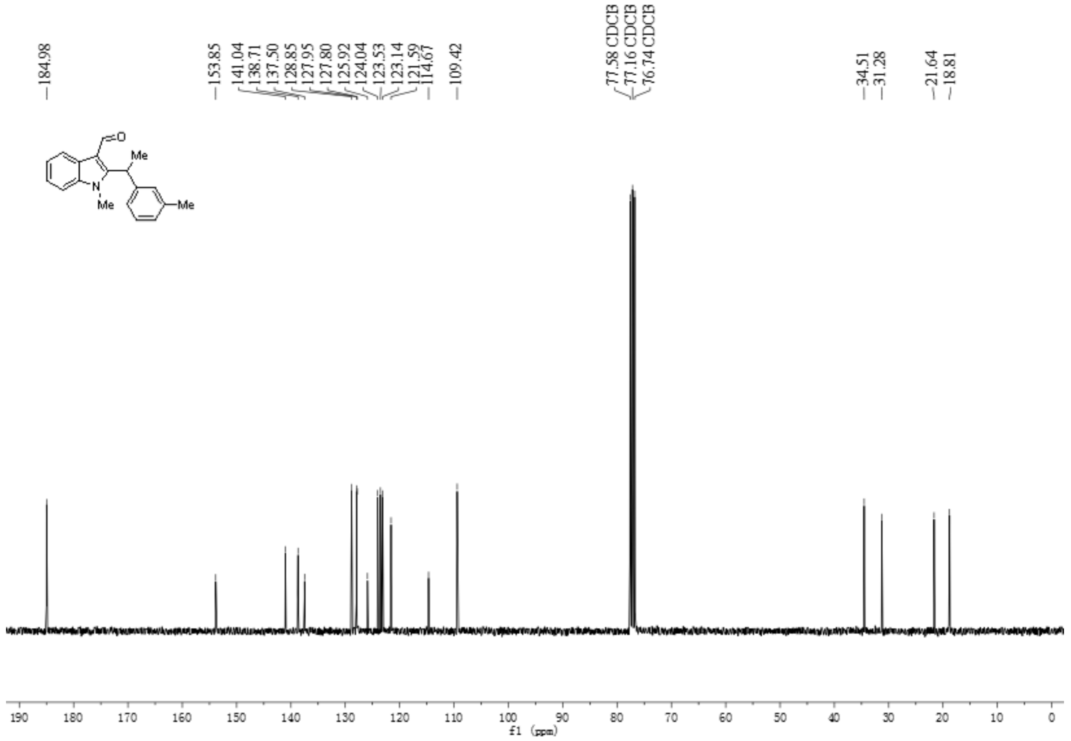

^{1}H-NMR spectrum of **3w**

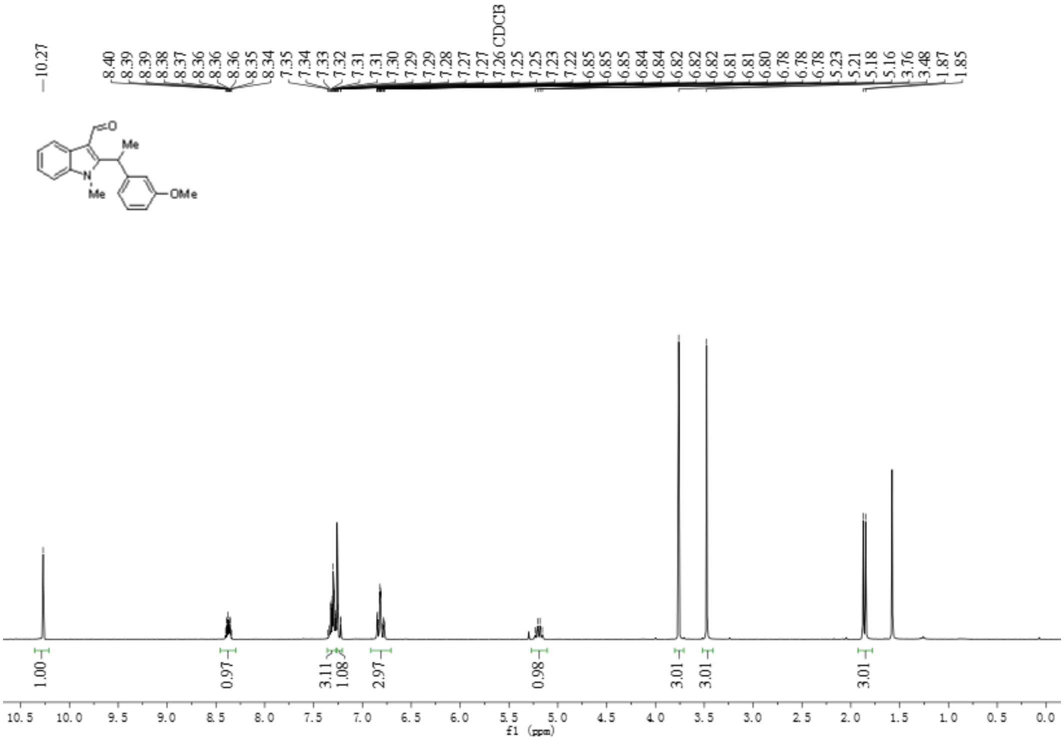

^{13}C-NMR spectrum of **3w**

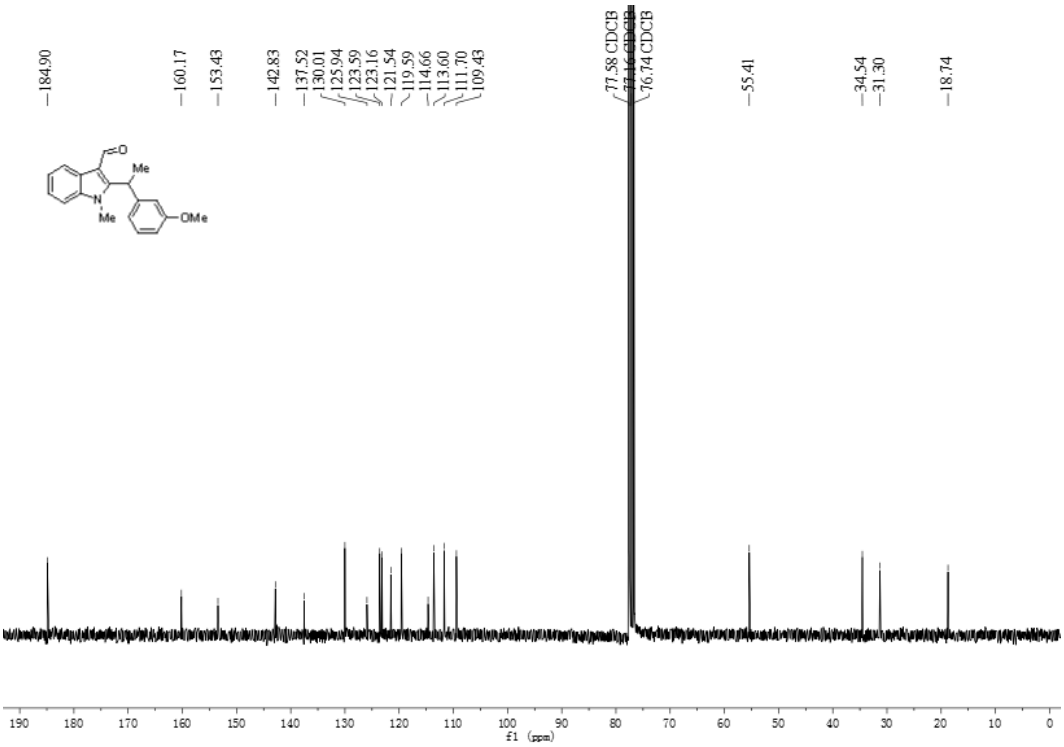

^{1}H-NMR spectrum of **3x**

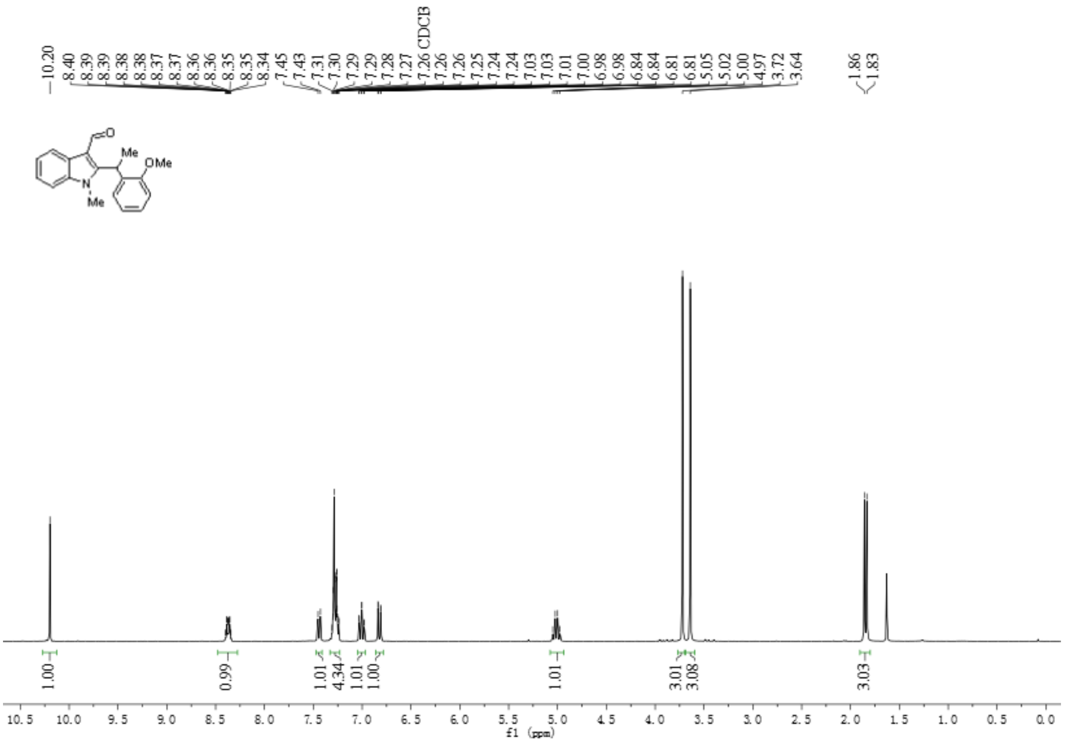

^{13}C-NMR spectrum of **3x**

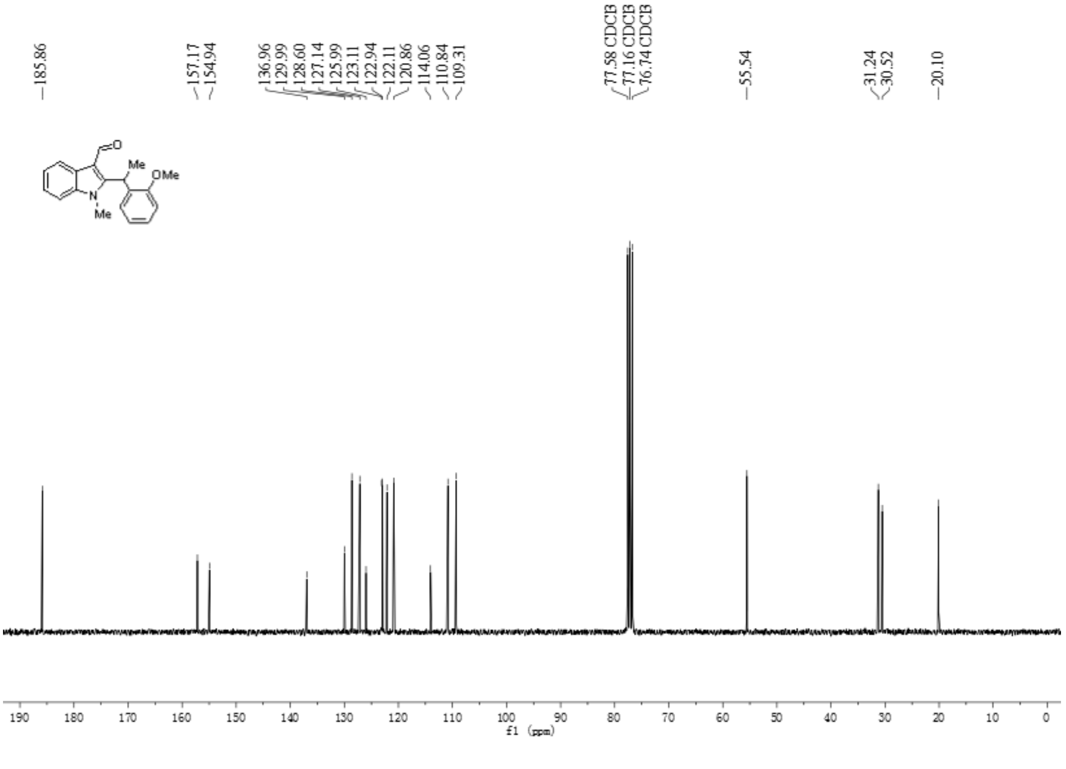

^{1}H-NMR spectrum of **3y**

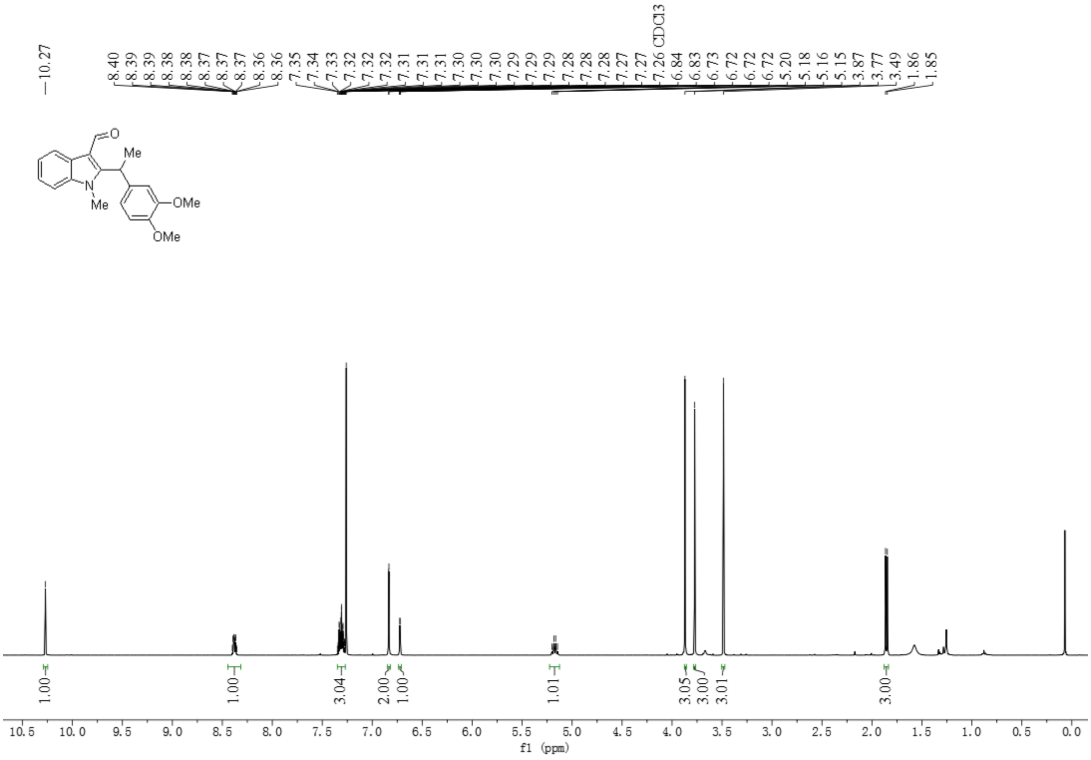

^{13}C-NMR spectrum of **3y**

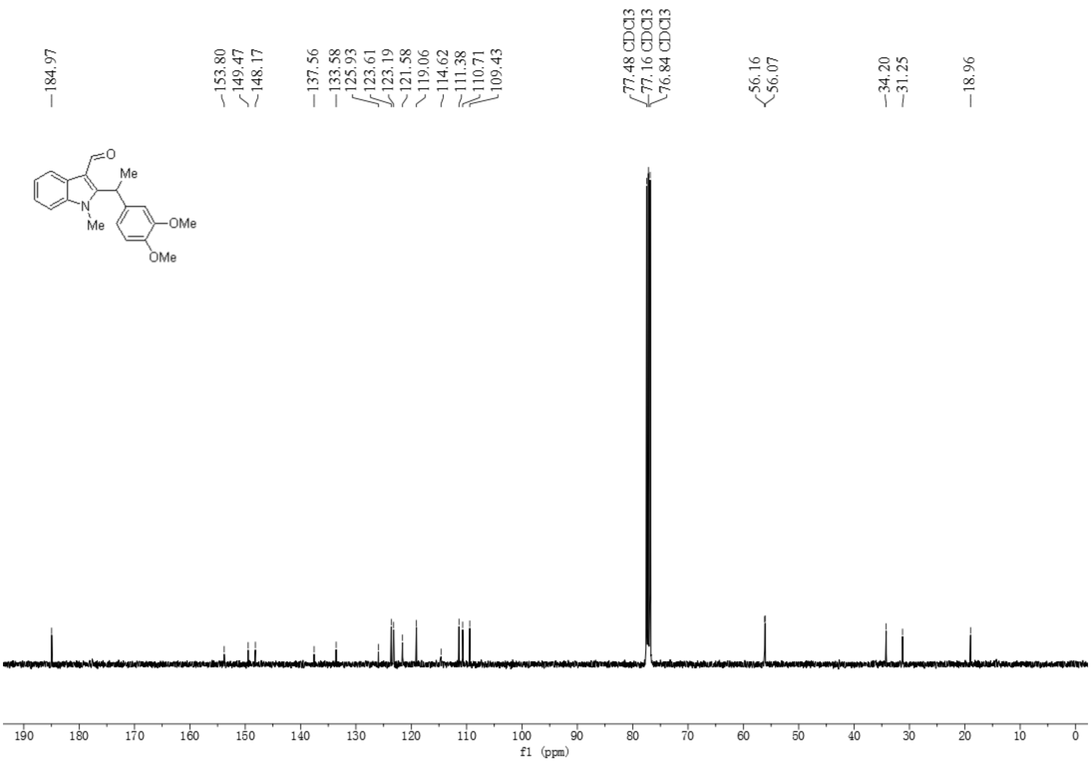

^{1}H-NMR spectrum of **3z**

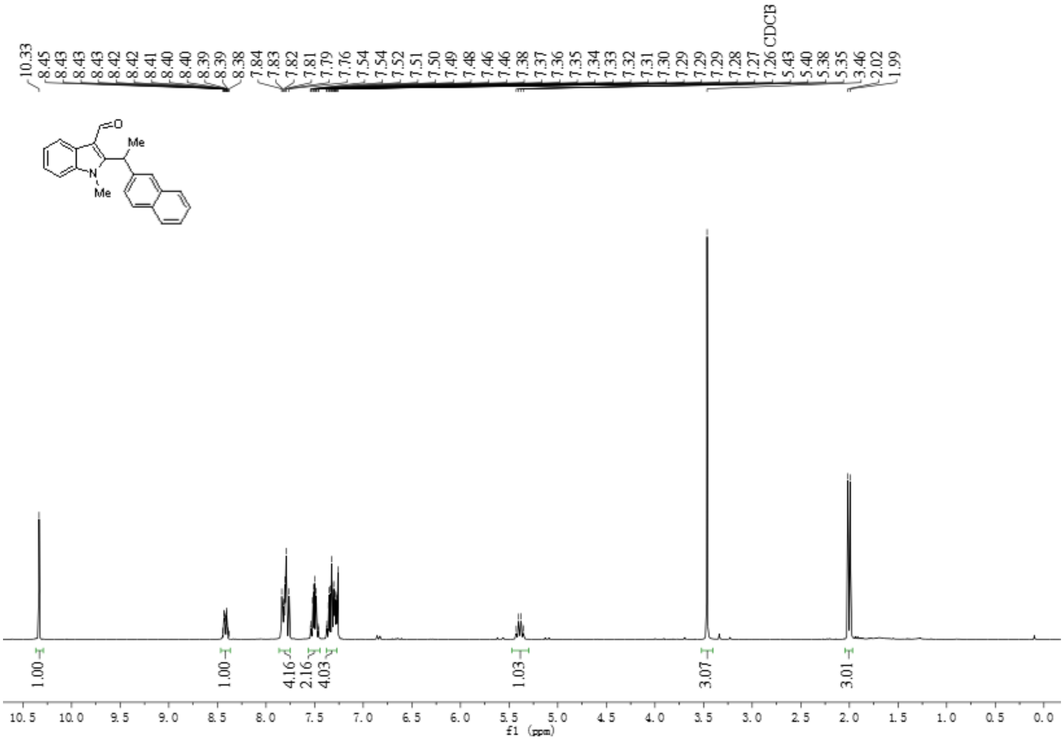

^{13}C-NMR spectrum of **3z**

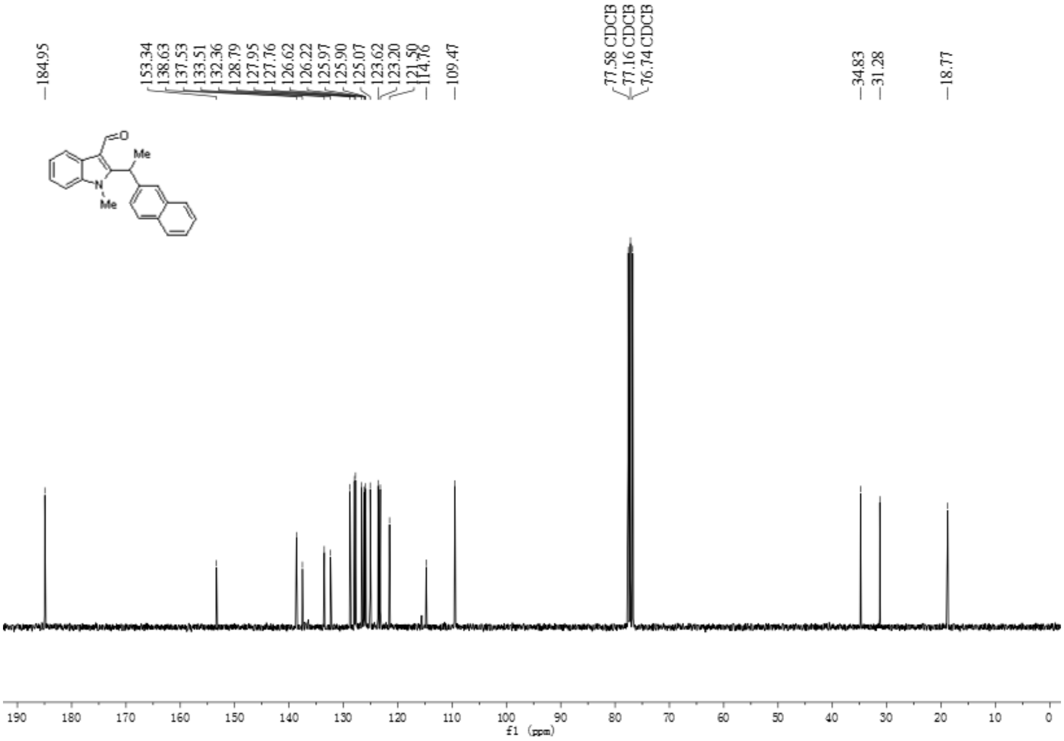

^{1}H-NMR spectrum of **3aa**

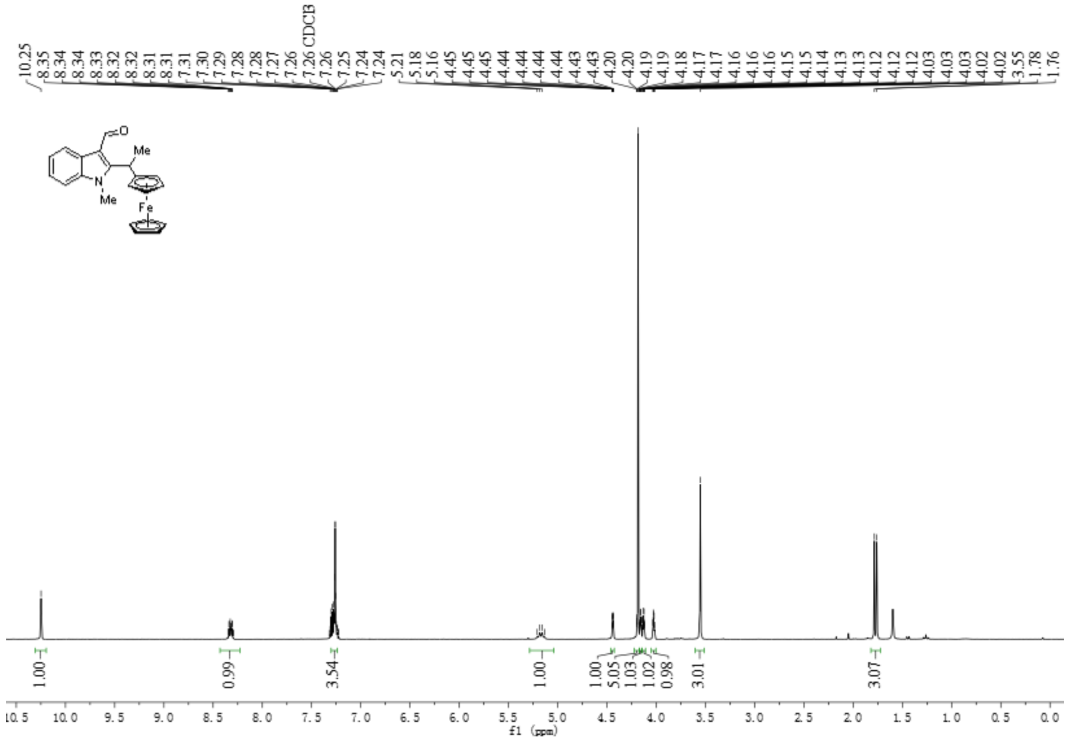

^{13}C-NMR spectrum of **3aa**

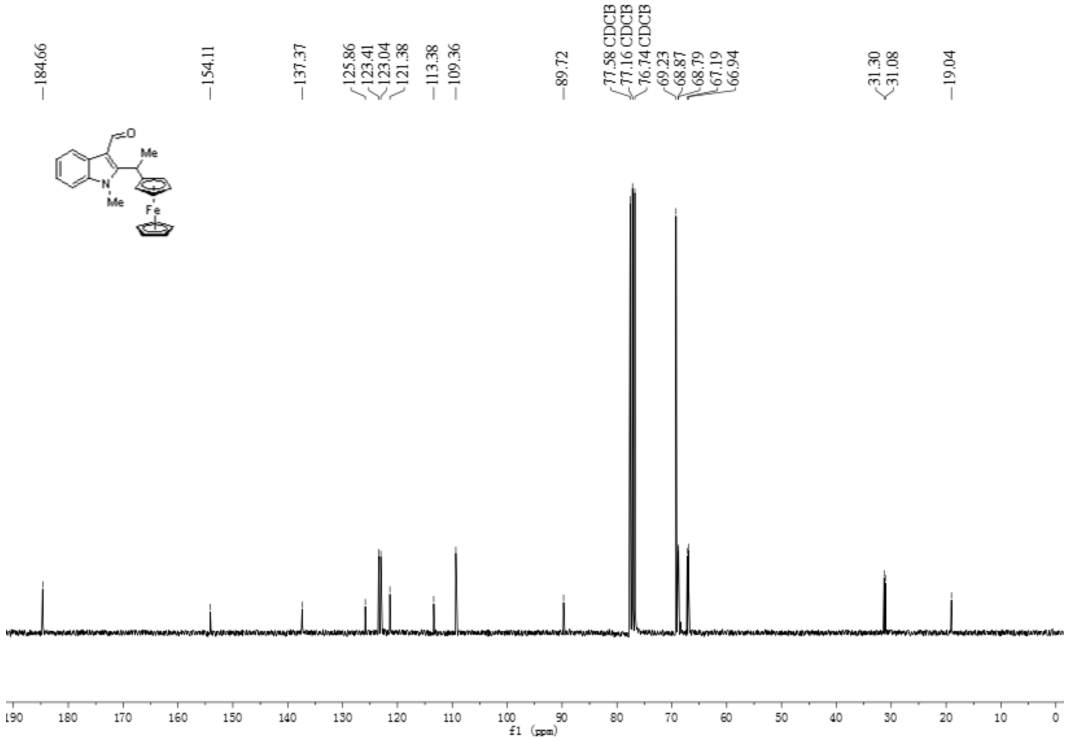

^{1}H-NMR spectrum of **3ab**

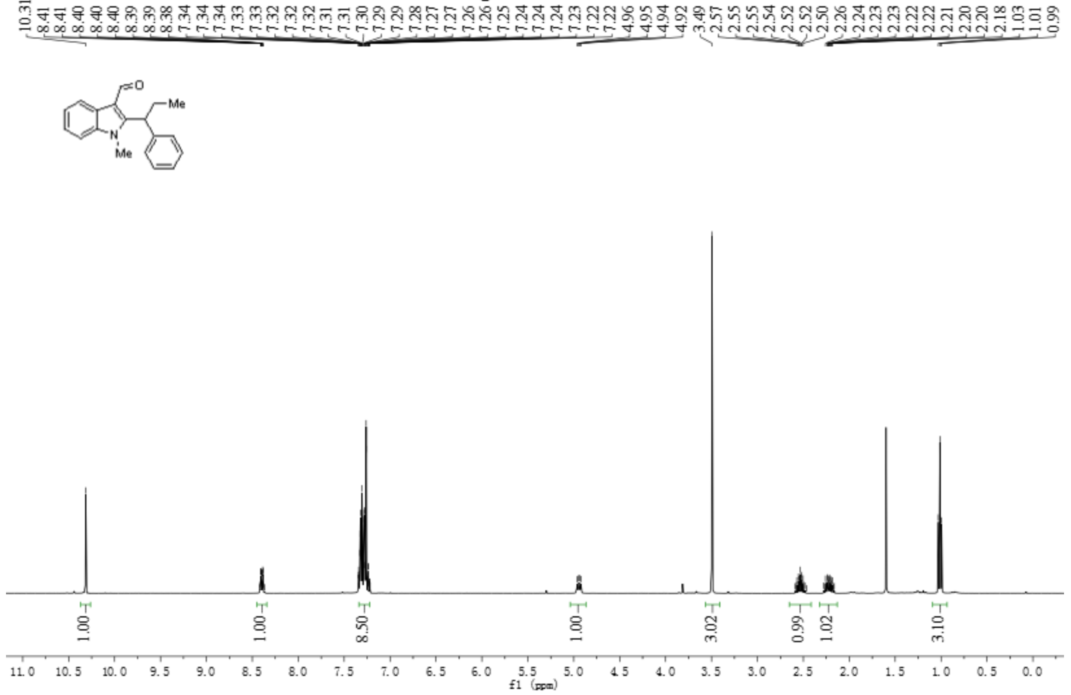

^{13}C-NMR spectrum of **3ab**

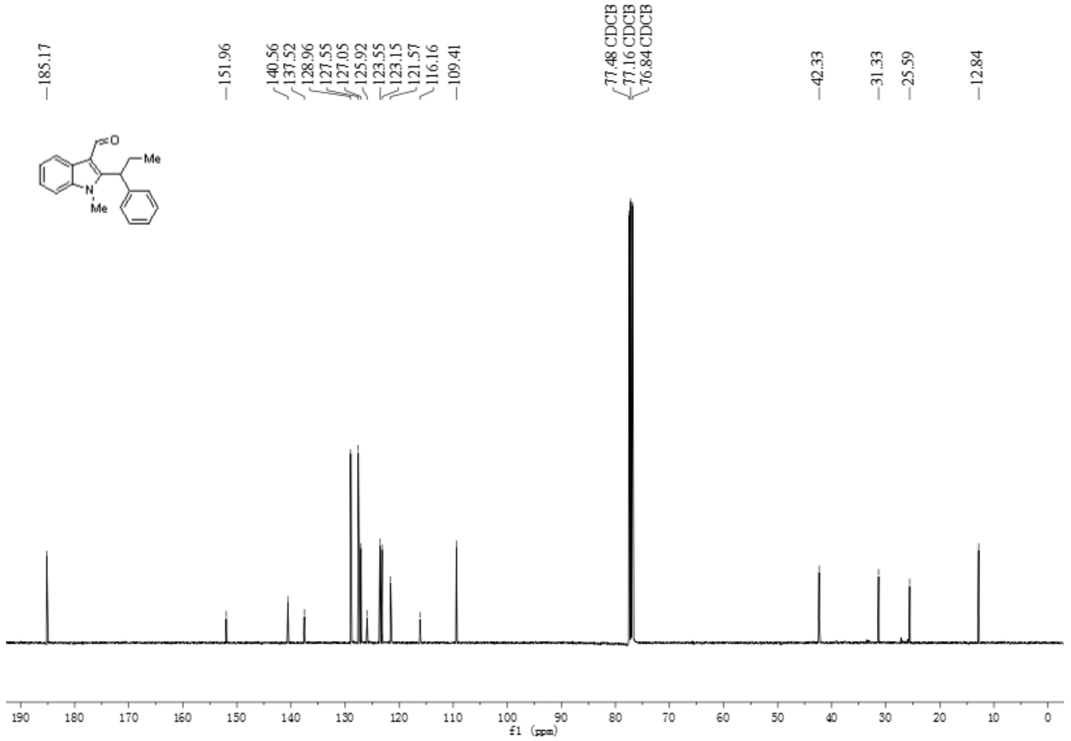

^{1}H-NMR spectrum of **3ac**

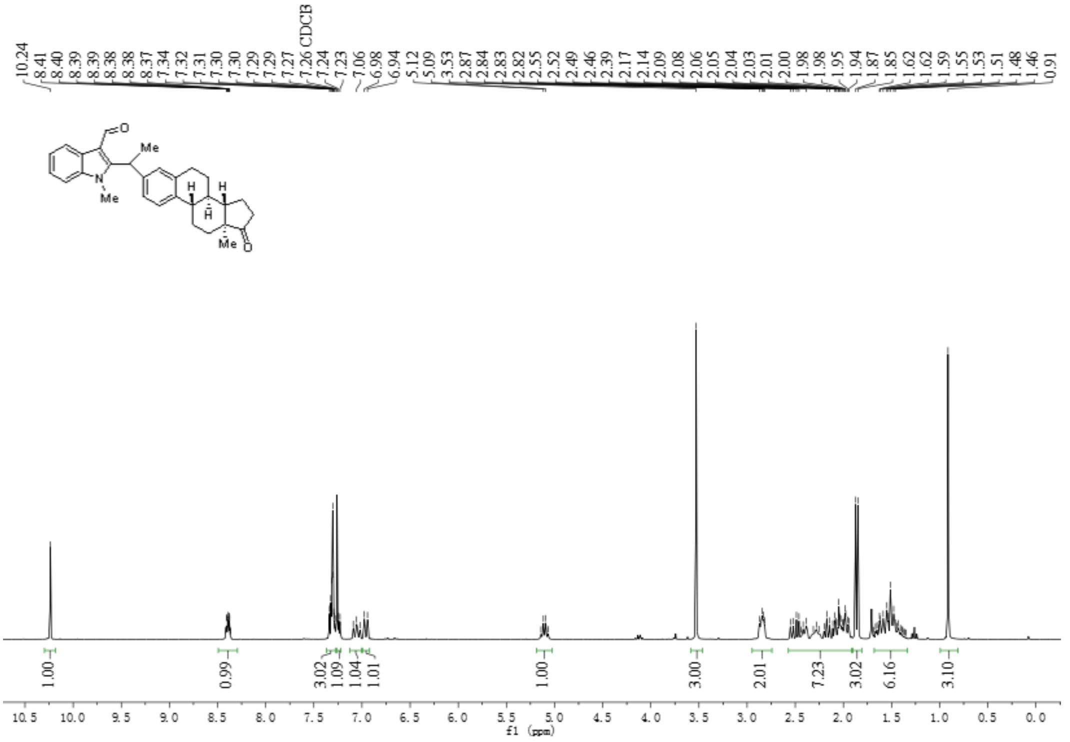

^{13}C-NMR spectrum of **3ac**

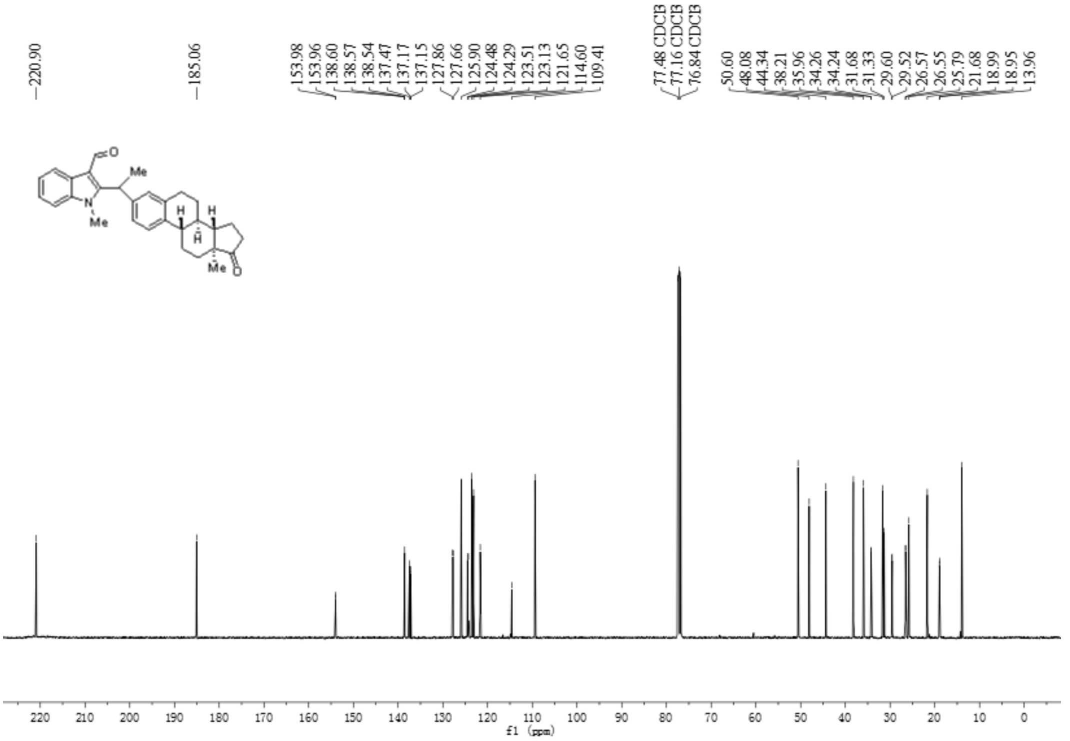

^{1}H-NMR spectrum of **3ad**

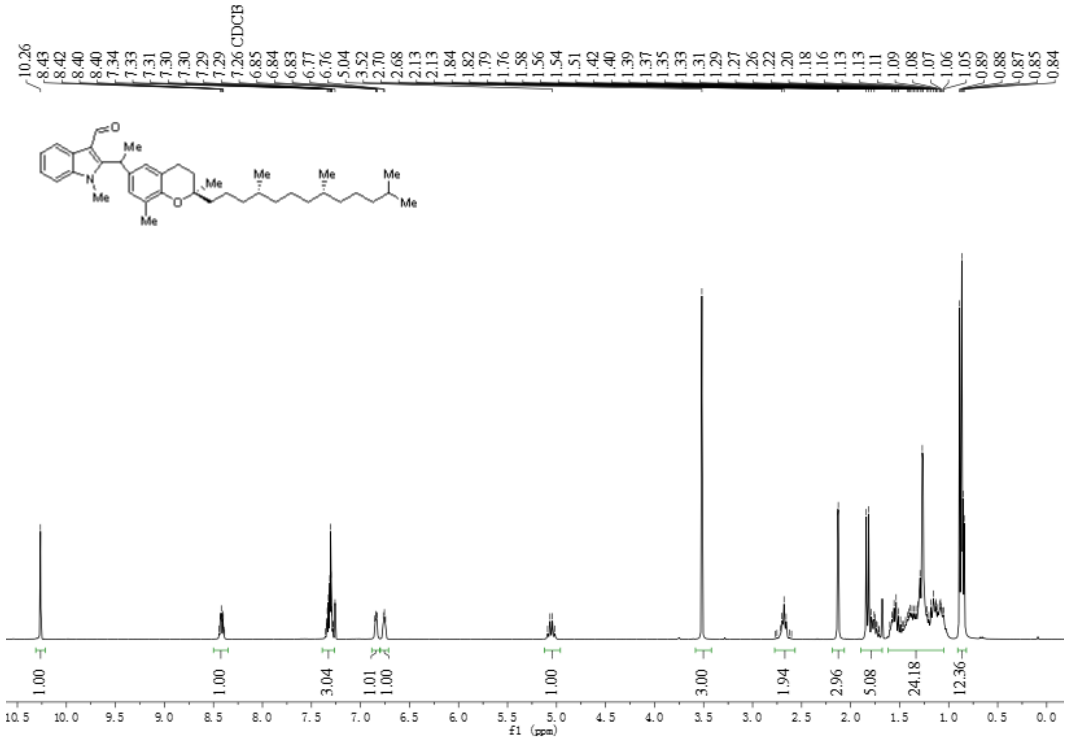

^{13}C-NMR spectrum of **3ad**

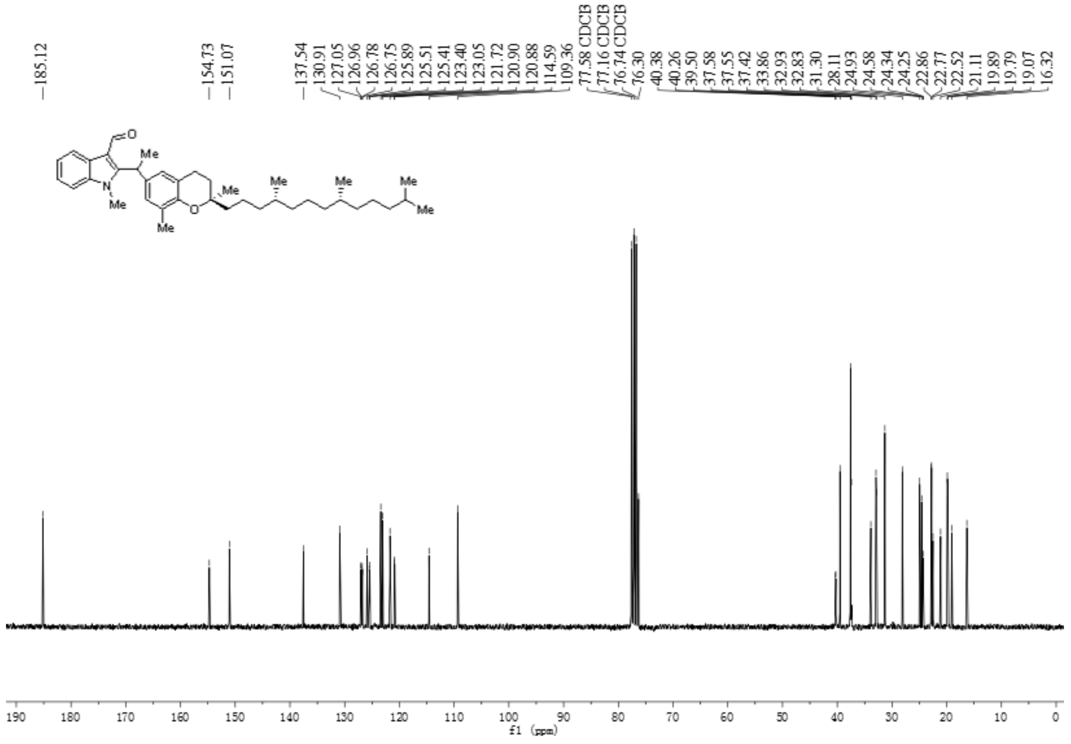

^{1}H-NMR spectrum of **5a**

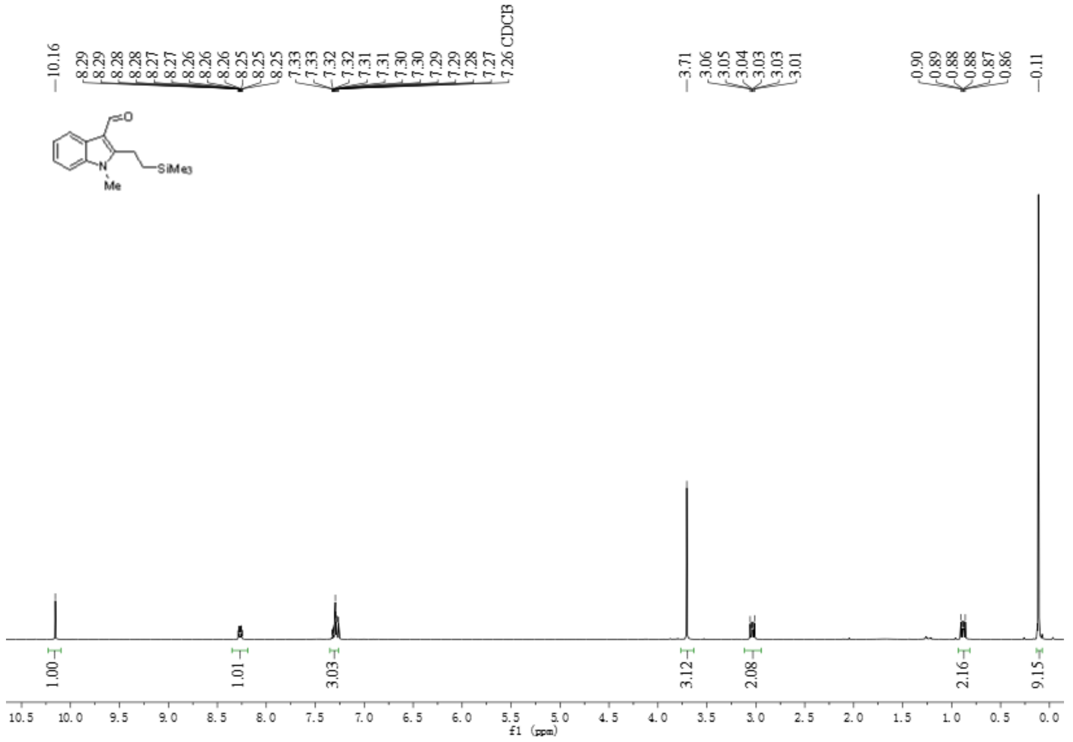

^{13}C-NMR spectrum of **5a**

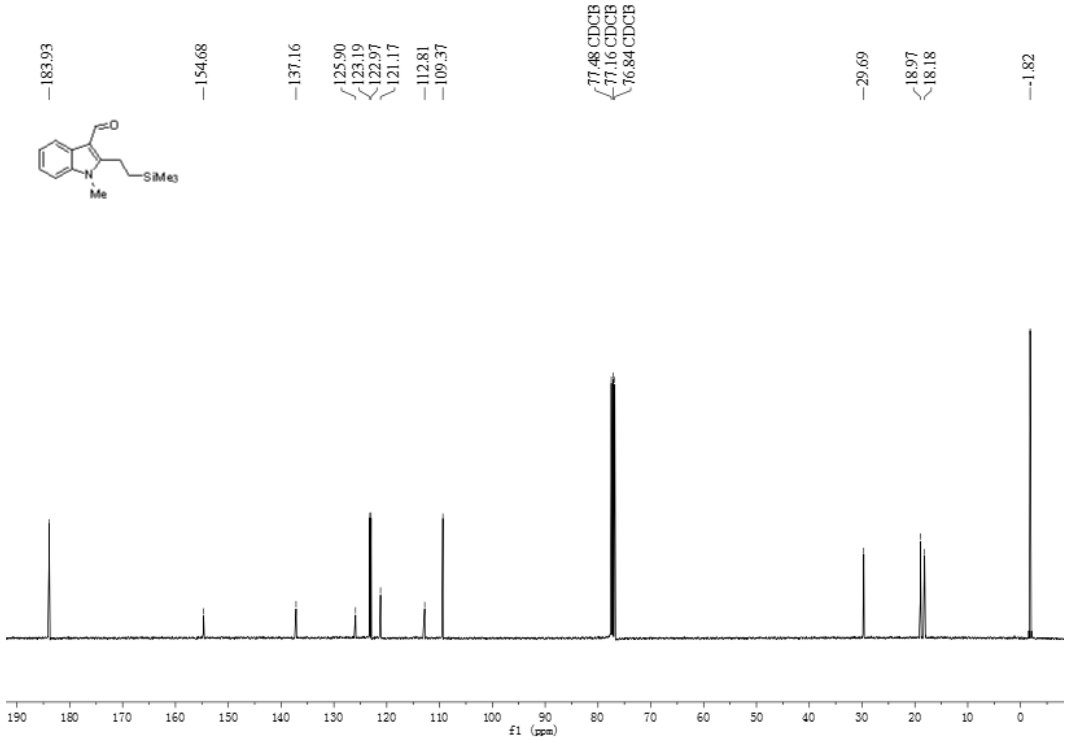

^{1}H-NMR spectrum of **5b**

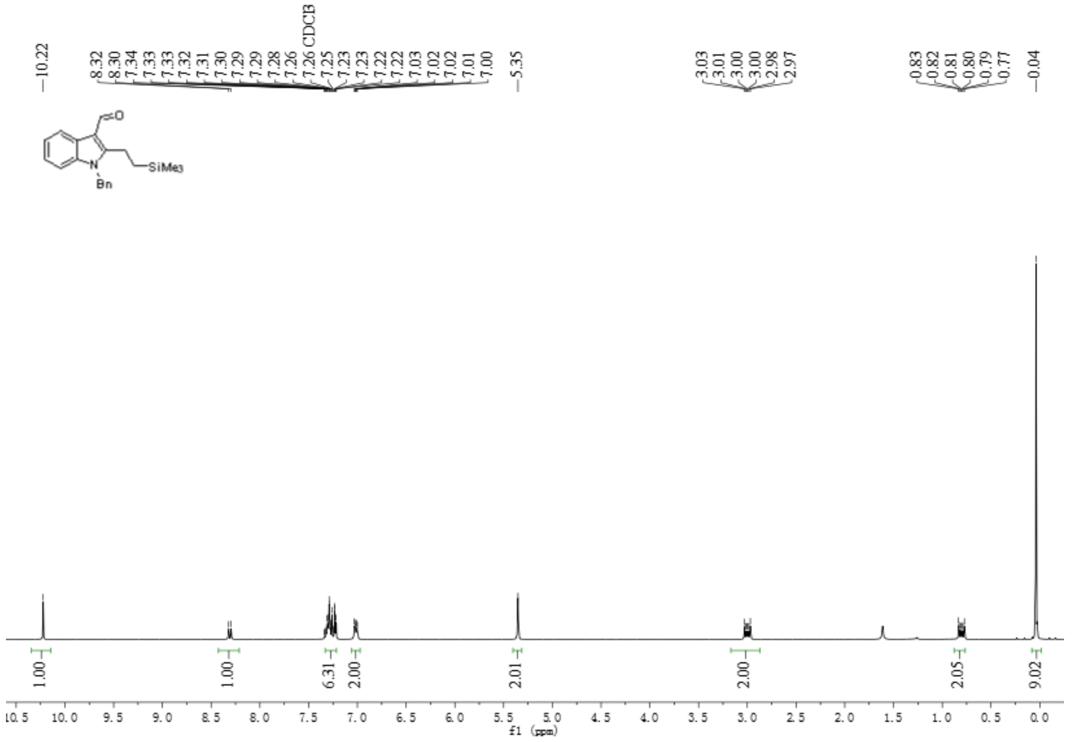

^{13}C-NMR spectrum of **5b**

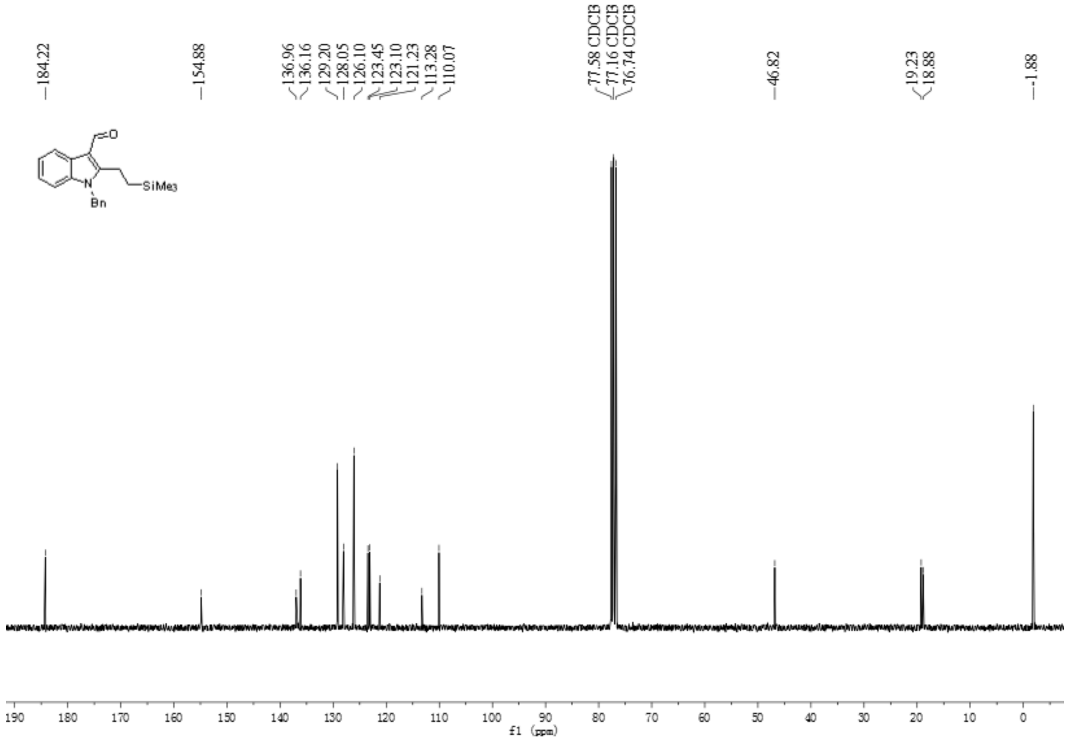

^{1}H-NMR spectrum of **5c**

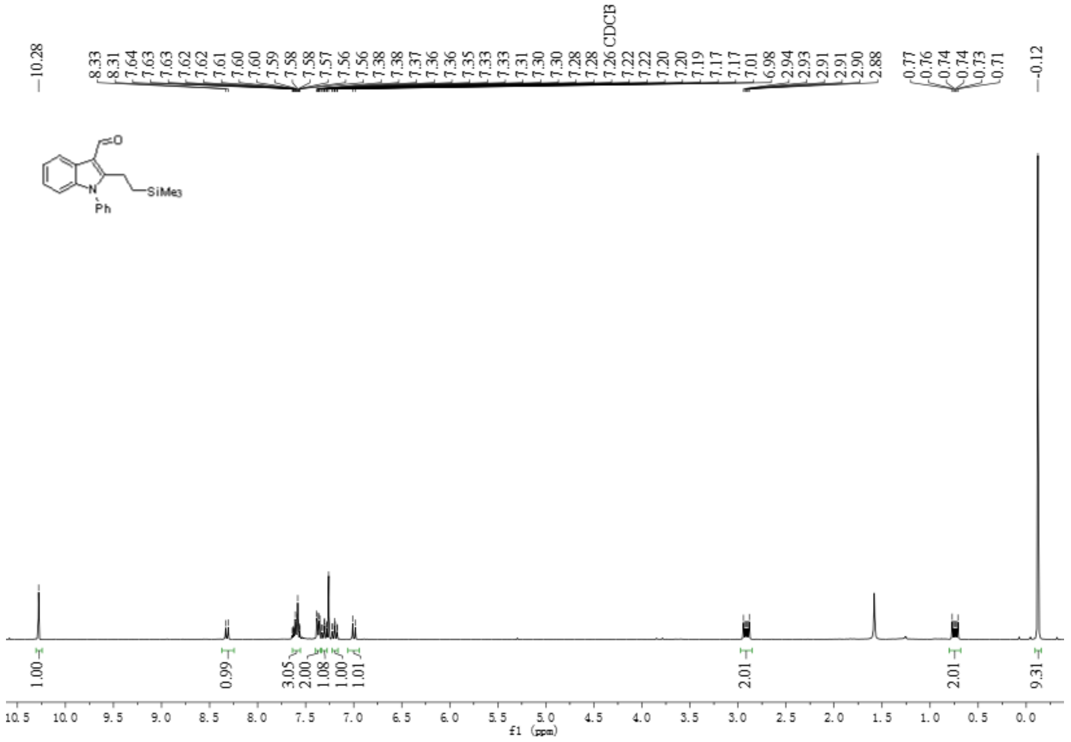

^{13}C-NMR spectrum of **5c**

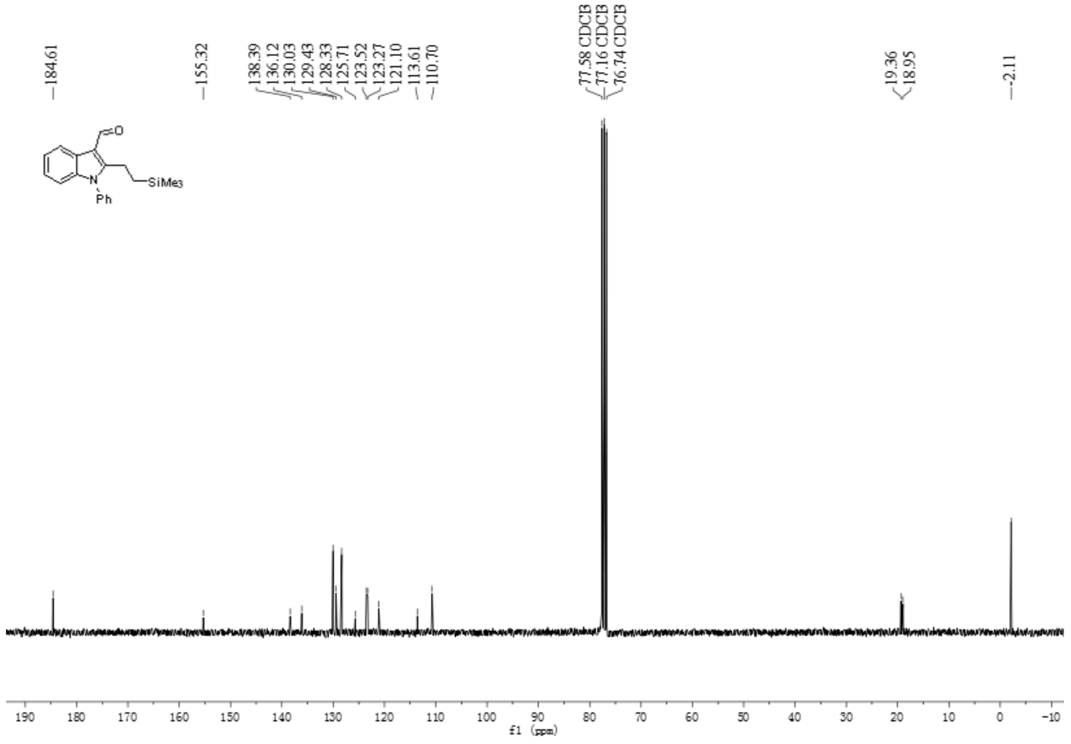

^{1}H-NMR spectrum of **5d**

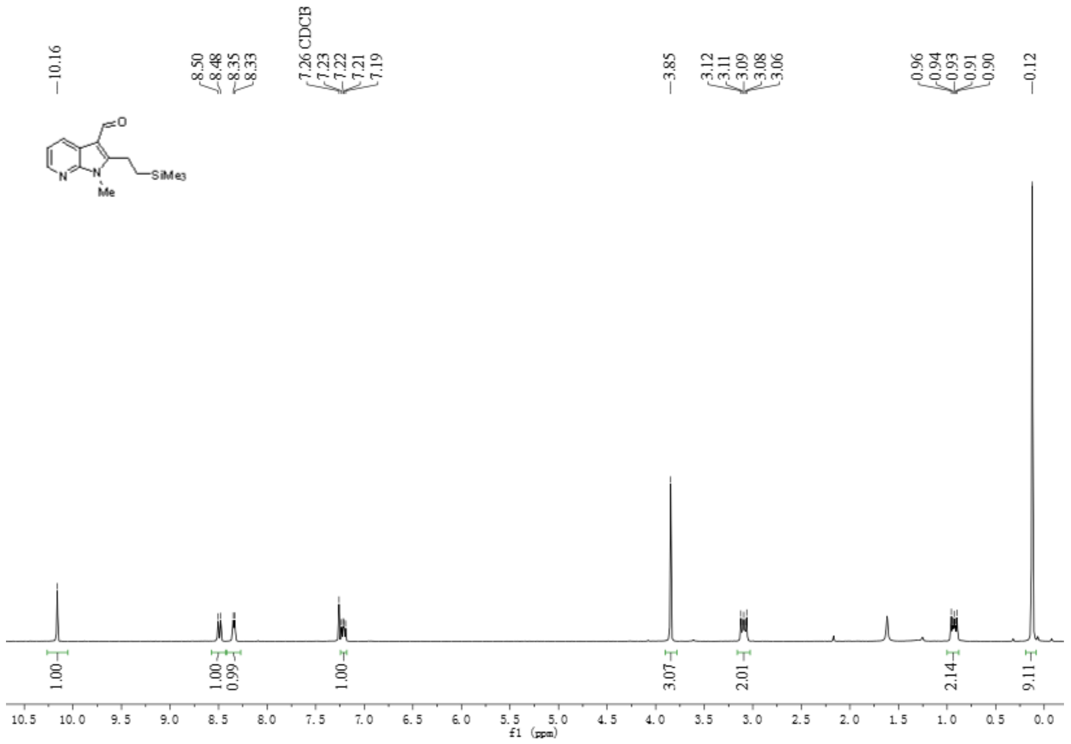

^{13}C-NMR spectrum of **5d**

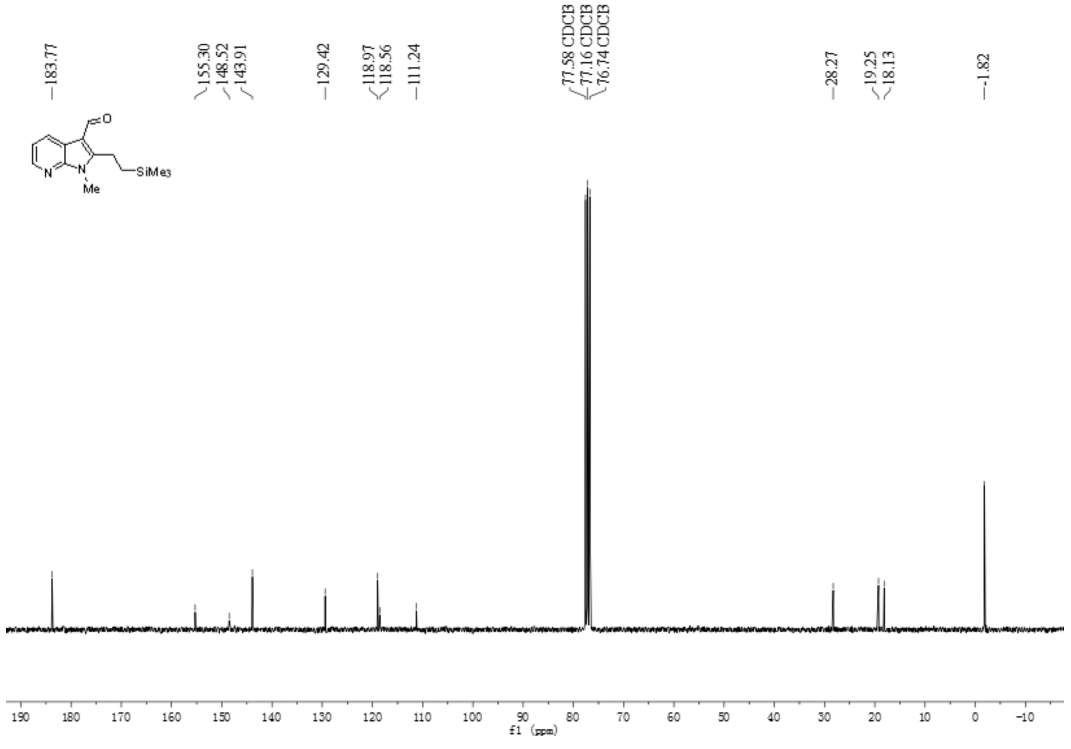

^{1}H-NMR spectrum of **5e**

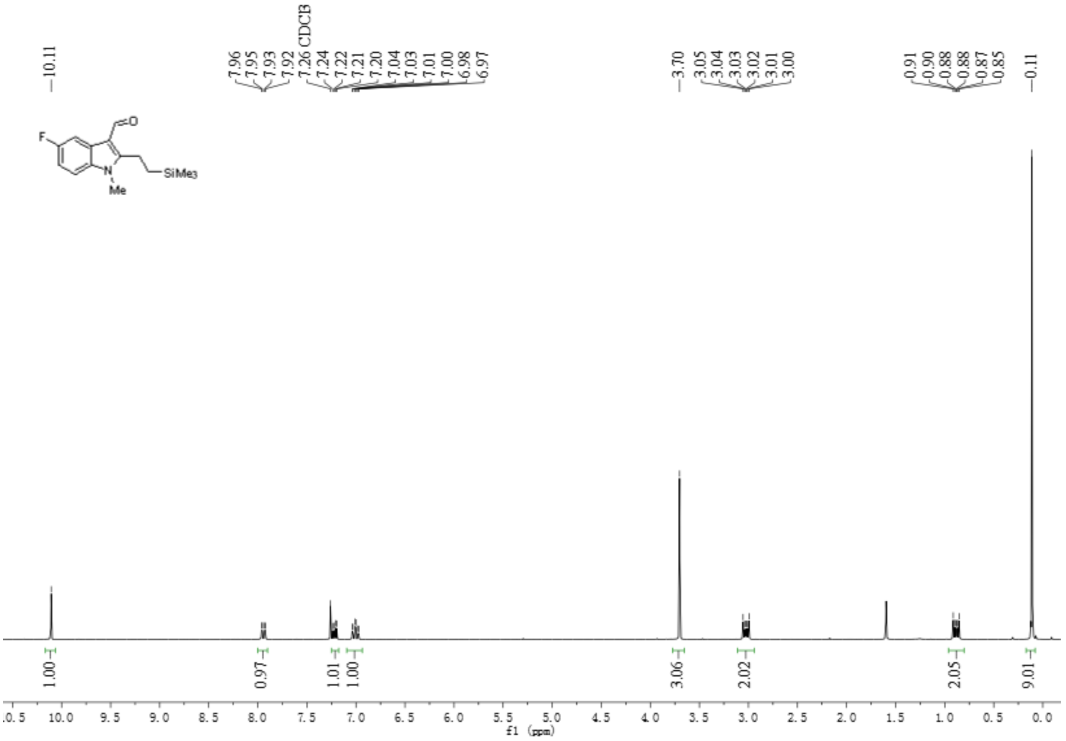

^{13}C-NMR spectrum of **5e**

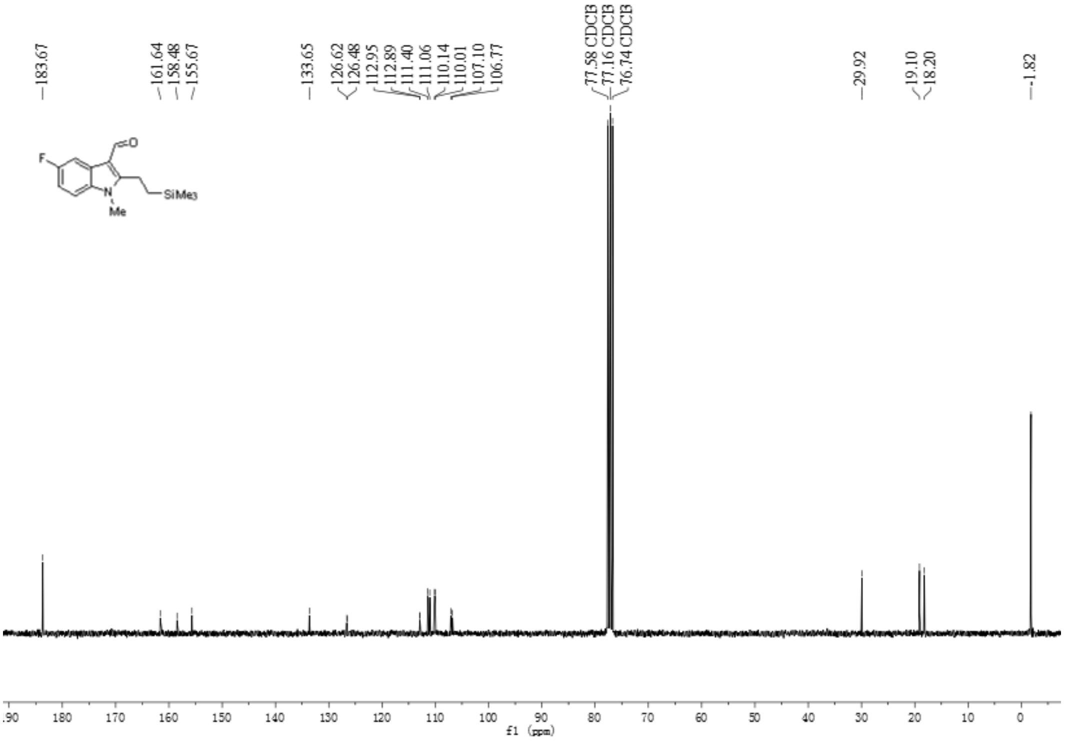

^{19}F-NMR spectrum of **5e**

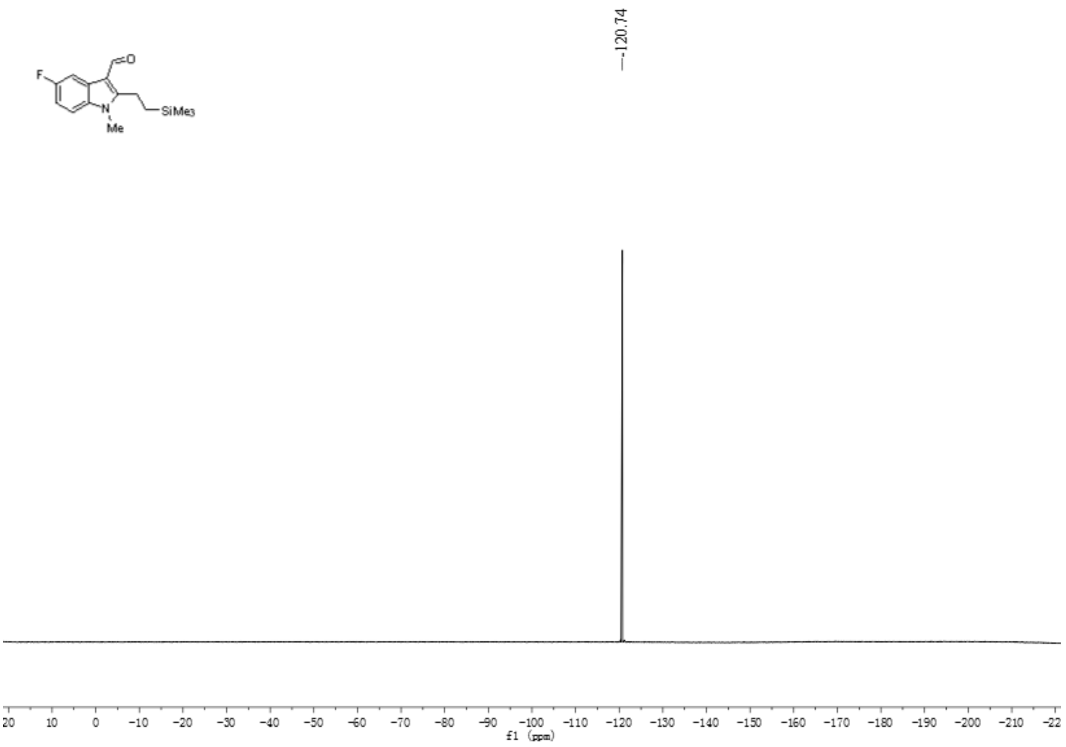

^{1}H-NMR spectrum of **5f**

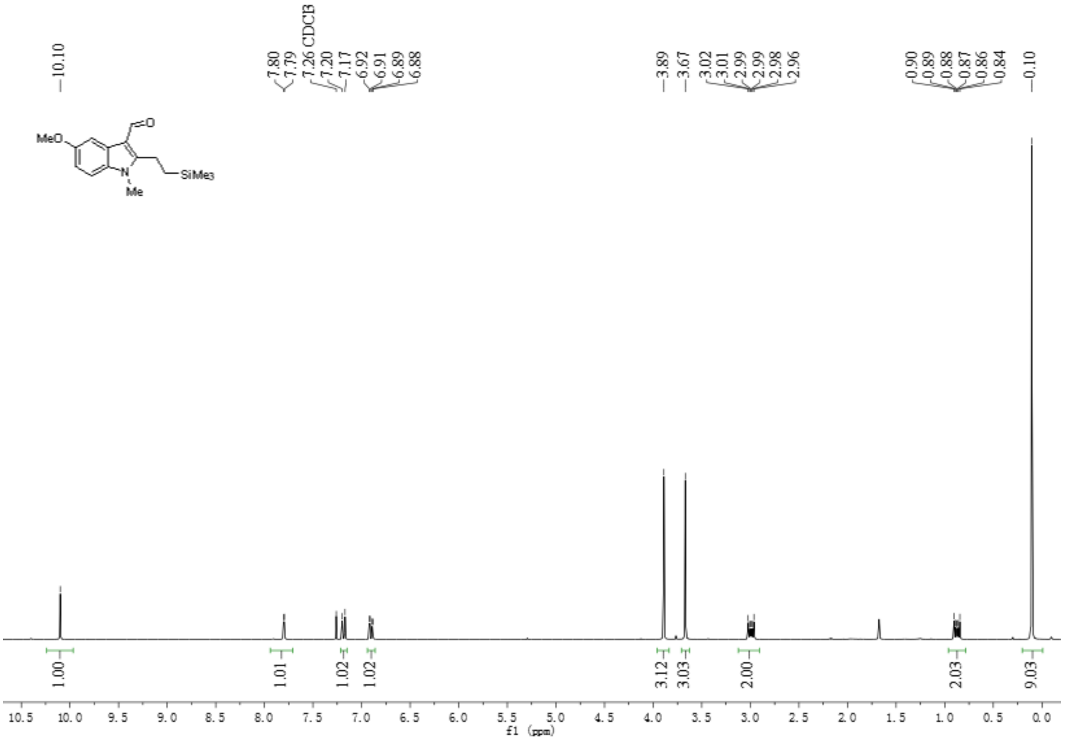

^{13}C-NMR spectrum of **5f**

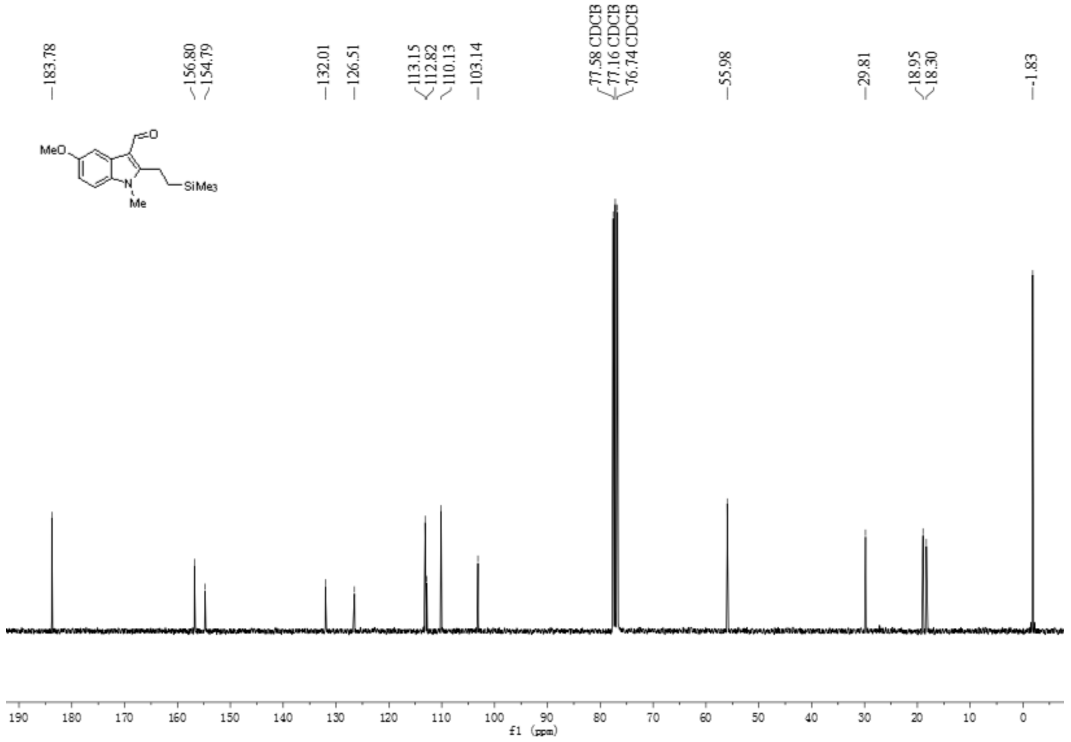

^{1}H-NMR spectrum of **5g**

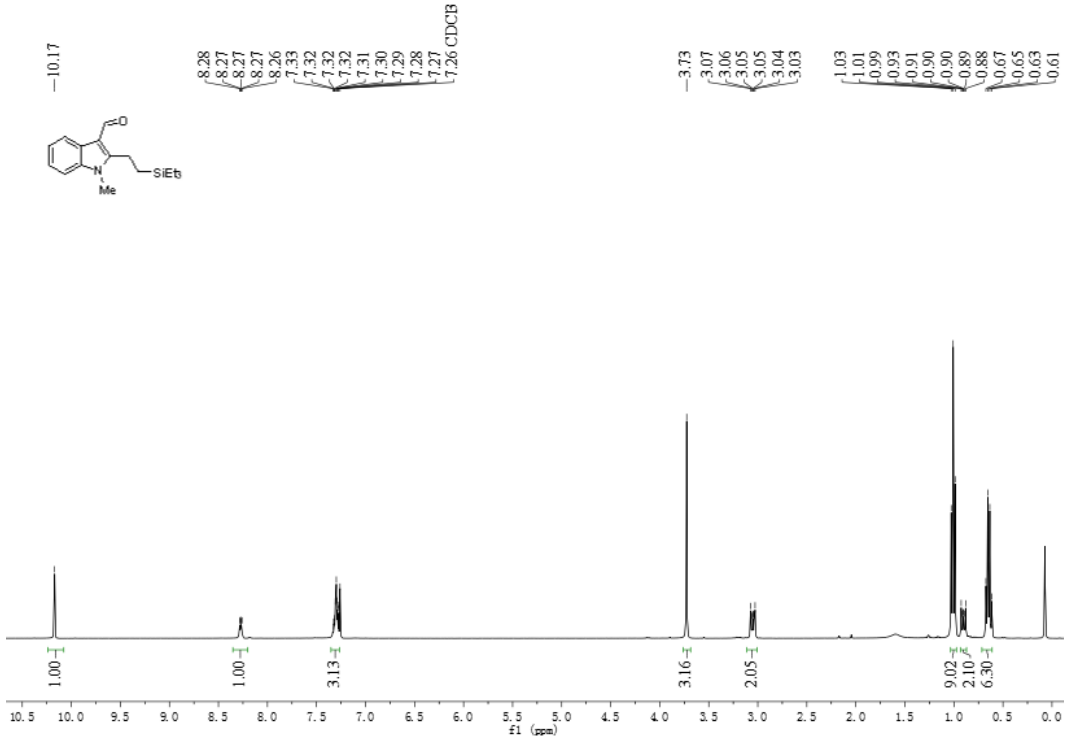

^{13}C-NMR spectrum of **5g**

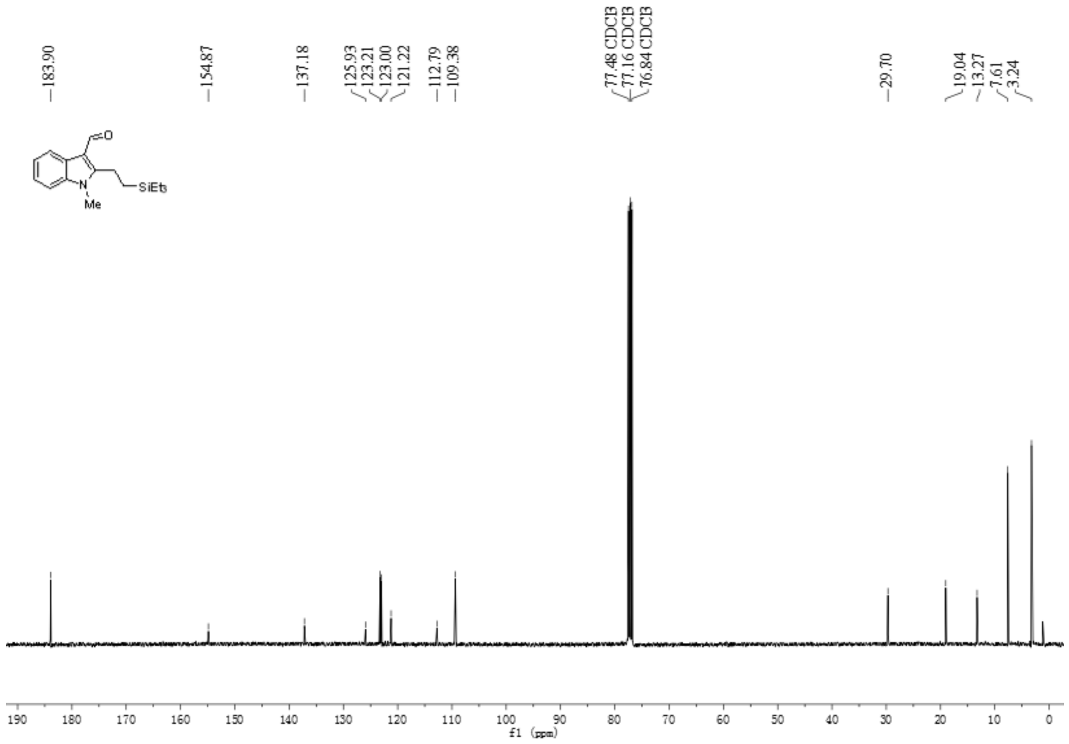

^{1}H-NMR spectrum of **5h**

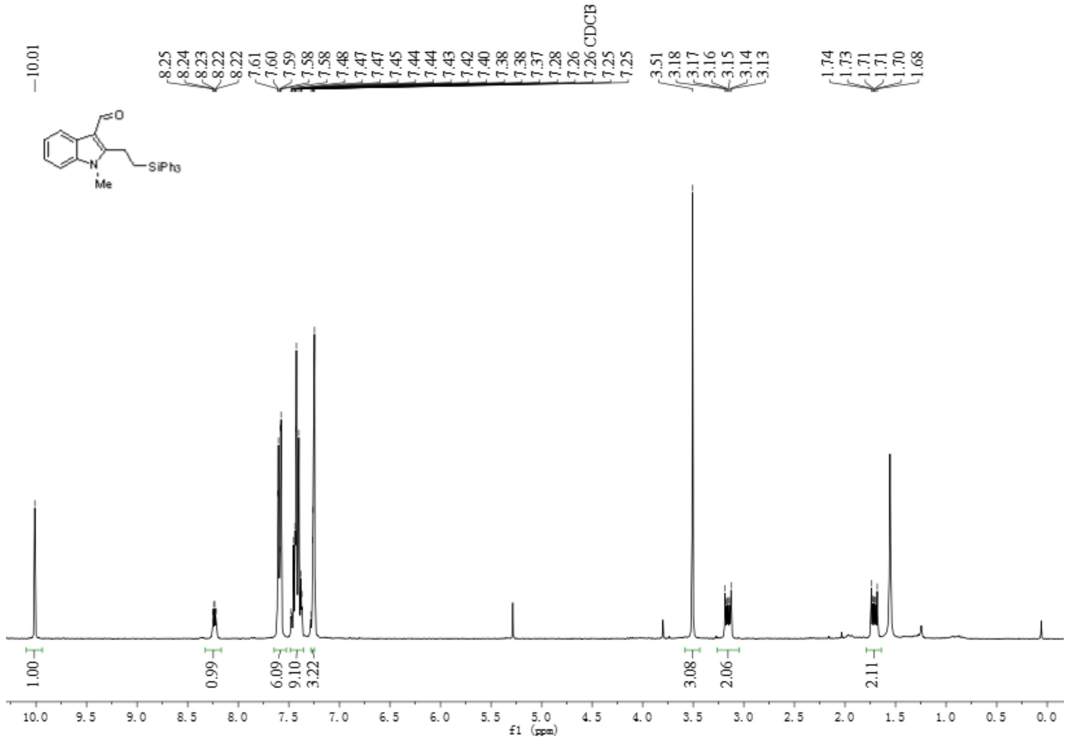

^{13}C-NMR spectrum of **5h**

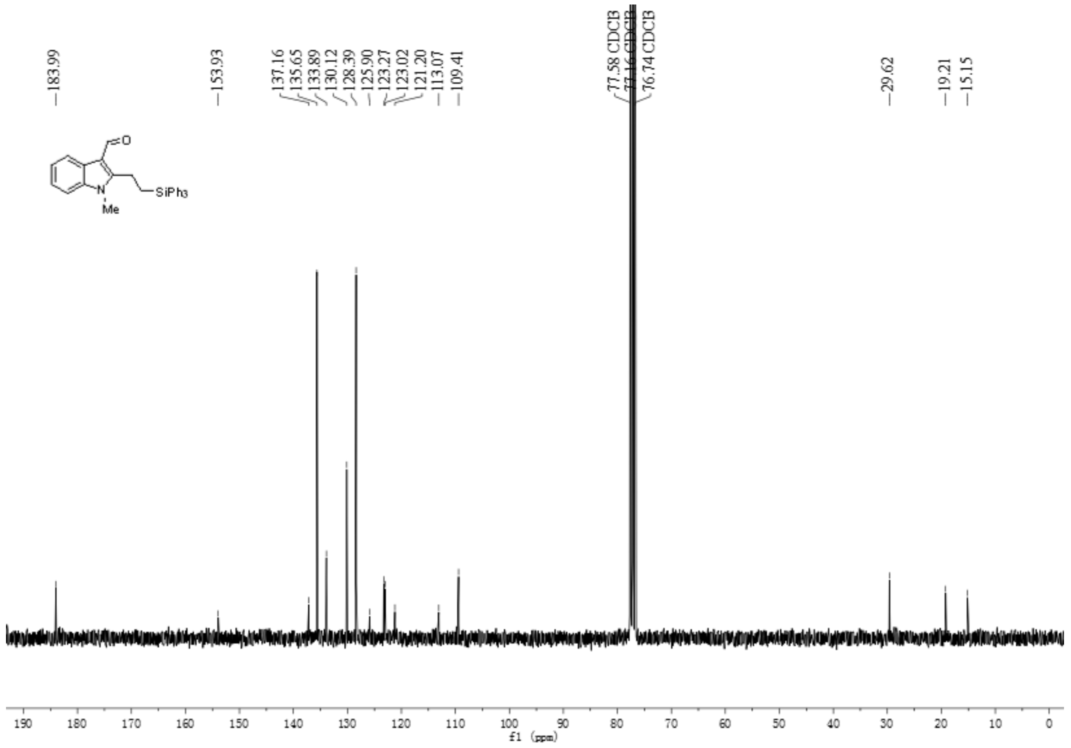

^{1}H-NMR spectrum of **6**

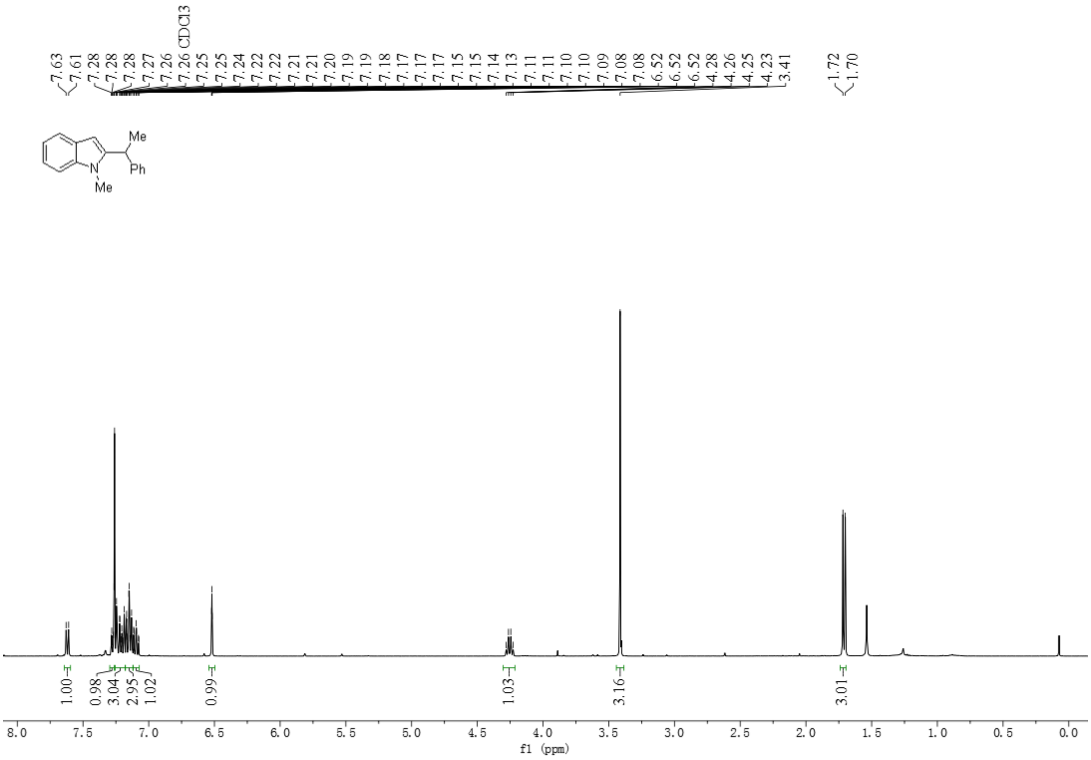

^{13}C-NMR spectrum of **6**

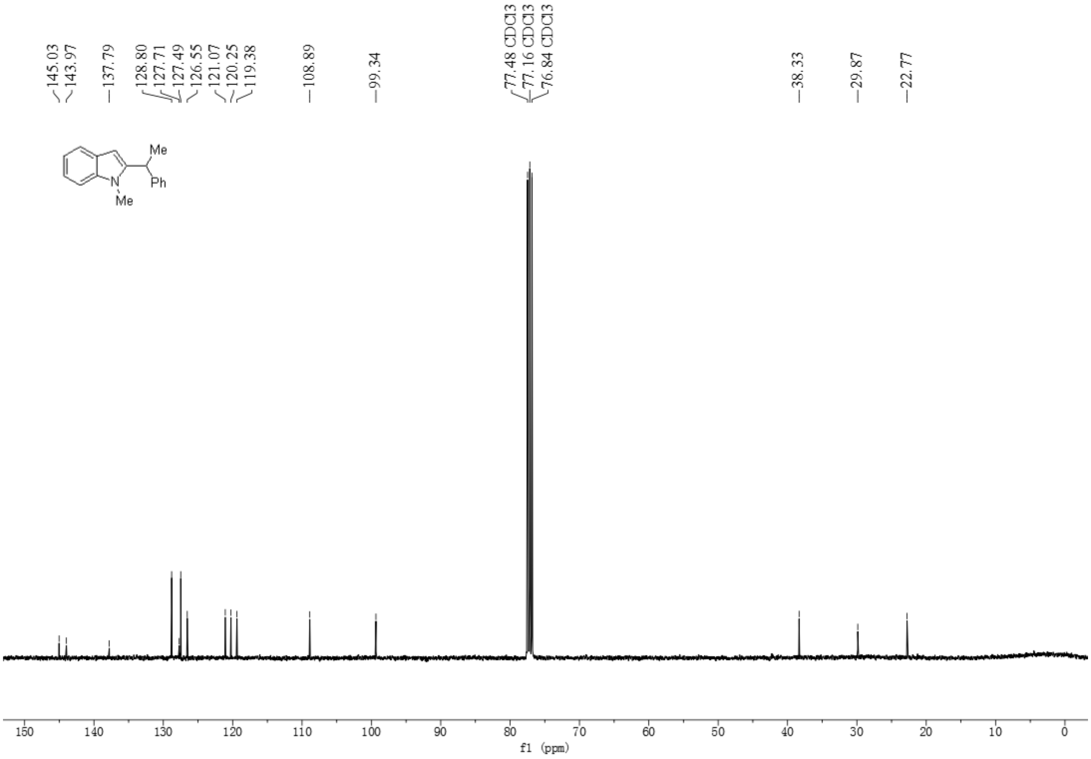

^{1}H-NMR spectrum of **7**

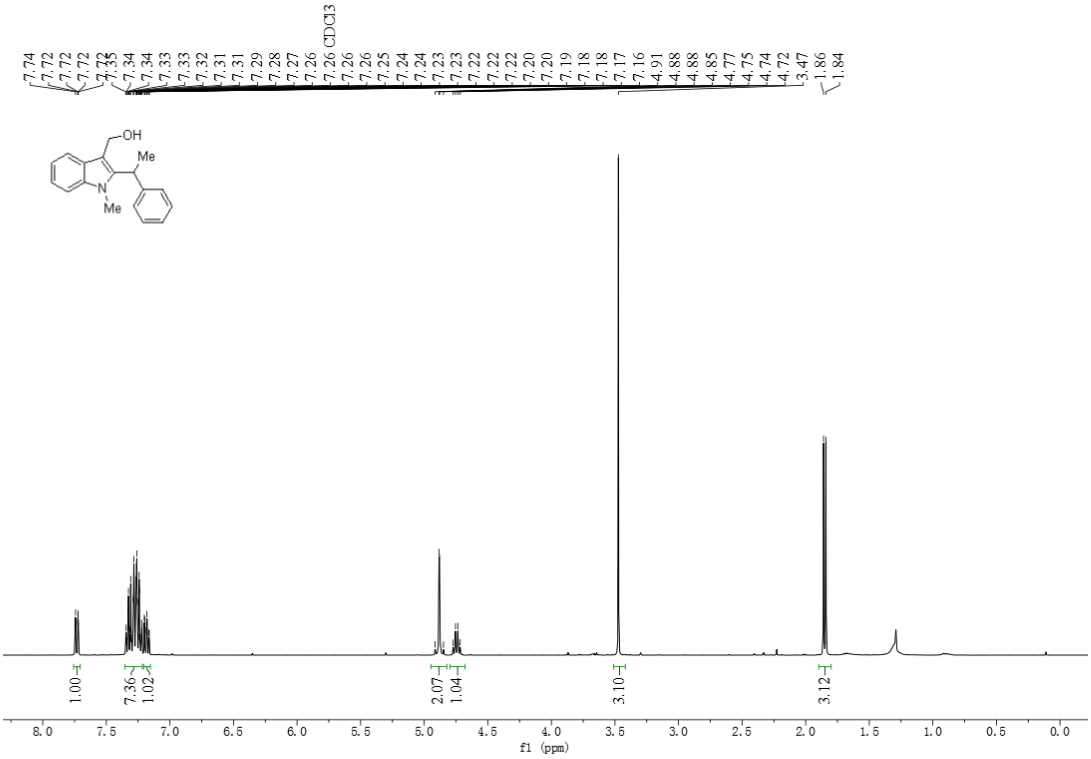

^{13}C-NMR spectrum of **7**

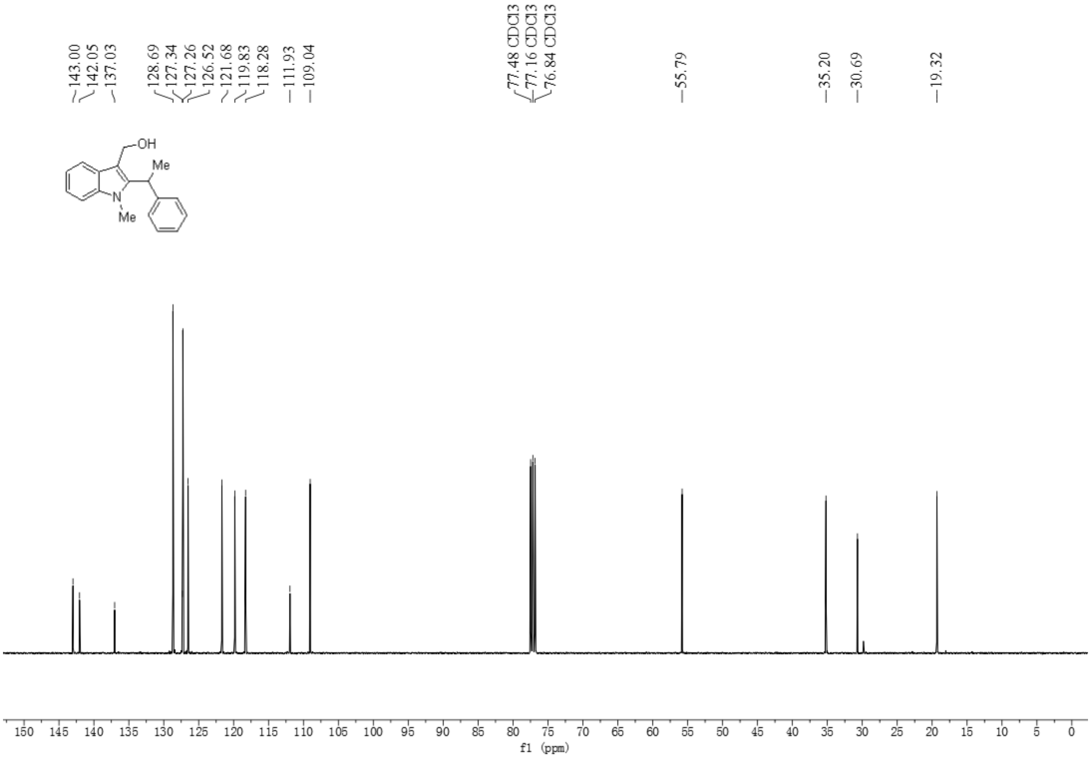

^{1}H-NMR spectrum of **8**

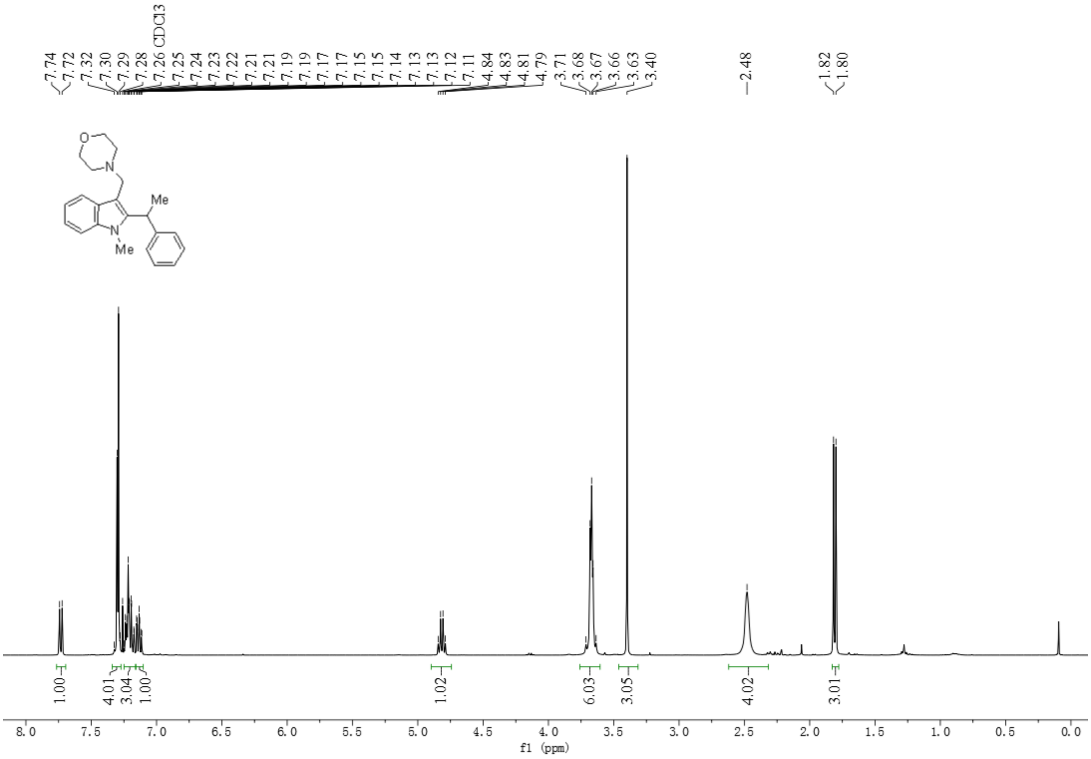

^{13}C-NMR spectrum of **8**

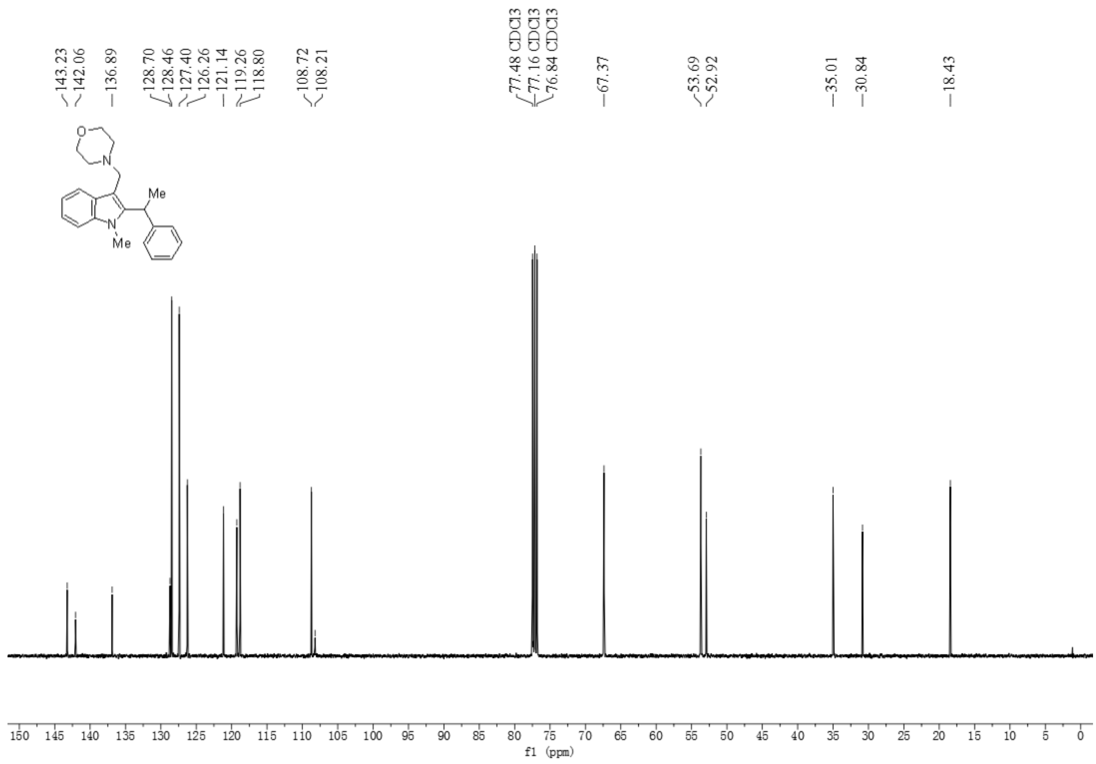

^{1}H-NMR spectrum of **9**

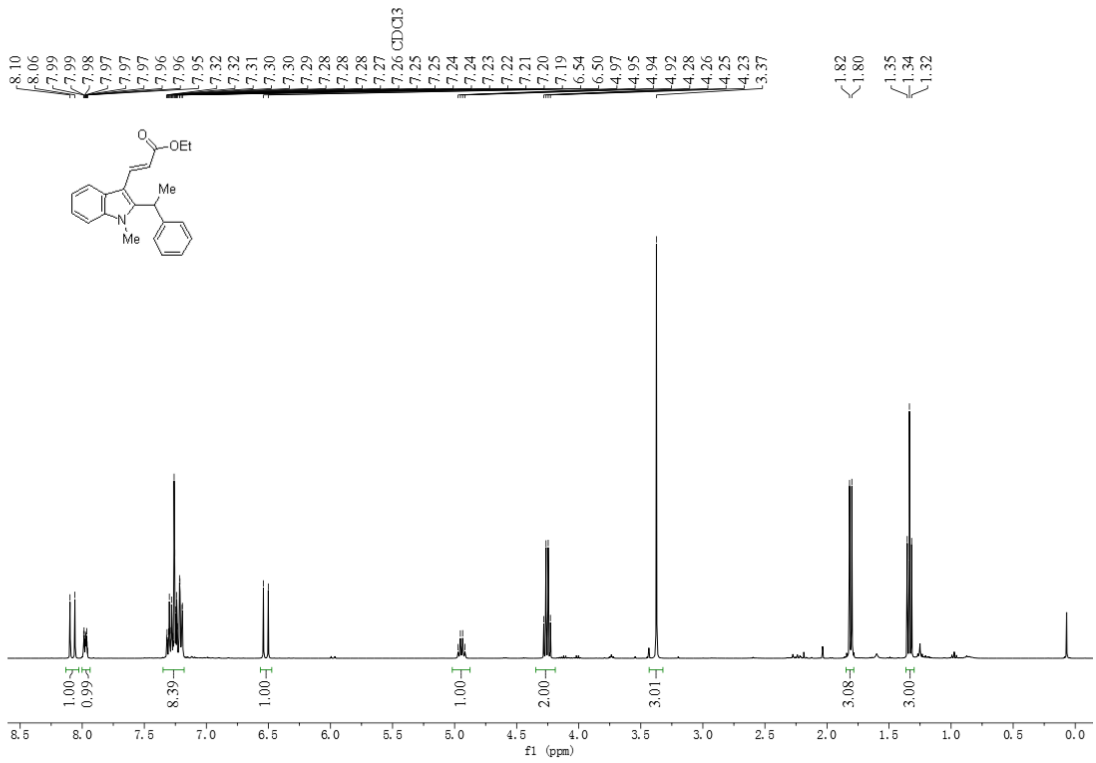

^{13}C-NMR spectrum of **9**

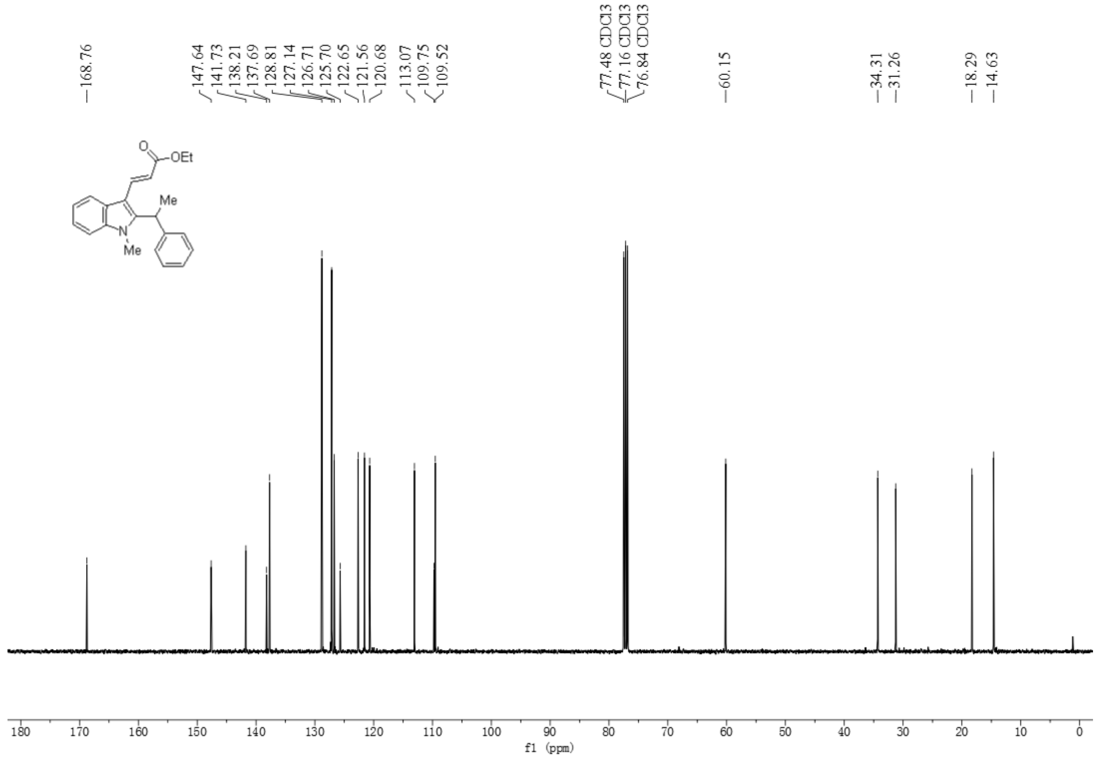

9. References

[1] Wong, M. Y.; Yamakawa, T.; Yoshikai, N. Iron-Catalyzed Directed C2-Alkylation and Alkenylation of Indole with Vinylarenes and Alkynes. *Org. Lett.* **2015**, *17*, 442–445.

[2] Loup, J.; Zell, D.; Oliveira, J. C. A.; Keil, H.; Stalke, D.; Ackermann, L. Asymmetric Iron-Catalyzed C−H Alkylation Enabled by Remote Ligand *meta*-Substitution. *Angew. Chem. Int. Ed.* **2017**, *56*, 14197–14201.

[3] Chen, W.; Chen, Q.; Ma, Y.; Leng, X.; Bai, S.-D.; Deng, L. Formal Co(0), Fe(0), and Mn(0) Complexes with NHC and Styrene Ligation. *Chin. Chem. Lett.* **2020**, *31*, 1342–1344.

[4] Zhang, Z.-J.; Jacob, N.; Bhatia, S.; Boos, P.; Chen, X.; DeMuth, J. C.; Messinis, A. M.; Jei, B. B.; Oliveira, J. C. A.; Radović, A.; Neidig, M. L.; Wencel-Delord, J.; Ackermann, L. Iron-Catalyzed Stereoselective C–H Alkylation for Simultaneous Construction of C–N axial and *C*-Central Chirality. *Nat. Commun.* **2024**, *15*, 3503.

[5] Zhang, H.; Ouyang, Z.; Liu, Y.; Zhang, Q.; Wang, L.; Deng, L. (Aminocarbene)(Divinyltetramethyldisiloxane)Iron(0) Compounds: A Class of Low-Coordinate Iron(0) Reagents. *Angew. Chem. Int. Ed.* **2014**, *53*, 8432–8436.

[6] Cheng, J.; Liu, J.; Leng, X.; Lohmiller, T.; Schnegg, A.; Bill, E.; Ye, S.; Deng, L. A Two-Coordinate Iron(II) Imido Complex with NHC Ligation: Synthesis, Characterization, and Its Diversified Reactivity of Nitrene Transfer and C–H Bond Activation. *Inorg. Chem.* **2019**, *58*, 7634−7644.

[7] Gaussian 16, Revision A.03, Frisch, M. J.; Trucks, G. W.; Schlegel, H. B.; Scuseria, G. E.; Robb, M. A.; Cheeseman, J. R.; Scalmani, G.; Barone, V.; Petersson, G. A.; Nakatsuji, H.; Li, X.; Caricato, M.; Marenich, A. V.; Bloino, J.; Janesko, B. G.; Gomperts, R.; Mennucci, B.; Hratchian, H. P.; Ortiz, J. V.; Izmaylov, A. F.; Sonnenberg, J. L.; Williams-Young, D.; Ding, F.; Lipparini, F.; Egidi, F.; Goings, J.; Peng, B.; Petrone, A.; Henderson, T.; Ranasinghe, D.; Zakrzewski, V. G.; Gao, J.; Rega, N.; Zheng, G.; Liang, W.; Hada, M.; Ehara, M.; Toyota, K.; Fukuda, R.; Hasegawa, J.; Ishida, M.; Nakajima, T.; Honda, Y.; Kitao, O.; Nakai, H.; Vreven,

T.; Throssell, K.; Montgomery, J. A., Jr.; Peralta, J. E.; Ogliaro, F.; Bearpark, M. J.; Heyd, J. J.; Brothers, E. N.; Kudin, K. N.; Staroverov, V. N.; Keith, T. A.; Kobayashi, R.; Normand, J.; Raghavachari, K.; Rendell, A. P.; Burant, J. C.; Iyengar, S. S.; Tomasi, J.; Cossi, M.; Millam, J. M.; Klene, M.; Adamo, C.; Cammi, R.; Ochterski, J. W.; Martin, R. L.; Morokuma, K.; Farkas, O.; Foresman, J. B.; Fox, D. J. Gaussian, Inc., Wallingford CT, 2016.

[8] Becke, A. D. Density-functional thermochemistry. III. The role of exact exchange. *J. Chem. Phys*. **1993**, *98*, 5648−5652.

[9] Lee, C.; Yang, W.; Parr, R. G. Development of the Colle-Salvetti correlation-energy formula into a functional of the electron density. *Phys. Rev. B: Condens. Matter Mater. Phys*. **1998**, *37*, 785−789.

[10] Grimme, S.; Ehrlich, S.; Goerigk, L. Effect of the damping function in dispersion corrected density functional theory. *J. Comp. Chem.* **2011**, *32*, 1456−1465.

[11] Weigend, F.; Ahlrichs, R. Balanced basis sets of split valence, triple zeta valence and quadruple zeta valence quality for H to Rn: Design and assessment of accuracy. *Physical Chemistry Chemical Physics,* **2005**, *7*, 3297–3305.

[12] Tao, J. M.; Perdew, J. P.; Staroverov, V. N.; Scuseria, G. E. Climbing the Density Functional Ladder: Nonempirical Meta–Generalized Gradient Approximation Designed for Molecules and Solids. *Phys. Rev. Lett.* **2003**, *91*, 146401.

[13] Staroverov, V. N.; Scuseria, G. E.; Tao, J.; Perdew, J. P. Comparative assessment of a new nonempirical density functional: Molecules and hydrogen-bonded complexes. *J. Chem. Phys.* **2003**, *119*, 12129.

[14] Marenich, A. V., Cramer, C. J.; Truhlar, D. G. Universal Solvation Model Based on Solute Electron Density and on a Continuum Model of the Solvent Defined by the Bulk Dielectric Constant and Atomic Surface Tensions. *J. Phys. Chem. B.*, **2009**, *113*, 6378−6396.

[15] Legault, C. Y. CYLview, version 1.0b; Université de Sherbrooke, **2009** (http://www.cylview.org).
